# From Propargylic Alcohols to Substituted Thiochromenes: *gem*-Disubstituent Effect in Intramolecular Alkyne Iodo/hydroarylation

**DOI:** 10.1021/acs.joc.1c00333

**Published:** 2021-04-30

**Authors:** Noelia Velasco, Anisley Suárez, Fernando Martínez-Lara, Manuel Ángel Fernández-Rodríguez, Roberto Sanz, Samuel Suárez-Pantiga

**Affiliations:** † Área de Química Orgánica, Departamento de Química, Facultad de Ciencias, Universidad de Burgos, Pza. Misael Bañuelos s/n, 09001 Burgos, Spain; ‡ Departamento de Química Orgánica y Química Inorgánica, Instituto de Investigación Química “Andrés M. del Río” (IQAR), Universidad de Alcalá (IRYCIS), 28805 Alcalá de Henares, Madrid, Spain

## Abstract

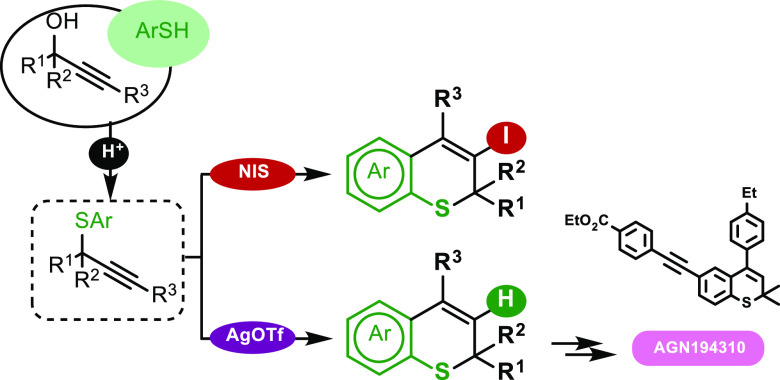

This work describes the 6-*endo*-*dig* cyclization of *S*-aryl propargyl sulfides to afford 2*H*-thiochromenes. The substitution at the propargylic position plays a crucial role in allowing intramolecular silver-catalyzed alkyne hydroarylation and *N*-iodosuccinimide-promoted iodoarylation. Additionally, a PTSA-catalyzed thiolation reaction of propargylic alcohols was developed to synthesize the required tertiary *S*-aryl propargyl ethers. The applicability of merging these two methods is demonstrated by synthesizing the retinoic acid receptor antagonist AGN194310.

## Introduction

**Scheme 1:**
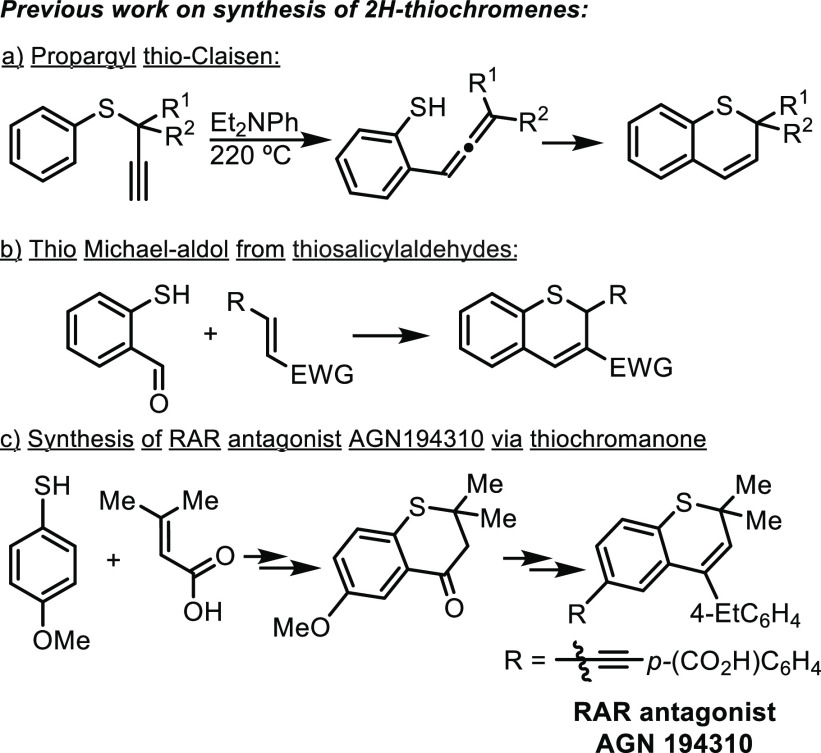
Selected Methodologies for Synthesizing 2*H*-Thiochromenes

Propargyl *N*-aryl amines and *O*-aryl ethers are versatile and precious building blocks.^1^ The intramolecular alkyne arylation^2^ of these building blocks provides straightforward access to relevant heterocycles such as hydroquinolines and chromenes,^3,4^ without requiring a previous arene *ortho* functionalization. By contrast, this strategy involving C–H bond functionalization remains almost unexplored when applied to the synthesis of 2*H*-thiocromenes from the related thioethers.^5^ Intramolecular alkyne hydroarylation of *S*-aryl propargyl thioethers has been scarcely reported and limited to propargyl Claisen rearrangement using terminal alkynes under harsh reaction conditions (Scheme 1a).^6^ This rearrangement delivers a reactive allene intermediate^7^ that evolves into the thiochromene. Therefore, the synthesis of substituted 2*H*-thiochromenes is commonly accomplished by using thiosalicylaldehydes and alkenes in the presence of an organocatalyst (Scheme 1b).^8^

Alternative classical strategies that do not require *ortho* prefunctionalization are based on multistep sequences to afford a 4-thiochromanone intermediate.^9^ Subsequent alcohol formation followed by elimination generates the 2*H*-thiochromene. This strategy has been employed to synthesize retinoic acid receptor (RAR) antagonist AGN194310 (Scheme 1c).^10^ Therefore, a more straightforward synthesis of thiochromenes through alkyne arylation of *S*-aryl propargyl thioethers under mild reaction conditions will be highly appealing.

**Scheme 2:**
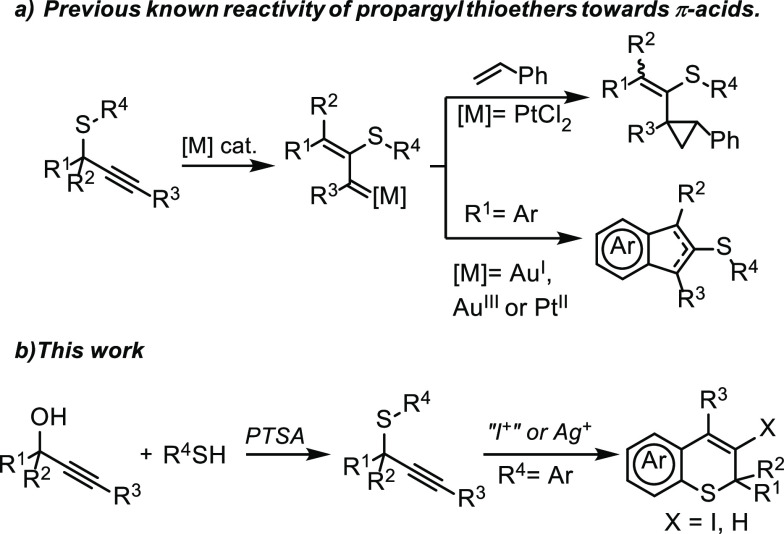
Alternative Reactivity of Propargyl Sulfides with π-Acid Metal Catalysts

The lack of electrophilic alkyne arylation methodologies of *S*-aryl propargyl thioethers is particularly surprising. A possible explanation could be that the sulfur atom favors competitive reaction pathways other than alkyne arylation. For instance, *S*-phenyl alkyl thioethers react with halonium ions enabling desulfurative halogenation reactions.^11^ Additionally, in a seminal contribution of the activation of propargyl thioethers by π-acids,^12^ Wang has reported a thiirenium ion formation after alkyne activation (Scheme 2a). Then, this intermediate evolves through 1,2 migration of the thio group to generate highly reactive metal–carbene species.^13^ Considering these reports, we hypothesized that tertiary propargyl sulfides, because of the *gem*-dimethyl effect,^14^ could react with an adequate alkynophilic reagent affording 2*H*-thiochromenes by favoring electrophilic alkyne arylation over other competitive pathways. Herein, we report the synthesis of 2*H*-thiochromenes through iodocyclization or hydroarylation of *S*-aryl propargyl thioethers (Scheme 2b).

## Results and Discussion

### Synthesis of Tertiary *S*-Aryl Propargyl Thioethers

**Scheme 3:**
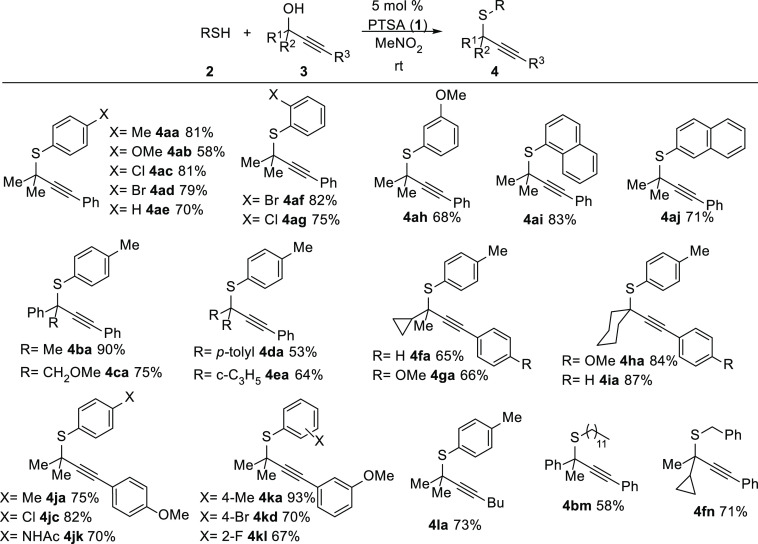
Synthesis of Propargyl Thioethers **4** from Thiols **2** and Tertiary Propargyl Alcohols **3**

To tackle our proposed idea, we need to start from tertiary *S*-aryl propargyl thioethers. However, few examples for synthesizing these types of compounds bearing a quaternary center at the propargylic position have been reported. Despite the advances achieved in the thiolation of propargyl alcohols,^5,15^ mainly secondary propargylic alcohols have been used. Although an acid catalyst can easily activate tertiary alcohols, the propargylic substitution reaction is more challenging. The generated tertiary propargyl cation intermediate, which could be more adequately represented as an allenium ion, evolves rapidly in different alternative reaction pathways from propargylic substitution, such as competitive elimination or S_N_′ reactions forming allenes.^16^ Moreover, thiols could react with propargylic alcohols through other different reactivity patterns that do not implicate a carbocation at the propargylic position, like hydrothiolation of alkynes.^17^ Whereas thiolation of tertiary propargyl alcohols was efficiently accomplished using alkyl thiols,^15a,15c,15f^ with less nucleophilic thiophenols the reaction takes place with low yields.^12^ Remarkably, in two recent reports, few examples of *S*-aryl propargyl thioethers were efficiently synthesized from tertiary propargyl alcohols and an excess of thiophenol (2–3 equiv) by using catalytic amounts of a bimetallic Ir–Sn complex^18^ or a lithium triflimidate salt.^19^ To this end, and on the basis of our previous experience in the direct nucleophilic substitutions of propargylic alcohols,^15c,16,20^ we evaluated the propargylation of thiophenols employing *p*-toluenesulfonic acid monohydrate (**1**, PTSA) as a promising, cheap, and easily accessible catalyst. After some optimization,^21^ this simple Brønsted acid proved to be an efficient catalyst for accomplishing thiolation of a variety of tertiary propargylic alcohols **3** with different thiols **2** (Scheme 3). When dimethyl-substituted propargylic alcohol **3a** was tested, under standard conditions using 5 mol % **1** in nitromethane, the desired *S*-aryl propargyl thioether **4aa** was obtained in high yields.

The reaction seemed to be quite general with various thioarenes bearing different electron-donating, neutral, and moderate electron-withdrawing substituents at the *para* position (**4ab–ae**, **4jc**, **4jk**, and **4kd**), although a slightly lower yield was observed with a methoxy substituent (**4ab**). Functional groups at the *ortho* (**4af**, **4ag**, and **4kl**) or *meta* (**4ah**) position were well tolerated. Interestingly, thionaphthols (**4ai** and **4aj**) also underwent the PTSA-catalyzed thiolation reaction. Next, we studied different alcohols. Modifications over a methyl group at the propargylic position revealed that phenyl (**4ba**) and vicinal (**4ca**) methoxy groups were compatible with the nucleophilic substitution reaction. Diaryl- and dicyclopropyl-substituted tertiary alcohols proved to be more challenging, though the desired propargyl thioethers **4da** and **4ea** were accessible. Other alcohols bearing one cyclopropyl substituent at the propargylic position also reacted with thiols, providing the desired products (**4fa** and **4ga**). Propargylic alcohols **3** derived from cyclohexanone performed well, affording the corresponding thioethers in high yields (**4ha** and **4ia**). Substrates bearing methoxy groups in the arene moiety R^3^ were suitable substrates for the thiolation reaction with different thioarenes (**4ja**, **4jc**, **4jk**, **4ka**, **4kd**, and **4kl**). Curiously, a methoxy group at the *meta* position of the arene attached to the alkyne (**4ka**) provides higher yields than the related substrate bearing a methoxy group at the *para* position (**4aj**). Presumably, this moderate difference might be caused by a stronger stabilization of the carbocation intermediate that slows the nucleophilic attack and allows competitive reaction pathways.^22^ The method was also productive with tertiary alcohols bearing an alkyl group as the alkyne substituent (**4la**) as well as with alkyl thiols (**4bm** and **4fn**). In addition, to demonstrate the practicability of the method, the reaction was scaled up, affording gram amounts of **4aa** (3.78 g, 71% yield) and **4ad** (5.21 g, 79% yield).

### Synthesis of 2*H*-Thiochromenes

Once we established an efficient and easily scalable methodology for synthesizing *S*-aryl propargyl thioethers, we investigated an intramolecular arylation procedure to obtain the desired 2*H*-thiochromenes. We nonetheless have foreseen that a combination of a suitable electrophilic agent with adequate *S*-aryl propargyl thioethers will be decisive in achieving the alkyne arylation. As we have already postulated, the Thorpe–Ingold effect could favor the 6-*endo-dig* cyclization.

**Scheme 4:**
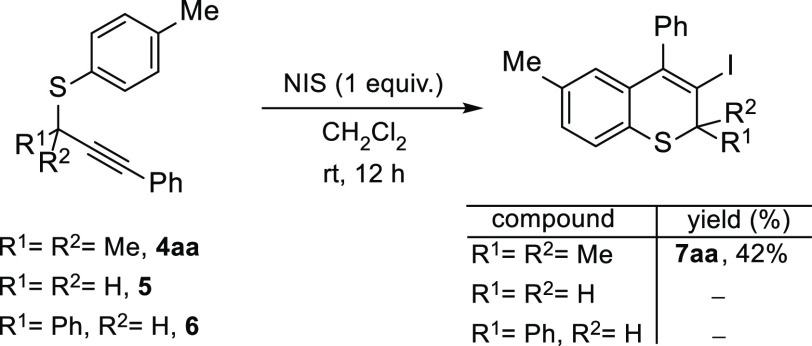
Preliminary Studies of Intramolecular Iodoarylation of *S*-Aryl Propargyl Thioethers

To test our hypothesis, we initially focused on electrophilic iodonium reagents based on our previous experience in iodocarbocyclizations.^23^ Dimethyl-substituted propargylic thioether **4aa**, the analogous propargyl unsubstituted compound **5**, and secondary propargyl sulfide **6** were evaluated with *N*-iodosuccinimide (NIS) (Scheme 4). Whereas the reaction of **4aa** with NIS gave rise to the desired 3-iodothiochromene **7aa**, primary and secondary propargyl thioethers **5** and **6** afforded only disulfide and multiple byproducts, possibly derived from sulfur–halogen substitution;^11^ the thiochromenes were not observed. This differentiated reactivity of **4aa** suggests a crucial *gem*-dimethyl effect.

Once we demonstrated the feasibility of the process, we continued with the optimization (Table 1). An increase in reaction time and a slight excess of NIS provide 3-iodothiochromene **7aa** in higher yields (entries 1 and 2). Lewis or Brønsted acid additives (entries 3–5) do not positively impact the reaction. Other possible iodonium sources, such as molecular iodine in the absence (entry 6) or presence of carbonates (entries 7 and 8), were less efficient. When NBS and NCS replaced NIS, no desired thiochromenes were obtained. Instead, the corresponding disulfide and multiple byproducts possibly derived from sulfur–halogen substitution^11^ and elimination reactions were obtained.

**Table 1:**
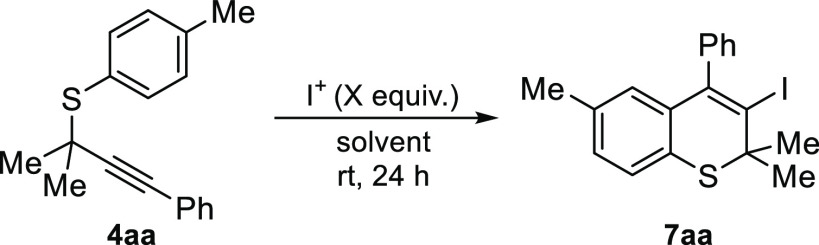
Optimization of the Reaction Conditions for the Iodocarbocyclization Reaction of **4aa**^a^

entry	I^+^ source	equiv	solvent	additive	yield (%)^b^
1	NIS	1.1	CH_2_Cl_2_	–	51
2	NIS	1.3	CH_2_Cl_2_	–	76 (74)^c^
3^d^	NIS	1.1	CH_2_Cl_2_	BF_3_·Et_2_O	56
4^e^	NIS	1.3	CH_2_Cl_2_	BF_3_·Et_2_O	60
5^e^	NIS	1.3	CH_2_Cl_2_	AcOH	50
6	I_2_	1.3	CH_2_Cl_2_	–	–
7^e^	I_2_	1.3	CH_2_Cl_2_	Na_2_CO_3_	32
8^e^	I_2_	1.3	CH_2_Cl_2_	K_2_CO_3_	35

a Reaction conditions: **4aa** (0.1 mmol) and NIS (0.13 mmol) in CH_2_Cl_2_ (1 mL).

b Determined by ^1^H NMR using CH_2_Br_2_ as the internal standard.

c Yield after column chromatography in parentheses.

d With 0.11 mmol of additive.

e With 0.13 mmol of additive.

**Scheme 5:**
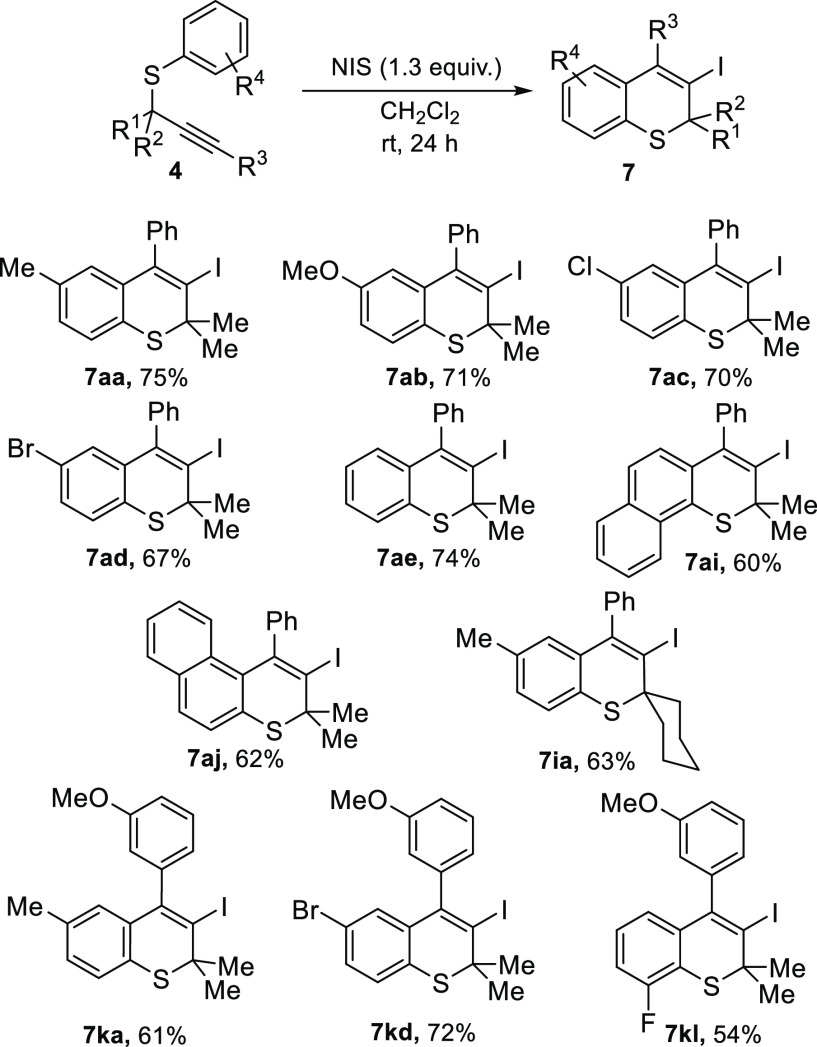
Synthesis of Iodothiochromene Derivatives **7** from Propargyl Thioethers **4**

Next, a selection of *S*-aryl propargyl thioethers **4** was subjected to the optimized reaction conditions (Table 1, entry 2), affording various 3-iodothiochromenes **7** (Scheme 5). Electron-donating and neutral groups (**7aa**, **7ab**, and **7ae**) at the *para* position of the thioaryl moiety (R^4^) gave rise to the corresponding 3-iodothiochromenes in high yields. Moderate electron-withdrawing groups such as halogens at the *para* (**7ac–ad** and **7kd**) and *ortho* (**7kl**) position are also well-tolerated. Interestingly, iodoarylation of **4aj** takes place selectively in the most activated position of the 2-thionaphthol moiety. Additionally, thioether **4ai** also provided access to a tricyclic scaffold (**7ai**). Modification over the propargylic position was also accomplished, affording spirocyclic compound **7ia**. Alkynes bearing methoxy-functionalized arenes in R^3^ also delivered the desired thiochromenes in variable yields (**7ka**, **7kd**, and **7kl**).

Once we studied the electrophilic iodoarylation of thioethers **4**, we envisioned that metal π-acid catalysts might behave like iodonium reagents allowing the alkyne hydroarylation reaction (Table 2).^21^ Considering the extraordinary ability of gold(I) complexes to activate alkynes,^2b,24^ we decided to start our study by employing these types of catalysts. Initial assays with IPrAuNTf_2_ complexes were unfruitful (entry 1). Gratifyingly, cationic gold complexes generated *in situ* using silver triflate as a halide scavenger could promote the cyclization generating the desired thiochromene **8aa** (entry 2). However, control experiments with the silver salt revealed that AgOTf is indeed the catalyst (entries 3–6).^25^ Other metal triflates also afforded the desired thiochromene **8aa**, although in lower proportions (entries 7 and 8). Next, we studied the nature of the silver catalyst (entries 9–11). Silver salts bearing a less coordinating counteranion gave rise to **8aa** in poor yields (entries 9 and 10), possibly due to their lower stability, which results in the formation of a silver mirror. Finally, Brønsted acids were also checked as suitable catalysts. Triflic acid proved to be effective, although lower yields were achieved, presumably due to the degradation of **4aa** into a diverse variety of unidentified byproducts (entries 12–14).

**Table 2:**
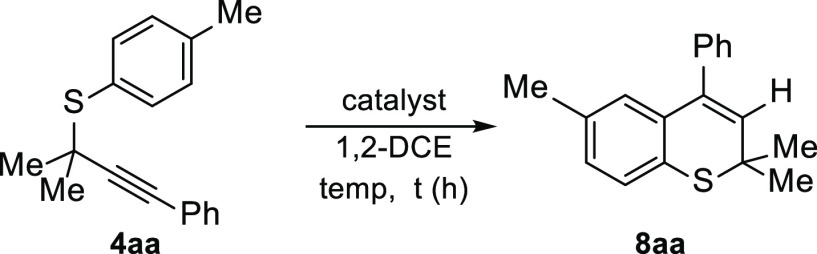
Optimization of the Reaction Conditions for the Hydroarylation Reaction of **4aa**^a^

entry	catalyst	mol %	temp	*t* (h)	yield (%)^b^
1	IPrAuNTf_2_	5	reflux	24	–
2	IPrAuCl/AgOTf	5	reflux	5	80
3	AgOTf	5	reflux	1	79
4	AgOTf	5	reflux	5	86 (83)^c^
5	AgOTf	5	rt	24	–
6	AgOTf	5	60	24	65
7	Bi(OTf)_3_	5	reflux	5	45
8	Sc(OTf)_3_	5	reflux	5	28
9^d^	AgSbF_6_	5	reflux	5	8
10^d^	AgBF_4_	5	reflux	5	5<
11^d^	AgNTf_2_	5	reflux	5	21
12	TfOH	5	reflux	5	40
13	TfOH	1	reflux	5	36
14^d^	TfOH	0.5	reflux	24	19

a Reaction conditions: **4aa** (0.1 mmol) in 1,2-dichloroethane (1 mL).

b Determined by ^1^H NMR using CH_2_Br_2_ as an internal standard.

c Yield after column chromatography in parentheses.

d No full conversion of **4aa** was achieved.

**Scheme 6:**
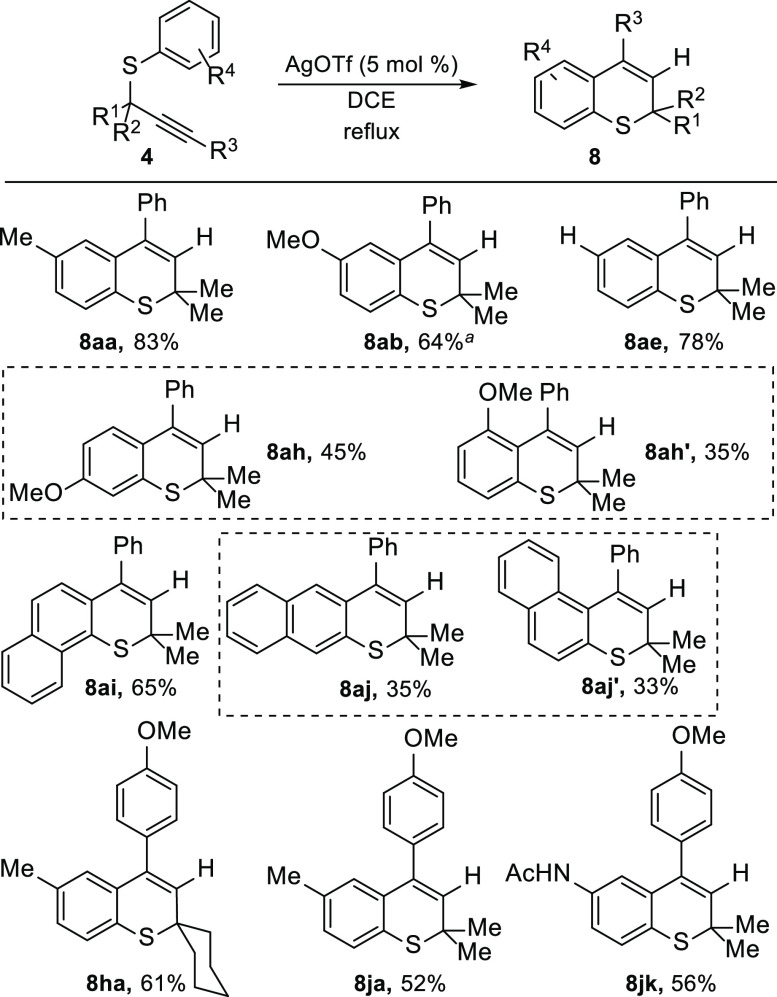
Synthesis of Thiochromene Derivatives **8** through Hydroarylation of Propargyl Thioethers **4** The reaction was performed at 110 °C under microwave irradiation.

With optimized reaction conditions in hand, we decided to evaluate the reactivity of a selection of tertiary propargyl thioethers **4** (Scheme 6). The hydroarylation was efficiently achieved when the thioaryl moiety bears electron-donating or neutral groups (**8aa**, **8ab**, **8ae**, **8ah**, **8ah′**, **8ha**, **8ja**, and **8jk**), whereas substrates bearing moderate electron-withdrawing groups (**4ac** and **4ad**) were not productive, affording inseparable mixtures of various compounds. The deactivation of the arene makes other alternative reaction pathways competitive. As expected, substitution at the *meta* position of the thioaryl fragment afforded a mixture of the two different possible regioisomeric thiochromenes **8ah** and **8ah′** (∼2:1) derived from the 6-*endo* cyclization. Analogous behavior was observed utilizing propargyl thioether **4aj** obtained from 2-thionaphthol. In this case, linear **8aj** and angular **8aj′** thiochromenes were obtained in a 1.2:1 mixture. Propargyl sulfide **4ai** derived from 1-thionaphthol gave rise to the complementary angular 2*H*-thiochromene **8ai**. Activated alkynes bearing methoxy-functionalized arene substituents also underwent hydroarylation (**8ha**, **8ja**, and **8jk**). Similar to iodocarbocyclization, a spiro[cyclohexane-1,2′-thiochromene] (**8ha**) could also be accessed.

### Synthesis of AGN194310

**Scheme 7:**
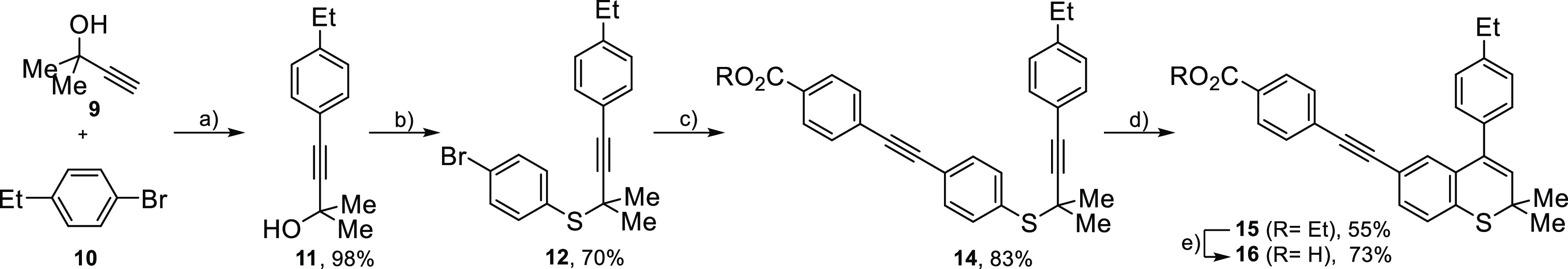
Synthesis of pan-RAR Antagonist AGN194310 **16** Reaction conditions: (a) 1 mol % PdCl_2_(PPh_3_)_2_, 1 mol % CuI, DIPA, 60 °C; (b) 5 mol % PTSA/MeNO_2_, rt; (c) ethyl 4-ethynylbenzoate **13**, 5 mol % PdCl_2_(MeCN)_2_, 10 mol % P*t*Bu_3_, 5 mol % CuI, Et_3_N, reflux; (d) 10 mol % AgOTf, 128 °C, MW (150 W), 25 min/1,2-DCE; (e) NaOH, 2:1 THF/Et_2_O, rt.

To further demonstrate the potential of our methodology, we decided to implement the developed process to synthesize relevant biologically active compounds. Pan-RAR antagonist AGN194310 is a thiochromene derivative that possesses significant activity in RAR signaling^10,26^ and anticancer activity.^27^ This compound has been synthesized in 11 steps with an overall yield of 3.5%, involving a critical 4-thiochromenone intermediate.^10^ In this context, we envisaged that the combination of PTSA-catalyzed thiolation of tertiary propargylic alcohols followed by alkyne hydroarylation could significantly shorten the previously established synthetic route (Scheme 7).

The strategy features cheap and readily available starting materials. At the outset, the Sonogashira cross-coupling between commercially available 2-methyl-3-butyn-2-ol **9** and 1-bromo-4-ethylbenzene **10** led quantitatively to **11**. PTSA-catalyzed thiolation gave rise to the corresponding tertiary *S*-aryl propargyl thioether **12** in high yield. Not surprisingly, the alkyne hydroarylation reaction to access the thiochromene core was inefficient with this substrate, likely due to the incompatibility between the bromo-substituted *S*-aryl propargyl thioethers and the silver catalyst used in the process. Another Sonogashira coupling occurred before the final cyclization to circumvent this issue, furnishing an alternative propargyl sulfide **14** bearing two differentiated alkynes. After careful tuning of the reaction conditions, using microwave irradiation and AgOTf (10 mol %) as a catalyst, selective activation of the propargyl alkyne occurs to generate the thiochromene core. Finally, using a well-established methodology for the ester’s deprotection allowed us to obtain AGN194310. This synthetic sequence affords the pan-RAR antagonist in only five steps in a 22% overall yield.

## Conclusions

In summary, herein, we have described the first electrophilic iodo- and hydroarylation of *S*-aryl propargyl thioethers to synthesize densely substituted 2*H*-thiochromenes by using NIS and a silver salt as the electrophilic reagent and catalyst, respectively. The applicability of this method was demonstrated by the synthesis of the highly selective retinoic acid receptor antagonist AGN194310. Upon application of this strategy, the synthesis was considerably shortened from 11 to 5 steps. The *gem*-disubstituent effect plays a crucial role in favoring 6-*endo*-*dig* iodo and hydroarylation reactions. The absence of substituents at the propargylic position makes other alternative reaction pathways competitive. Additionally, we have developed a reliable, easily scalable methodology for synthesizing the required *S*-aryl propargyl thioethers bearing a quaternary center at the propargylic position by employing PTSA as a cheap and readily available catalyst.

## Experimental Section

### General Methods

All reactions involving air-sensitive compounds were carried out under a N_2_ atmosphere (99.99%). All glassware was oven-dried (120 °C), evacuated, and purged with nitrogen. Temperatures were reported as bath temperatures. All common reagents and solvents were obtained from commercial suppliers and used without further purification. Non-commercially available propargyl alcohols were prepared following previously described procedures: addition of alkynyl organometallic to a carbonyl^16c,28^ and/or Sonogashira cross-coupling reaction.^29^ Solvents were dried following standard methods. Hexane and ethyl acetate were purchased as extra pure grade reagents and used as received. TLC was performed on alumina-backed plates coated with silica gel 60 with the F254 indicator; the chromatograms were visualized by UV light (254 nm) and/or by staining with a Ce/Mo reagent, anisaldehyde, or phosphomolybdic acid solution and subsequent heating. *R_f_* values refer to silica gel. Flash column chromatography was carried out on silica gel 60, 230–400 mesh. ^1^H and ^13^C NMR spectra were recorded on a Varian Mercury-Plus (300 MHz for ^1^H, 75.4 MHz for ^13^C) or Bruker Avance (300 MHz for ^1^H, 75.4 MHz for ^13^C, 282 MHz for ^19^F) spectrometer at room temperature. NMR splitting pattern abbreviations are as follows: s, singlet; br s, broad singlet; d, doublet; dd, doublet of doublets; dt, doublet of triplets; ddd, doublet of doublet of doublets; t, triplet; td, triplet of doublets; q, quartet; quint, quintet; sext, sextet; m, multiplet. Chemical shifts are reported in parts per million using the residual solvent peak as a reference (CDCl_3_, ^1^H δ 7.26 and ^13^C δ 77.16; DMSO-*d*_6_, ^1^H δ 2.50 and ^13^C δ 39.50; acetone-*d*_6_, ^1^H δ 2.05 and ^13^C δ 206.26, 29.84), and the multiplicities of ^13^C signals were determined by DEPT experiments.

GC-MS spectra were recorded on an Agilent 6890*N*/5973 Network GC System, equipped with an HP-5MS column or a Thermo 1300GC instrument equipped with an MS 7000ISQ STDNOVPI MS detector, using Chromeleon software. Low-resolution electron impact mass spectra (EI-LRMS) were obtained at 70 eV on a mass spectrometer, and only the molecular ions and/or base peaks, as well as significant peaks in MS, are given. High-resolution mass spectrometry (HRMS) was carried out on a 6545 Q-TOF (Agilent) mass spectrometer (ESI or APCI as ion source) as specified.

Reactions were carried out in common Pyrex round-bottom flasks, and those performed under microwave irradiation in 10 mL microwave vials crimped on top with 20 mm Sil/PTFE septa. When needed, pH values were determined using pH indicator strips (pH 0–14 Universal indicator paper, Merck MColorpHaspt). Microwave irradiation was realized with a CEM Discover S-Class Reactor with a single-mode microwave cavity producing continuous irradiation. Temperature measurements were conducted using an IR sensor located below the microwave cavity floor, and reaction times refer to the total hold time at the indicated temperature. The maximum wattage supplied was 220 W.

### General Procedure A for the Synthesis of Propargyl Sulfides **4** from Alcohols **3**

Thiol **2** (1.3 equiv, 0.56 mmol) and *p*-toluenesulfonic acid (4 mg, 0.02 equiv, 5 mol %) were sequentially added to a solution of the corresponding propargyl alcohol **3** (1 equiv, 0.4 mmol) in MeNO_2_ (0.8 mL, 0.5 M). The mixture was allowed to stir at rt for 30 min, until full depletion of the alcohol was determined by TLC (spots were visualized using Ce/Mo reagent and heat as the staining agent). Then, the reaction was quenched by the addition of aqueous NaOH (0.5 M, 10 mL) and CH_2_Cl_2_ (2 mL). The separated aqueous phase was extracted with CH_2_Cl_2_ (3 × 10 mL). The combined organic layers were dried over anhydrous Na_2_SO_4_, filtered, and concentrated under reduced pressure. The residue was purified by column chromatography on silica gel (eluent, hexane/EtOAc mixture), affording the corresponding propargyl sulfides **4**.

#### (2-Methyl-4-phenylbut-3-yn-2-yl) (*p*-Tolyl) Sulfide (**4aa**)

Compound **4aa** was prepared according to general procedure A (reaction time, 30 min). The crude product was purified by flash column chromatography on silica gel (hexane), affording pure **4aa** (81% yield, 87 mg). Pale yellow liquid. *R_f_* = 0.22 (hexane). ^1^H NMR (300 MHz, CDCl_3_, 25 °C): δ 7.69–7.66 (m, 2H), 7.46–7.42 (m, 2H), 7.38–7.33 (m, 3H), 7.25–7.22 (m, 2H), 2.44 (s, 3H), 1.71 (s, 6H). ^13^C{^1^H} NMR (75.4 MHz, CDCl_3_): δ 139.3 (C), 137.1 (2 × CH), 131.6 (2 × CH), 129.4 (2 × CH), 129.0 (C), 128.2 (2 × CH), 128.0 (CH), 123.4 (C), 94.2 (C), 83.2 (C), 42.5 (C), 30.4 (2 × CH_3_), 21.4 (CH_3_). LRMS (EI) *m*/*z* (%): 143 (100), 128 (36), 233 (14), 251 (12), 266 (M^+^, 10). HRMS (ESI+) *m*/*z*: [M + H]^+^ calcd for C_18_H_19_S, 267.1202; found, 267.1205.

#### (4-Methoxyphenyl) (2-Methyl-4-phenylbut-3-yn-2-yl) Sulfide (**4ab**)

Compound **4ab** was prepared according to general procedure A (reaction time, 30 min). The crude product was purified by flash column chromatography on silica gel (50:1 hexane/EtOAc), affording pure **4ab** (58% yield, 65 mg). Pale yellow oil. *R_f_* = 0.12 (50:1 hexane/EtOAc). ^1^H NMR (300 MHz, CDCl_3_): δ 7.68–7.62 (m, 2H), 7.42–7.36 (m, 2H), 7.34–7.30 (m, 3H), 6.95–6.90 (m, 2H), 3.85 (s, 3H), 1.65 (s, 6H). ^13^C{^1^H} NMR (75.4 MHz, CDCl_3_): δ 160.8 (C), 138.8 (2 × CH), 131.6 (2 × CH), 128.3 (2 × CH), 128.0 (CH), 123.4 (C), 114.1 (2 × CH), 94.2 (C), 83.3 (C), 55.4 (CH_3_), 42.7 (C), 30.3 (2 × CH_3_), one C peak missed due to overlapping. LRMS (EI) *m*/*z* (%): 128 (100), 175 (67), 115 (50), 77 (50), 282 (M^+^, 27). HRMS (ESI+) *m*/*z*: [M + H]^+^ calcd for C_18_H_19_OS, 283.1151; found, 283.1156.

#### (4-Chlorophenyl) (2-Methyl-4-phenylbut-3-yn-2-yl) Sulfide (**4ac**)

Compound **4ac** was prepared according to general procedure A (reaction time, 2 h). The crude product was purified by flash column chromatography on silica gel (hexane), affording pure **4ac** (81% yield, 90 mg). Pale yellow solid. Mp: 49–51 °C. *R_f_* = 0.24 (hexane). ^1^H NMR (300 MHz, CDCl_3_): δ 7.72–7.71 (m, 1H), 7.69–7.68 (m, 1H), 7.44–7.39 (m, 3H), 7.37–7.34 (m, 4H), 1.71 (s, 6H). ^13^C{^1^H} NMR (75.4 MHz, CDCl_3_): δ 138.2 (2 × CH), 135.6 (C), 131.5 (2 × CH), 131.1 (C), 128.7 (2 × CH), 128.3 (2 × CH), 128.1 (CH), 123.1 (C), 93.7 (C), 83.6 (C), 42.9 (C), 30.4 (2 × CH_3_). LRMS (EI) *m*/*z* (%): 143 (100), 128 (32), 286 (M^+^, 5). HRMS (ESI+) *m*/*z*: [M + H]^+^ calcd for C_17_H_16_ClS, 287.0656; found, 287.0651.

#### (4-Bromophenyl) (2-Methyl-4-phenylbut-3-yn-2-yl) Sulfide (**4ad**)

Compound **4ad** was prepared according to general procedure A (reaction time, 2 h). The crude product was purified by flash column chromatography on silica gel (hexane), affording pure **4ad** (79% yield, 102 mg). White oil. *R_f_* = 0.5 (hexane). ^1^H NMR (300 MHz, CDCl_3_): δ 7.63–7.58 (m, 2H), 7.55–7.50 (m, 2H), 7.41–7.33 (m, 5H), 1.68 (s, 6H). ^13^C{^1^H} NMR (75.4 MHz, CDCl_3_): δ 138.4 (2 × CH).131.8 (C), 131.8 (2 × CH), 131.6 (2 × CH), 128.4 (2 × CH), 128.2 (CH), 124.0 (C), 123.1 (C), 93.7 (C), 83.7 (C), 42.9 (C), 30.5 (2 × CH_3_). LRMS (EI) *m*/*z* (%): 143 (100), 128 (38), 108 (15), 330 (M^+^, 5). HRMS (ESI+) *m*/*z*: [M + H]^+^ calcd for C_17_H_16_BrS, 331.0151; found, 331.0149.

#### (2-Methyl-4-phenylbut-3-yn-2-yl) (Phenyl) Sulfide (**4ae**)

Compound **4ae** was prepared according to general procedure A (reaction time, 30 min). The crude product was purified by flash column chromatography on silica gel (hexane), affording pure **4ae** (70% yield, 71 mg). Pale yellow liquid. *R_f_* = 0.23 (hexane). ^1^H NMR (300 MHz, CDCl_3_): δ 7.71–7.75 (m, 2H), 7.35–7.43 (m, 6H), 7.28–7.33 (m, 2H), 1.67 (s, 6H). ^13^C{^1^H} NMR (75.4 MHz, CDCl_3_, 25 °C): δ 137.1 (2 × CH), 132.6 (C), 131.6 (2 × CH), 129.2 (CH), 128.6 (2 × CH), 128.3 (2 × CH), 128.1 (CH), 123.4 (C), 94.1 (C), 83.4 (C), 42.7 (C), 30.6 (2 × CH_3_). NMR data are in full agreement with previously described data.^19^ LRMS (EI) *m*/*z* (%): 143 (100), 128 (64), 115 (31), 65 (28), 252 (M^+^, 3).

#### (2-Bromophenyl) (2-Methyl-4-phenylbut-3-yn-2-yl) Sulfide (**4af**)

Compound **4af** was prepared according to general procedure A (reaction time, 2 h). The crude product was purified by flash column chromatography on silica gel (hexane), affording pure **4af** (82% yield, 110 mg). Yellow liquid. *R_f_* = 0.2 (hexane). ^1^H NMR (300 MHz, CDCl_3_): δ 8.02 (dd, *J* = 7.72, 1.69 Hz, 1H), 7.72 (dd, *J* = 7.93, 1.35 Hz, 1H), 7.44–7.40 (m, 2H), 7.37–7.32 (m, 4H), 7.23 (td, *J* = 7.86, 1.70 Hz, 1H), 1.77 (s, 6H). ^13^C{^1^H} NMR (75.4 MHz, CDCl_3_): δ 137.9 (CH), 134.6 (C), 133.4 (CH), 131.7 (2 × CH), 131.0 (C), 130.1 (CH), 128.4 (2 × CH), δ 128.3 (CH), 127.5 (CH), 123.3 (C), 93.6 (C), 83.9 (C), 44.3 (C), 30.8 (2 × CH_3_). LRMS (EI) *m*/*z* (%): 143 (100), 128 (30), 251 (13), 108 (12), 330 (M^+^, 10). HRMS (ESI+) *m*/*z*: [M + H]^+^ calcd for C_17_H_16_BrS, 331.0151; found, 331.0149.

#### (2-Chlorophenyl) (2-Methyl-4-phenylbut-3-yn-2-yl) Sulfide (**4ag**)

Compound **4ag** was prepared according to general procedure A (reaction time, 2 h). The crude product was purified by flash column chromatography on silica gel (hexane), affording pure **4ag** (75% yield, 86 mg). Yellow oil. *R_f_* = 0.23 (hexane). ^1^H NMR (300 MHz, CDCl_3_): δ 8.03–8.01 (m, 1H), 7.57–7.54 (m, 1H), 7.46–7.43 (m, 2H), 7.37–7.34 (m, 5H), 1.79 (s, 6H). ^13^C{^1^H} NMR (75.4 MHz, CDCl_3_): δ 139.8 (C),138.4 (CH), 132.1 (C), 131.5 (2 × CH), 130.1 (CH), 129.9 (CH), 128.2 (2 × CH), 128.1 (CH), 126.7 (CH), 123.1 (C), 93.5 (C), 83.7 (C), 44.1 (C), 30.7 (2 × CH_3_). LRMS (EI) *m*/*z* (%): 143 (100), 128 (40), 77 (13), 127 (12), 286 (M^+^, 3). HRMS (ESI+) *m*/*z*: [M + H]^+^ calcd for C_17_H_16_ClS, 287.0656; found, 287.0652.

#### (3-Methoxyphenyl) (2-Methyl-4-phenylbut-3-yn-2-yl) Sulfide (**4ah**)

Compound **4ah** was prepared according to general procedure A (reaction time, 2 h). The crude product was purified by flash column chromatography on silica gel (20:1 hexane/EtOAc), affording pure **4ah** (68% yield, 73 mg). Yellow oil. *R_f_* = 0.35 (20:1 hexane/EtOAc). ^1^H NMR (300 MHz, CDCl_3_): δ 7.48–7.45 (m, 2H), 7.42–7.41 (m, 1H), 7.39–7.34 (m, 5H), 7.04–7.00 (m, 1H), 3.77 (s, 3H), 1.76 (s, 6H). ^13^C{^1^H} NMR (75.4 MHz, CDCl_3_): δ 159.3 (C), 133.6 (C), 131.5 (2 × CH), 129.2 (CH), 128.9 (CH), 128.2 (2 × CH), 128.0 (CH), 123.2 (C), 121.5 (CH), 115.3 (CH), 94.1 (C), 83.3 (C), 55.1 (CH_3_), 42.5 (C), 30.5 (2 × CH_3_). 139 LRMS (EI) *m*/*z* (%): 143 (100), 138 (33), 267 (22), 282 (M+, 15). HRMS (ESI+) *m*/*z*: [M + H]^+^ calcd for C_18_H_19_OS, 283.1151; found, 283.1155.

#### (2-Methyl-4-phenylbut-3-yn-2-yl) (Naphthalen-1-yl) Sulfide (**4ai**)

Compound **4ai** was prepared according to general procedure A (reaction time, 2 h). The crude product was purified by flash column chromatography on silica gel (hexane), affording pure **4ai** (83% yield, 102 mg). Orange oil. *R_f_* = 0.22 (hexane). ^1^H NMR (300 MHz, CDCl_3_): δ 8.96–8.93 (m, 1H), 8.18–8.10 (m, 1H), 8.02–7.94 (m, 2H), 7.65–7.54 (m, 3H), 7.34–7.30 (m, 3H), 7.26–7.23 (m, 2H), 1.80 (s, 6H). ^13^C{^1^H} NMR (75.4 MHz, CDCl_3_): δ 137.1 (CH), 136.4 (C), 134.0 (C), 131.3 (CH), 130.2 (CH), 130.1 (C), 128.1 (CH), 127.9 (CH), 127.7 (CH), 127.1 (CH), 126.4 (CH), 125.9 (CH), 125.1 (CH), 123.0 (C), 94.0 (C), 83.5 (C), 43.7 (C), 30.8 (2 × CH_3_). LRMS (EI) *m*/*z* (%): 143 (100), 115 (50), 302 (M^+^, 35). HRMS (ESI+) *m*/*z*: [M + H]^+^ calcd for C_21_H_19_S, 303.1202; found, 303.1201.

#### (2-Methyl-4-phenylbut-3-yn-2-yl) (Naphthalen-2-yl) Sulfide (**4aj**)

Compound **4aj** was prepared according to general procedure A (reaction time, 2 h). The crude product was purified by flash column chromatography on silica gel (hexane), affording pure **4aj** (71% yield, 88 mg). Colorless solid. Mp: 62–64 °C. *R_f_* = 0.16 (hexane). ^1^H NMR (300 MHz, CDCl_3_): δ 8.40 (s, 1H), 7.97–7.92 (m, 4H), 7.63–7.60 (m, 2H), 7.52–7.48 (m, 2H), 7.41–7.38 (m, 3H), 1.85 (s, 6H). ^13^C{^1^H} NMR (75.4 MHz, CDCl_3_): δ 136.8 (CH), 133.6 (CH), 133.4 (C), 133.4 (C), 131.5 (2 × CH), 131.0 (C), 128.2 (2 × CH), 128.0 (CH), 128.0 (CH), 127.9 (CH), 127.7 (CH), 126.8 (CH), 126.3 (CH), 123.2 (C), 94.1 (C), 83.6 (C), 42.9 (C), 30.6 (2 × CH_3_). LRMS (EI) *m*/*z* (%): 143 (100), 115 (43), 128 (40), 302 (M^+^, 37). HRMS (ESI+) *m*/*z*: [M + H]^+^ calcd for C_21_H_19_S, 303.1202; found, 303.1203.

#### (2,4-Diphenylbut-3-yn-2-yl) (*p*-Tolyl) Sulfide (**4ba**)

Compound **4ba** was prepared according to general procedure A (reaction time, 30 min). The crude product was purified by flash column chromatography on silica gel (hexane), affording pure **4ba** (90% yield, 120 mg). Colorless oil. *R_f_* = 0.17 (hexane). ^1^H NMR (300 MHz, CDCl_3_, 25 °C): δ 7.68–7.64 (m, 2H), 7.44–7.42 (m, 2H), 7.35–7.29 (m, 8H), 7.08–7.05 (m, 2H), 2.34 (s, 3H), 2.02 (s, 3H). ^13^C{^1^H} NMR (75.4 MHz, CDCl_3_): δ 142.6 (C), 139.4 (C), 136.9 (2 × CH), 131.5 (2 × CH), 129. (C), 129.0 (2 × CH), 128.2 (2 × CH), 128.1 (CH), 128.0 (2 × CH), 127.3 (CH), 126.8 (2 × CH), 123.2 (C), 91.9 (C), 86.8 (C), 50.2 (CH), 29.9 (CH_3_), 21.3 (CH_3_). LRMS (EI) *m*/*z* (%): 205 (100), 127 (18), 328 (M^+^, 14). HRMS (ESI+) *m*/*z*: [M + H]^+^ calcd for C_23_H_21_S, 329.1358; found, 329.1361.

#### (1-Methoxy-2,4-diphenylbut-3-yn-2-yl) (*p*-Tolyl) Sulfide (**4ca**)

Compound **4ac** was prepared according to general procedure A (reaction time, 30 min). The crude product was purified by flash column chromatography on silica gel (50:1 hexane/EtOAc), affording pure **4ac** (65% yield, 92 mg). Orange oil. *R_f_* = 0.23 (50:1 hexane/EtOAc). ^1^H NMR (300 MHz, CDCl_3_): δ 7.77–7.74 (m, 2H), 7.47–7.44 (m, 2H), 7.41–7.40 (m, 1H), 7.39–7.31 (m, 7H), 7.09 (d, *J* = 7.8 Hz, 2H), 4.09 (d, *J* = 9.7 Hz, 1H), 3.91 (d, *J* = 9.7 Hz, 1H), 3.45 (s, 3H), 2.36 (s, 3H). ^13^C{^1^H} NMR (75.4 MHz, CDCl_3_): δ 139.6 (C), 139.4 (C), 137.3 (2 × CH), 131.8 (2 × CH), 129.3 (2 × CH), 128.4 (2 × CH), 128.3 (2 × CH), 128.3 (2 × CH), 128.1 (C), 127.8 (2 × CH), 123.2 (C), 89.4 (C), 88.5 (C), 79.0 (CH_2_), 59.9 (CH_3_), 55.1 (C), 21.4 (CH_3_). LRMS (EI) *m*/*z* (%): 207 (100), 221 (49), 299 (38), 281 (33), 358 (M^+^, 5). HRMS (ESI+) *m*/*z*: [M + H]^+^ calcd for C_24_H_23_OS, 359.1464; found, 359.1466.

#### (3-Phenyl-1,1-di-*p*-tolylprop-2-yn-1-yl) (*p*-Tolyl) Sulfide (**4da**)

Compound **4da** was prepared according to general procedure A (reaction time, 30 min). The crude product was purified by flash column chromatography on silica gel (hexane), affording pure **4da** (53% yield, 87 mg). Yellow oil. *R_f_* = 0.18 (hexane). ^1^H NMR (300 MHz, CDCl_3_): δ 7.62 (d, *J* = 8.3 Hz, 4H), 7.42–7.38 (m, 2H), 7.35–7.32 (m, 3H), 7.29–7.27 (m, 2H), 7.15 (d, *J* = 8.1 Hz, 4H), 7.02 (d, *J* = 7.9 Hz, 2H), 2.37 (s, 6H), 2.33 (s, 3H). ^13^C{^1^H} NMR (75.4 MHz, CDCl_3_): δ 139.7 (2 × C), 139.0 (C), 137.1 (CH), 136.3 (2 × CH), 131.7 (2 × CH), 129.9 (2 × C),129.1 (2 × CH), 128.9 (4 × CH), 128.7 (C), 128.3 (4 × CH), 128.2 (2 × CH), 123.4 (C), 91.6 (C), 88.8 (C), 59.2 (C), 21.4 (CH_3_), 21.2 (2 × CH_3_). HRMS (ESI+) *m*/*z*: [M + H]^+^ calcd for C_30_H_27_S, 419.1828; found, 419.1833.

#### (1,1-Dicyclopropyl-3-phenylprop-2-yn-1-yl) (*p*-Tolyl) Sulfide (**4ea**)

Compound **4ea** was prepared according to general procedure A (reaction time, 2 h). The crude product was purified by flash column chromatography on silica gel (hexane), affording pure **4ea** (64% yield, 80 mg). Yellow oil. *R_f_* = 0.12 (hexane). ^1^H NMR (300 MHz, CDCl_3_): δ 7.65 (d, *J* = 7.02 Hz, 2H), 7.36–7.28 (m, 5H), 7.15 (d, *J* = 7.9 Hz, 2H), 2.39 (s, 3H), 1.32–1.23 (m, 2H), 0.62–0.40 (m, 8H). ^13^C{^1^H} NMR (75.4 MHz, CDCl_3_): δ 139.2 (C), 137.8 (2 × CH), 131.6 (2 × CH), 129.0 (2 × CH), 128.6 (C), 128.3 (2 × CH), 128.2 (CH), 123.0 (C), 86.8 (C), 85.4 (C), 56.2 (C), 21.5 (CH_3_), 20.3 (2 × CH), 2.6 (2 × CH_2_), 2.6 (2 × CH_2_). LRMS (EI) *m*/*z* (%): 195 (100), 136 (54), 318 (M^+^, 19). HRMS (APCI+) *m*/*z*: [M + H]^+^ calcd for C_22_H_23_S, 319.1515; found, 319.1519.

#### (2-Cyclopropyl-4-phenylbut-3-yn-2-yl) (*p*-Tolyl) Sulfide (**4fa**)

Compound **4fa** was prepared according to general procedure A (reaction time, 2 h). The crude product was purified by flash column chromatography on silica gel (hexane), affording pure **4fa** (66% yield, 71 mg). Pale yellow oil. *R_f_* = 0.19 (hexane). ^1^H NMR (300 MHz, CDCl_3_): δ 7.72 (d, *J* = 8.10 Hz, 2H), 7.44–7.42 (m, 2H), 7.37–7.35 (m, 3H), 7.24 (d, *J* = 7.9 Hz, 2H), 2.45 (s, 3H), 1.74 (s, 3H), 1.30–1.23 (m, 1H), 0.71–0.54 (m, 4H). ^13^C{^1^H} NMR (75.4 MHz, CDCl_3_): δ 139.2 (C), 137.4 (2 × CH), 131.5 (2 × CH), 129.1 (2 × CH), 128.7 (C), 128.2 (2 × CH), 128.0 (CH), 123.2 (C), 89.4 (C), 85.2 (C), 49.3 (C), 29.3 (CH_3_), 21.2 (CH), 21.0 (CH_3_), 3.6 (CH_2_), 2.5 (CH_2_). LRMS (EI) *m*/*z* (%): 154 (100), 169 (98), 141 (78), 292 (M^+^, 8). HRMS (ESI+) *m*/*z*: [M + H]^+^ calcd for C_20_H_21_S, 293.1358; found, 293.1361.

#### [2-Cyclopropyl-4-(4-methoxyphenyl)but-3-yn-2-yl] (*p*-Tolyl) Sulfide (**4ga**)

Compound **4ga** was prepared according to general procedure A (reaction time, 2 h). The crude product was purified by flash column chromatography on silica gel (20:1 hexane/EtOAc), affording pure **4ga** (65% yield, 84 mg). Colorless oil. *R_f_* = 0.37 (20:1 hexane/EtOAc). ^1^H NMR (300 MHz, CDCl_3_): δ 7.65–7.61 (m, 2H), 7.33–7.28 (m, 2H), 7.17–7.16 (m, 2H), 6.88–6.82 (m, 2H), 3.83 (s, 3H), 2.4 (s, 3H), 1.66 (s, 3H), 1.24–1.15 (m, 1H), 0.63–0.46 (m, 4H). ^13^C{^1^H} NMR (75.4 MHz, CDCl_3_): δ 159.5 (C), 139.2 (C), 137.4 (2 × CH), 133 (2 × CH), 129.2 (2 × CH), 128.6 (C), 115.4 (C), 113.9 (2 × CH), 87.9 (C), 85.1 (C), 55.4 (CH_3_), 49.6 (C), 29.4 (CH_3_), 21.4 (CH_3_), 21.1 (CH), 3.6 (CH_2_), 2.5 (CH_2_). LRMS (EI) *m*/*z* (%): 91 (100), 231 (76), 199 (64), 115 (61), 322 (M^+^, 20). HRMS (ESI+) *m*/*z*: [M + H]^+^ calcd for C_21_H_23_OS, 323.1464; found, 323.1466.

#### {1-[(4-Methoxyphenyl)ethynyl]cyclohexyl} (*p*-Tolyl) Sulfide (**4ha**)

Compound **4ha** was prepared according to general procedure A (reaction time, 2 h). The crude product was purified by flash column chromatography on silica gel (10:1 hexane/EtOAc), affording pure **4ha** (84% yield, 113 mg). Pale yellow liquid. *R_f_* = 0.39 (10:1 hexane/EtOAc). ^1^H NMR (300 MHz, CDCl_3_): δ 7.66–7.62 (m, 2H), 7.39–7.34 (m, 2H), 7.21–7.19 (m, 2H), 6.90–6.85 (m, 2H), 3.84 (s, 3H), 2.41 (s, 3H), 2.09–2.04 (m, 2H), 1.78–1.65 (m, 7H), 1.36–1.31 (m, 1H). ^13^C{^1^H} NMR (75.4 MHz, CDCl_3_): δ 159.4 (C), 139.2 (C), 137.3 (2 × CH), 133.0 (2 × CH), 129.3 (2 × CH), 128.2 (C), 115.8 (C), 113.9 (2 × CH), 91.0 (C), 85.3 (C), 55.4 (CH_3_), 48.3 (C), 38.8 (2 × CH_2_), 25.7 (CH_2_), 23.8 (2 × CH_2_), 21.4 (CH_3_). LRMS (EI) *m*/*z* (%): 91 (100), 213 (91), 336 (M^+^, 89). HRMS (ESI+) *m*/*z*: [M + H]^+^ calcd for C_22_H_25_OS, 337.1621; found, 337.1626.

#### [1-(Phenylethynyl)cyclohexyl] (*p*-Tolyl) Sulfide (**4ia**)

Compound **4ia** was prepared according to general procedure A (reaction time, 2 h). The crude product was purified by flash column chromatography on silica gel (hexane), affording pure **4ia** (87% yield, 106 mg). Colorless oil. *R_f_* = 0.59 (hexane). ^1^H NMR (300 MHz, CDCl_3_): δ 7.66–7.63 (m, 2H), 7.44–7.41 (m, 2H), 7.35–7.33 (m, 3H), 7.22–7.19 (m, 2H), 2.42 (s, 3H), 2.13–2.04 (m, 2H), 1.81–1.66 (m, 7H), 1.37–1.33 (m, 1H). ^13^C{^1^H} NMR (75.4 MHz, CDCl_3_): δ 139.2 (C), 137.3 (2 × CH), 131.6 (2 × CH), 129.3 (2 × CH), 128.3 (2 × CH), 128 (C), 127.9 (CH), 123.7 (C), 92.6 (C), 85.5 (C), 48.1 (C), 38.8 (2 × CH_2_), 25.6 (CH_2_), 23.7 (2 × CH_2_), 21.4 (CH_3_). LRMS (EI) *m*/*z* (%): 115 (100), 79 (92), 155 (88), 141 (81), 306 (M^+^, 71). HRMS (ESI+) *m*/*z*: [M + H]^+^ calcd for C_21_H_23_S, 307.1515; found, 307.1519.

#### [4-(4-Methoxyphenyl)-2-methylbut-3-yn-2-yl] (*p*-Tolyl) Sulfide (**4ja**)

Compound **4ja** was prepared according to general procedure A (reaction time, 1 h). The crude product was purified by flash column chromatography on silica gel (20:1 hexane/EtOAc), affording pure **4ja** (75% yield, 89 mg). Yellow oil. *R_f_* = 0.38 (20:1 hexane/EtOAc). ^1^H NMR (300 MHz, CDCl_3_): δ 7.61 (d, *J* = 8.00 Hz, 2H), 7.34–7.31 (m, 2H), 7.21–7.18 (m, 2H), 6.85 (d, *J* = 8.8 Hz, 2H), 3.83 (s, 3H), 2.41 (s, 3H), 1.65 (s, 6H). ^13^C{^1^H} NMR (75.4 MHz, CDCl_3_): δ 159.4 (C), 139.3 (C), 137.1 (2 × CH), 133 (2 × CH), 129.4 (2 × CH), 129.2 (C), 115.6 (C), 113.9 (2 × CH), 92.7 (C), 83.1 (C), 55.4 (CH_3_), 42.7 (C), 30.6 (2 × CH_3_), 21.4 (CH_3_). LRMS (EI) *m*/*z* (%): 173 (100), 115 (23), 128 (22), 296 (M^+^, 7). HRMS (ESI+) *m*/*z*: [M + H]^+^ calcd for C_19_H_21_OS, 297.1308; found, 297.1311.

#### (4-Chlorophenyl) [4-(4-Methoxyphenyl)-2-methylbut-3-yn-2-yl] Sulfide (**4jc**)

Compound **4jc** was prepared according to general procedure A (reaction time, 2 h). The crude product was purified by flash column chromatography on silica gel (20:1 hexane/EtOAc), affording pure **4jc** (82% yield, 103 mg). Orange oil. *R_f_* = 0.38 (20:1 hexane/EtOAc). ^1^H NMR (300 MHz, CDCl_3_): δ 7.66–7.61 (m, 2H), 7.36–7.28 (m, 4H), 6.88–6.83 (m, 2H), 3.83 (s, 3H), 1.64 (s, 6H). ^13^C{^1^H} NMR (75.4 MHz, CDCl_3_): δ 159.6 (C), 138.2 (2 × CH), 135.7 (C), 133 (2 × CH), 131.3 (C), 128.8 (2 × CH), 115.3 (C), 114.0 (2 × CH), 92.2 (C), 83.6 (C), 55.4 (CH_3_), 43.1 (C), 30.6 (2 × CH_3_). LRMS (EI) *m*/*z* (%): 173 (100), 128 (20), 115 (19), 316 (M^+^, 3). HRMS (ESI+) *m*/*z*: [M + H]^+^ calcd for C_18_H_18_ClOS, 317.0761; found, 317.0764.

#### *N*-(4-{[4-(4-Methoxyphenyl)-2-methylbut-3-yn-2-yl]thio}phenyl)acetamide (**4jk**)

Compound **4jk** was prepared according to general procedure A (reaction time, 2 h). The crude product was purified by flash column chromatography on silica gel (2:1 hexane/EtOAc), affording pure **4jk** (70% yield, 95 mg). Pale yellow oil. *R_f_* = 0.12 (2:1 hexane/EtOAc). ^1^H NMR (300 MHz, CDCl_3_): δ 8.04 (s, 1H), 7.61–7.57 (m, 2H), 7.54–7.50 (m, 2H), 7.28–7.25 (m, 2H), 6.80–6.77 (m, 2H), 3.76 (s, 3H), 2.13 (s, 3H), 1.58 (s, 6H). ^13^C{^1^H} NMR (75.4 MHz, CDCl_3_): δ 168.9 (C), 159.4 (C), 139.1 (2 × CH),137.9 (C), 132.9 (2 × CH), 127.5 (C), 119.6 (2 × CH), 115.3 (C), 113.9 (2 × CH), 92.5 (C), 83.2 (C), 55.3 (CH_3_), 42.9 (C), 30.4 (2 × CH_3_), 24.6 (CH_3_). LRMS (EI) *m*/*z* (%): 173 (100), 205 (92), 115 (67), 43 (64), 339 (M^+^, 15). HRMS (ESI+) *m*/*z*: [M + H]^+^ calcd for C_20_H_22_NO_2_S, 340.1366; found, 340.1372.

#### [4-(3-Methoxyphenyl)-2-methylbut-3-yn-2-yl] (*p*-Tolyl) Sulfide (**4ka**)

Compound **4ka** was prepared according to general procedure A (reaction time, 1 h). The crude product was purified by flash column chromatography on silica gel (20:1 hexane/EtOAc), affording pure **4ka** (93% yield, 110 mg). Yellow oil. *R_f_* = 0.31 (20:1 hexane/EtOAc). ^1^H NMR (300 MHz, CDCl_3_): δ 7.63 (d, *J* = 7.9 Hz, 2H), 7.26–7.20 (m, 3H), 7.00 (d, *J* = 7.6 Hz, 1H), 6.92–6.87 (m, 2H), 3.83 (s, 3H), 2.41 (s, 3H), 1.66 (s, 6H). ^13^C{^1^H} NMR (75.4 MHz, CDCl_3_): δ 159.3 (C), 139.4 (C), 137.2 (2 × CH), 129.4 (2 × CH), 129.3 (CH), 129.0 (C), 124.4 (C), 124.1 (CH), 116.4 (CH), 114.6 (CH), 94.0 (C), 83.2 (C), 55.3 (CH_3_), 42.5 (C), 30.43 (2 × CH_3_), 21.4 (CH_3_). LRMS (EI) *m*/*z* (%): 173 (100), 281 (23), 115 (18), 296 (M^+^, 16). HRMS (ESI+) *m*/*z*: [M + H]^+^ calcd for C_19_H_21_OS, 297.1308; found, 297.1311.

#### (4-Bromophenyl) [4-(3-Methoxyphenyl)-2-methylbut-3-yn-2-yl] Sulfide (**4kd**)

Compound **4kd** was prepared according to general procedure A (reaction time, 2 h). The crude product was purified by flash column chromatography on silica gel (20:1 hexane/EtOAc), affording pure **4kd** (70% yield, 102 mg). Pale yellow oil. *R_f_* = 0.36 (20:1 hexane/EtOAc). ^1^H NMR (300 MHz, CDCl_3_): δ 7.62–7.57 (m, 2H), 7.54–7.50 (m, 2H), 7.30–7.21 (m, 1H), 7.00–6.97 (m, 1H), 6.92–6.88 (m, 2H), 3.83 (s, 3H), 1.67 (s, 6H). ^13^C{^1^H} NMR (75.4 MHz, CDCl_3_): δ 159.4 (C), 138.4 (2 × CH), 131.8 (C), 131.7 (2 × CH), 129.4 (CH), 124.1 (CH), 124.0 (C), 116.0 (CH), 114.8 (CH), 93.4 (C), 83.7 (C), 55.3 (CH_3_), 42.9 (C), 30.5 (2 × CH_3_), one C peak missing due to overlapping. LRMS (EI) *m*/*z* (%): 173 (100), 115 (18), 128 (15), 368 (M^+^, 8). HRMS (ESI+) *m*/*z*: [M + H]^+^ calcd for C_18_H_18_BrOS, 361.0256; found, 361.0254.

#### (2-Fluorophenyl) [4-(3-Methoxyphenyl)-2-methylbut-3-yn-2-yl] Sulfide (**4kl**)

Compound **4kl** was prepared according to general procedure A (reaction time, 2 h). The crude product was purified by flash column chromatography on silica gel (20:1 hexane/EtOAc), affording pure **4kl** (67% yield, 81 mg). Pale yellow oil. *R_f_* = 0.33 (20:1 hexane/EtOAc). ^1^H NMR (300 MHz, CDCl_3_): δ 7.80–7.74 (m, 1H), 7.44–7.36 (m, 1H), 7.23–7.13 (m, 3H), 6.97–6.94 (m, 1H), 6.89–6.84 (m, 2H), 3.79 (s, 3H), 1.70 (s, 6H). ^13^C{^1^H} NMR (75.4 MHz, CDCl_3_): δ 164 (C, *J*_C–F_ = 247.6 Hz), 159.2 (CH), 139.9 (CH), 131.8 (C, *J*_C–F_ = 8.2 Hz), 129.3 (CH), 124.1 (CH, *J*_C–F_ = 4.1 Hz), 124.0 (2 × CH), 119.4 (C, *J*_C–F_ = 18.3 Hz), 116.4 (CH), 115.8 (CH, *J*_C–F_ = 24.1 Hz), 114.6 (CH), 93.1 (C), 83.4 (C), 55.2 (CH_3_), 43.7 (C), 30.6 (2 × CH_3_). ^19^F NMR (282 MHz, CDCl_3_): δ −105.0. HRMS (ESI+) *m*/*z*: [M + H]^+^ calcd for C_18_H_18_FOS, 301.1057; found, 301.1061.

#### (2-Methyloct-3-yn-2-yl) (*p*-Tolyl) Sulfide (**4la**)

Compound **4la** was prepared according to general procedure A (reaction time, 4 h). The crude product was purified by flash column chromatography on silica gel (50:1 hexane/CH_2_Cl_2_), affording pure **4la** (73% yield, 72 mg). Colorless oil. *R_f_* = 0.33 (50:1 hexane/CH_2_Cl_2_). ^1^H NMR (300 MHz, CDCl_3_): δ 7.51 (d, *J* = 8.1 Hz, 2H), 7.15 (dt, *J* = 7.8, 0.7 Hz, 2H), 2.37 (s, 3H), 2.17 (t, *J* = 7.0 Hz, 2H), 1.54–1.28 (m, 10H), 0.90 (t, *J* = 7.2 Hz, 3H). ^13^C{^1^H} NMR (75.4 MHz, CDCl_3_): δ 139.1 (C), 136.9 (2 × CH), 129.4 (C), 129.3 (2 × CH), 84.8 (C), 83.6 (C), 42.4 (C), 31.0 (CH_2_), 30.9 (2 × CH_3_), 22.1 (CH_2_), 21.4 (CH_3_), 18.6 (CH_2_), 13.8 (CH_3_). LRMS (EI) *m*/*z* (%): 123 (100), 246 (M, 55), 231 (30), 216 (33). HRMS (APCI+) *m*/*z*: [M + H]^+^ calcd for C_16_H_23_S, 247.1515; found, 247.1519.

#### (2,4-Diphenylbut-3-yn-2-yl) (Dodecyl) Sulfide (**4bm**)

Compound **4bm** was prepared according to general procedure A (reaction time, 2 h). The crude product was purified by flash column chromatography on silica gel (hexane), affording pure **4bm** (58% yield, 61 mg). Yellow oil. *R_f_* = 0.22 (hexane). ^1^H NMR (300 MHz, CDCl_3_): δ 7.83–7.80 (m, 2H), 7.56–7.53 (m, 2H), 7.42–7.36 (m, 5H), 7.32–7.28 (m, 1H), 2.77–2.68 (m, 1H), 2.54–2.45 (m, 1H), 2.00 (s, 3H), 1.56–1.47 (m, 2H), 1.28–1.23 (m, 18H), 0.91 (t, *J* = 6.73 Hz, 3H). ^13^C{^1^H} NMR (75.4 MHz, CDCl_3_): δ 144.1 (C), 132.3 (2 × CH), 128.9 (2 × CH), 128.8 (2 × CH), 128.5 (C), 127.8 (CH), 127.1 (2 × CH), 123.8 (C), 92.4 (C), 86.1 (C), 47.1 (C), 32.5 (CH_2_), 32.1 (CH_2_), 32.0 (CH_2_), 30.2 (2 × CH_2_), 30.1 (CH_2_), 30.0 (CH_2_), 29.9 (CH_2_), 29.7 (CH_2_), 29.6 (CH_2_), 29.3 (CH_2_), 14.7 (CH_3_), 13.3 (CH_3_). LRMS (EI) *m*/*z* (%): 402 (100), 57 (60), 43 (38), 71 (35), 406 (M^+^, 5). HRMS (ESI+) *m*/*z*: [M + H]^+^ calcd for C_28_H_39_S, 407.2767; found, 407.2770.

#### Benzyl(2-cyclopropyl-4-phenylbut-3-yn-2-yl) Sulfide (**4fn**)

Compound **4fn** was prepared according to general procedure A (reaction time, 2 h). The crude product was purified by flash column chromatography on silica gel (hexane), affording pure **4fn** (71% yield, 81 mg). Yellow oil. *R_f_* = 0.17 (hexane). ^1^H NMR (300 MHz, CDCl_3_): δ 7.61–7.57 (m, 2H), 7.44–7.23 (m, 6H), 7.16–7.09 (m, 2H), 2.37 (s, 2H), 1.62 (s, 3H), 1.21–1.12 (m, 1H), 0.55–0.45 (m, 4H). ^13^C{^1^H} NMR (75.4 MHz, CDCl_3_): δ 139.3 (C), 137.5 (2 × CH), 131.5 (2 × CH), 129.2 (2 × CH), 128.7 (CH), 128.3 (2 × CH), 128.1 (CH), 123.3 (C), 89.5 (C), 85.2 (C), 49.5 (C), 29.3 (CH_2_), 21.5 (CH), 21.0 (CH_3_), 3.7 (CH_2_), 2.5 (CH_2_). LRMS (EI) *m*/*z* (%): 169 (100), 141 (78), 292 (M^+^, 12). HRMS (APCI+) *m*/*z*: [M + H]^+^ calcd for C_20_H_21_S, 293.1358; found, 293.1356.

### Gram Scale Synthesis of Selected Propargyl Sulfides **4**

To a solution of propargyl alcohol 2-methyl-4-phenylbut-3-yn-2-ol **3a** (3.2 g, 1 equiv, 20 mmol) in MeNO_2_ (40 mL, 0.5 M) were added *p*-toluenethiophenol **2a** (3.23 g, 1.3 equiv, 26 mmol) or *p*-bromothiophenol **2d** (3.12 mL, 1.3 equiv, 26 mmol) and *p*-toluenesulfonic acid **1** (76 mg, 0.02 equiv, 5 mol %). The resulting mixture was stirred at rt for 30 min. After the alcohol was consumed, the reaction was quenched with aqueous NaOH (0.5 M, 50 mL). The separated aqueous phase was extracted with CH_2_Cl_2_ (3 × 50 mL). The combined organic layers were dried with anhydrous Na_2_SO_4_ and concentrated under reduced pressure. The residue was purified by silica gel column chromatography (eluent, hexane/EtOAc mixture) to afford the corresponding propargyl sulfides: (2-methyl-4-phenylbut-3-yn-2-yl) (*p*-tolyl) sulfide **4aa** (3.78 g, 71% yield) or (2-bromophenyl) (2-methyl-4-phenylbut-3-yn-2-yl) sulfide **4ad** (5.21 g, 79% yield).

### Synthesis of (3-Phenylprop-2-yn-1-yl) (*p*-Tolyl) Sulfide **5**

Primary propargyl sulfide 1-methyl-4-[(3-phenyl-2-propyn-1-yl)thio]-benzene **5** (CAS Registry No. 2306760-67-6) was prepared via a modified version of a previously described procedure.^30^ Compound 2-propynyl *p*-tolyl sulfide^31^ (0.36 g, 1.1 equiv, 2.2 mmol) was dissolved in diisopropylamine (4 mL, 0.5 M). Then iodobenzene (0.22 mL, 1 equiv, 2 mmol), PdCl_2_Ph_3_ (28 mg, 2 mol %), and CuI (7.6 mg, 2 mol %) were sequentially added. The reaction mixture was allowed to stir at rt for 3 h. The crude was quenched by addition of brine. The separated aqueous phase was extracted with Et_2_O (3 × 10 mL). The combined organic layers were dried over anhydrous Na_2_SO_4_ and concentrated under reduced pressure. The residue was purified by silica gel column chromatography (eluent hexane) to afford the corresponding propargyl sulfide **5**, 1-methyl-4-[(3-phenyl-2-propyn-1-yl)thio]benzene (0.31 g, 65% yield, CAS Registry No. 2306760-67-6). NMR data are in full agreement with previously described data.^30^

#### 1-Methyl-4-[(3-phenyl-2-propyn-1-yl)thio]benzene (**5**)

Brown oil. *R_f_* = 0.17 (hexane). ^1^H NMR (300 MHz, CDCl_3_): δ 7.50–7.49 (m, 2H), 7.46–7.41 (m, 2H), 7.35–7.33 (m, 3H), 7.23–7.20 (m, 2H), 3.86 (s, 2H), 2.41 (s, 3H). ^13^C{^1^H} NMR (75.4 MHz, CDCl_3_): δ 137.4 (C), 137.7 (2 × CH), 137.7 (2 × CH), 137.4 (C), 131.6 (2 × CH), 131.5 (C), 129.8 (2 × CH), 128.3 (2 × CH), 128.2 (CH), 123.1 (C), 85.6 (C), 83.7 (C), 24.6 (CH_2_), 21.2 (CH_3_). LRMS (EI) *m*/*z* (%): 115 (100), 89 (21), 238 (M^+^, 20).

### Synthesis of (1,3-Diphenylprop-2-yn-1-yl) (*p*-Tolyl) Sulfide **6**

Compound **6** was prepared according to general procedure A. The crude product was purified by flash column chromatography on silica gel (hexane), affording pure **6** (88% yield, 111 mg).

#### (1,3-Diphenylprop-2-yn-1-yl) (*p*-Tolyl) Sulfide (**6**)

Pale yellow liquid. *R_f_* = 0.18 (hexane). ^1^H NMR (300 MHz, CDCl_3_): δ 7.56–7.35 (m, 12H), 7.19 (d, *J* = 7.9 Hz, 2H), 5.26 (s, 1H), 2.42 (s, 3H). ^13^C{^1^H} NMR (75.4 MHz, CDCl_3_): δ 138.8 (C) 138.4 (C), 135.1 (2 × CH), 131.7 (2 × CH), 129.7 (C), 129.5 (2 × CH), 128.5 (2 × CH), 128.3 (3 × CH), 128.2 (2 × CH), 127.8 (CH), 123.1 (C),87.7 (C), 86.9 (C), 44.7 (CH), 21.3 (CH_3_). LRMS (EI) *m*/*z* (%): 191 (100), 314 (M^+^, 35), 207 (18). HRMS (ESI+) *m*/*z*: [M + H]^+^ calcd for C_22_H_19_S, 315.1202; found, 315.1203.

### General Procedure B for the Synthesis of Iodothiochromenes **7** by Iodoarylation of Propargyl Thioethers **4**

*N*-Iodosuccinimide (58.5 mg, 1.3 equiv, 0.23 mmol) was added to a solution of propargyl thioether **4** (1 equiv, 0.2 mmol) in CH_2_Cl_2_ (2 mL, 0.1 M) at 0 °C. The reaction mixture was allowed to warm to rt. Then the reaction mixture was allowed to stir overnight (24 h) until the full depletion of the propargyl sulfide was determined by GC-MS. The reaction was quenched by the addition of saturated aqueous Na_2_S_2_O_3_ (10 mL). The aqueous phase was extracted with CH_2_Cl_2_ (3 × 10 mL). The combined organic layer was dried over anhydrous Na_2_SO_4_, filtered, and concentrated under reduced pressure. Then, the residual succinimide was precipitated by the addition of Et_2_O (10 mL) to the crude. The solids were filtered off through a plug of Celite and washed thoroughly with Et_2_O (3 × 30 mL). The filtrate was concentrated in vacuo, affording crude 3-iodothiochromenes **7**, which were purified by column chromatography on silica gel (eluent, hexane/EtOAc mixture) to afford the corresponding pure thiochromenes **7**.

#### 3-Iodo-2,2,6-trimethyl-4-phenyl-2*H*-thiochromene (**7aa**)

Compound **7aa** was prepared according to general procedure B. The crude product was purified by flash column chromatography on silica gel (hexane), affording pure **7aa** (75% yield, 58 mg). Yellow oil. *R_f_* = 0.28 (hexane). ^1^H NMR (300 MHz, CDCl_3_): δ 7.41–7.38 (m, 2H), 7.37–7.34 (m, 1H), 7.33–7.29 (m, 1H), 7.28–7.25 (m, 2H), 7.11–7.08 (m, 2H), 2.34 (s, 3H), 1.31 (s, 6H). ^13^C NMR (75.4 MHz, CDCl_3_): δ 153.7 (C), 152.5 (C), 142.7 (C), 131.0 (C), 129.8 (2 × CH), 129.3 (2 × CH), 128.7 (C), 127.4 (CH), 127.3 (CH), 123.5 (CH), 121.4 (CH), 108.1 (C), 55.1 (C), 24.7 (2 × CH_3_), 21.2 (CH_3_). LRMS (EI) *m*/*z* (%): 392 (M^+^, 100), 393 (23), 265 (18). HRMS (ESI+) *m*/*z*: [M + O + H]^+^ calcd for C_18_H_18_ISO^+^, 409.0119; found, 409.0122.^32^

#### 3-Iodo-6-methoxy-2,2-dimethyl-4-phenyl-2*H*-thiochromene (**7ab**)

Compound **7ab** was prepared according to general procedure B. The crude product was purified by flash column chromatography on silica gel (hexane), affording pure **7ab** (71% yield, 58 mg). Yellow oil. *R_f_* = 0.26 (hexane). ^1^H NMR (300 MHz, CDCl_3_): δ 7.39–7.36 (m, 3H), 7.34–7.25 (m, 3H), 6.85–6.82 (m, 2H), 3.81 (s, 3H), 1.27 (s, 6H). ^13^C{^1^H} NMR (75.4 MHz, CDCl_3_): δ 159.1 (C), 154.5 (C), 152.5 (C), 142.7 (C), 132.4 (2 × CH), 127.3 (CH), 127.2 (CH), 124.8 (C), 123.3 (CH), 121.3 (CH), 114.7 (2 × CH), 105.9 (C), 55.5 (CH_3_), 55.0 (C), 24.9 (2 × CH_3_). LRMS (EI) *m*/*z* (%): 408 (M^+^, 100), 281 (76), 142 (50). HRMS (ESI+) *m*/*z*: [M + H]^+^ calcd for C_18_H_18_IOS, 409.0118; found, 409.0108.

#### 6-Chloro-3-iodo-2,2-dimethyl-4-phenyl-2*H*-thiochromene (**7ac**)

Compound **7ac** was prepared according to general procedure B. The crude product was purified by flash column chromatography on silica gel (hexane), affording pure **7ac** (70% yield, 59 mg). Pale yellow oil. *R_f_* = 0.44 (hexane). ^1^H NMR (300 MHz, CDCl_3_): δ 7.41–7.29 (m, 5H), 7.26–7.21 (m, 3H), 1.30 (s, 6H). ^13^C{^1^H} NMR (75.4 MHz, CDCl_3_): δ 152.6 (C), 152.4 (C), 142.4 (C), 133.6 (C), 132.2 (C), 129.8 (2 × CH), 129.2 (2 × CH), 127.8 (CH), 127.4 (CH), 123.7 (CH), 121.5 (CH), 110.0 (C), 55.2 (C), 24.5 (2 × CH_3_). LRMS (EI) *m*/*z* (%): 412 (M^+^, 100), 285 (62), 142 (53). HRMS (APCI+) *m*/*z*: [M + H]^+^ calcd for C_17_H_15_ClIS, 412.9622; found, 412.9620.

#### 6-Chloro-3-iodo-2,2-dimethyl-4-phenyl-2*H*-thiochromene (**7ad**)

Compound **7ad** was prepared according to general procedure B. The crude product was purified by flash column chromatography on silica gel (hexane), affording pure **7ad** (67% yield, 63 mg). Yellow oil. *R_f_* = 0.6 (hexane). ^1^H NMR (300 MHz, CDCl_3_): δ 7.32–7.42 (m, 6H), 7.16–7.20 (m, 2H), 1.31 (s, 6H). ^3^C{^1^H} NMR (75.4 MHz, CDCl_3_): δ 152.4 (C), 142.4 (C), 134.3 (C), 132.1 (2 × CH), 129.9 (2 × CH), 127.8 (CH), 127.4 (CH), 123.7 (CH), 121.5 (CH), 120.0 (C), 110.0 (C), 55.2 (C), 24.5 (2 × CH_3_), one C peak is missing due to overlapping. LRMS (EI) *m*/*z* (%): 458 (100), 456 (M^+^, 84), 331 (45). HRMS (APCI+) *m*/*z*: [M + H]^+^ calcd for C_17_H_15_BrIS, 456.9117; found, 456.9117.

#### 3-Iodo-2,2-dimethyl-4-phenyl-2*H*-thiochromene (**7ae**)

Compound **7ae** was prepared according to general procedure B. The crude product was purified by flash column chromatography on silica gel (hexane), affording pure **7ae** (74% yield, 56 mg). Pale yellow oil. *R_f_* = 0.33 (hexane). ^1^H NMR (300 MHz, CDCl_3_): δ 7.42–7.40 (m, 1H), 7.39–7.37 (m, 1H), 7.36–7.35 (m, 1H), 7.33–7.31 (m, 2H), 7.30–7.27 (m, 2H), 7.26–7.25 (m, 1H), 7.23–7.19 (m, 1H), 1.32 (s, 6H). ^13^C{^1^H} NMR (75.4 MHz, CDCl_3_): δ 153.2 (C), 152.5 (C), 142.6 (C), 134.9 (C), 129.1 (2 × CH), 128.8 (2 × CH), 127.6 (CH), 127.3 (CH), 126.4 (CH), 123.5 (CH), 121.5 (CH), 109.1 (C), 55.2 (C), 24.6 (2 × CH_3_). LRMS (EI) *m*/*z* (%): 378 (M^+^, 100), 251 (39), 142 (30). HRMS (ESI+) *m*/*z*: [M + H]^+^ calcd for C_17_H_16_IS, 379.0012; found, 379.0006.

#### 3-Iodo-2,2-dimethyl-4-phenyl-2*H*-benzo[*h*]thiochromene (**7ai**)

Compound **7ai** was prepared according to general procedure B. The crude product was purified by flash column chromatography on silica gel (hexane), affording pure **7ai** (60% yield, 52 mg). Pale yellow oil. *R_f_* = 0.26 (hexane). ^1^H NMR (300 MHz, CDCl_3_): δ 8.51–8.47 (m, 1H), 7.91–7.88 (m, 1H), 7.79–7.76 (m, 1H), 7.65–7.57 (m, 2H), 7.56–7.53 (m, 2H), 7.42–7.36 (m, 3H), 7.34–7.33 (m, 1H), 1.23 (s, 6H). ^13^C{^1^H} NMR (75.4 MHz, CDCl_3_): δ 153.4 (C), 152.7 (C), 142.6 (C), 134.1 (C), 132.9 (C), 131.2 (C), 128.7 (2 × CH), 128.1 (CH), 127.3 (2 × CH), 126.7 (CH), 126.4 (CH), 125.6 (CH), 125.1 (CH), 123.4 (CH), 121.3 (CH), 107.0 (C), 55.4 (C), 24.7 (2 × CH_3_). LRMS (EI) *m*/*z* (%): 301 (100), 284 (98), 428 (M^+^, 82). HRMS (ESI+) *m*/*z*: [M + H]^+^ calcd for C_21_H_18_IS, 429.0168; found, 429.0165.

#### 2-Iodo-2,2-dimethyl-1-phenyl-3*H*-benzo[*f*]thiochromene (**7aj**)

Compound **7aj** was prepared according to general procedure B. The crude product was purified by flash column chromatography on silica gel (hexane), affording pure **7ai** (62% yield, 54 mg). Pale yellow oil. *R_f_* = 0.19 (hexane). ^1^H NMR (300 MHz, CDCl_3_): δ 7.92–7.65 (m, 4H), 7.58–7.30 (m, 7H), 1.35 (s, 6H). ^13^C{^1^H} NMR (75.4 MHz, CDCl_3_): δ 153.0 (C), 152.6 (C), 142.6 (C), 133.8 (C), 132.3 (C), 132.0 (C), 128.7 (CH), 127.9 (CH), 127.6 (CH), 127.4 (CH), 127.3 (CH), 127.0 (CH), 126.9 (CH), 126.7 (CH), 126.7 (CH), 125.9 (CH), 123.6 (CH), 121.5 (CH), 109.3 (C), 55.2 (C), 24.6 (2 × CH_3_). LRMS (EI) *m*/*z* (%): 301 (100), 284 (90), 428 (M^+^, 70). HRMS (APCI+) *m*/*z*: [M + H]^+^ calcd for C_21_H_18_IS, 429.0168; found, 429.0172.

#### 3′-Iodo-6′-methyl-4′-phenylspiro[cyclohexane-1,2′-thiochromene] (**7ia**)

Compound **7ia** was prepared according to general procedure B. The crude product was purified by flash column chromatography on silica gel (hexane), affording pure **7ia** (63% yield, 56 mg). Pale yellow oil. *R_f_* = 0.48 (hexane). ^1^H NMR (300 MHz, CDCl_3_): δ 7.80 (d, *J* = 7.50 Hz, 1H), 7.46–7.39 (m, 2H), 7.34–7.29 (m, 1H), 7.16–7.12 (m, 2H), 7.07 (d, *J* = 8.2 Hz, 2H), 2.33 (s, 3H), 2.11–2.01 (m, 2H), 1.96–1.79 (m, 5H), 1.50–1.42 (m, 1H), 1.28–1.25 (m, 2H). ^13^C{^1^H} NMR (75.4 MHz, CDCl_3_): δ 153.8 (C), 151.3 (C), 143.3 (C), 135.7 (C), 132 (C), 129.8 (2 × CH), 128.7 (CH), 127.6 (2 × CH), 126.6 (CH), 124.1 (CH), 123.8 (CH), 110.9 (C), 58.5 (CH), 31.9 (2 × CH_2_), 25.1 (CH_2_), 22.6 (2 × CH_2_), 21.1 (CH_3_). LRMS (EI) *m*/*z* (%): 182 (100), 141 (58), 181 (57), 432 (M^+^, 52). HRMS (ESI+) *m*/*z*: [M – I]^+^ calcd for C_21_H_21_S, 305.1364; found, 305.1358.

#### 3-Iodo-4-(3-methoxyphenyl)-2,2,6-trimethyl-2*H*-thiochromene (**7ka**)

Compound **7ka** was prepared according to general procedure B. The crude product was purified by flash column chromatography on silica gel (100:1 hexane/EtOAc), affording pure **7ka** (61% yield, 52 mg). Colorless oil. *R_f_* = 0.21 (100:1 hexane/EtOAc). ^1^H NMR (300 MHz, CDCl_3_): δ 7.32–7.38 (m, 1H), 7.21–7.24 (m, 2H), 7.03–7.09 (m, 3H), 6.85–6.89 (m, 1H), 3.90 (s, 3H), 2.32 (s, 3H), 1.39 (s, 6H). ^13^C{^1^H} NMR (75.4 MHz, CDCl_3_): δ 155.1 (C), 144.2 (C), 138.2 (C), 136.2 (C), 131.3 (CH), 132.1 (CH), 129.8 (CH), 128.9 (CH), 128.4 (CH), 122.3 (C), 116.2 (CH), 112.2 (C), 110 (CH), 108.9 (C), 55.8 (CH_3_), 55.6 (C), 25.9 (CH_3_), 21.8 (2 × CH_3_). LRMS (EI) *m*/*z* (%): 295 (100), 422 (M^+^, 86), 299 (78), 128 (74). HRMS (APCI+) *m*/*z*: [M – I]^+^ calcd for C_19_H_19_OS, 295.1157; found, 295.1152.

#### 6-Bromo-3-iodo-4-(3-methoxyphenyl)-2,2-dimethyl-2*H*-thiochromene (**7kd**)

Compound **7kd** was prepared according to general procedure B. The crude product was purified by flash column chromatography on silica gel (hexane), affording pure **7kd** (72% yield, 68 mg). Colorless oil. *R_f_* = 0.21 (hexane). ^1^H NMR (300 MHz, CDCl_3_): δ 7.32–7.37 (m, 3H), 7.11–7.15 (m, 2H), 7.04 (d, *J* = 7.4 Hz, 1H), 6.86 (d, *J* = 8.2 Hz, 1H), 3.89 (s, 3H), 1.36 (s, 6H). ^13^C{^1^H} NMR (75.4 MHz, CDCl_3_): δ 159.6 (C), 144.7 (C), 143.7 (C), 134.2 (C), 132.3 (CH), 132.1 (CH), 130 (CH), 129.7 (CH), 122.2 (CH), 120 (C), 114 (CH), 111.9 (C), 108.9 (CH), 106.5 (C), 55.8 (CH_3_), 54.6 (C), 24.7 (2 × CH_3_). LRMS (EI) *m*/*z* (%): 299 (100), 488 (44), 486 (M^+^, 43). HRMS (APCI+) *m*/*z*: [M + H]^+^ calcd for C_18_H_17_BrIOS, 486.9223; found, 486.9223.

#### 8-Fluoro-3-iodo-4-(3-methoxyphenyl)-2,2-dimethyl-2*H*-thiochromene (**7kl**)

Compound **7kl** was prepared according to general procedure B. The crude product was purified by flash column chromatography on silica gel (100:1 hexane/EtOAc), affording pure **7kl** (54% yield, 45 mg). Colorless oil. *R_f_* = 0.29 (100:1 hexane/EtOAc). ^1^H NMR (300 MHz, CDCl_3_): δ 7.26–7.18 (m, 3H), 7.12–7.01 (m, 2H), 6.95 (d, *J* = 2.4 Hz, 1H), 6.89 (dd *J* = 8.2, 2.4 Hz, 1H), 3.90 (s, 3H), 1.30 (s, 6H). ^13^C{^1^H} NMR (75.4 MHz, CDCl_3_): δ 162.3 (C), 159.6 (C), 159.0 (C), 152.6 (C), 144.3 (C, *J*_C–F_ = 73.3 Hz), 131.0 (CH), 128.3 (CH, *J*_C–F_ = 7.6 Hz), 124.5 (CH, *J*_C–F_ = 3.7 Hz), 122.1 (CH), 121.8 (C), 115.8 (CH, *J*_C–F_ = 21.6 Hz), 113.7 (CH), 108.7 (CH), 108.3 (C), 55.8 (CH_3_), 54.6 (C), 24.7 (2 × CH_3_). ^19^F{^1^H} NMR (282 MHz, CDCl_3_): δ −110. LRMS (EI) *m*/*z* (%): 299 (100), 426 (M^+^, 42), 300 (14). HRMS (ESI+) *m*/*z*: [M + H]^+^ calcd for C_18_H_17_FIOS, 427.0023; found, 427.0023.

### General Procedure C for the Synthesis of Thiochromenes **8** by Hydroarylation of Propargyl Thioethers **4**

Propargyl sulfide **4** (1 equiv, 0.2 mmol) was dissolved in 1,2-dichloroethane (1 mL, 0.2 M). Then AgOTf (2.6 mg, 0.05 equiv, 0.01 mmol) was added at once. The obtained suspension was allowed to stir at 85 °C in a preheated bath until full depletion of the propargyl thioether was determined by GC-MS. Then, the reaction mixture was allowed to cool to rt, and hexane (2 mL) was added. The mixture was filtered through a plug of silica and washed with hexane. The filtrate was concentrated under reduced pressure. The crude was purified by column chromatography on silica gel (eluent, hexane/EtOAc mixture) to afford the corresponding thiochromenes **8**.

### General Procedure D for the Synthesis of Thiochromenes **8** by Hydroarylation of Propargyl Thioethers **4** under Microwave Irradiation

Propargyl sulfide **3** (1 equiv, 0.2 mmol) was dissolved in 1,2-dichloroethane (1 mL, 0.2 M) in a microwave tube. Then catalyst AgOTf (2.6 mg, 0.05 equiv, 0.01 mmol) was added at once. The obtained suspension was heated under microwave irradiation at 110 °C for 10 min. Then, the reaction mixture was allowed to cool to rt, and hexane (2 mL) was added. The mixture was filtered through a plug of silica and washed with hexane. The filtrate was concentrated under reduced pressure. The crude was purified by column chromatography on silica gel (eluent, hexane/EtOAc mixture) to afford the corresponding thiochromenes **8**.

#### 2,2,6-Trimethyl-4-phenyl-2*H*-thiochromene (**8aa**)

Compound **8aa** was prepared according to general procedure C. The crude product was purified by flash column chromatography on silica gel (hexane), affording pure **8aa** (83% yield, 42 mg). Pale yellow oil. *R_f_* = 0.31 (hexane). ^1^H NMR (300 MHz, CDCl_3_): δ 7.37–7.33 (m, 3H), 7.31–7.28 (m, 2H), 7.19 (d, *J* = 1.8 Hz, 1H), 6.94 (d, *J* = 7.9 Hz, 1H), 6.85 (dd, *J* = 8.1, 1.8 Hz, 1H), 5.77 (s, 1H), 2.33 (s, 3H), 1.48 (s, 6H). ^13^C{^1^H} NMR (75.4 MHz, CDCl_3_): δ 141.3 (C) 138.8 (C), 137.8 (C), 132.8 (CH), 130.5 (C), 129.4 (2 × CH), 128.8 (C), 128.5 (CH), 128.3 (2 × CH), 127.8 (CH), 127.5 (CH), 126.0 (CH), 40.9 (C), 29.1 (2 × CH_3_), 21.2 (CH_3_), LRMS (EI) *m*/*z* (%): 251 (100), 250 (20), 266 (M^+^, 13). HRMS (ESI+) *m*/*z*: [M + H]^+^ calcd for C_18_H_19_S, 267.1202; found, 267.1203.

#### 6-Methoxy-2,2-dimethyl-4-phenyl-2*H*-thiochromene (**8ab**)

Compound **8ab** was prepared according to general procedure D. The crude product was purified by flash column chromatography on silica gel (hexane), affording pure **8ab** (64% yield, 36 mg). Pale yellow oil. *R_f_* = 0.13 (hexane). ^1^H NMR (300 MHz, CDCl_3_): δ 7.38–7.35 (m, 3H), 7.32–7.29 (m, 2H), 6.99 (d, *J* = 8.6 Hz, 1H), 6.93 (d, *J* = 2.7 Hz, 1H), 6.59–6.63 (m, 1H), 5.71 (s, 1H), 3.83 (s, 3H), 1.50 (s, 6H). ^13^C{^1^H} NMR (75.4 MHz, CDCl_3_): δ 158.9 (C), 141.3 (C), 138.6 (C), 134.8 (C), 131.4 (CH), 129.3 (2 × CH), 129.1 (CH), 128.3 (2 × CH), 127.5 (CH), 126.4 (C), 112.7 (CH), 111.4 (CH), 55.5 (CH_3_), 41.3 (C), 29.1 (2 × CH_3_). LRMS (EI) *m*/*z* (%): 267 (100), 268 (18), 282 (M^+^, 14). HRMS (ESI+) *m*/*z*: [M + H]^+^ calcd for C_18_H_19_OS, 283.1151; found, 283.1158.

#### 2,2-Dimethyl-4-phenyl-2*H*-thiochromene (**8ae**)

Compound **8ae** (CAS Registry No. 132007-64-8) was prepared according to general procedure C. The crude product was purified by flash column chromatography on silica gel (hexane), affording pure **8ae** (78% yield, 40 mg). NMR spectra are in accordance with previously described data.^33^ Pale yellow oil. *R_f_* = 0.25 (hexane). ^1^H NMR (300 MHz, CDCl_3_): δ 7.40–7.37 (m, 4H), 7.33–7.29 (m, 2H), 7.30–7.14 (m, 1H), 7.07–7.04 (m, 2H), 5.84 (s, 1H), 1.50 (s, 6H). ^13^C{^1^H} NMR (75.4 MHz, CDCl_3_): δ 141 (C), 138.8 (C), 133.7 (C), 132.1 (C), 129.2 (2 × CH), 128.2 (2 × CH), 128.1 (CH), 127.9 (CH), 127.8 (CH), 127.6 (CH), 127.5 (CH), 125.0 (CH), 40.7 (C), 28.9 (2 × CH_3_). LRMS (EI) *m*/*z* (%): 237 (100), 238 (17), 252 (M^+^, 13).

#### 7-Methoxy-2,2-dimethyl-4-phenyl-2*H*-thiochromene (**8ah**)

Compound **8ah** was prepared according to general procedure D. The crude product (as a 1.2:1 **8ah/8ah′** mixture) was purified by flash column chromatography on silica gel (hexane), affording **8ah** (with small traces of **8ah′**) (45% yield, 26 mg). Brown oil. *R_f_* = 0.15 (hexane). ^1^H NMR (300 MHz, CDCl_3_): δ 7.63–7.60 (m, 1H), 7.42–7.30 (m, 5H), 6.95 (d, *J* = 2.7 Hz, 1H), 6.85 (dd, *J* = 8.2, 2.7 Hz, 1H), 6.05 (s, 1H), 3.85 (s, 3H), 1.51 (s, 6H). ^13^C{^1^H} NMR (75.4 MHz, CDCl_3_): δ 157.7 (C), 138.4 (C), 133.6 (C), 132.9 (C), 132.2 (CH), 128.6 (2 × CH), 128.3 (CH), 128.2 (CH), 127.5 (C), 126.8 (2 × CH), 112.8 (CH), 111.0 (CH), 55.5 (CH_3_), 37.4 (C), 28.8 (2 × CH_3_). LRMS (EI) *m*/*z* (%): 267 (100), 224 (19), 268 (19), 282 (M^+^, 3). HRMS (ESI+) *m*/*z*: [M + H]^+^ calcd for C_18_H_19_OS, 283.1151; found, 283.1156.

#### 5-Methoxy-2,2-dimethyl-4-phenyl-2*H*-thiochromene (**8ah′**)

Compound **8ah′** was prepared according to general procedure D. The crude product was purified by flash column chromatography on silica gel (hexane), affording pure **8ah′** (with small traces of **8ah**) (35% yield, 20 mg). Brown oil. *R_f_* = 0.18 (hexane). ^1^H NMR (300 MHz, CDCl_3_): δ 7.57–7.54 (m, 2H), 7.38–7.34 (m, 3H), 7.12 (t, *J* = 8 Hz, 1H), 6.84 (dd, *J* = 7.9, 1.2 Hz, 1H), 6.75 (dd, *J* = 8.1, 1.0 Hz, 1H), 5.73 (s, 1H), 3.86 (s, 3H), 1.60 (s, 6H). ^13^C{^1^H} NMR (75.4 MHz, CDCl_3_): δ 159.1 (C), 138.6 (C), 131.4 (C), 130.4 (CH), 128.6 (2 × CH), 128.2 (CH), 127.2 (CH), 126.9 (C), 126.3 (2 × CH), 125.7 (C), 118.9 (CH), 110.1 (CH), 55.4 (CH_3_), 37.7 (C), 29.9 (2 × CH_3_). LRMS (EI) *m*/*z* (%): 267 (100), 252 (25), 268 (17), 282 (M^+^, 6). HRMS (ESI+) *m*/*z*: [M + H]^+^ calcd for C_18_H_19_OS, 283.1151; found, 283.1154.

#### 2,2-Dimethyl-4-phenyl-2*H*-benzo[*h*]thiochromene (**8ai**)

Compound **8ai** was prepared according to general procedure C. The crude product was purified by flash column chromatography on silica gel (hexane), affording pure **8ai** (65% yield, 39 mg). Yellow oil. *R_f_* = 0.24 (hexane). ^1^H NMR (300 MHz, CDCl_3_): δ 8.37–8.34 (m, 1H), 7.83–7.80 (m, 1H), 7.56–7.51 (m, 3H), 7.42–7.38 (m, 3H), 7.36–7.32 (m, 2H), 7.23 (d, *J* = 8.7 Hz, 1H), 5.94 (s, 1H), 1.56 (s, 6H). ^13^C{^1^H} NMR (75.4 MHz, CDCl_3_): δ 141.3 (C), 139.9 (C), 133.1 (CH), 130.9 (C), 130.4 (C), 129.7 (C), 129.4 (2 × CH), 128.5 (C), 128.3 (3 × CH), 127.6 (CH), 126.4 (CH), 126.3 (CH), 125.8 (CH), 125.5 (CH), 124.3 (CH), 41.0 (C), 28.7 (2 × CH_3_). LRMS (EI) *m*/*z* (%): 287 (100), 207 (62), 302 (M^+^, 44). HRMS (ESI+) *m*/*z*: [M + H]^+^ calcd for C_21_H_19_S, 303.1202; found, 303.1202.

#### 5-Methoxy-2,2-dimethyl-4-phenyl-2*H*-thiochromene (**8aj**)

Compound **8aj** was prepared according to general procedure C. The crude (as a 1.25:1 **8aj/8aj′** mixture) was purified by flash column chromatography on silica gel (hexane), affording pure **8aj** (with small traces of **8aj′**) (35% yield, 22 mg). Light brown solid. Mp: 88–90 °C. *R_f_* = 0.20 (hexane). ^1^H NMR (300 MHz, CDCl_3_): δ 8.40–8.37 (m, 1H), 7.85–7.82 (m, 1H), 7.62–7.59 (m, 1H), 7.57–7.48 (m, 3H), 7.44–7.42 (m, 2H), 7.37–7.34 (m, 2H), 7.25 (d, *J* = 8.6 Hz, 1H), 5.81 (s, 1H), 1.58 (s, 6H). ^13^C{^1^H} NMR (75.4 MHz, CDCl_3_): δ 141.3 (C), 139.8 (C), 133.0 (2 × CH), 130.8 (C), 130.3 (C), 129.3 (2 × CH), 128.3 (2 × CH), 127.6 (CH), 126.8 (C), 126.7 (C), 126.3 (CH), 126.2 (CH), 125.7 (CH), 125.4 (CH), 124.2 (CH), 40.9 (C), 28.6 (2 × CH_3_). LRMS (EI) *m*/*z* (%): 287 (100), 288 (20), 302 (M^+^, 17). HRMS (ESI+) *m*/*z*: [M + H]^+^ calcd for C_21_H_19_S, 303.1202; found, 303.1203.

#### 3,3-Dimethyl-1-phenyl-3*H*-benzo[*f*]thiochromene (**8aj′**)

Compound **8aj′** was prepared according to general procedure C. The crude product was purified by flash column chromatography on silica gel (hexane), affording pure **8aj′** (with small traces of **8aj**) (33% yield, 19 mg). Cream-colored solid. Mp: 87–89 °C. *R_f_* = 0.17 (hexane). ^1^H NMR (300 MHz, CDCl_3_): δ 7.80–7.79 (m, 2H), 7.71 (d, *J* = 8.5 Hz, 1H), 7.57 (d, *J* = 8.5 Hz, 1H), 7.29–7.26 (m, 5H), 7.13–7.07 (m, 2H), 6.08 (s, 1H), 1.50 (s, 6H). ^13^C{^1^H} NMR (75.4 MHz, CDCl_3_): δ 143.5 (C), 139.1 (C), 135.5 (CH), 134.8 (C), 132.9 (C), 129.8 (C), 128.5 (2 × CH), 128.4 (CH), 128.1 (CH), 127.9 (C), 127.8 (2 × CH), 127.7 (CH), 127.1 (CH), 126.2 (CH), 125.1 (CH), 124.5 (CH), 41.2 (C), 27.6 (2 × CH_3_). LRMS (EI) *m*/*z* (%): 287 (100), 302 (M^+^, 31). HRMS (ESI+) *m*/*z*: [M + H]^+^ calcd for C_21_H_19_S, 303.1202; found, 303.1203.

#### 4′-(4-Methoxyphenyl)-6′-methylspiro[cyclohexane-1,2′-thiochromene] (**8ha**)

Compound **8ha** was prepared according to general procedure C. The crude product was purified by flash column chromatography on silica gel (100:1 hexane/EtOAc), affording pure **8ha** (61% yield, 41 mg). Colorless oil. *R_f_* = 0.23 (100:1 hexane/EtOAc). ^1^H NMR (300 MHz, CDCl_3_): δ 7.26–7.22 (m, 2H), 7.22–7.21 (m, 1H), 6.97–6.94 (m, 1H), 6.93–6.89 (m, 2H), 6.87–6.84 (m, 1H), 5.82 (s, 1H), 3.87 (s, 3H), 2.33 (s, 3H), 1.91–1.69 (m, 8H), 1.61–1.56 (m, 2H), ^13^C{^1^H} NMR (75.4 MHz, CDCl_3_): δ 159.2 (C), 138.3 (C), 137.6 (C), 133.9 (C), 132.8 (C), 131.4 (C), 131.2 (C), 130.5 (2 × CH), 128.7 (CH), 127.8 (CH), 126.0 (CH), 113.7 (2 × CH), 55.4 (CH_3_), 45.4 (C), 37.0 (CH_2_), 29.1 (CH_2_), 25.9 (CH_2_), 21.9 (2 × CH_2_), 21.2 (CH_3_). LRMS (EI) *m*/*z* (%): 293 (100), 336 (M^+^, 40). HRMS (ESI+) *m*/*z*: [M + H]^+^ calcd for C_22_H_25_OS, 337.1621; found, 337.1628.

#### 4-(3-Methoxyphenyl)-2,2,6-trimethyl-2*H*-thiochromene (**8ja**)

Compound **8ja** was prepared according to general procedure C. The crude product was purified by flash column chromatography on silica gel (40:1 hexane/EtOAc), affording pure **8ja** (52% yield, 32 mg). Pale yellow oil. *R_f_* = 0.25 (40:1 hexane/EtOAc). ^1^H NMR (300 MHz, CDCl_3_): δ 7.25–7.21 (m, 2H), 7.20–7.19 (m, 1H), 6.99–6.96 (m, 1H), 6.94–6.90 (m, 2H), 6.89–6.85 (m, 1H), 5.74 (s, 1H), 3.86 (s, 3H), 2.19 (s, 3H), 1.47 (s, 6H). ^13^C{^1^H} NMR (75.4 MHz, CDCl_3_): δ 159.2 (C), 138.3 (C), 137.7 (C), 133.7 (C), 133.1 (C), 132.1 (CH), 130.7 (C), 130.4 (2 × CH), 129.4 (C), 128.5 (CH), 127.8 (CH), 126.0 (CH), 113.7 (CH), 55.5 (CH_3_), 41.0 (C), 29.1 (2 × CH_3_), 21.2 (CH_3_). LRMS (EI) *m*/*z* (%): 281 (100), 282 (20), 296 (M^+^, 11). HRMS (ESI+) *m*/*z*: [M + H]^+^ calcd for C_19_H_21_OS, 297.1309; found, 297.1308.

#### *N*-[4-(4-Methoxyphenyl)-2,2-dimethyl-2*H*-thiochromen-6-yl]acetamide (**8jk**)

Compound **8jk** was prepared according to general procedure C. The crude product was purified by flash column chromatography on silica gel (2:1 hexane/EtOAc), affording pure **8jk** (56% yield, 38 mg). Yellow oil. *R_f_* = 0.42 (2:1 hexane/EtOAc). ^1^H NMR (300 MHz, CDCl_3_): δ 7.54–7.53 (m, 1H), 7.24–7.20 (m, 3H), 7.04 (d, *J* = 8.5 Hz, 1H), 6.94–6.90 (m, 2H), 5.74 (s, 1H), 3.86 (s, 3H), 2.19 (s, 3H), 1.47 (s, 6H), one H corresponding to the NH group is missing. ^13^C{^1^H} NMR (75.4 MHz, CDCl_3_): δ 168.3 (C), 138 (C), 159.3 (C), 132.1 (C), 130.4 (2 × CH), 129.8 (CH), 128.7 (C), 128.5 (CH), 118.6 (CH), 117.8 (C), 116.4 (C), 114.1 (CH), 113.7 (2 × CH), 55.5 (CH_3_), 53.9 (CH_3_), 41.0 (C), 29.1 (2 × CH_3_). LRMS (EI) *m*/*z* (%): 324 (100), 282 (20), 325 (19), 339 (M^+^, 13). HRMS (ESI+) *m*/*z*: [M + H]^+^ calcd for C_20_H_22_NO_2_S, 340.1366; found, 340.1370.

### Synthesis of AGN 194310 and Synthesis of 4-(4-Ethylphenyl)-2-methylbut-3-yn-2-ol (**11**)

In a Schlenk flask under a N_2_ atmosphere, 1-bromo-4-ethylbenzene **10** (2.7 mL, 1 equiv, 20 mmol) and 2-methylbut-3-yn-2-ol **9** (2.33 mL, 1.2 equiv, 24 mmol) were dissolved in diisopropylamine (40 mL, 0.5 M). Then PdCl_2_(PPh_3_)_2_ (140 mg, 1 mol %) and CuI (38 mg, 1 mol %) were added to the mixture. The obtained solution was heated at 60 °C overnight in an oil bath. The crude was quenched with brine (50 mL), and the separated aqueous phase was extracted with Et_2_O (3 × 50 mL). The combined organic layers were dried with anhydrous Na_2_SO_4_ and concentrated under reduced pressure, and the filtrate was concentrated under reduced pressure. Then, the crude product was purified by silica gel column chromatography (eluent, 10:1 hexane/EtOAc mixture) to afford the alkynol 4-(4-ethylphenyl)-2-methylbut-3-yn-2-ol^34^**11** (1.74 g, 98% yield, CAS Registry No. 155105-68-3).

#### 4-(4-Ethylphenyl)-2-methylbut-3-yn-2-ol (**11**)^34^

Yellow liquid. *R_f_* = 0.3 (10:1 hexane/AcOEt). ^1^H NMR (300 MHz, CDCl_3_): δ 7.39–7.35 (m, 2H), 7.17–7.12 (m, 2H), 2.66 (q, *J* = 7.6 Hz, 2H), 1.66 (s, 6H), 1.25 (t, *J* = 7.6 Hz, 3H), one H corresponding to the OH group is missing, NMR spectra match those previously reported.^34^^13^C{^1^H} NMR (75.4 MHz, CDCl_3_): δ 144.6 (C), 127.8 (2 × CH), 131.6 (2 × CH), 120.0 (C), 93.2 (C), 82.3 (C), 65.6 (C), 31.6 (2 × CH_3_), 28.8 (CH_2_), 15.4 (CH_3_). LRMS (EI) *m*/*z* (%): 43 (100), 173 (52), 115 (30), 188 (M^+^, 12).

### Synthesis of AGN 194310 and Synthesis of (4-Bromophenyl) [4-(4-Ethylphenyl)-2-methylbut-3-yn-2-yl] Sulfide (**12**)

First, *p*-bromothiophenol **2e** (1.97 g, 1.3 equiv, 10.4 mmol) and *p*-toluenesulfonic acid **1** (76 mg, 5 mol %) were added to a previously prepared solution of alkynol **11** (1.46 g, 1 equiv, 8 mmol) in MeNO_2_ (20 mL, 0.5 M). The mixture was allowed to stir for 30 min until full depletion of the alcohol was determined by TLC; spots were visualized using UV–vis and a Ce/Mo reagent as the staining agent. Then, the reaction was quenched by the addition of aqueous NaOH (0.5 M, 30 mL). The separated aqueous phase was extracted with CH_2_Cl_2_ (3 × 30 mL). The combined organic layers were dried over anhydrous Na_2_SO_4_, filtered, and concentrated under reduced pressure. The residue was purified by silica gel column chromatography (eluent, hexane) to afford pure propargyl sulfide **12** (1.81 g, 70% yield).

#### (4-Bromophenyl) [4-(4-Ethylphenyl)-2-methylbut-3-yn-2-yl] Sulfide (**12**)

Yellow liquid. *R_f_* = 0.26 (hexane). ^1^H NMR (300 MHz, CDCl_3_): δ 7.78–7.62 (m, 2H), 7.60–7.56 (m, 2H), 7.31–7.27 (m, 2H), 7.17–7.14 (m, 2H), 2.67 (q, *J* = 7.7 Hz, 2H), 1.64 (s, 6H), 1.26 (t, *J* = 7.6 Hz, 3H). ^13^C{^1^H} NMR (75.4 MHz, CDCl_3_): δ 144.6 (C), 138.4 (2 × CH), 131.9 (C), 131.8 (2 × CH), 131.6 (2 × CH), 128.0 (2 × CH), 124.0 (C), 120.3 (C), 92.9 (C), 83.8 (C), 43.0 (C), 30.6 (2 × CH_3_), 28.9 (CH_2_), 15.5 (CH_3_). LRMS (EI) *m*/*z* (%): 171 (100), 128 (24), 141 (17), 358 (M^+^, 2). HRMS (APCI+) *m*/*z*: [M + H]^+^ calcd for C_19_H_20_BrS^+^, 359.0464; found, 359.0461.

### Synthesis of AGN 194310 and Synthesis of Ethyl 4-[(4-{[4-(4-Ethylphenyl)-2-methylbut-3-yn-2-yl]thio}phenyl)ethynyl]benzoate **14**

Anhydrous triethylamine (8 mL, 0.25 M) was added to a mixture of propargyl sulfide **12** (716 mg, 1 equiv, 2 mmol) and ethyl 4-ethynylbenzoate^35^**13** (522 mg, 1.5 equiv, 3.0 mmol) under a N_2_ atmosphere. Then, PdCl_2_(MeCN)_2_ (26 mg, 5 mol %), tri-*tert*-butylphosphonium tetrafluoroborate (58 mg, 10 mol %), and CuI (19 mg, 5 mol %) were added to the solution. This mixture was allowed to stir at 85 °C overnight (14 h) in an oil bath. The reaction was quenched by the addition of brine (10 mL). The separated aqueous phase was extracted with Et_2_O (3 × 10 mL). The combined organic layers were dried over anhydrous Na_2_SO_4_, filtered, and concentrated under reduced pressure. Then, the crude product was purified by silica gel column chromatography (eluent, 20:1 hexane/EtOAc mixture) to afford ethyl 4-[(4-{[4-(4-ethylphenyl)-2-methylbut-3-yn-2-yl]thio}phenyl)ethynyl]benzoate **14** (750 mg, 83% yield).

#### 4-[(4-{[4-(4-Ethylphenyl)-2-methylbut-3-yn-2-yl]thio}phenyl)ethynyl]benzoate (**14**)

Pale yellow oil. *R_f_* = 0.23 (20:1 hexane/EtOAc). ^1^H NMR (300 MHz, CDCl_3_): δ 1.26 (t, *J* = 7.6 Hz, 3H), 1.44 (t, *J* = 7.1 Hz, 3H), 1.68 (s, 6H), 2.67 (q, *J* = 7.6 Hz, 2H), 4.42 (q, *J* = 7.1 Hz, 2H), 7.18–7.15 (m, 2H), 7.33–7.31 (m, 2H), 7.64–7.54 (m, 4H), 7.75–7.72 (m, 2H), 8.09–8.06 (m, 2H). ^13^C{^1^H} NMR (75.4 MHz, CDCl_3_): δ 166.1 (C) 144.5 (C), 136.5 (2 × CH), 134.0 (C), 131.7 (2 × CH), 131.6 (2 × CH), 131.5 (2 × CH), 130.1 (C), 129.6 (2 × CH), 127.9 (2 × CH), 127.7 (C), 123.4 (C), 120.3 (C), 93.0 (C), 91.9 (C), 90.2 (C), 83.8 (C), 61.2 (CH_2_), 43.1 (C), 30.7 (2 × CH_3_), 28.9 (CH_2_), 15.5 (CH_3_), 14.4 (CH_3_). LRMS (EI) *m*/*z* (%): 420 (100), 151 (51), 150 (38), 452 (M^+^, 3). HRMS (ESI+) *m*/*z*: [M + H]^+^ calcd for C_30_H_29_O_2_S, 453.1902; found, 453.1883.

### Synthesis of AGN 194310 and Synthesis of Ethyl 4-{[4-(4-Ethylphenyl)-2,2-dimethyl-2*H*-thiochromen-6-yl]ethynyl}benzoate **15**

To a solution of propargyl sulfide **14** (181 mg, 1 equiv, 0.4 mmol) in 1,2-dichloroethane (2 mL, 0.2 M) was added AgOTf (5.2 mg, 10 mol %). The reaction mixture was heated under microwave irradiation (128 °C, 150 W, 20 min). After that, the crude was allowed to cool to room temperature, hexane (4 mL) was added, and the mixture was filtered through a plug of silica and washed with hexane. The filtrate was concentrated under reduced pressure. The residue was purified by silica gel column chromatography (eluent, 20:1 hexane/EtOAc mixture) to afford ethyl 4-{[4-(4-ethylphenyl)-2,2-dimethyl-2*H*-thiochromen-6-yl]ethynyl}benzoate **15** (98 mg, 55% yield, CAS Registry No. 229961-27-7). NMR data were in full agreement with previously reported spectra.^10^

#### 4-{[4-(4-Ethylphenyl)-2,2-dimethyl-2*H*-thiochromen-6-yl]ethynyl}benzoate (**15**)

Pale yellow oil. *R_f_* = 0.28 (20:1 hexane/AcOEt). ^1^H NMR (300 MHz, CDCl_3_): δ 8.07–8.03 (m, 2H), 7.61–7.51 (m, 4H), 7.23–7.20 (m, 4H), 7.09 (d, *J* = 8.1 Hz, 1H), 5.88 (s, 1H), 4.41 (q, *J* = 7.1 Hz, 2H), 2.72 (q, *J* = 7.6 Hz, 2H), 1.50 (s, 6H), 1.43 (t, *J* = 7.1 Hz, 3H), 1.30 (t, *J* = 7.6 Hz, 3H). ^13^C{^1^H} NMR (75.4 MHz, CDCl_3_): δ 166.2 (C),138.5 (C), 137.9 (C), 134.5 (C), 133.8 (C), 133.5 (C), 132.3 (C), 131.6 (2 × CH), 131.5 (C), 131.0 (CH), 130.0 (C), 127.9 (2 × CH), 127.8 (CH), 121.9 (CH), 129.6 (2 × CH), 129.3 (2 × CH), 128.3 (CH), 92.1 (C), 89.6 (C), 61.3 (CH_2_), 41.0 (C), 29.0 (2 × CH_3_), 28.7 (CH_2_), 15.7 (CH_3_), 14.5 (CH_3_).

### Synthesis of AGN 194310 and Synthesis of 4-{[4-(4-Ethylphenyl)-2,2-dimethyl-2*H*-thiochromen-6-yl]ethynyl}benzoic Acid **16**

A solution of thiochromene **15** (90 mg, 0.2 mmol) in THF (2 mL) was treated with aqueous NaOH (2 mL, 4 M). The solution was allowed to stir at rt overnight. Then, the mixture was acidified with HCl [10% (w/w) aqueous solution]. The separated aqueous phase was extracted with EtOAc (3 × 10 mL). The combined organic layers were washed with water and brine, dried over anhydrous Na_2_SO_4_, filtered, and concentrated under reduced pressure. The residue was purified by silica gel column chromatography (eluent, 100:1:5 CH_2_Cl_2_/MeOH/HCOOH), affording retinoid acid antagonist AGN194310 **16** (62 mg, 73% yield, CAS Registry No. 229961-45-9). NMR data were in full agreement with previously reported data.^10^

#### 4-{[4-(4-Ethylphenyl)-2,2-dimethyl-2*H*-thiochromen-6-yl]ethynyl}benzoic Acid (**16**, AGN194310)

Pale yellow oil that solidifies upon refrigeration. *R_f_* = 0.28 (100:1:5 CH_2_Cl_2_/MeOH/HCOOH). ^1^H NMR [300 MHz, (CD_3_)_2_CO]: δ 8.09 (d, *J* = 8.4 Hz, 2H), 7.72–7.69 (m, 2H), 7.59–7.58 (m, 1H), 7.59–7.58 (m, 1H), 7.33–7.28 (m, 3H), 7.24–7.21 (m, 2H), 7.10 (d, *J* = 8.1 Hz, 2H), 5.97 (s, 1H), 2.71 (d, *J* = 7.6 Hz, 2H), 1.49 (s, 6H), 1.27 (t, *J* = 7.6 Hz, 3H), one H corresponding to the COOH group is missing. ^13^C{^1^H} NMR [75.4 MHz, (CD_3_)_2_CO]: δ 167.1 (C), 144.8 (C), 139.0 (C),138.6 (C), 135.6 (C), 134.9 (C), 134.3 (C), 133.1 (C), 132.5 (2 × CH), 131.4 (CH), 130.7 (2 × CH), 129.9 (2 × CH), 129.1 (CH), 128.8 (2 × CH), 128.6 (CH), 128.3 (C), 122.7 (CH), 92.3 (C), 90.2 (C), 41.6 (C), 29.2 (CH_2_), 29.0 (2 × CH_3_), 16.1 (CH_3_).

## Supporting Information Available

Copies of ^1^H and ^13^C{^1^H} NMR spectra for all new compounds (PDF)
FAIR data, including the primary NMR FID files, for compounds **4aa–4aj**, **4ba**, **4bm**, **4aa–4ka**, **4jc**, **4jk**, **4kd**, **4kl**, **4fn**, **5**, **6**, **7aa–7ae**, **7ai**, **7ia**, **7kd**, **7kl**, **8aa**, **8ab**, **8ae**, **8ah**, **8ah′**, **8ai**, **8aj**, **8aj′**, **8ha**, **8ja**, **8jk**, **11**, **12**, **13**, **14**, **15**, and **16** (ZIP)
FAIR data, including the primary NMR FID files, for compounds **4al**, **7aj**, and **7ka** (ZIP)
