# Supplementary material for: From Propargylic Alcohols to Substituted Thiochromenes: gem-Disubstituent Effect in Intramolecular Alkyne Iodo/hydroarylation
Source: J Org Chem. 2021 Apr 30;86(10):7078–91. doi: 10.1021/acs.joc.1c00333 (PMC8474117; doi:10.1021/acs.joc.1c00333)

From propargylic alcohols to substituted thiochromenes: *gem*-disubstituent effect in intramolecular alkyne iodo/hydroarylation.

Supporting Information

Noelia Velasco,[†] Anisley Suárez,[†] Fernando Martínez-Lara,[†] Manuel Ángel Fernández-Rodríguez,[‡] Roberto Sanz[†] and Samuel Suárez-Pantiga[†]**

[†]Área de Química Orgánica, Departamento de Química, Facultad de Ciencias, Universidad de Burgos, Pza. Misael Bañuelos s/n, 09001-Burgos (Spain). E-mail S. S.-P. svusarez@ubu.es; R. S.: rsd@ubu.es

[‡] Departamento de Química Orgánica y Química Inorgánica, Instituto de Investigación Química "Andrés M. del Río" (IQAR), Universidad de Alcalá (IRYCIS), 28805 Alcalá de Henares, Madrid, Spain

Table of Contents for the Supporting Information

A. Synthesis of Propargyl Thioethers: Control experiments and optimization. S3

B. Iodoarylation reaction of Propargyl Thioethers: Control experiments and optimization S4

C. Hydroarylation reaction of Propargyl Thioethers: Control experiments and optimization S6

D. Synthesis of AGN194310. .... S9

^{1}H and ^{13}C{^{1}H} NMR spectra S10

A. Synthesis of Propargyl Thioethers: Control experiments and optimization.

Table S1. Influence of different parameters in the thiolation reaction of tertiary propargyl alcohols.

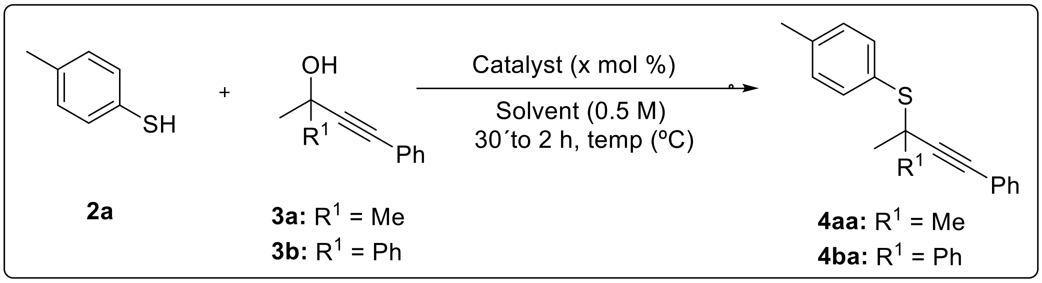

entry	R[1]	2a (equiv)	cat.	cat. (mol %)	solvent	temp (ºC)	t (h)	conversion	yield (%)[a]
**1**[b]	Me	1.3	PTSA	5	MeCN	80	2	100	54
**2**[b]	Me	1.3	PTSA	5	MeCN	rt	5	100	60
**3**[b]	Me	1.3	PTSA	5	CH_2Cl_2	rt	2	100	35
**4**	Me	1.3	PTSA	5	$MeNO_2$	rt	2	100	83
**5**	Me	1.1	PTSA	5	$MeNO_2$	rt	2	100	74
**6**[b]	Me	1.1	PTSA	5	MeCN	rt	5	100	53
**7**[b]	Me	1.5	PTSA	5	MeCN	rt	5	100	62
**8**[b]	Me	1.3	PTSA	10	MeCN	rt	5	100	32
**9**	Me	1.3	PTSA	5	$MeNO_2$	rt	0.5	100	84 (81)[c]
**10**	Me	1.3	none	none	$MeNO_2$	rt	2	100	–
**11**	Ph	1.3	PTSA	5	MeCN	rt	2	100	87
**12**	Ph	1.3	PTSA	5	$MeNO_2$	rt	0.5	100	92 (90)[c]
**13**	Ph	1.1	PTSA	5	$MeNO_2$	rt	2	100	65
**14**	Ph	1.3	PTSA	5	MeCN	rt	2	100	80
**15**	Me	1.3	AuCl	5	$MeNO_2$	rt	2	100	69
**16**	Me	1.3	$NaAuCl_4$	5	$MeNO_2$	rt	2	100	67
**17**[d]	Me	1.3	$MgCl_2$	5	$MeNO_2$	rt	24	34	15
**18**	Me	1.3	CF_3CO_2H	5	$MeNO_2$	rt	15	81	50[e]
**19**	Me	1.3	CF_3CO_2H	5	$MeNO_2$	rt	2	40	27
**20**	Me	1.3	PTSA	5	DMF	rt	7	100	52

Reaction conditions: propargyl alcohol **3a** or **3b** (0.2 mmol, 1 equiv.), thiophenol **2a** (0.26 mmol, 1.3 equiv) and PTSA (5 mol %) in $MeNO_2$ (0.4 mL) at room temperature.

[a]Yield determined by ^{1}H NMR (300 Hz) analysis employing CH_2Br_2 as internal standard. [b]From GC-MS and NMR analysis of the crude, the formation of 1,3 enyne (3-methylbut-3-en-1-yn-1-yl)benzene and of some amounts of *2a* disulfide derivative was observed. Additionally, under extended reaction times hydrothiolation of the alkyne was also

observed. [c]Yield after column chromatography. [c]Stoichiometric amounts of $MgCl_2$ or $CaCl_2$ do not improved the observed yields.[1] [d]Formation of multiple unidentified by-products was observed.

different parameters in the thiolation reaction of tertiary propargyl alcohols.

B. Iodoarylation reaction of Propargyl Thioethers: Control experiments and optimization

Table S2. preliminary experiments of the iodoarylation of propargyl thioethers.

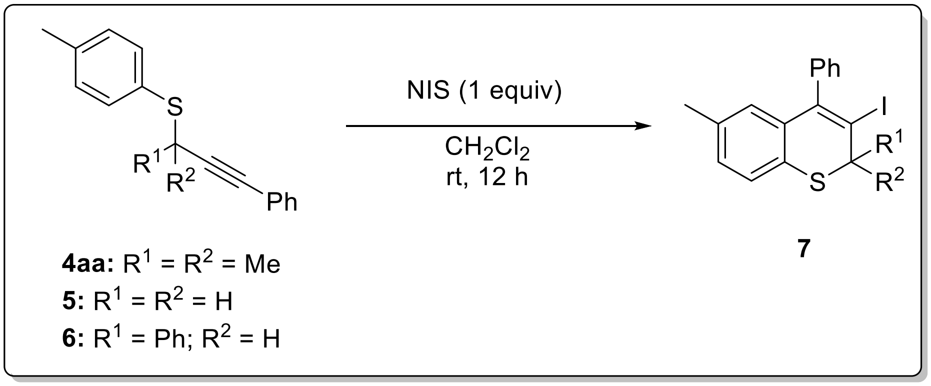

Entry	Sulfide	I+	Yield (%)[a]
**1**	**4aa**	NIS	42
**2**	**5**	NIS	Not observed
**3**	**6**	NIS	Not observed

Reaction conditions: propargyl thioether **4aa**, **5**, or **6** (0.1 mmol, 1 equiv.), *N*-iodosuccinimide (0.13 mmol, 1.3 equiv) in CH_2Cl_2 (1 mL) at room temperature overnight.
[a]Yield determined by ^1H NMR (300 Hz) employing CH_2Br_2 as internal standard.

[1] For activation of alcohols using alkaline earth metal chlorides salts see: (*a*) Dharanipragada, R.; Ferguson, S. B.; Diederich, F., A novel optically active host: design, computer graphics, synthesis, and diastereomeric complex formation in aqueous solution. *J. Am. Chem. Soc.* **1988,** 110, 1679–90. (b) Van Pham, T.; McClelland, R. A., The nature of the transition state in diarylmethyl cation - nucleophile combination reactions as probed by secondary α-deuterium isotope effects. *Can. J. Chem.* **2001,** 79 (12), 1887–1897.

Table S3. Optimization of in the iodoarylation reaction of propargyl thioethers.

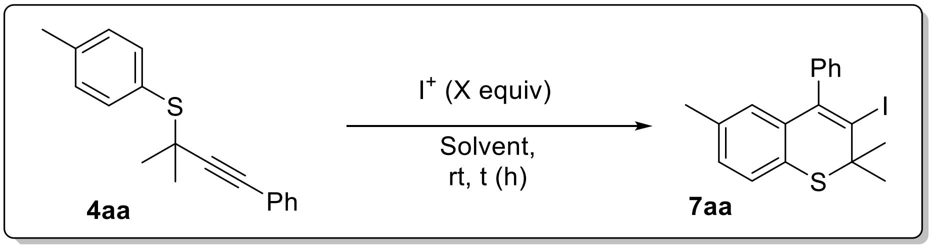

**entry**	**I+**	**X (equiv)**	**additive**	**solvent**	**t (h)**	**yield (%)**[a]
**1**	NIS	1.1	–	CH_2Cl_2	24	51
**2**	NIS	1.3	–	CH_2Cl_2	24	76 (74)[b]
**3**[c]	NIS	1.1	$BF_3.OEt_2$	CH_2Cl_2	24	56
**4**[c]	NIS	1.3	$BF_3.OEt_2$	CH_2Cl_2	24	60
**5**[d]	NIS	1.3	AcOH	CH_2Cl_2	24	50
**6**[e]	NIS	1.3	–	1,2-DCE	24	28
**7**	NIS	1.3	–	$MeNO_2$	24	32
**8**	I_2	1.3	–	CH_2Cl_2	24	–
**9**[f]	I_2	1.3	K_2CO_3	CH_2Cl_2	24	35
**10**[g]	I_2	1.3	Na_2CO_3	CH_2Cl_2	24	32

Reaction conditions: propargyl thioether **4aa** (0.1 mmol, 1 equiv.), *N*-iodosuccinimide (0.13 mmol, 1.3 equiv) in the noted solvent (1 mL) at room temperature.

[a]Yield determined by ^{1}H NMR (300 Hz) analysis, employing CH_2Br_2 as internal standard.

[b]Yield after column chromatography.

[c]$BF_3.OEt_2$ as additive, proportion NIS/ $BF_3.OEt_2$ = 1:1. $BF_3.OEt_2$ (0.11 mmol, 1.1 equiv) in entry 3, $BF_3.OEt_2$ (0.13 mmol, 1.3 equiv) in entry 4.

[d]AcOH as additive, proportion NIS/CH_3COOH = 1:1. CH_3COOH (0.13 mmol, 1.3 equiv).

[e]A precipitate of succinimide was observed making the reaction sluggish.

[f]K_2CO_3 as additive, proportion I_2/ K_2CO_3 = 1:1. K_2CO_3 (0.13 mmol, 1.3 equiv).

[g]Na_2CO_3 as additive, proportion I_2/ Na_2CO_3 = 1:1. Na_2CO_3 (0.13 mmol, 1.3 equiv).

C. Hydroarylation reaction of Propargyl Thioethers: Control experiments and optimization.

Table S4. Control experiments and catalyst optimization of hydroarylation reaction of **4aa**.

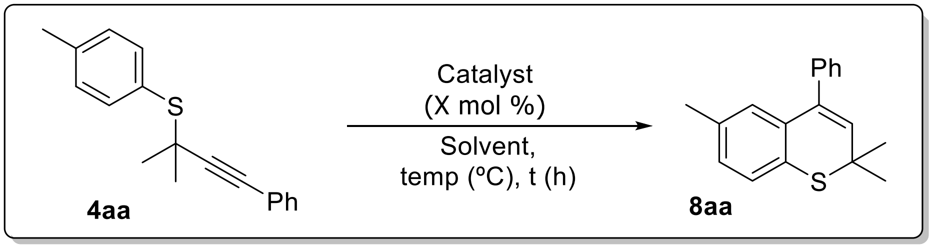

entry	catalyst	cat. (mol %)	solvent	temp (ºC)	t (h)	yield (%)[a]
**1**	$IPrAuNTf_2$	5	1,2-DCE	85	24	–[d]
**2**	$JohnPhosAuNTf_2$	5	1,2-DCE	85	24	–[d]
**3**	$L1AuClN_2/AgOTf$	5	1,2-DCE	85	24	51
**4**	L2AuCl/AgOTf	5	1,2-DCE	85	24	61
**5**	IPrAuCl/AgOTf	5	1,2-DCE	85	5	80
**6**	AuCl	5	1,2-DCE	85	5	–[d]
**7**	$NaAuCl_4$	5	1,2-DCE	85	5	–[d]
**6**	AgOTf	5	1,2-DCE	85	1	79
**7**	AgOTf	5	1,2-DCE	85	5	86 (83)[b]
**8**[c]	AgOTf	5	1,2-DCE	85	5	85
**9**	AgOTf	5	1,2-DCE	rt	24	–
**10**	AgOTf	5	1,2-DCE	60	24	65
**11**	$Bi(OTf)_3$	5	1,2-DCE	85	5	45
**12**	$Sc(OTf)_3$	5	1,2-DCE	85	5	28
**13**	$AgSbF_6$	5	1,2-DCE	85	5	8
**14**	$AgBF_4$	5	1,2-DCE	85	5	5<
**15**	$AgNTf_2$	5	1,2-DCE	85	5	21
**16**	AgOTs	5	1,2-DCE	85	24	–

**17**	AgOAc	5	1,2-DCE	85	24	–
**18**	TfOH	5	1,2-DCE	85	5	40
**19**	TfOH	1	1,2-DCE	85	5	36
**20**	TfOH	0.5	1,2-DCE	85	24	19
**21**	TfOH	0.1	1,2-DCE	85	24	–
**22**	PTSA	5	1,2-DCE	85	24	15

Reaction conditions: propargyl thioether **4aa** (0.1 mmol, 1 equiv.), catalyst (0.1–5 mol %) in 1,2-dichloroethane (1 mL), under heating at 85 ºC (reflux).
[a]Yield determined by ^{1}H NMR (300 Hz) analysis, employing CH_2Br_2 as internal standard.
[b]Yield after column chromatography.
[c]Reaction carried out under inert atmosphere (N_2), using a Schlenk flask.
[d]No desired thiochromene **8aa** was observed, instead, several unidentified by-products were formed.

Scheme S1. Different gold catalysts.

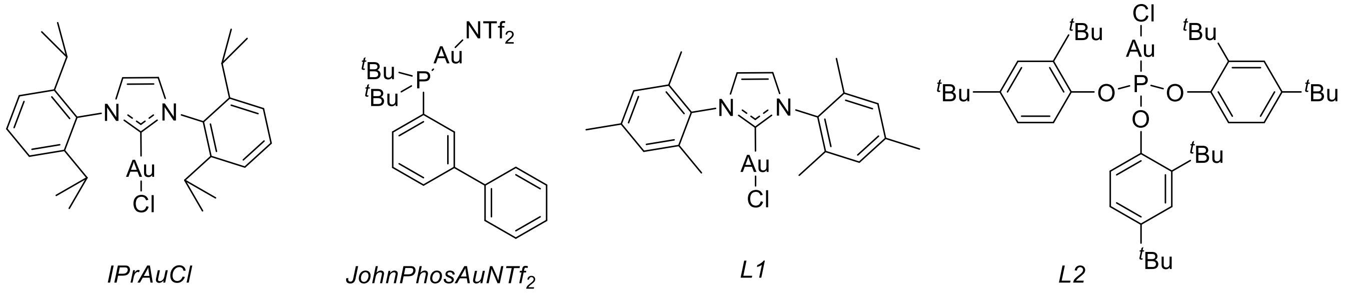

IPrAuCl *JohnPhosAuNTf$_2$* *L1* *L2*

Table S5. Solvent optimization.

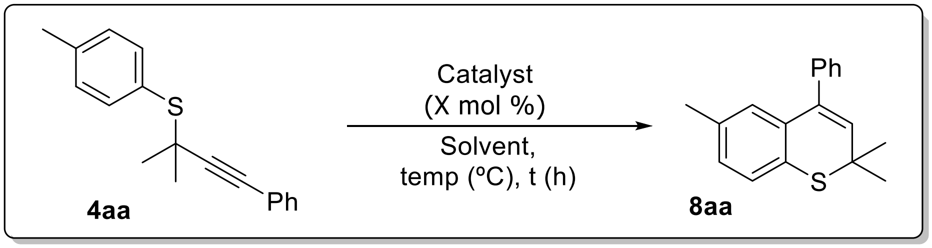

entry	catalyst	cat. (mol %)	solvent	temp (ºC)	t (h)	yield (%)[a]
**1**	AgOTf	5	Toluene	85	5	18
**2**	AgOTf	5	AcOEt	85	5	27
**3**	AgOTf	5	1,2-dioxane	85	5	10
**4**	AgOTf	5	$MeNO_2$	85	5	–
**5**	AgOTf	5	1,2-DCE	85	5	86 (83)[b]

Reaction conditions: propargyl thioether **4aa** (0.1 mmol, 1 equiv.), silver trifluoromethanesulfonate (5 mol %) in 1,2-dichloroethane (1 mL), heating at 85 ºC.
[a]Yield determined by ^{1}H NMR (300 Hz) analysis, employing CH_2Br_2 as internal standard.
[b]Yield after column chromatography.

Table S6. Optimization under microwave irradiation:

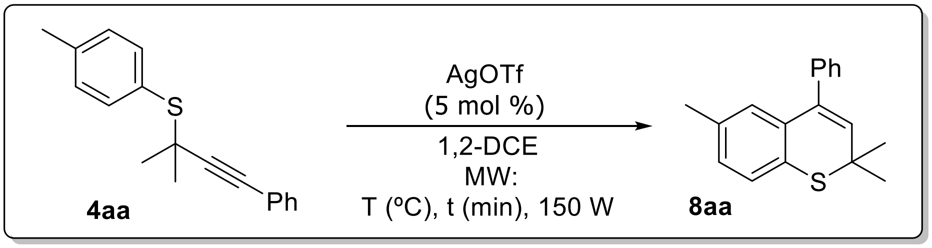

**Entry**	**temp (ºC)**	**t (min)**	**Yield (%)[a]**
**1**	130	30	42
**2**	110	30	57
**3**	110	15	80
**4**	110	10	81

Reaction conditions: propargylic thioether **4aa** (0.1 mmol, 1 equiv.), silver trifluoromethanesulfonate (5 mol %) in 1,2-dichloroethane (1 mL), under microwave irradiation at 110 ºC, 10 min.
[a]Yield determined by ^{1}H NMR (300 Hz) analysis, employing CH_2Br_2 as internal standard.

D. Synthesis of AGN194310.

Scheme S2. Synthesis of AGN194310.

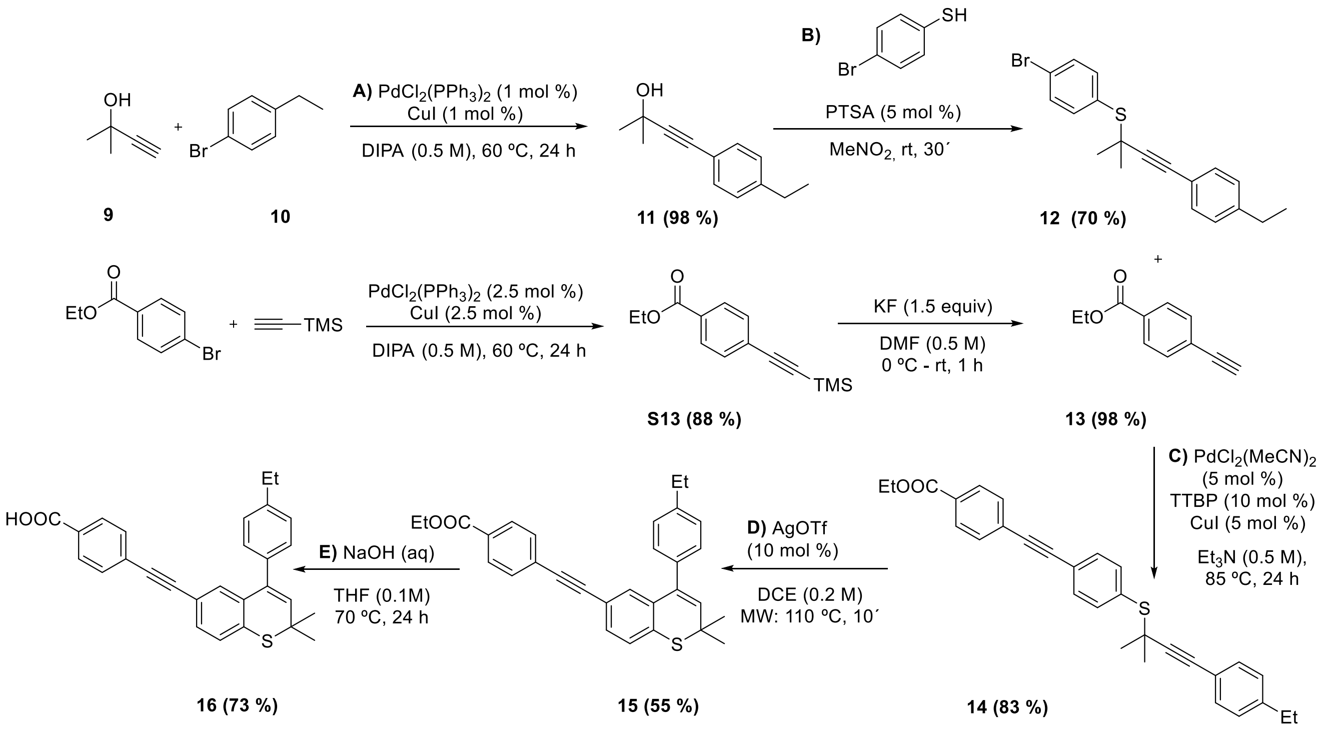

^{1}H and $^{13}C\{^{1}H\}$ NMR spectra

4aa: ^{1}H-NMR (300 MHz, $CDCl_3$)

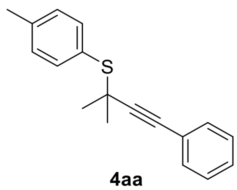

4aa

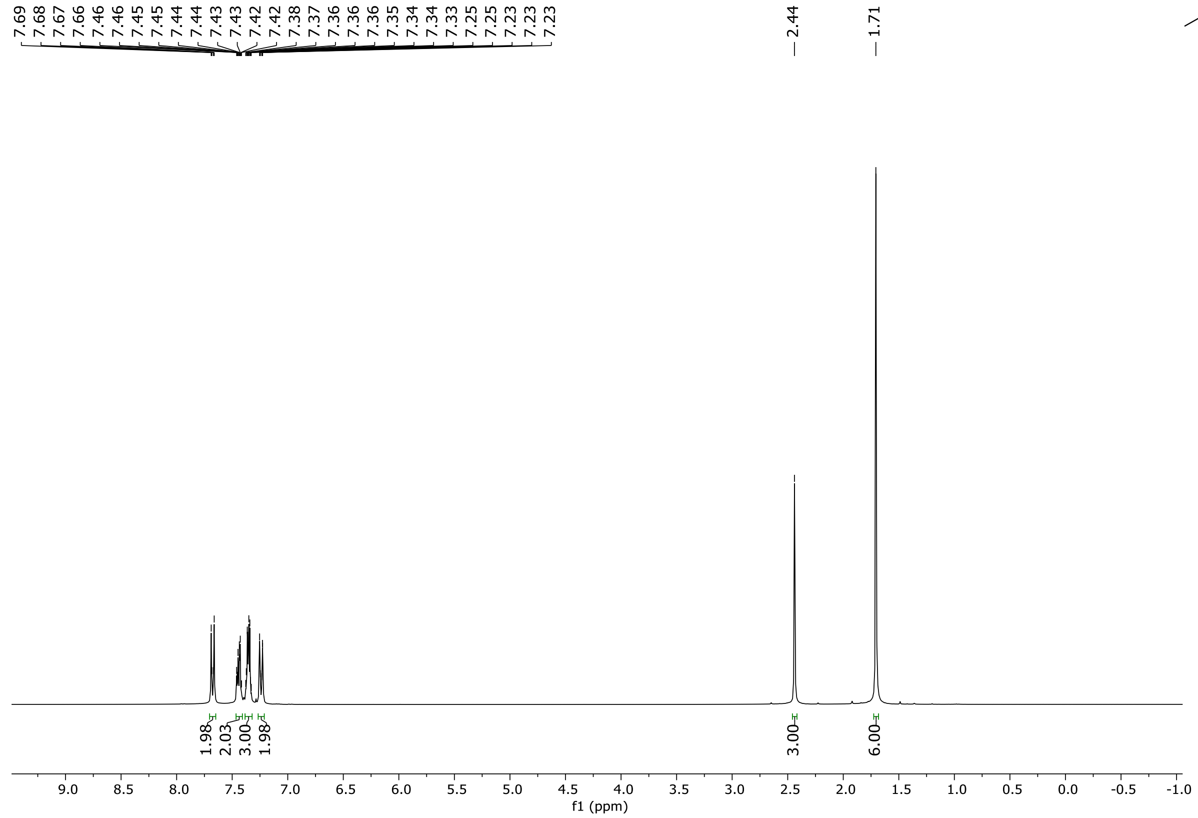

^{13}C{^{1}H} NMR (75.4 MHz, $CDCl_3$)

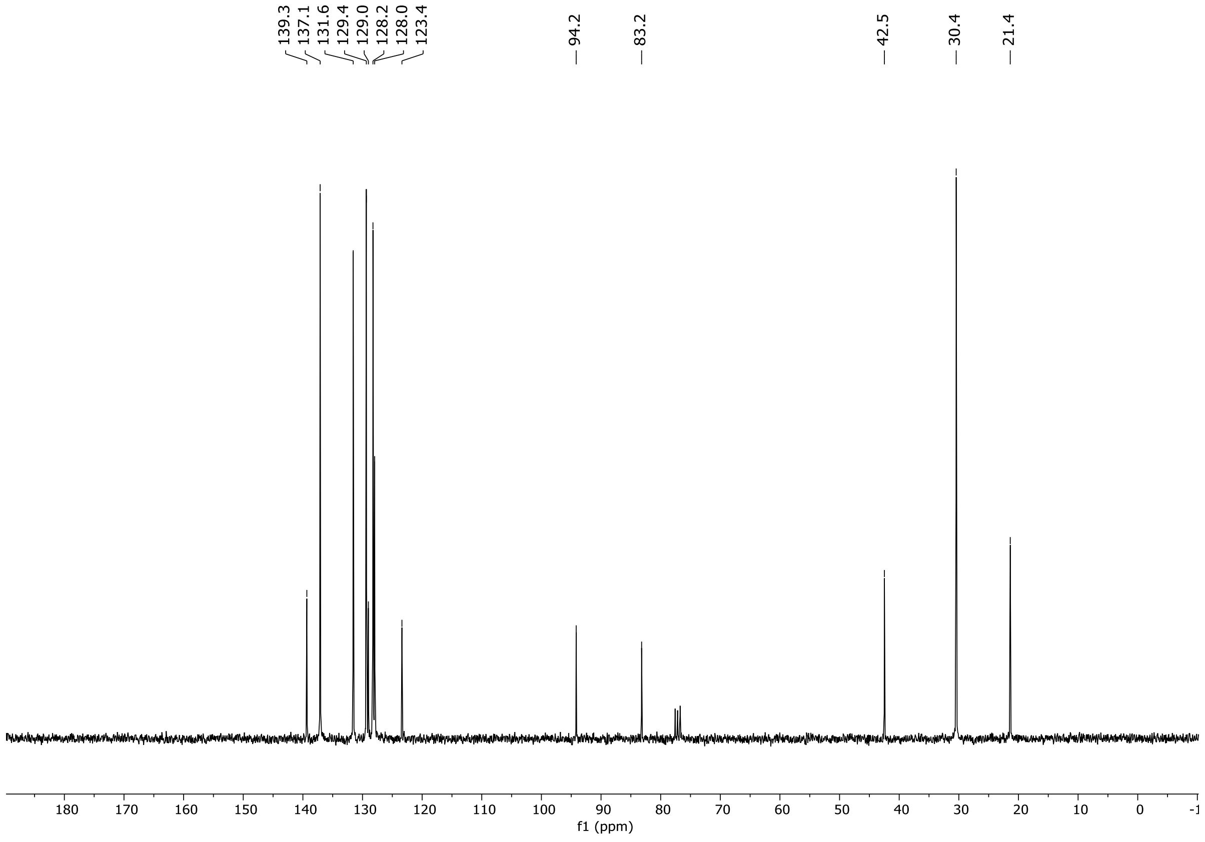

4ab: ^{1}H-NMR (300 MHz, $CDCl_3$)

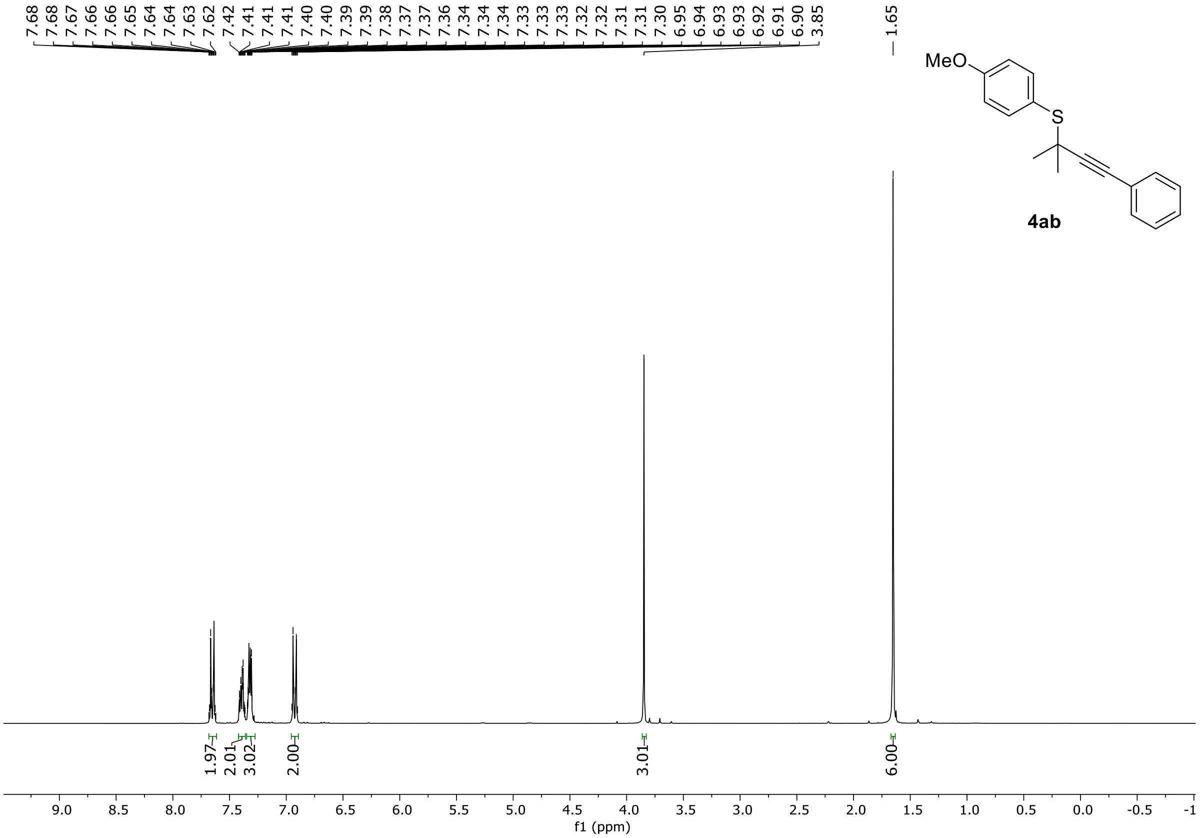

^{13}C{^{1}H} NMR (75.4 MHz, $CDCl_3$)

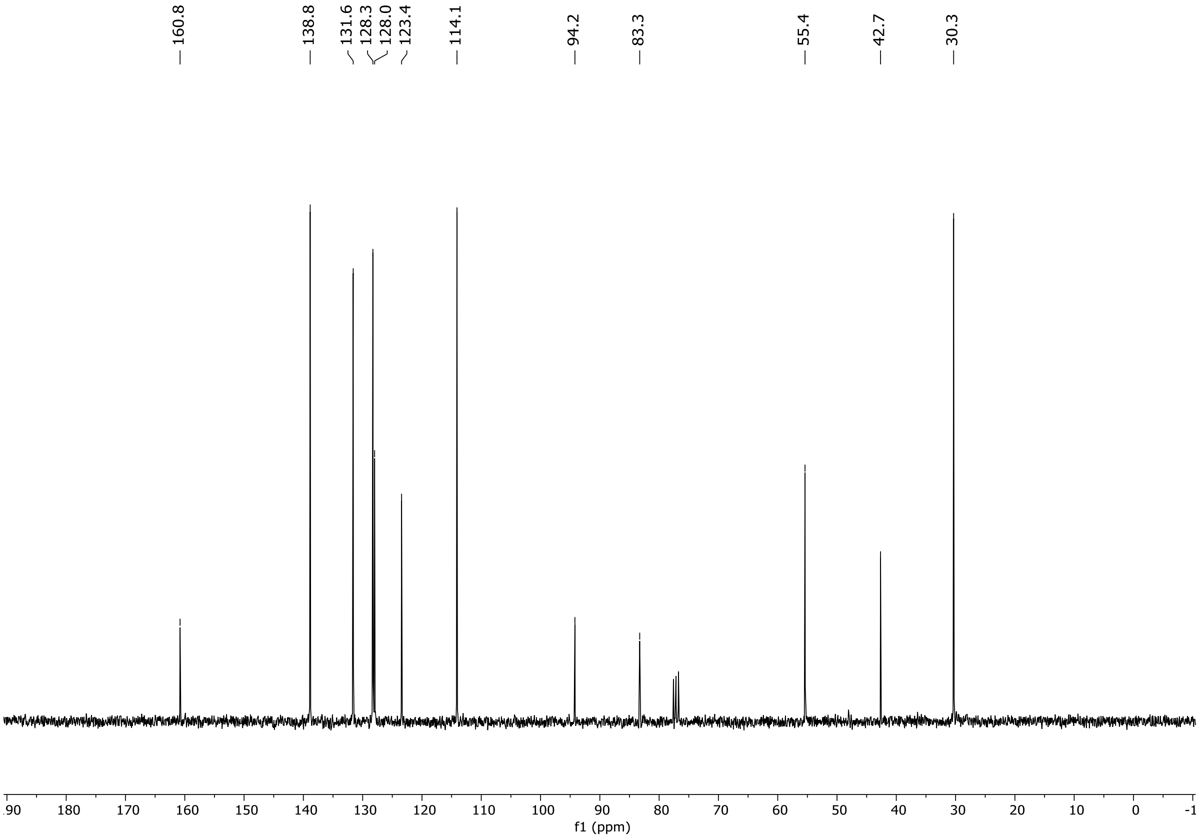

4ac: ^{1}H-NMR (300 MHz, CDCl$_3$)

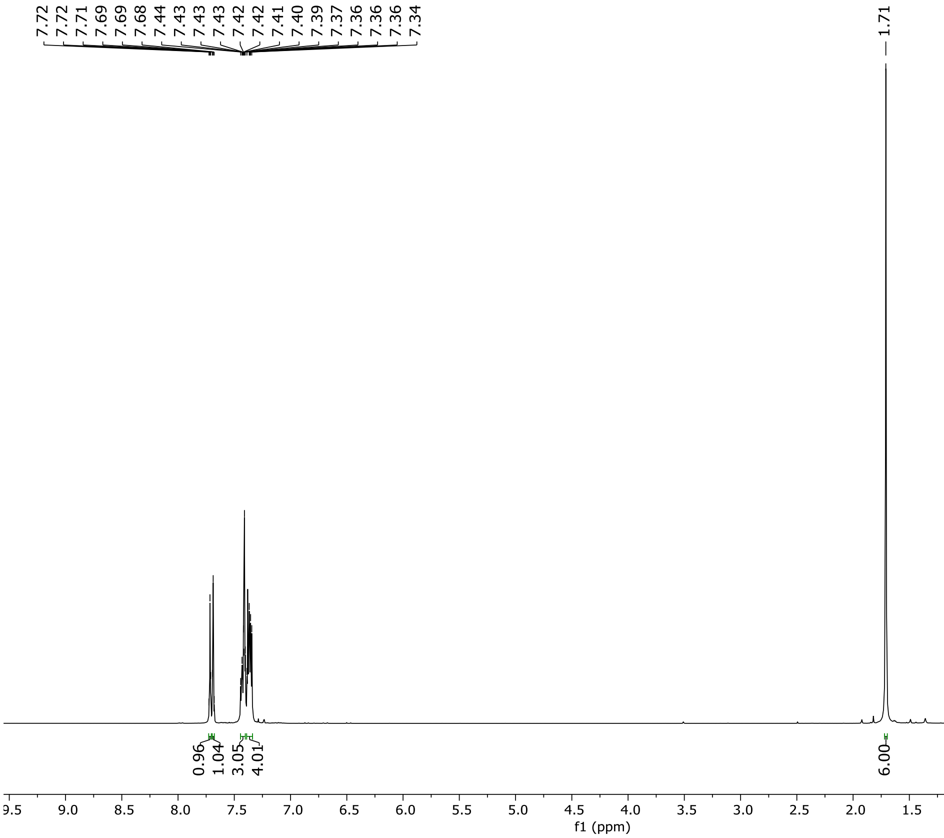

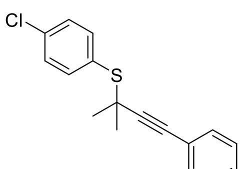

4ac

^{13}C{^{1}H} NMR (75.4 MHz, CDCl$_3$)

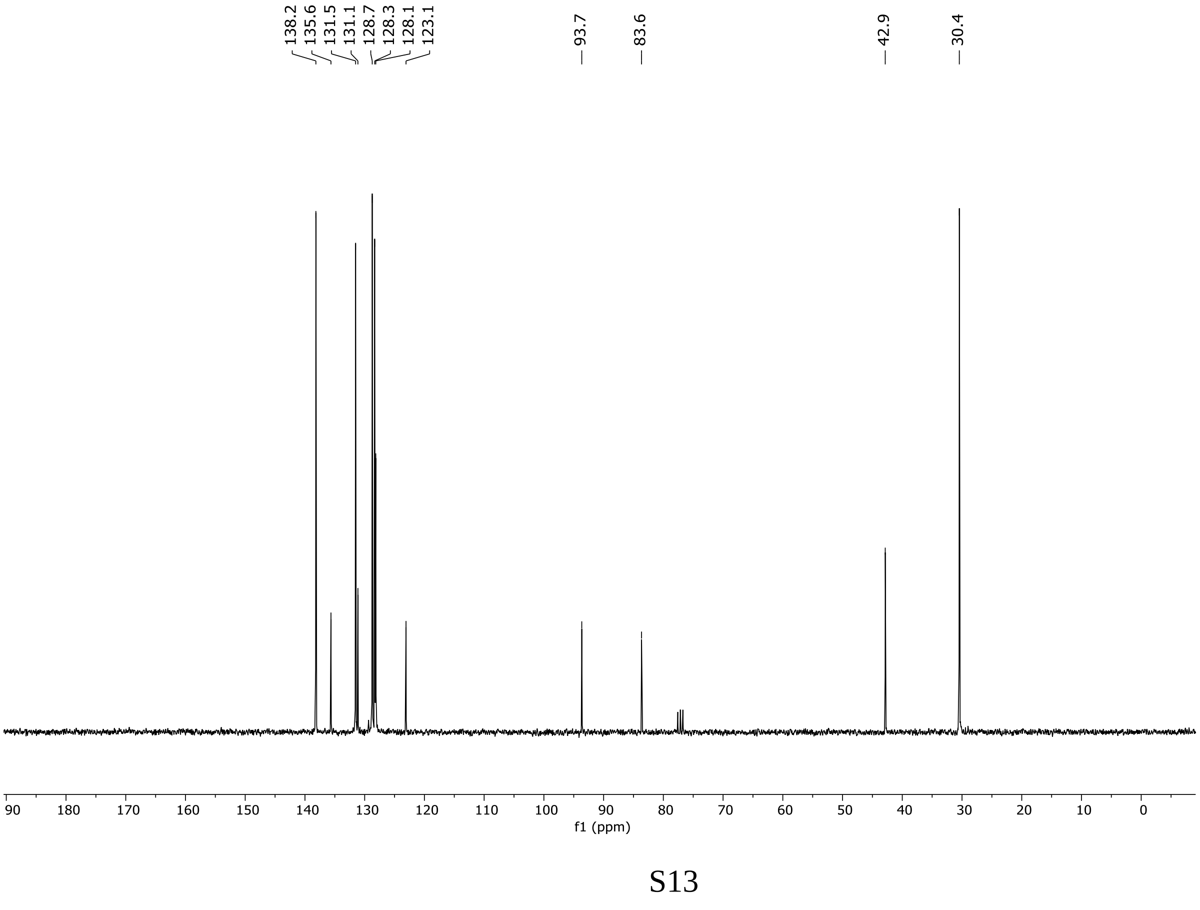

4ad: ^{1}H-NMR (300 MHz, $CDCl_3$)

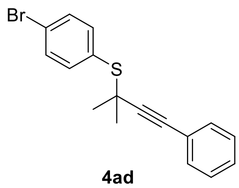

4ad

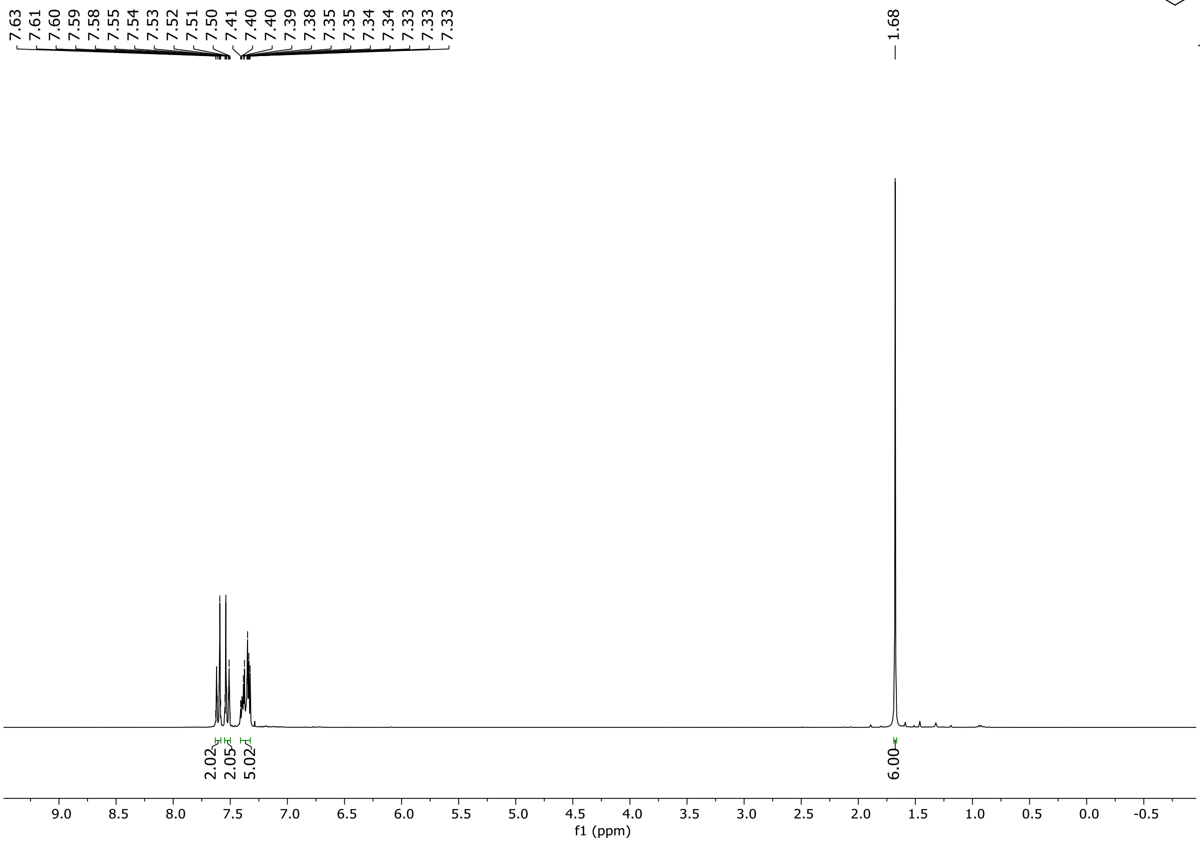

^{13}C{^{1}H} NMR (75.4 MHz, $CDCl_3$)

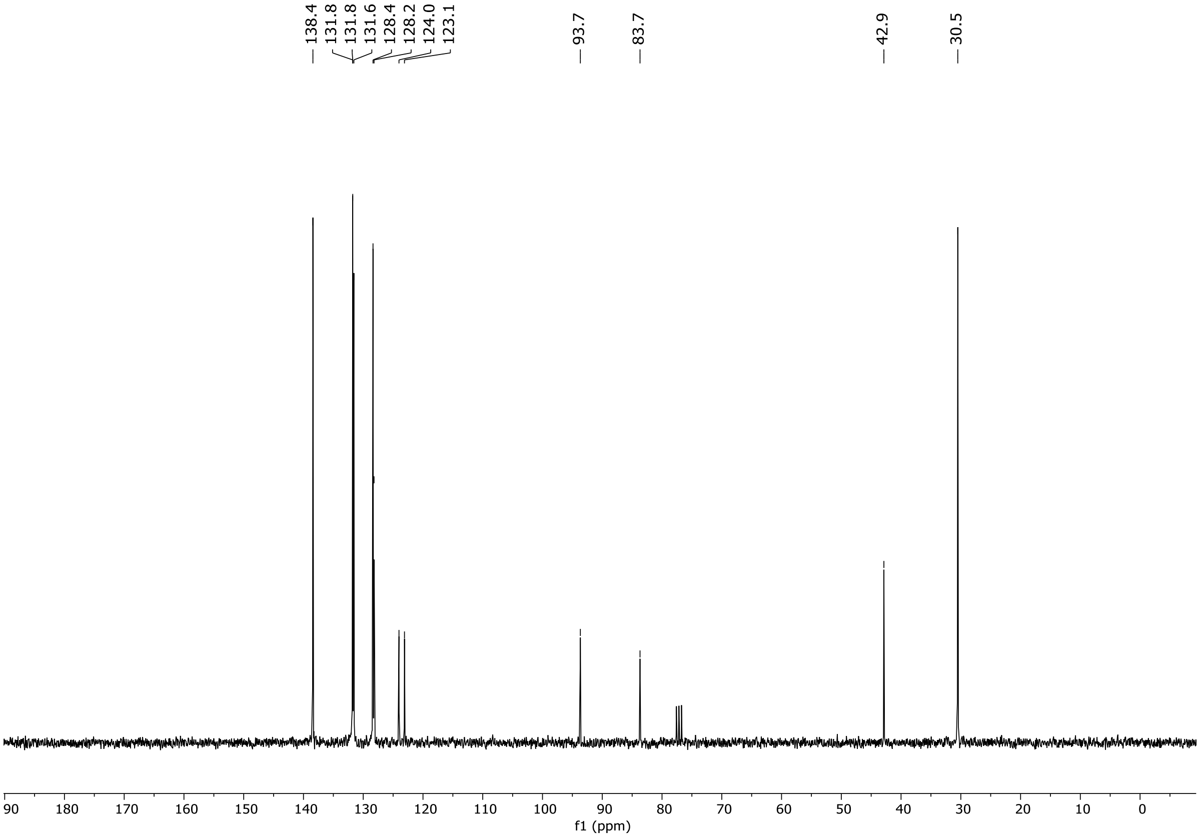

4af: ^{1}H-NMR (300 MHz, CDCl$_3$)

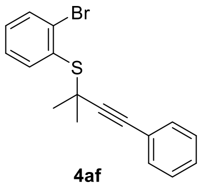

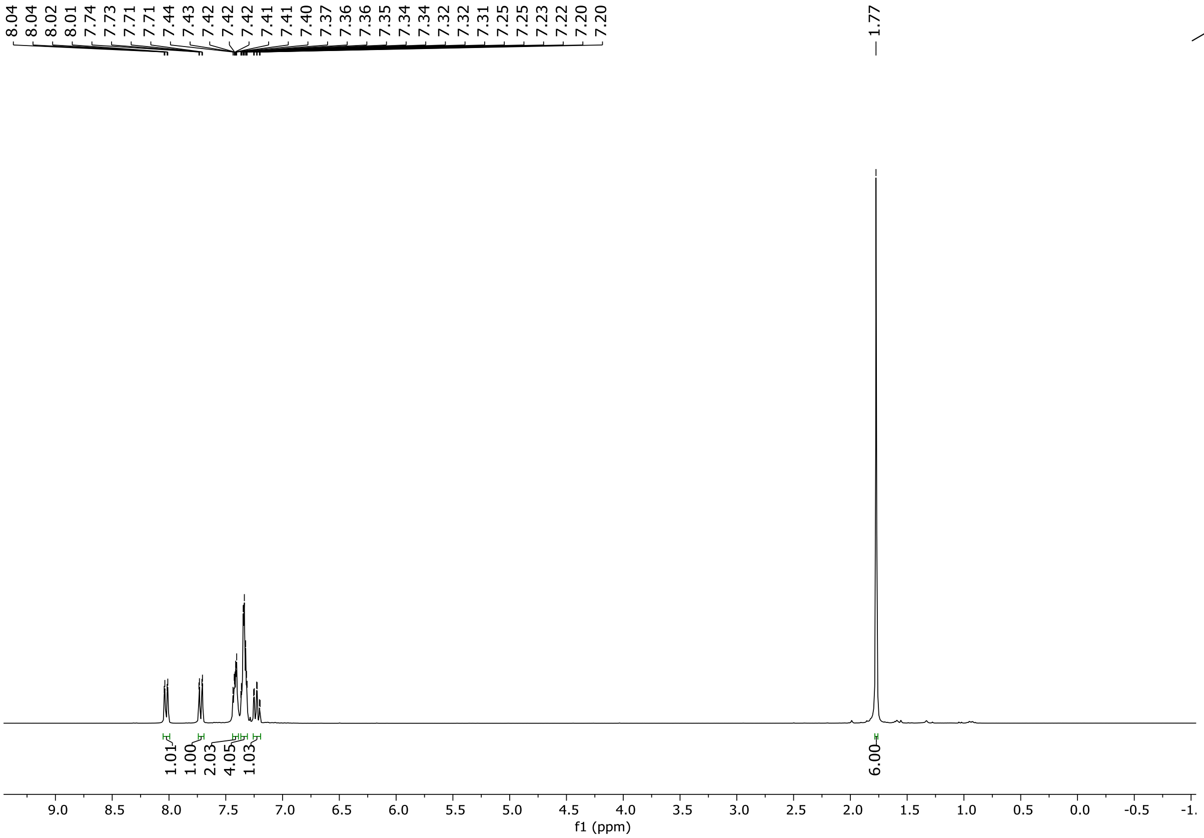

^{13}C{^{1}H} NMR (75.4 MHz, CDCl$_3$)

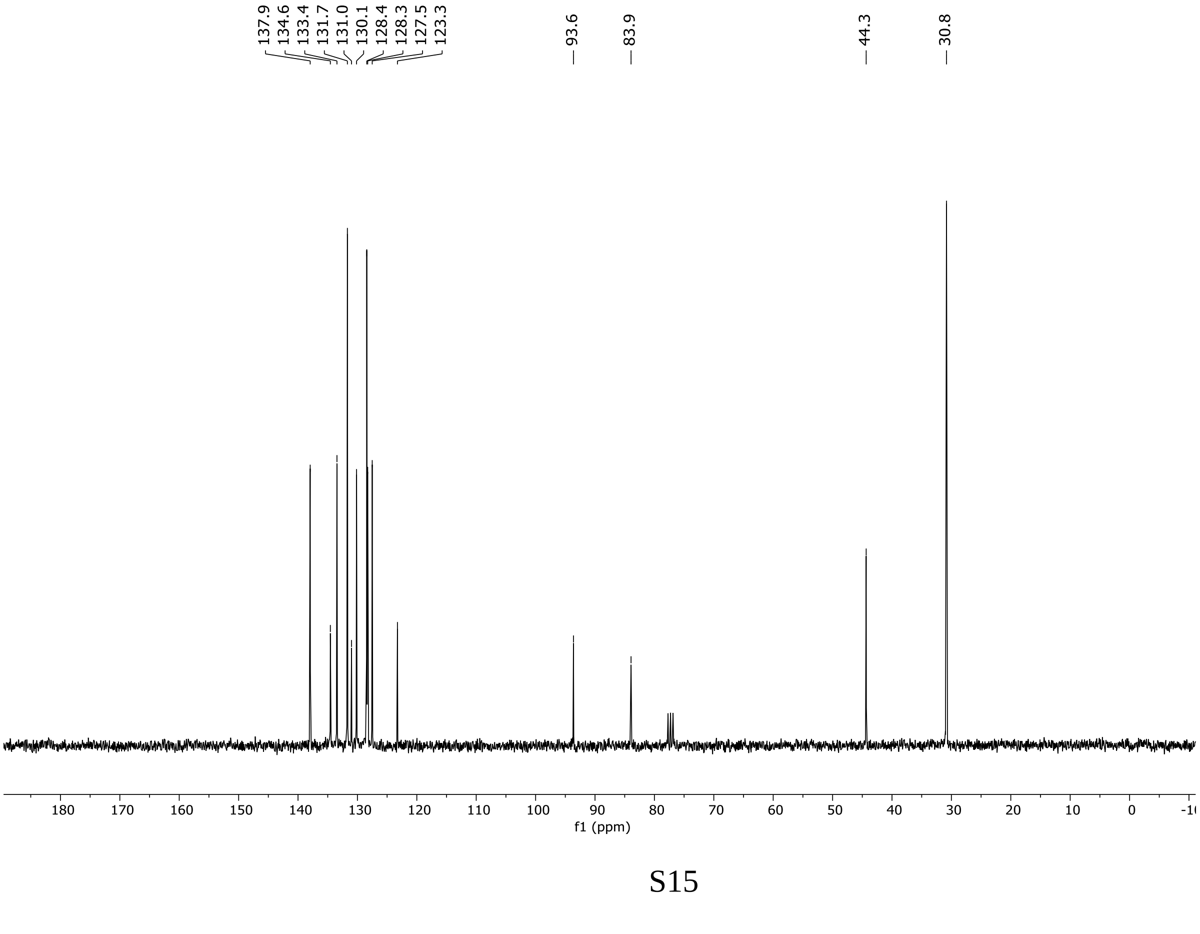

4ag: ^{1}H-NMR (300 MHz, $CDCl_3$)

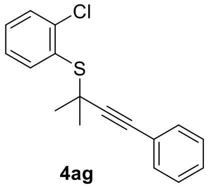

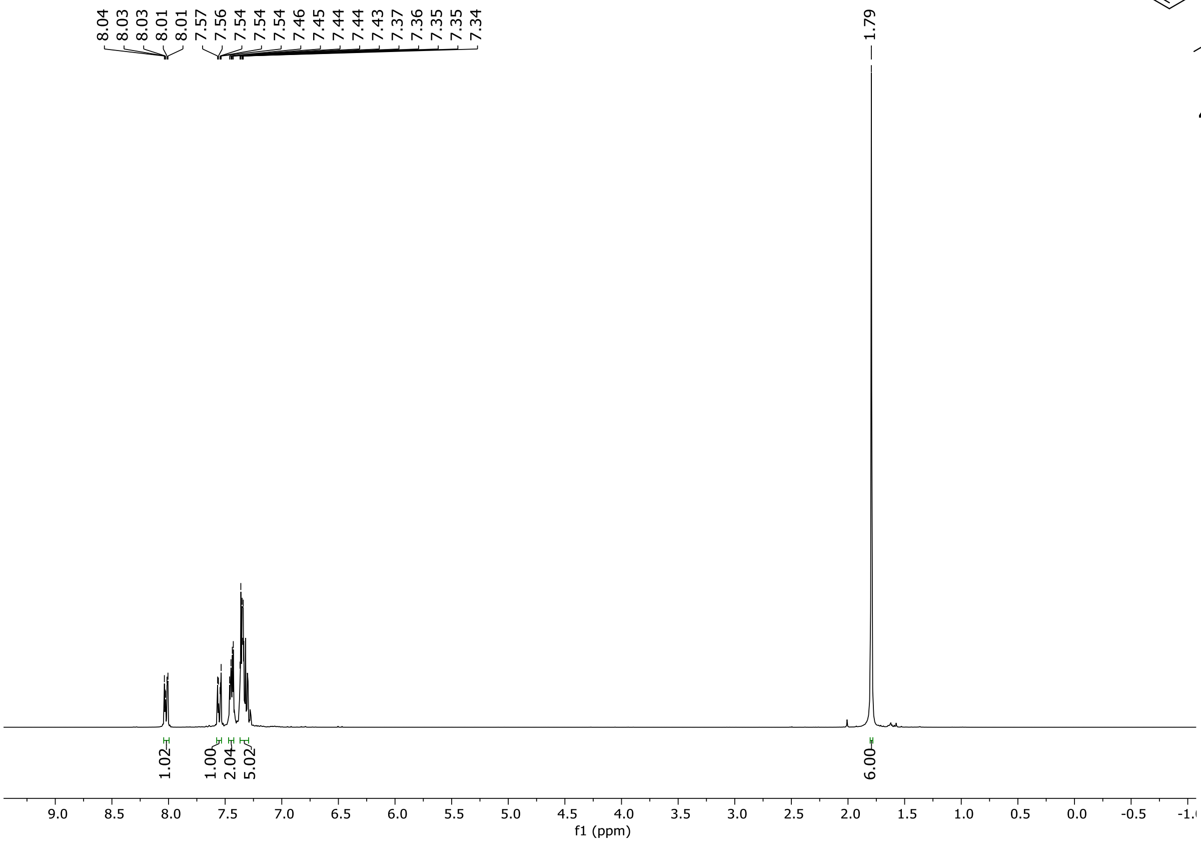

^{13}C{^{1}H} NMR (75.4 MHz, $CDCl_3$)

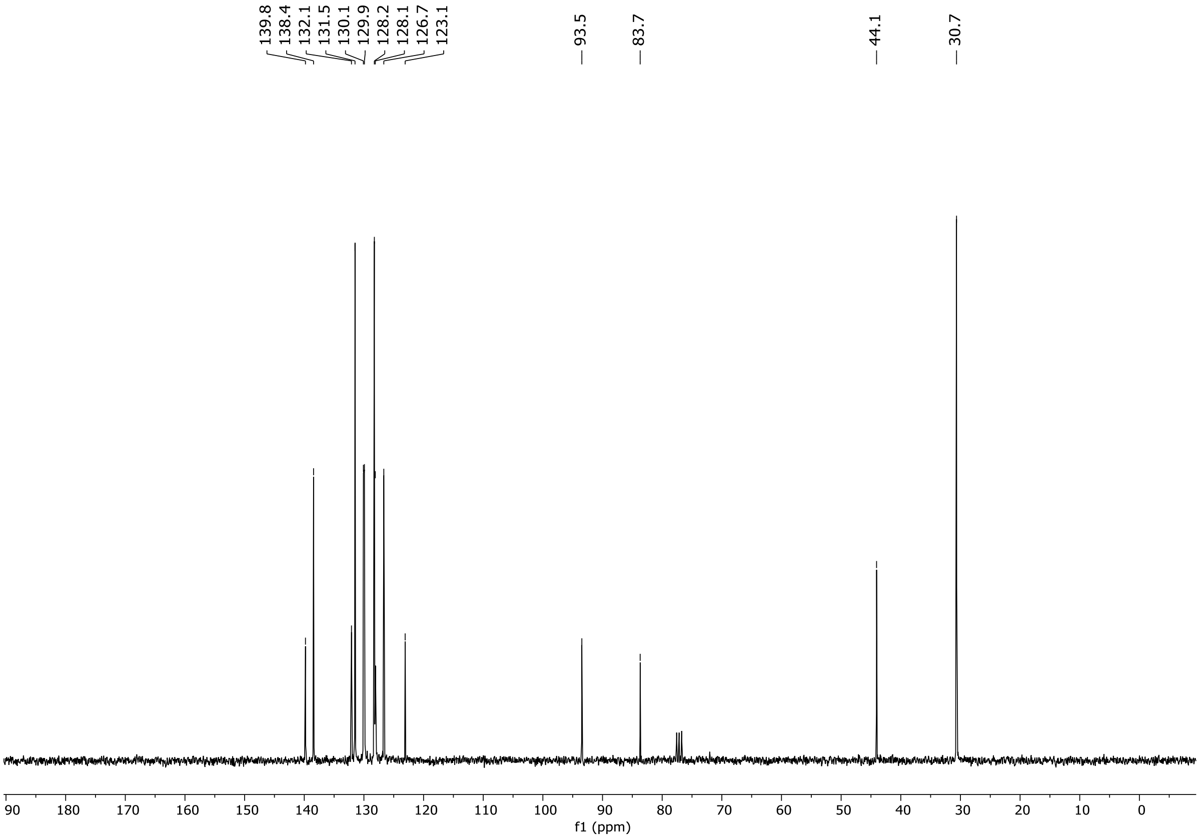

4ah: ^{1}H-NMR (300 MHz, $CDCl_3$)

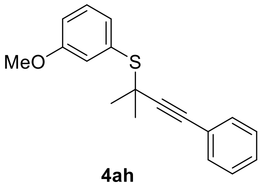

4ah

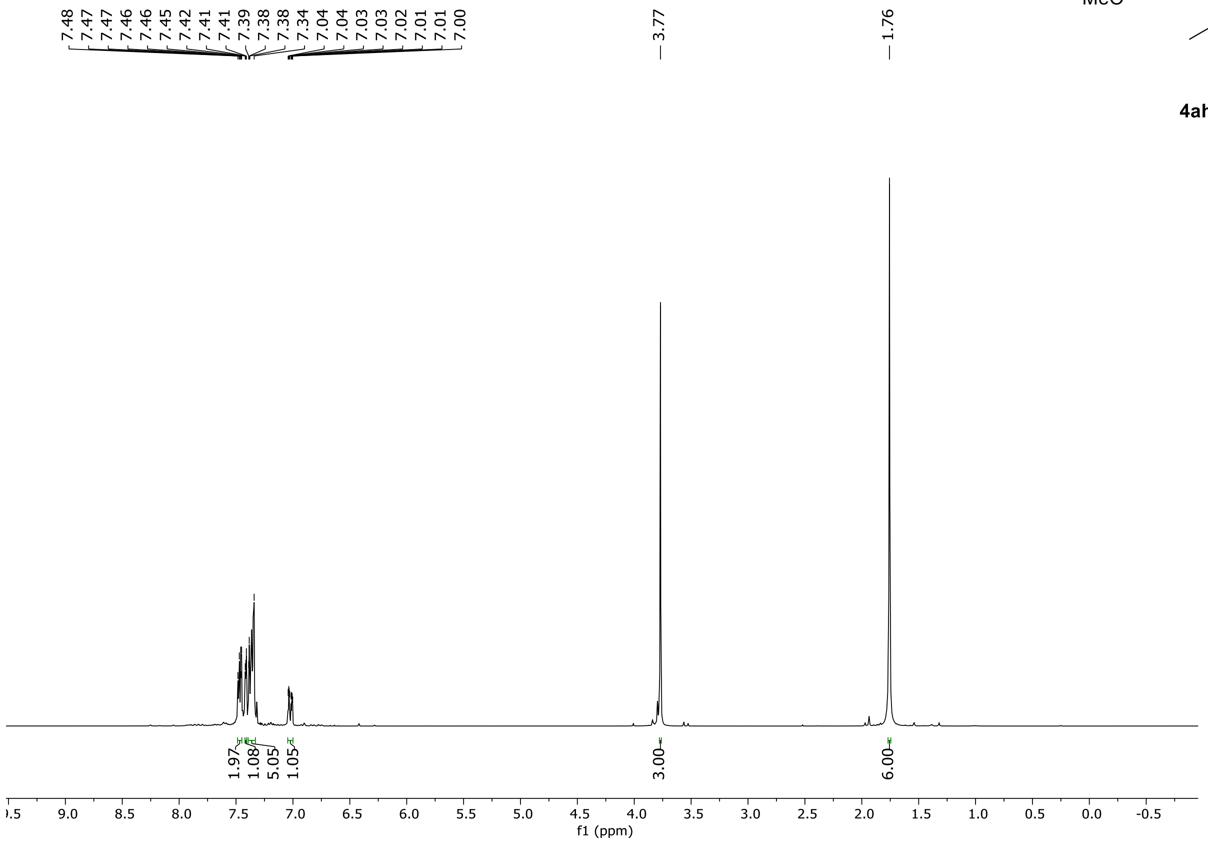

$^{13}C\{^1H\}$ NMR (75.4 MHz, $CDCl_3$)

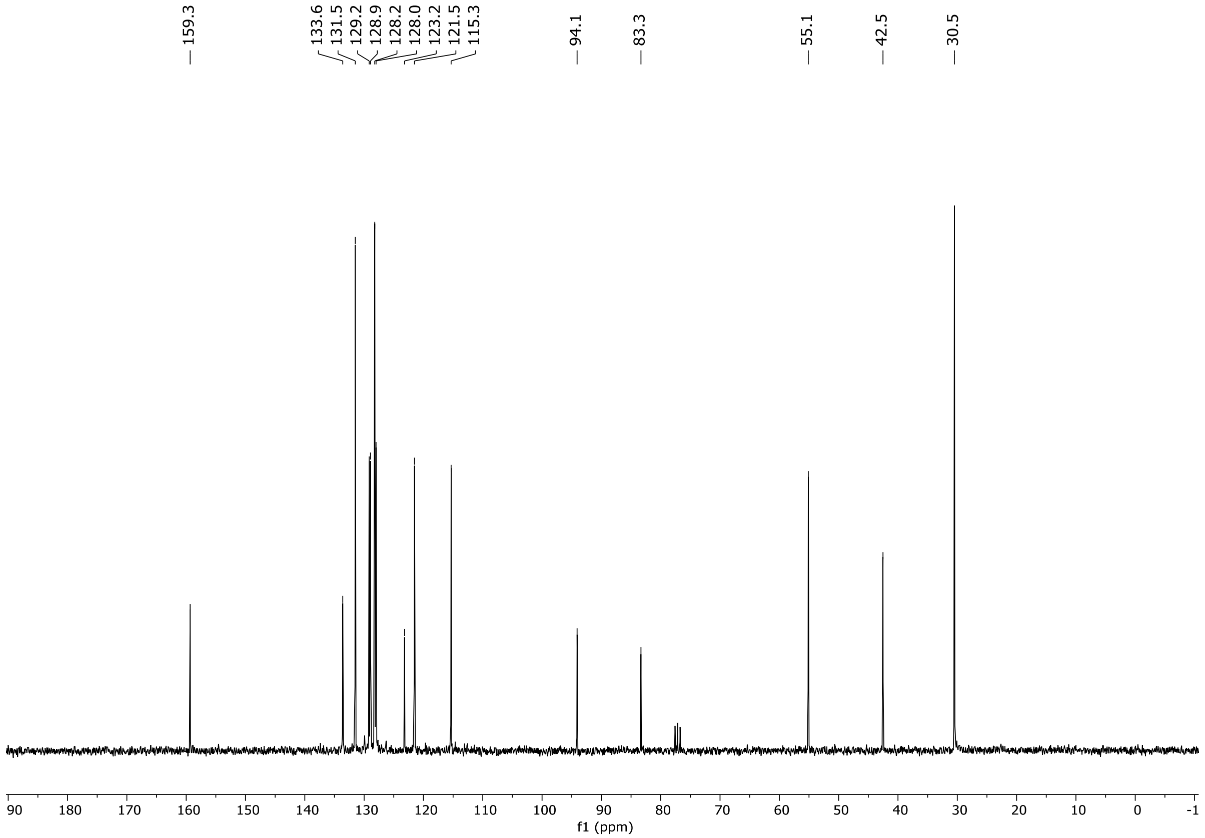

4ai: ^{1}H-NMR (300 MHz, $CDCl_3$)

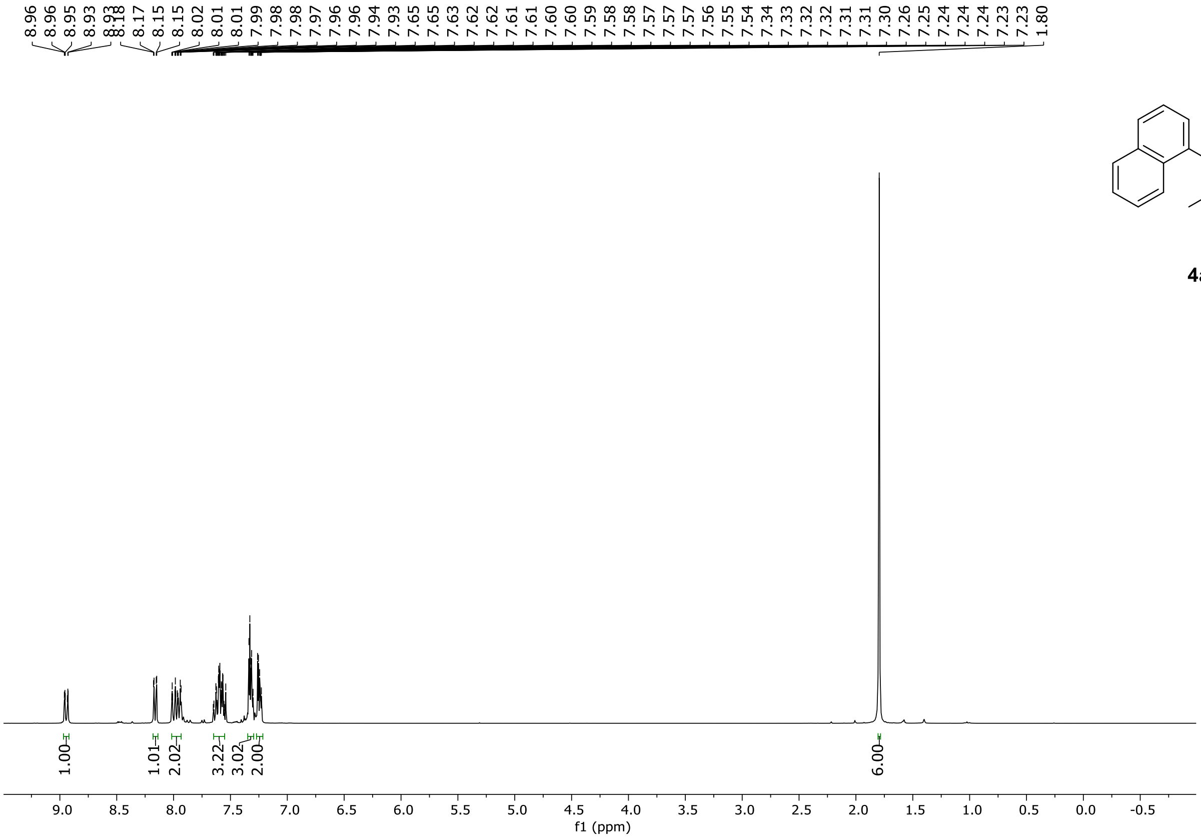

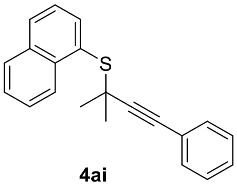

4ai

^{13}C{^{1}H} NMR (75.4 MHz, $CDCl_3$)

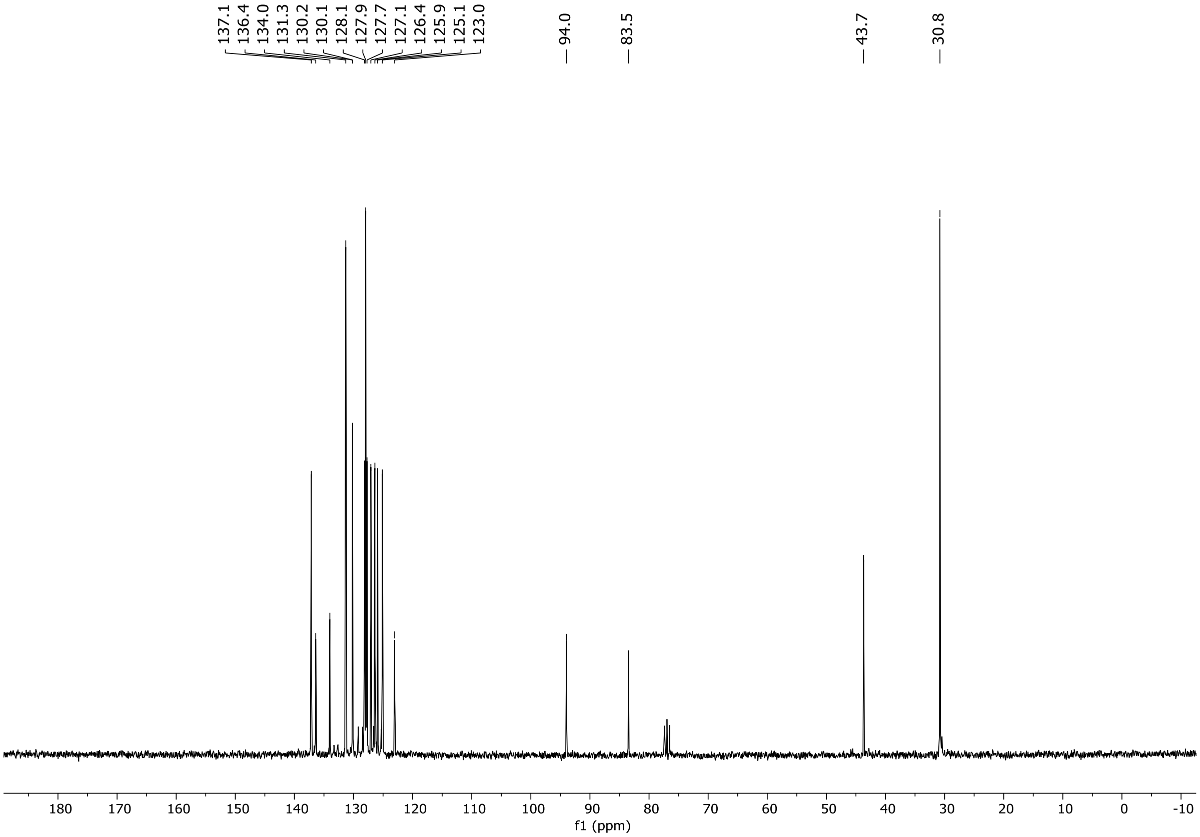

4aj: ^{1}H-NMR (300 MHz, CDCl$_3$)

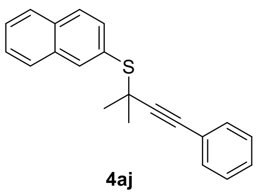

4aj

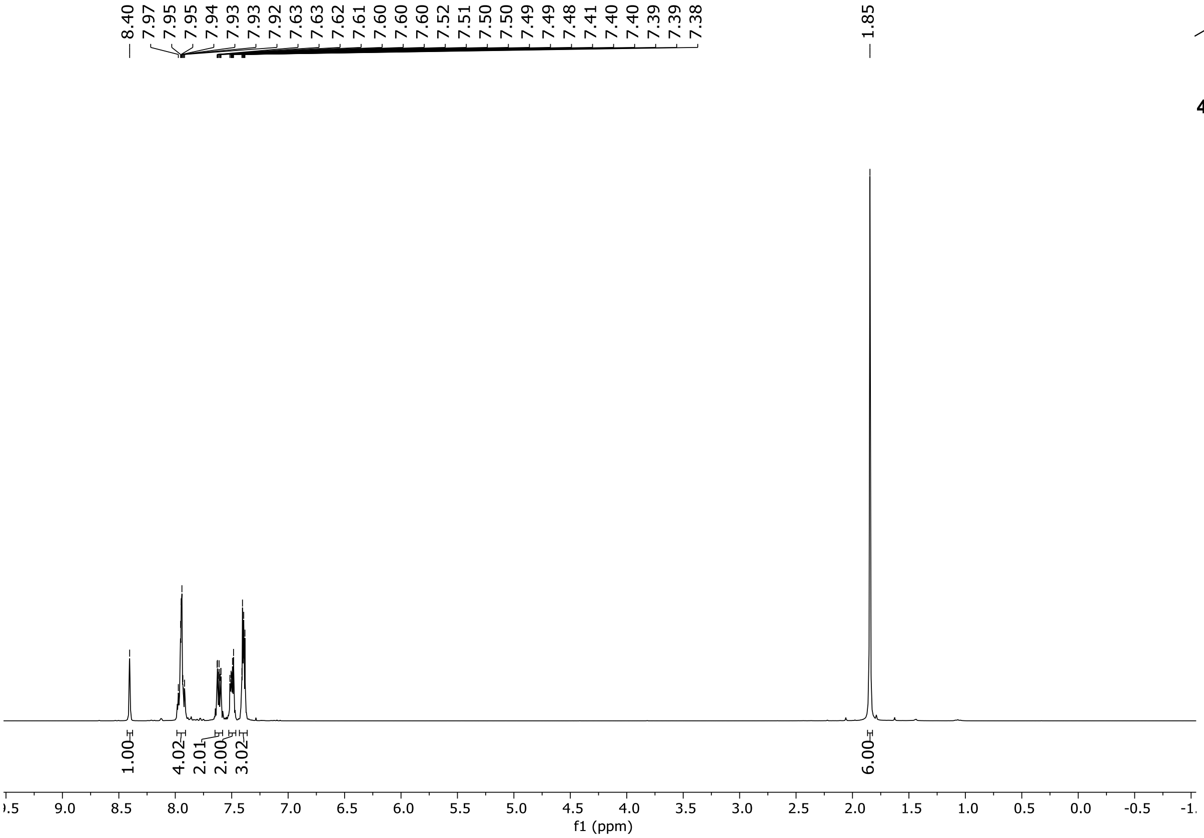

^{13}C{^{1}H} NMR (75.4 MHz, CDCl$_3$)

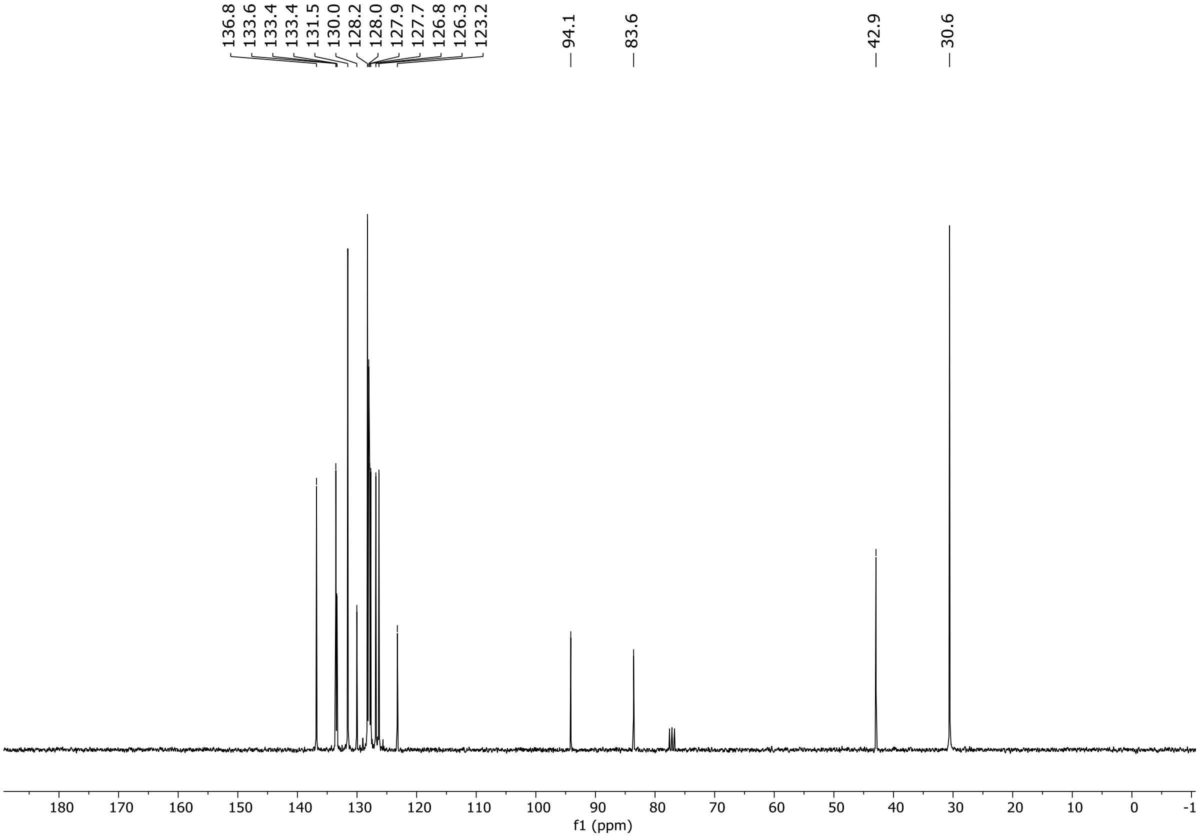

4ba: ^{1}H-NMR (300 MHz, $CDCl_3$)

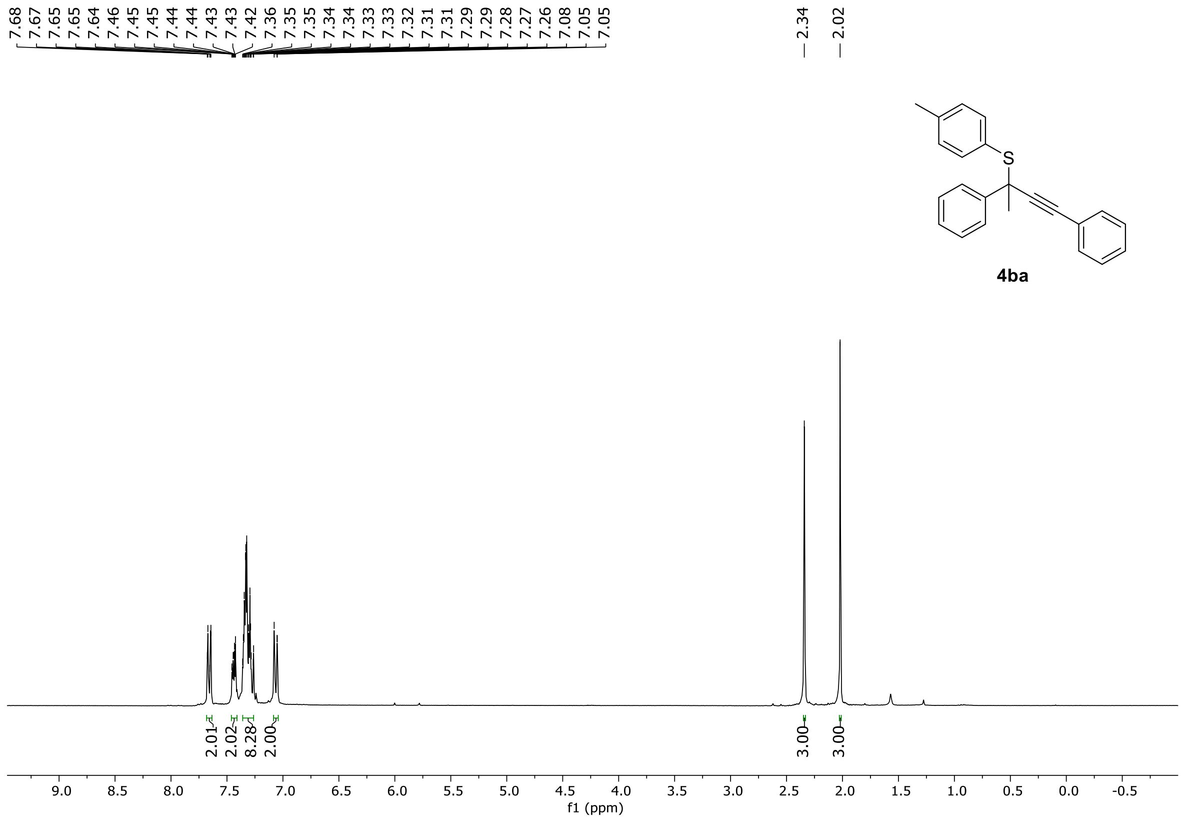

^{13}C{^{1}H} NMR (75.4 MHz, $CDCl_3$)

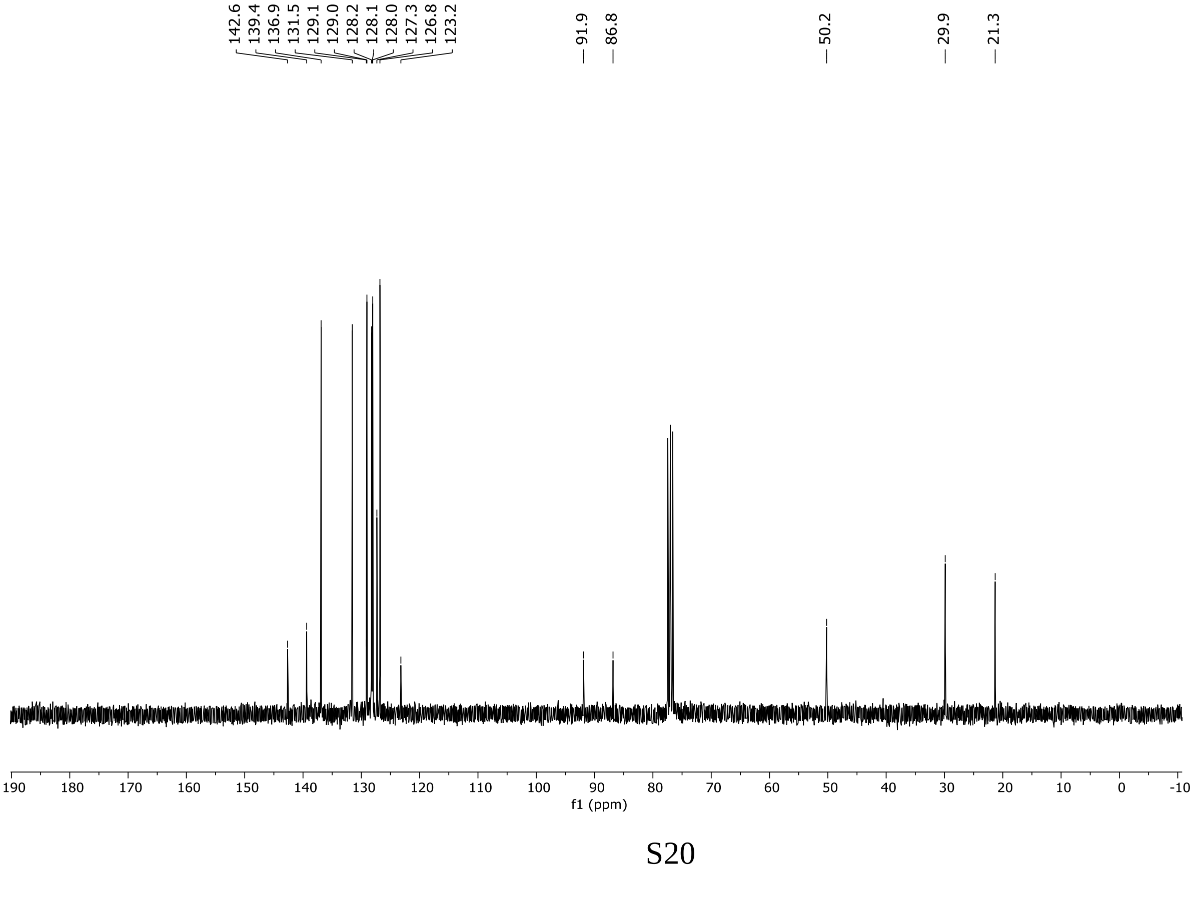

4ca: ^{1}H-NMR (300 MHz, $CDCl_3$)

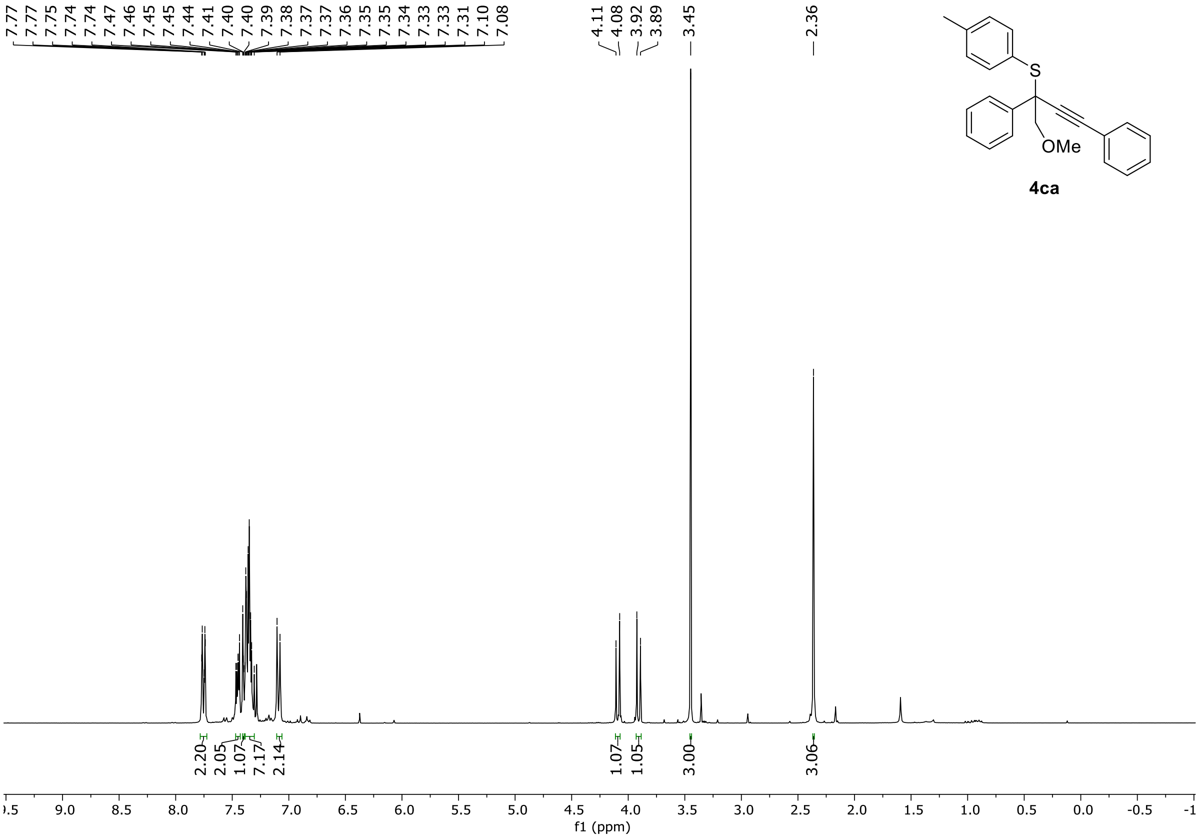

^{13}C{^{1}H} NMR (75.4 MHz, $CDCl_3$)

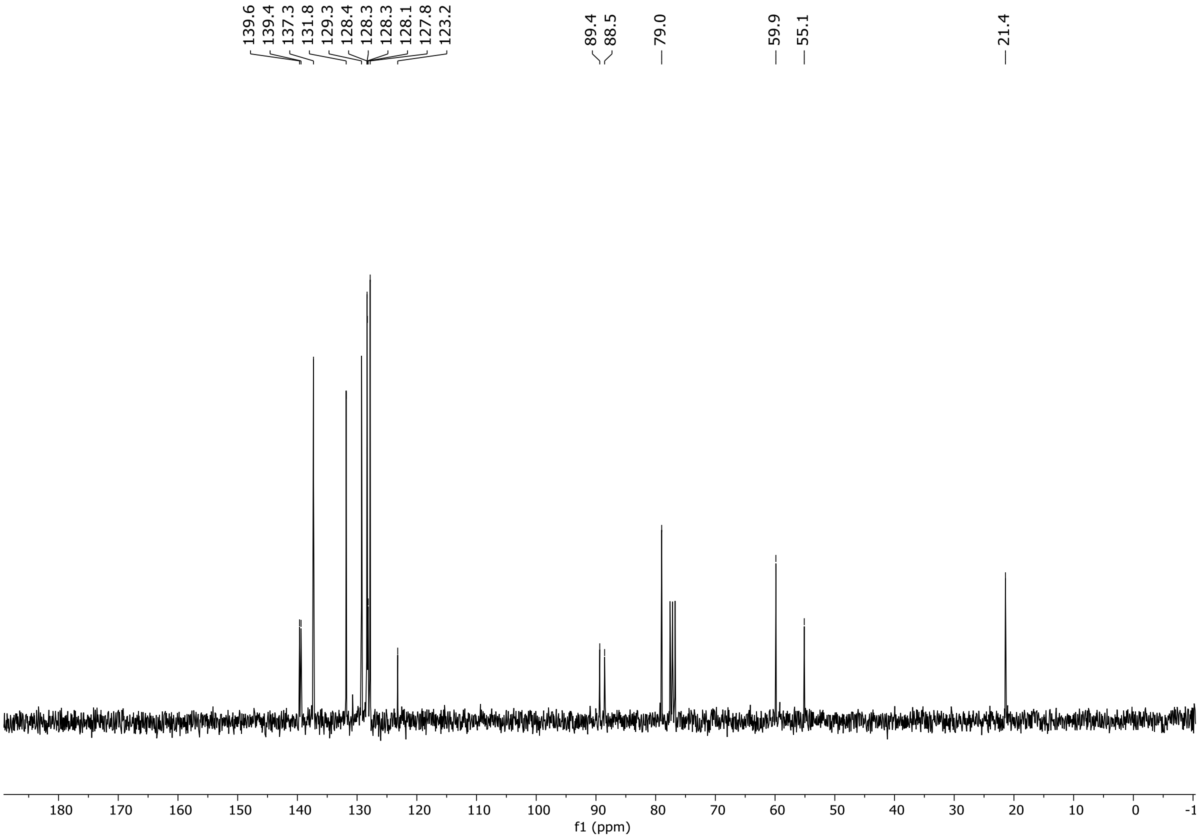

4da: ^{1}H-NMR (300 MHz, $CDCl_3$)

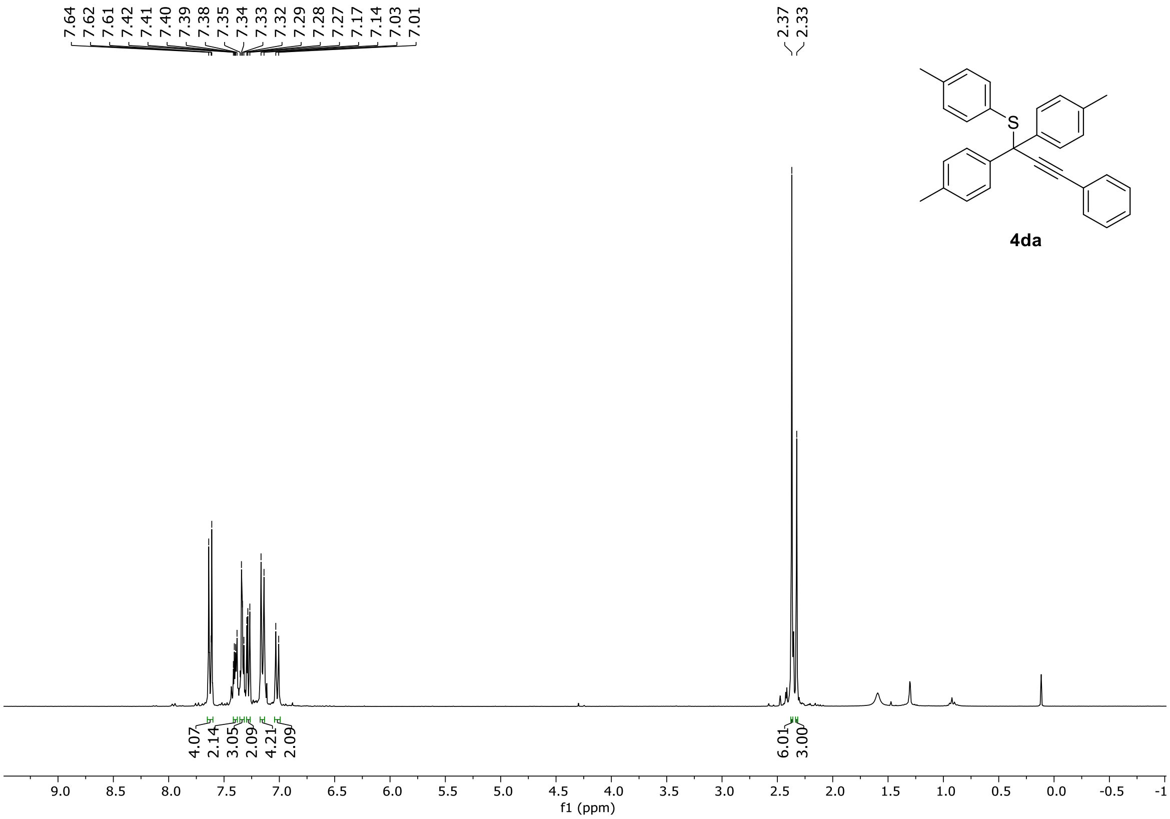

^{13}C{^{1}H} NMR (75.4 MHz, $CDCl_3$)

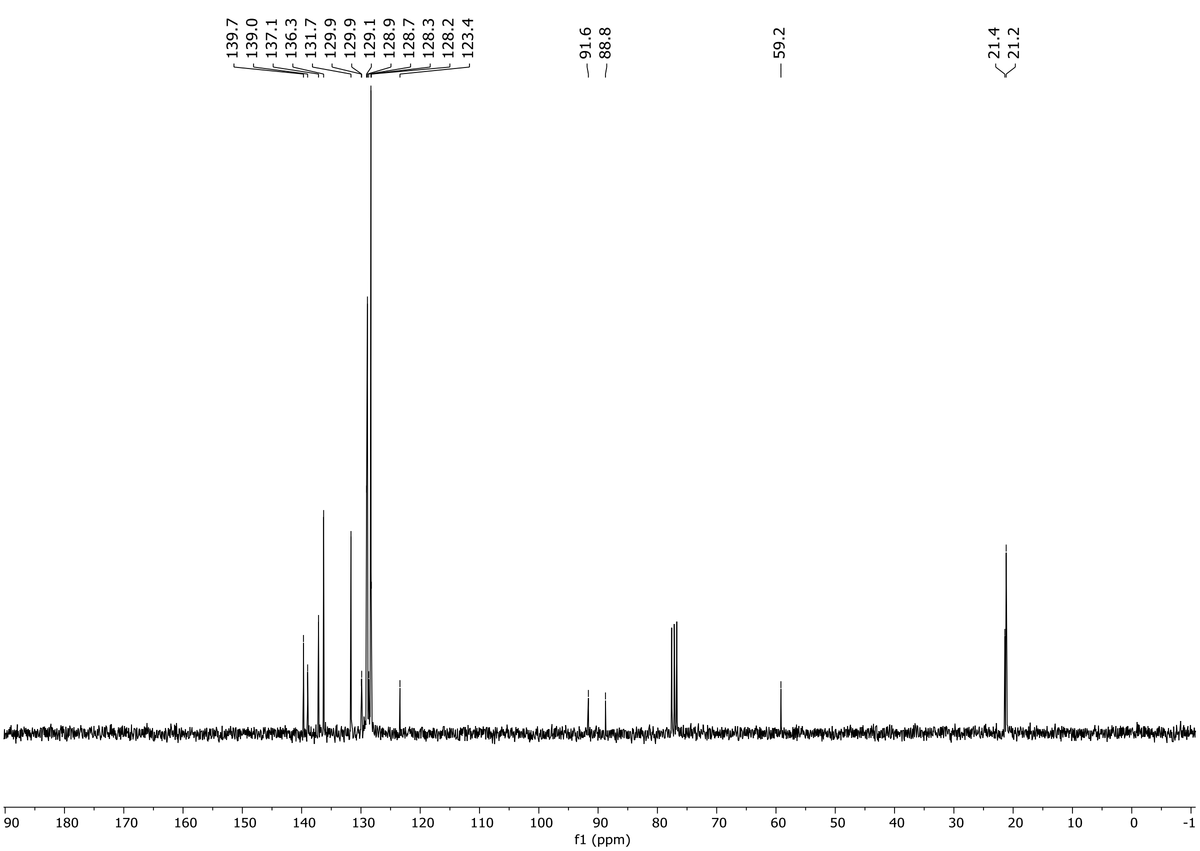

4ea: ^{1}H-NMR (300 MHz, $CDCl_3$)

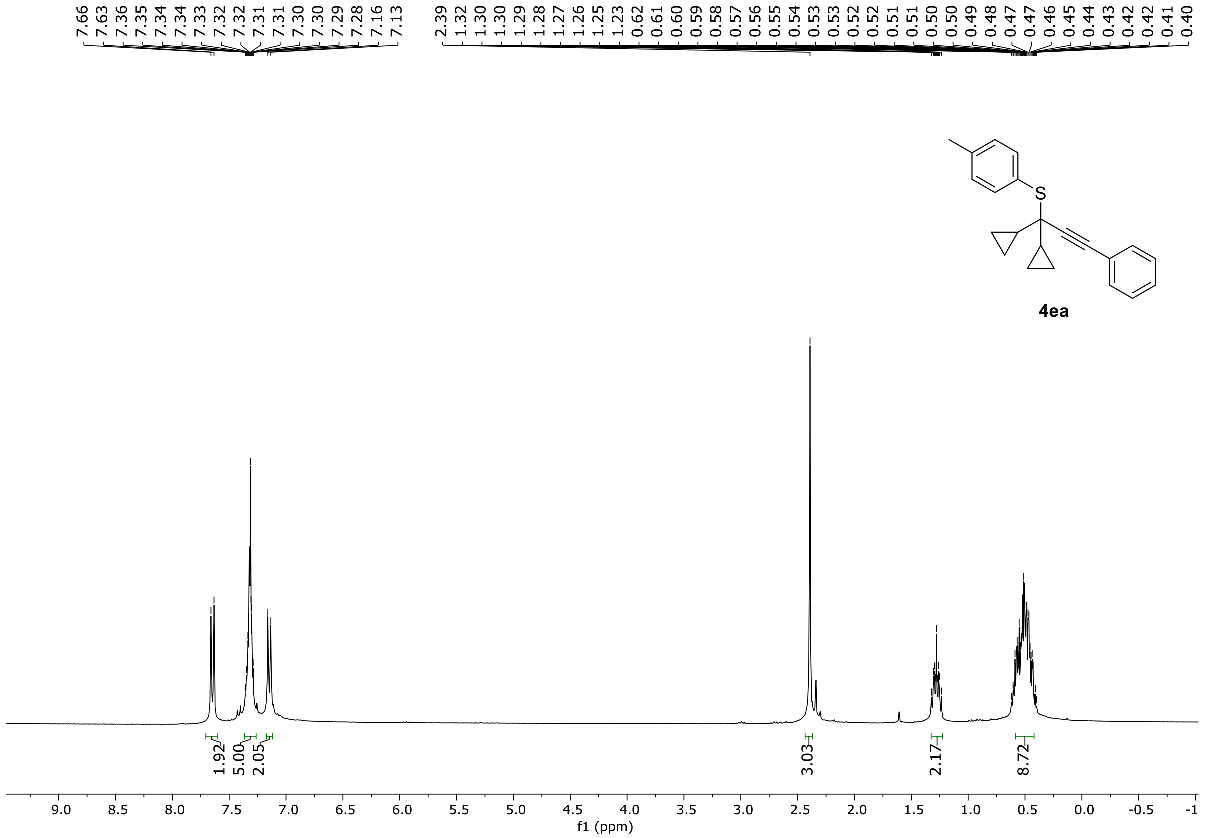

^{13}C{^{1}H} NMR (75.4 MHz, $CDCl_3$)

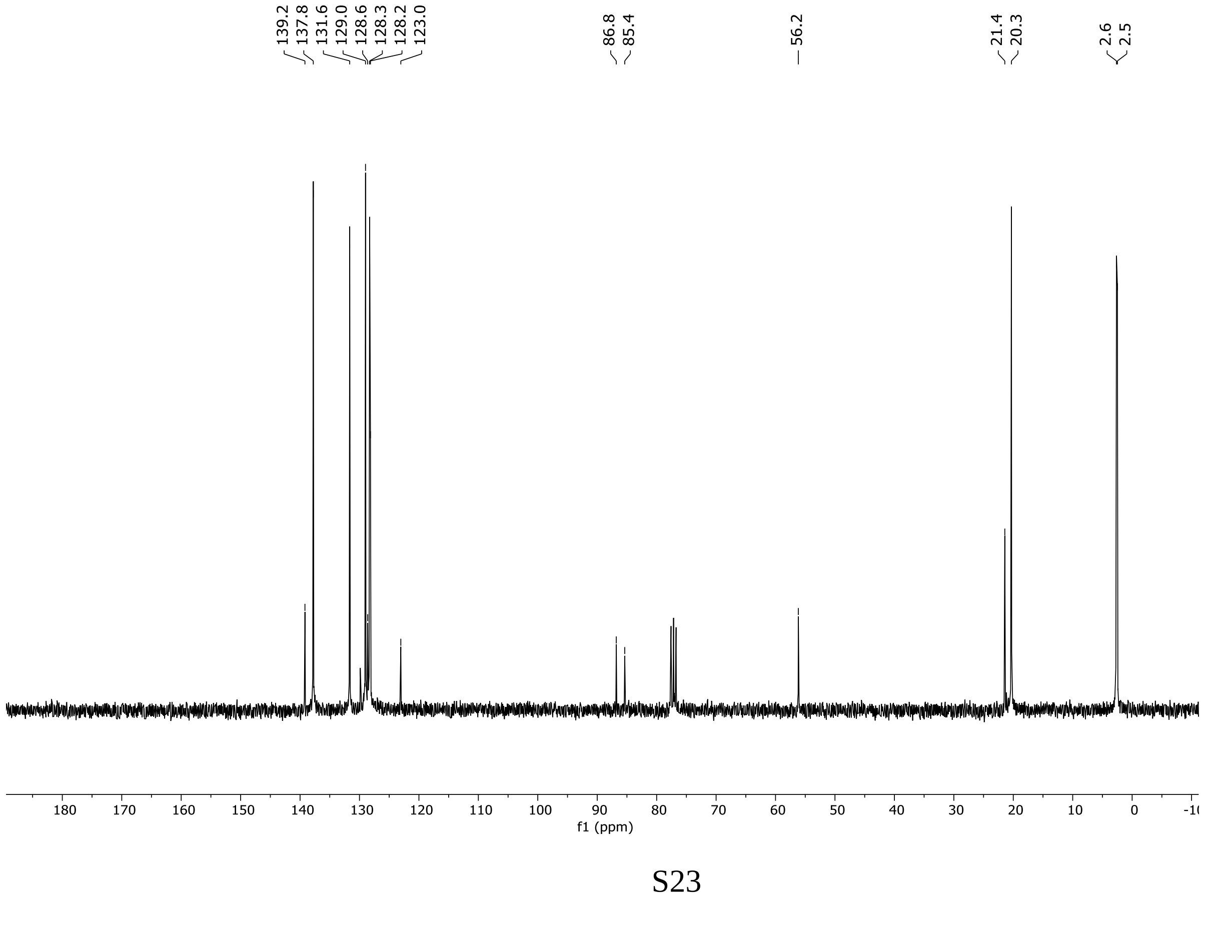

4fa: ^{1}H-NMR (300 MHz, $CDCl_3$)

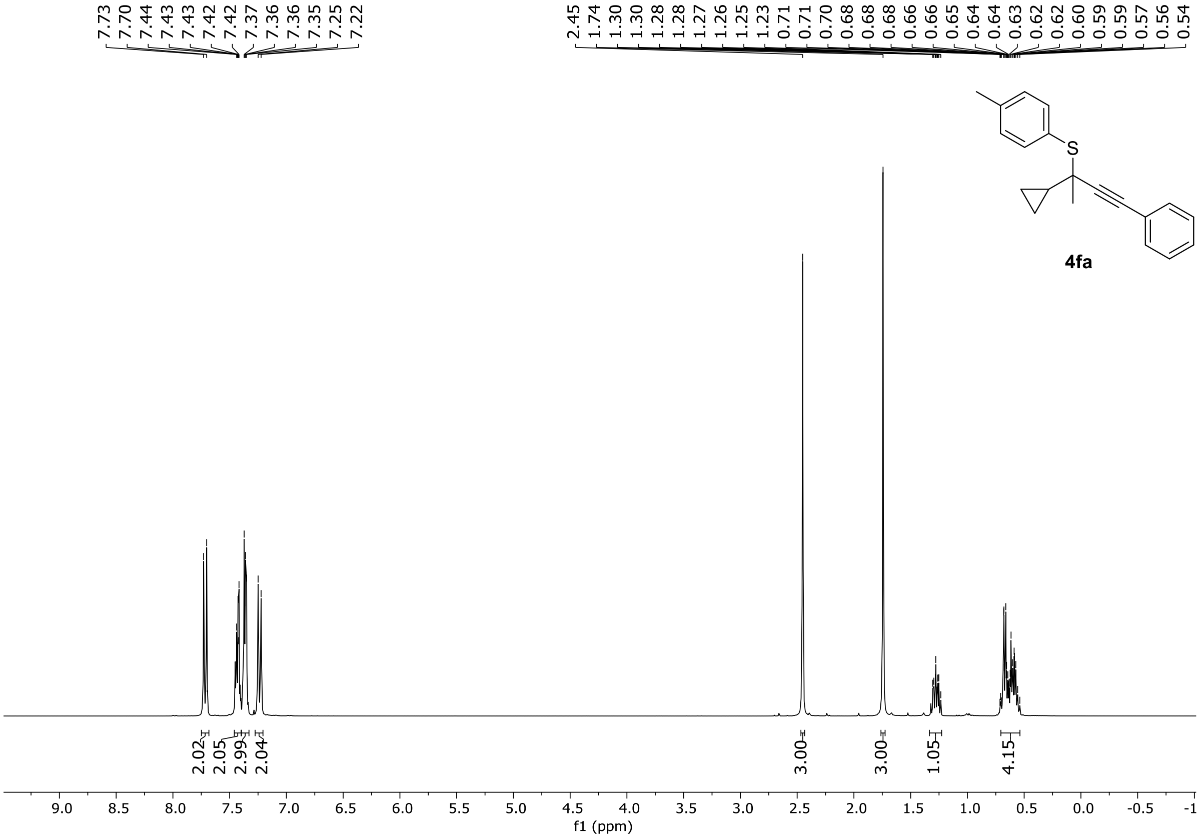

^{13}C{^{1}H} NMR (75.4 MHz, $CDCl_3$)

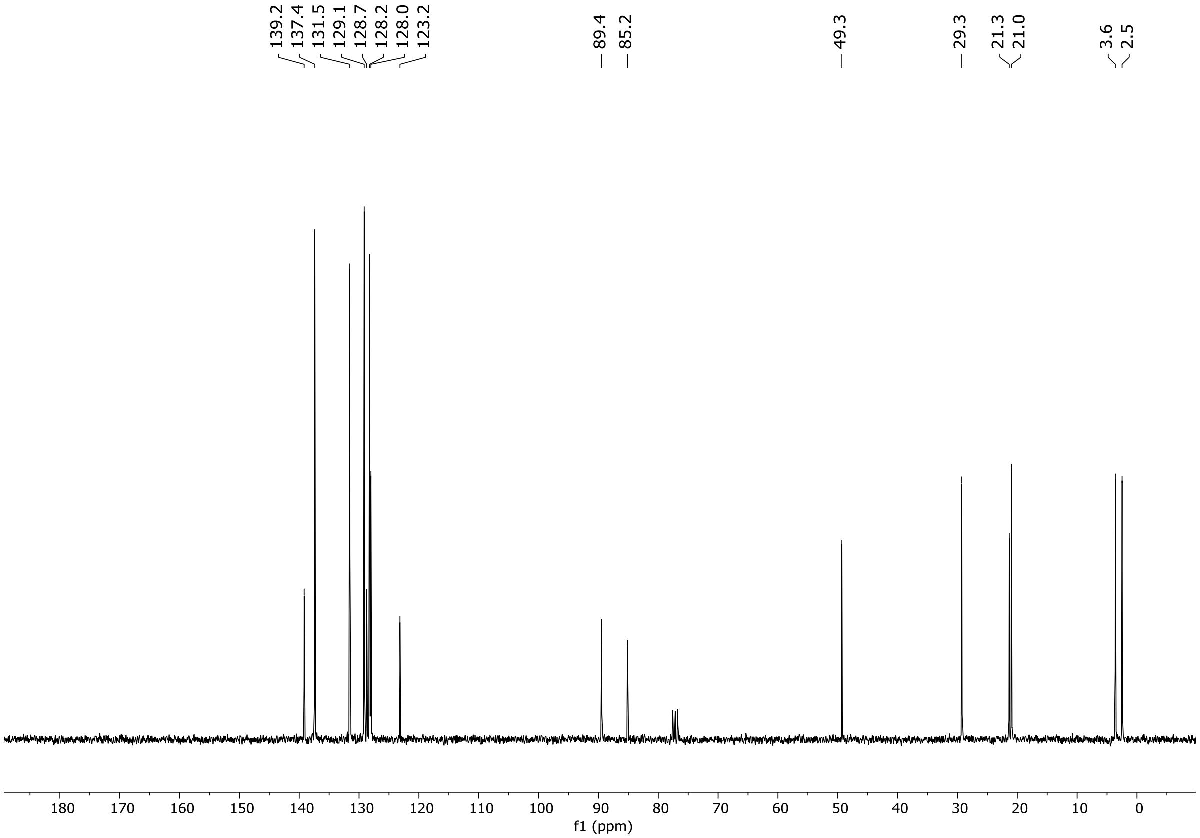

4ga: ^{1}H-NMR (300 MHz, $CDCl_3$)

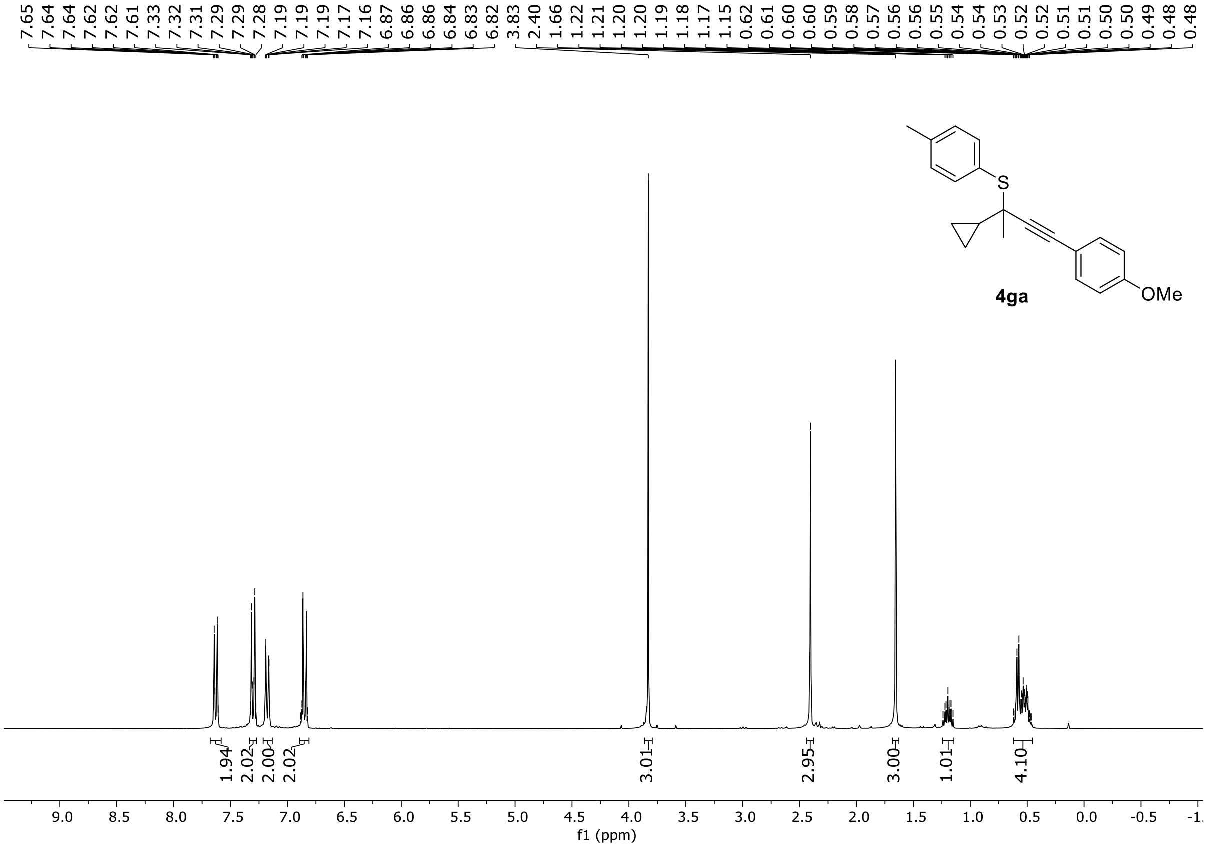

^{13}C{^{1}H} NMR (75.4 MHz, $CDCl_3$)

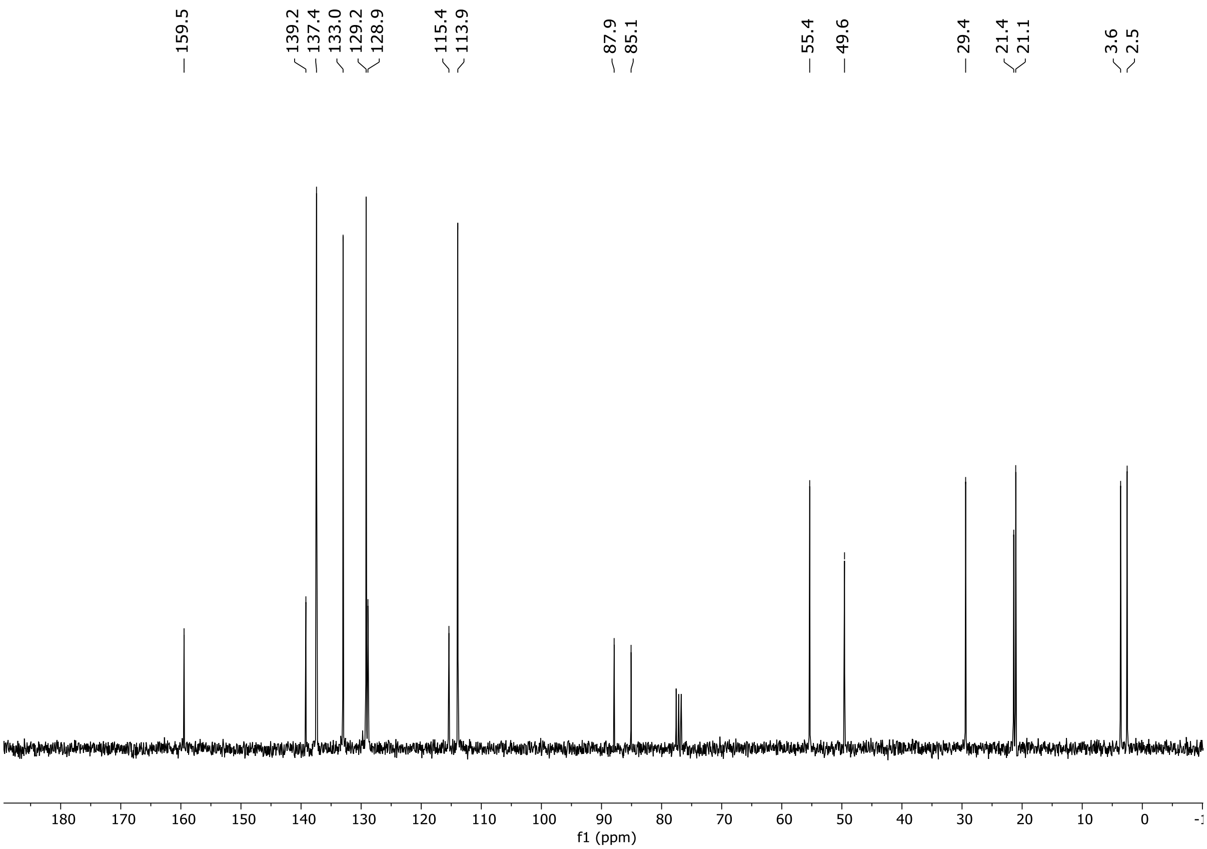

4ha: ^{1}H-NMR (300 MHz, $CDCl_3$)

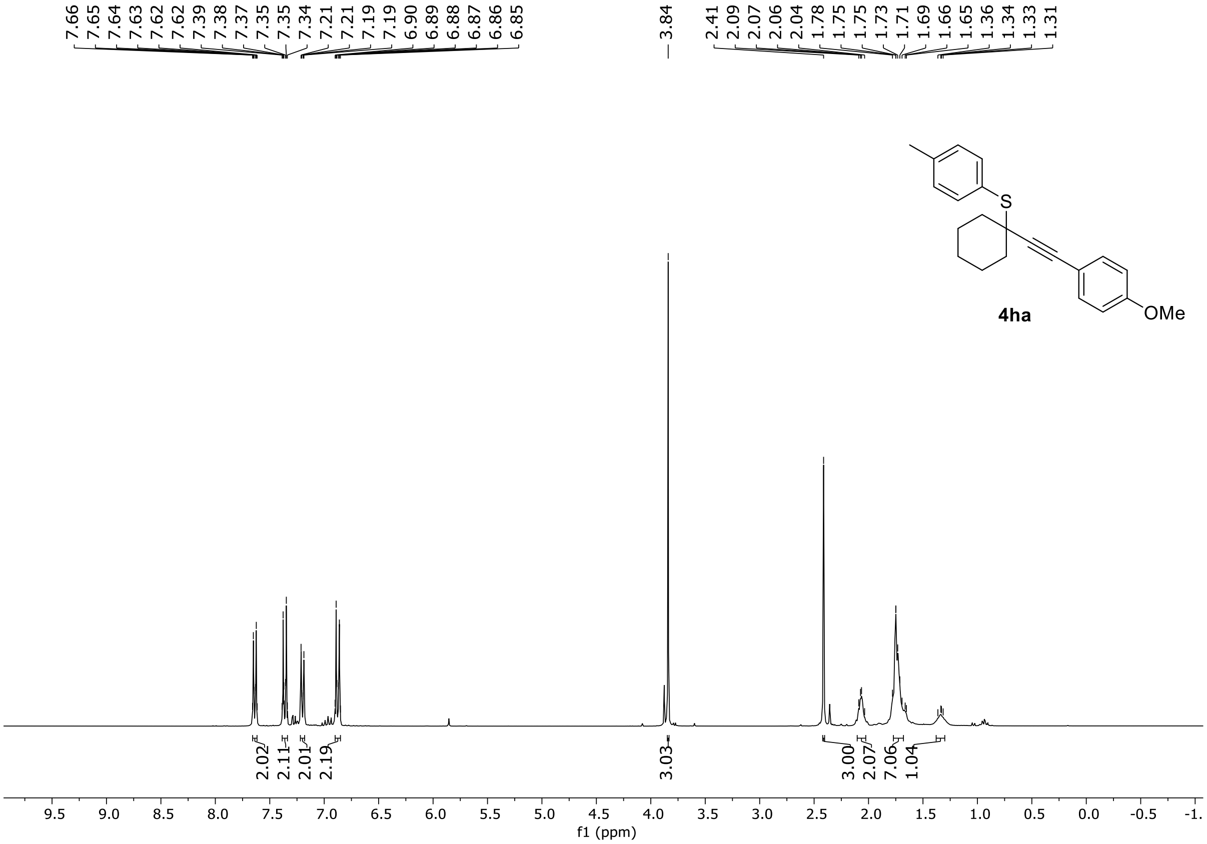

^{13}C{^{1}H} NMR (75.4 MHz, $CDCl_3$)

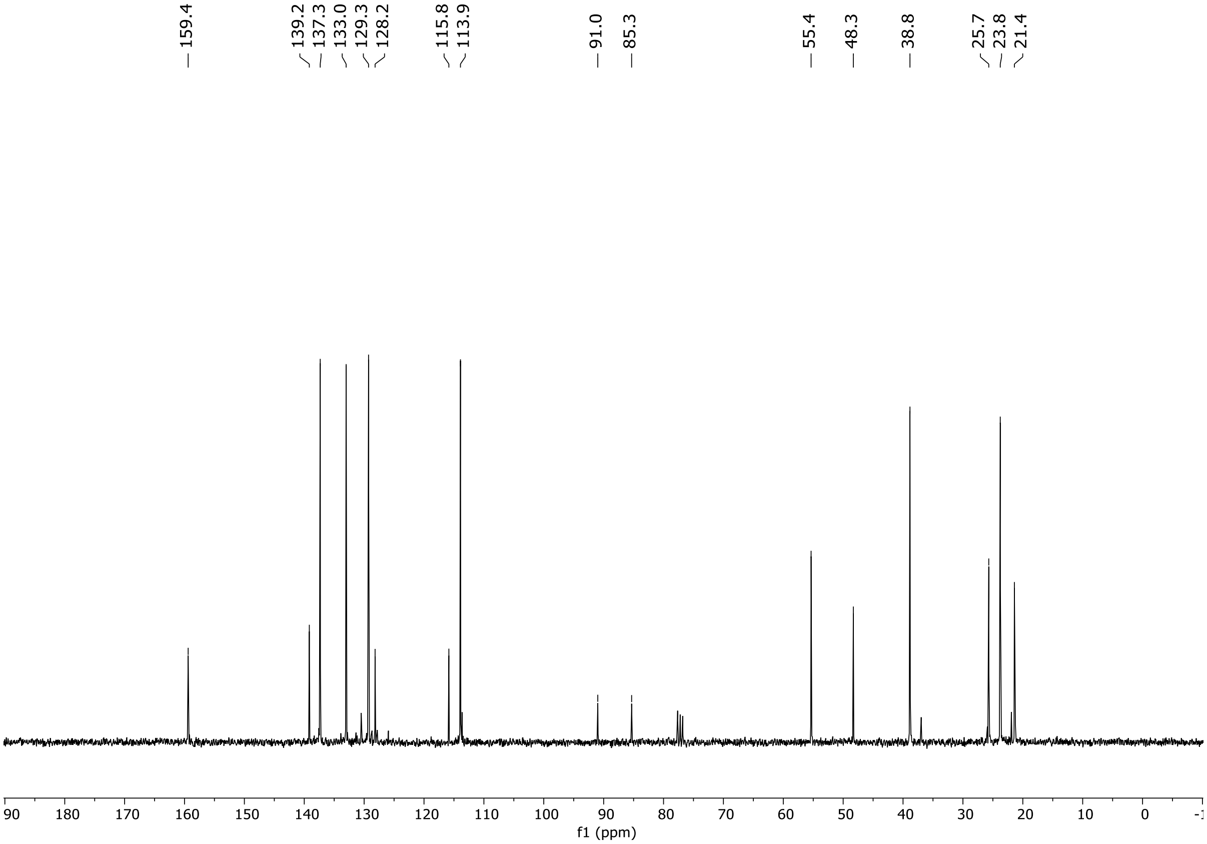

4ia: ^{1}H-NMR (300 MHz, $CDCl_3$)

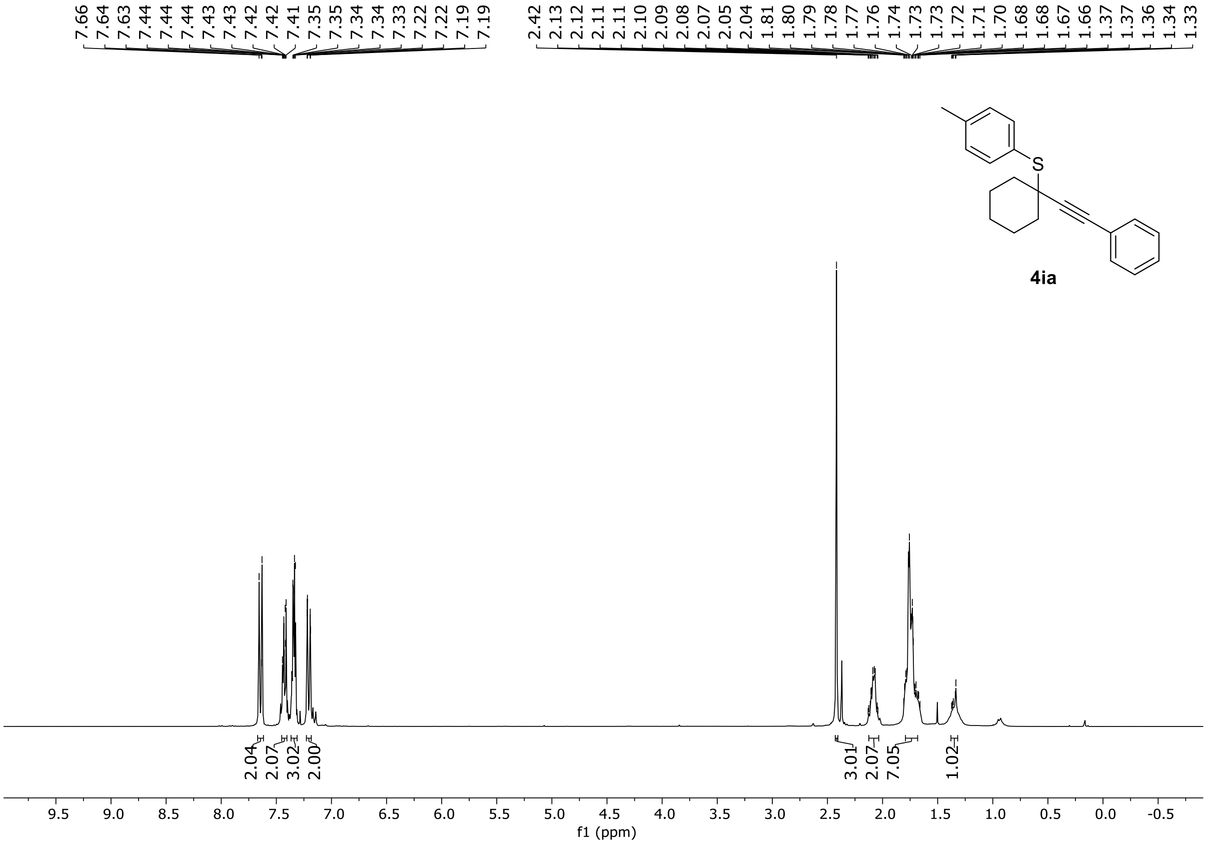

^{13}C{^{1}H} NMR (75.4 MHz, $CDCl_3$)

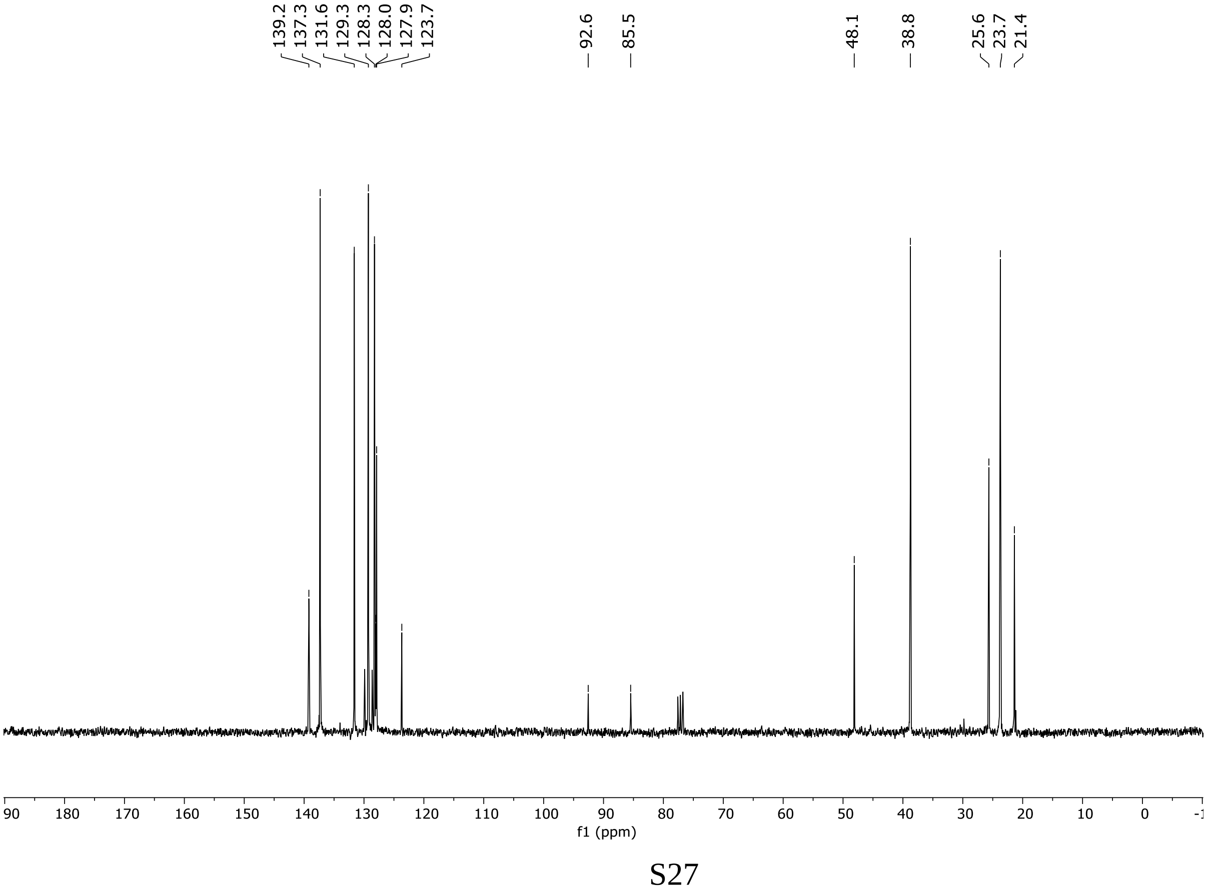

4ja: ^{1}H-NMR (300 MHz, $CDCl_3$)

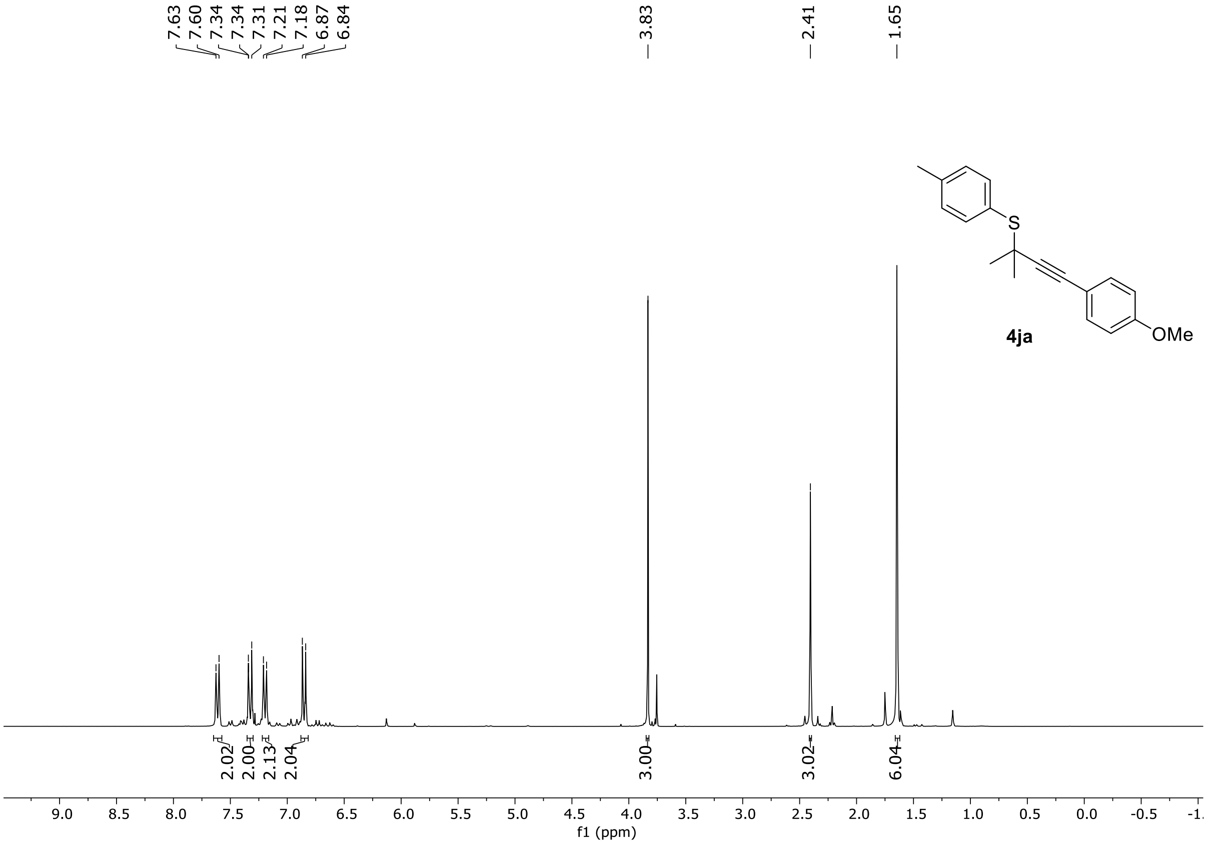

$^{13}C\{^1H\}$ NMR (75.4 MHz, $CDCl_3$)

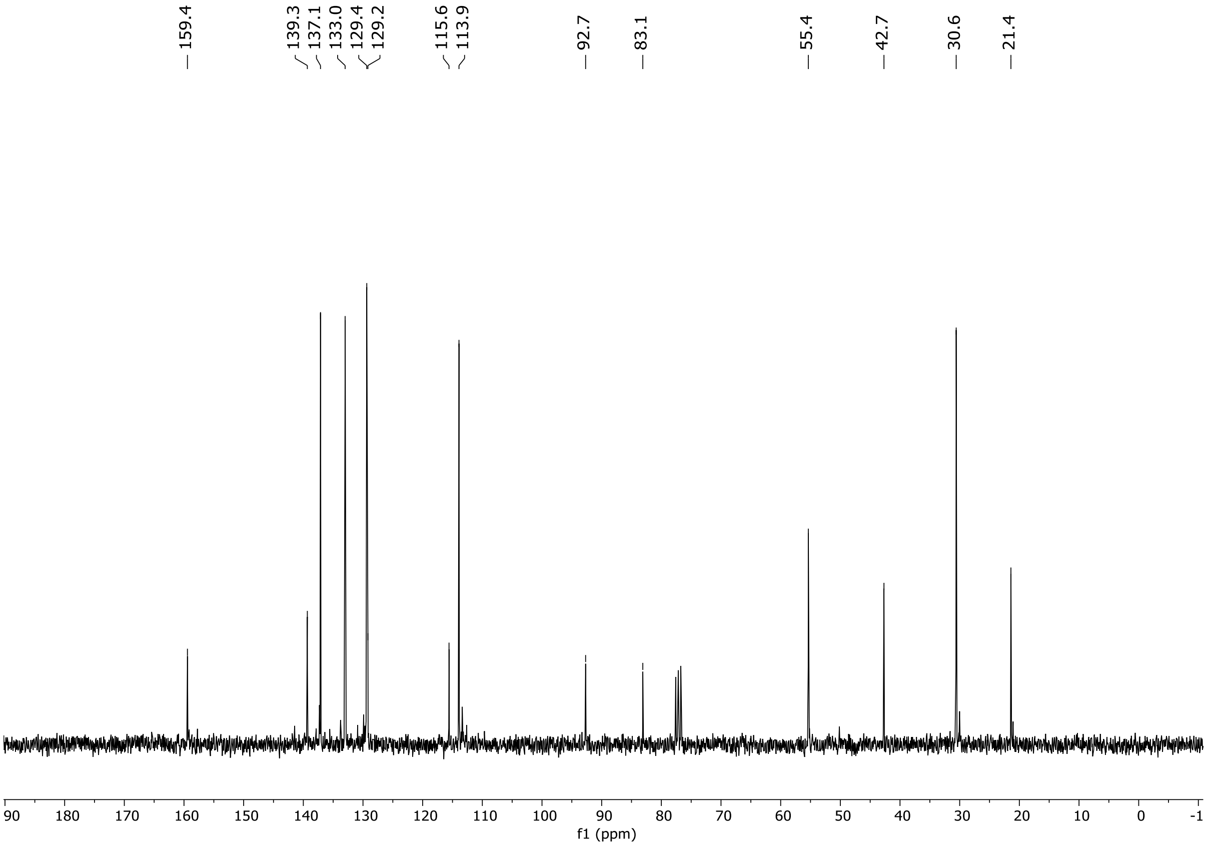

4jc: ^{1}H-NMR (300 MHz, $CDCl_3$)

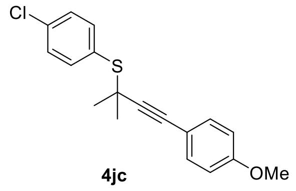

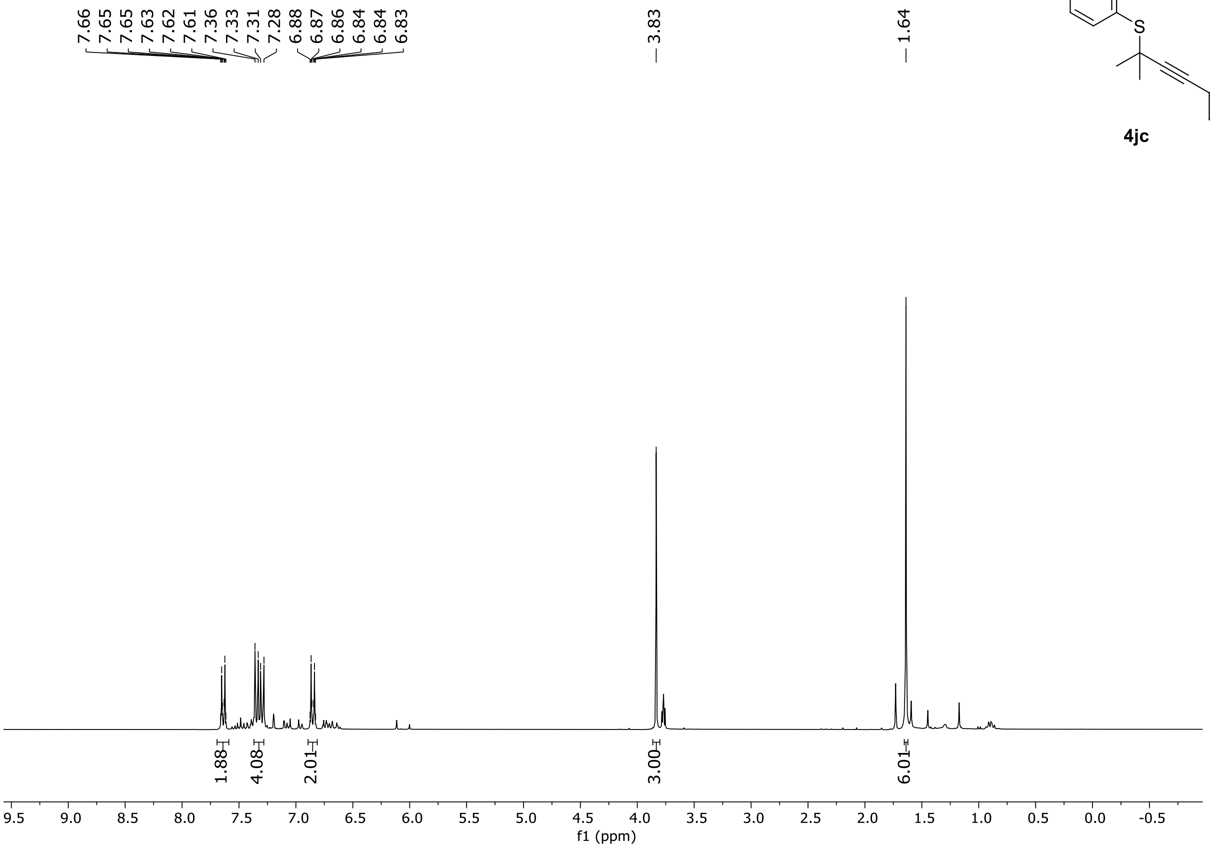

^{13}C{^{1}H} NMR (75.4 MHz, $CDCl_3$)

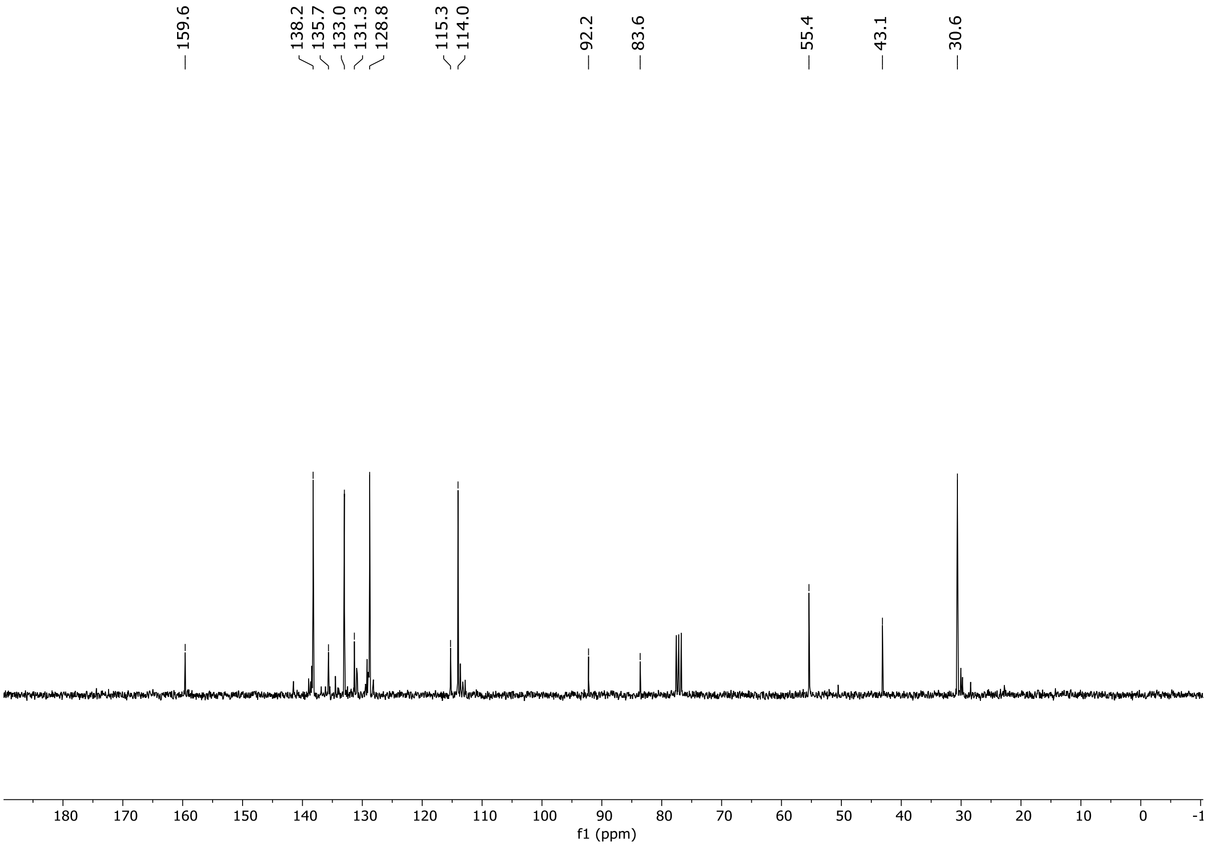

4jk: ^{1}H-NMR (300 MHz, $CDCl_3$)

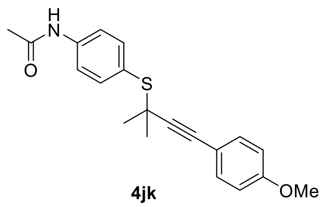

4jk

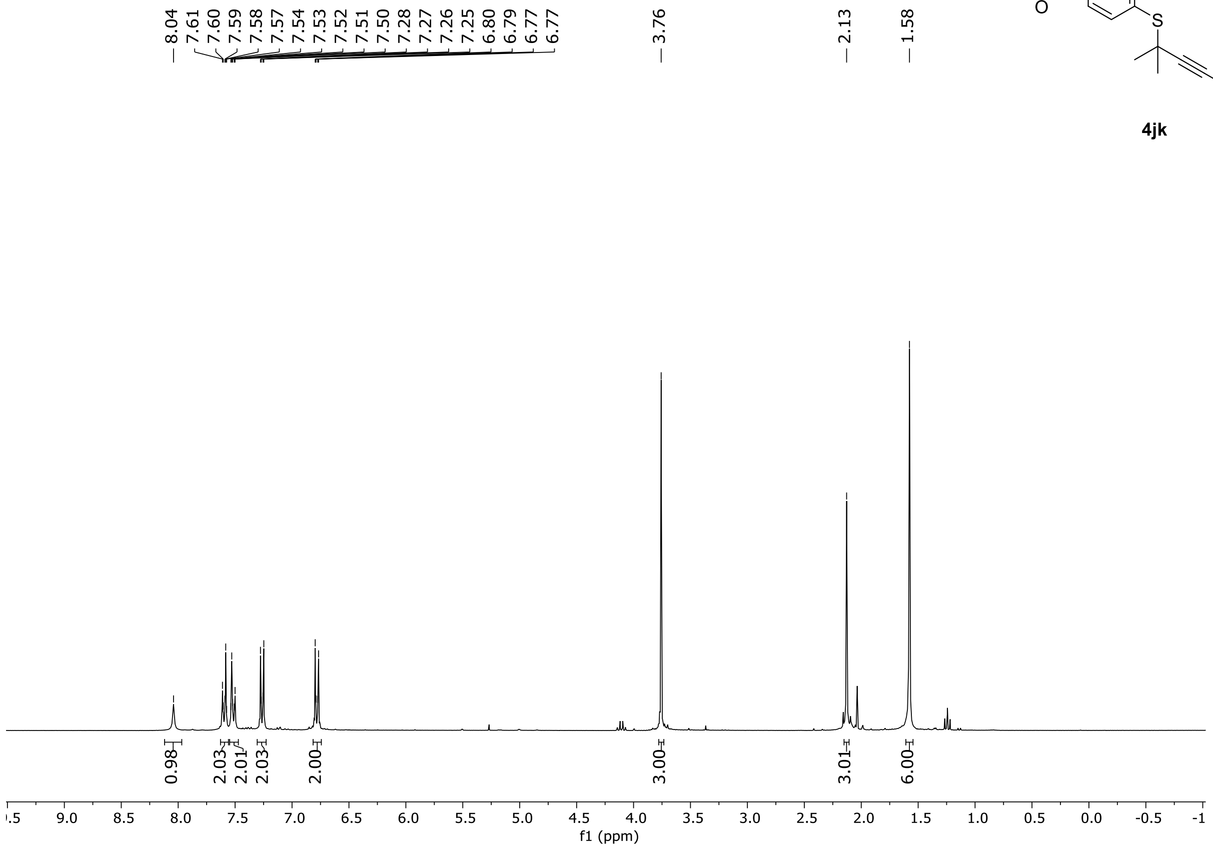

$^{13}C\{^1H\}$ NMR (75.4 MHz, $CDCl_3$)

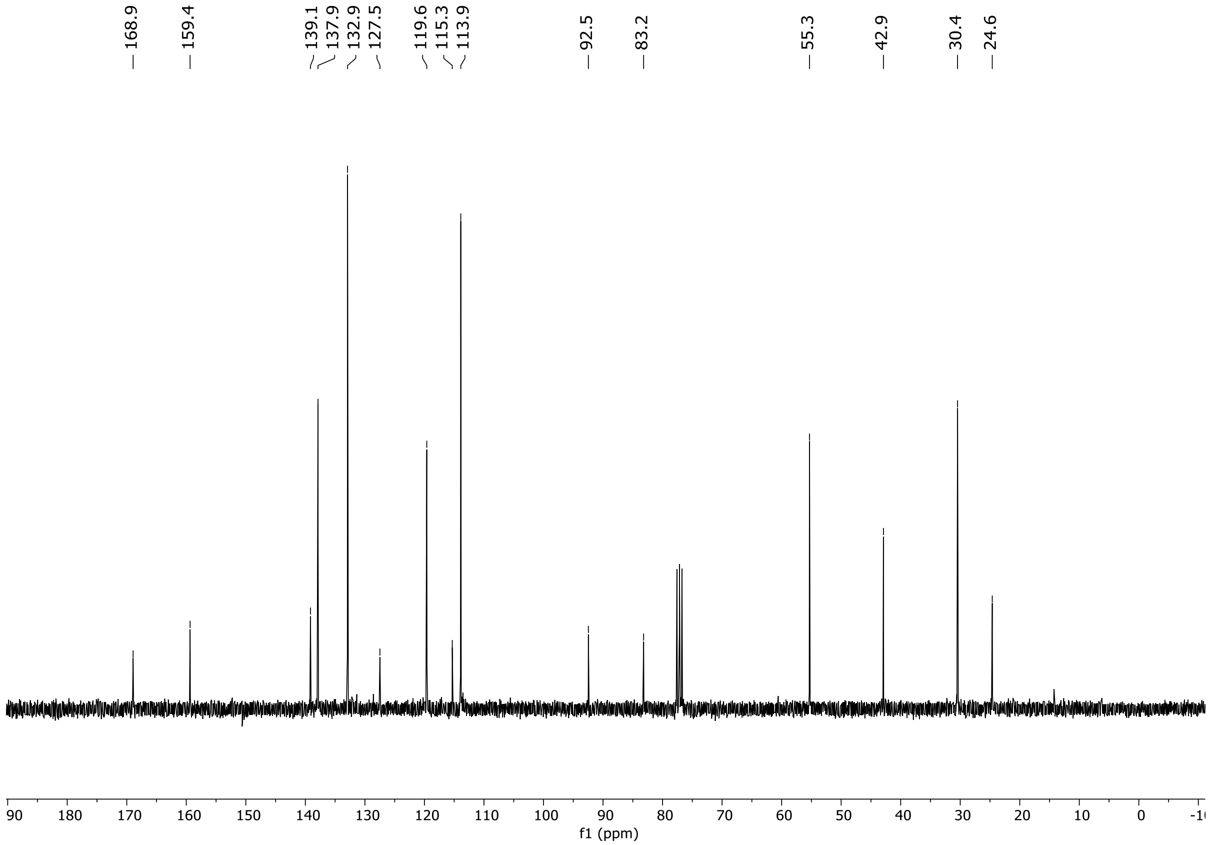

4ka: ^{1}H-NMR (300 MHz, $CDCl_3$)

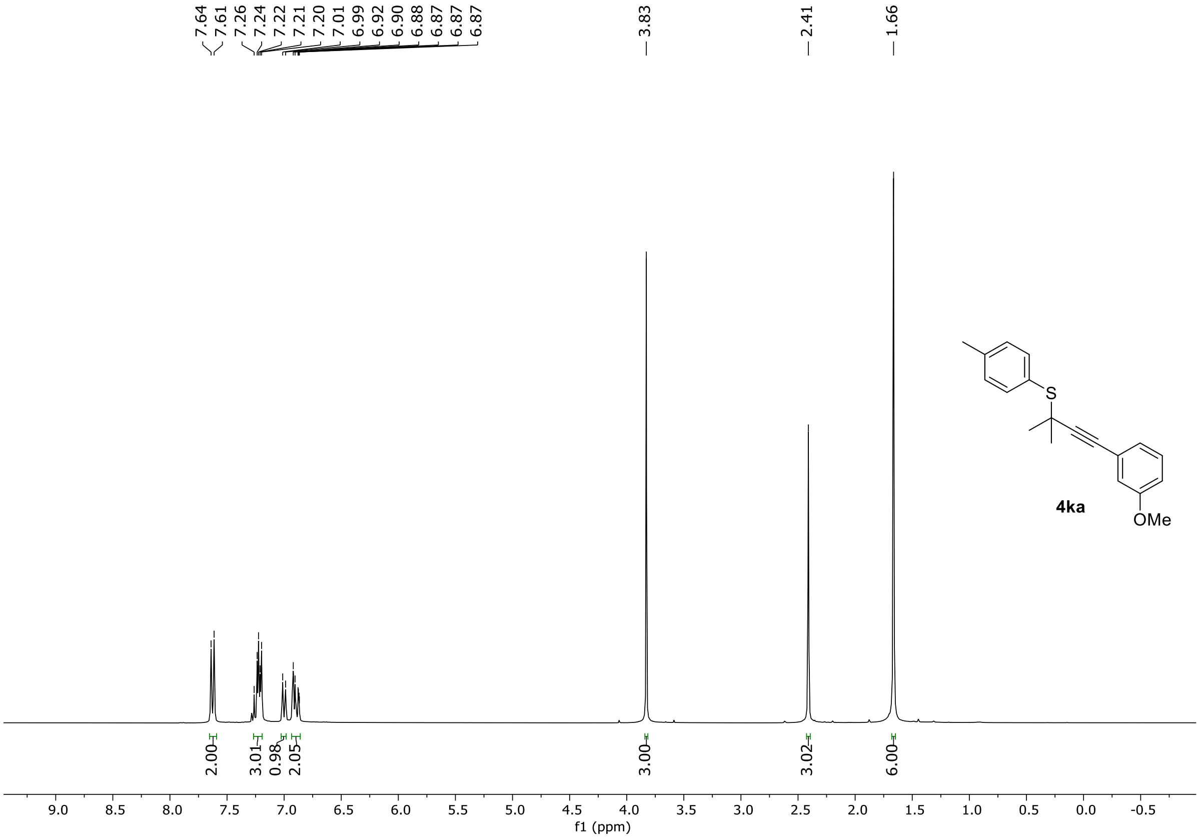

^{13}C{^{1}H} NMR (75.4 MHz, $CDCl_3$)

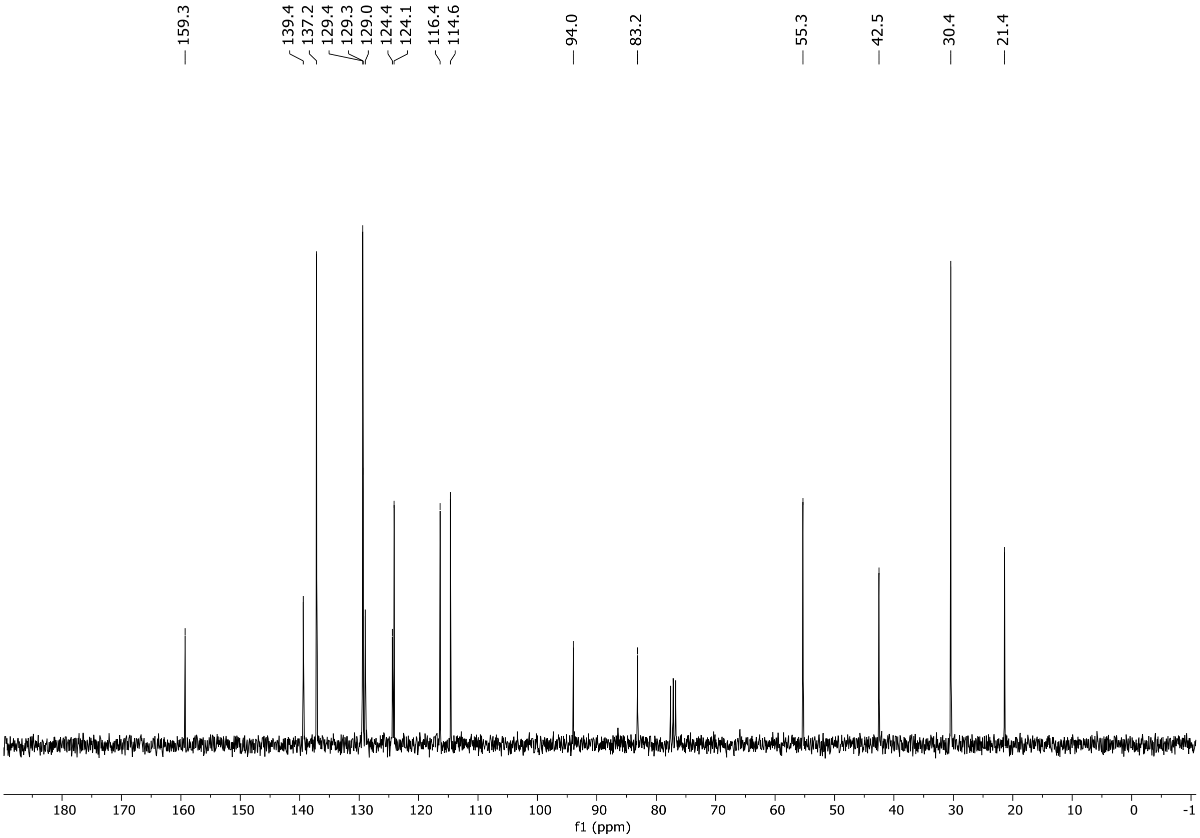

4kd: ^{1}H-NMR (300 MHz, $CDCl_3$)

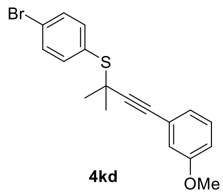

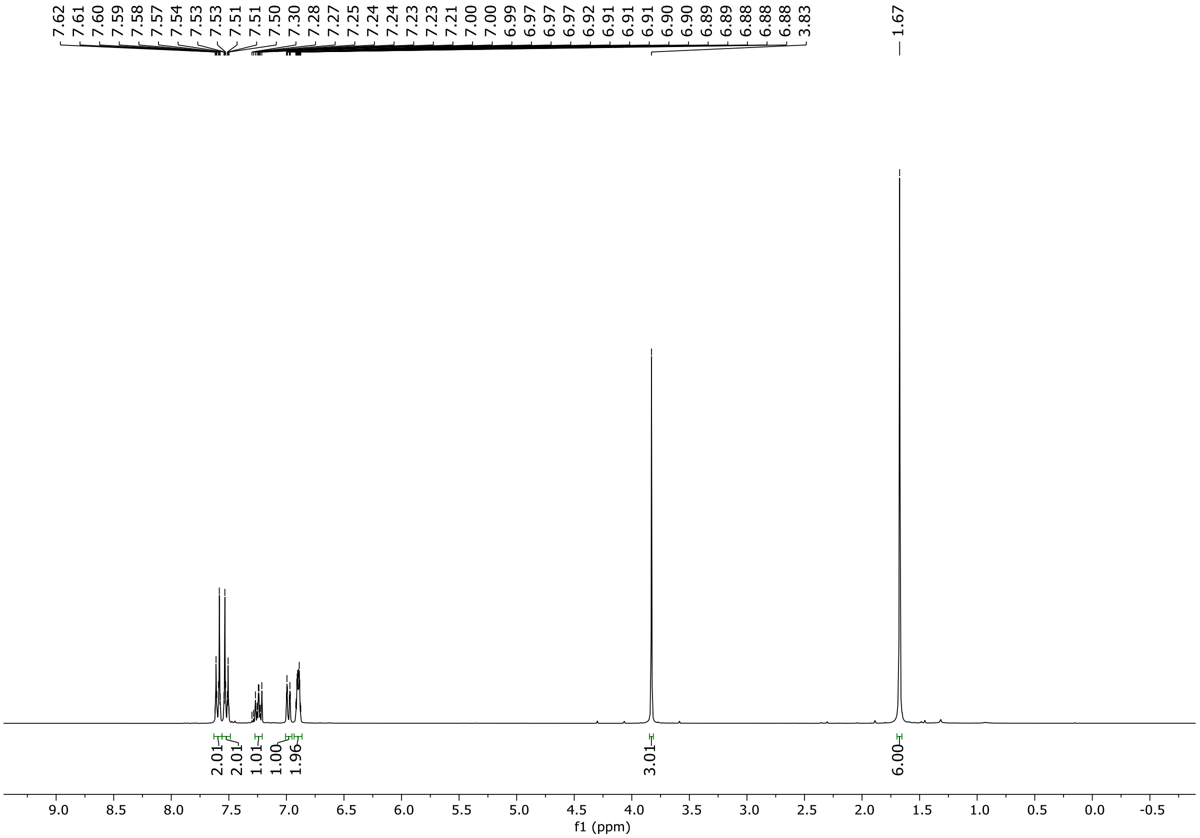

^{13}C{^{1}H} NMR (75.4 MHz, $CDCl_3$)

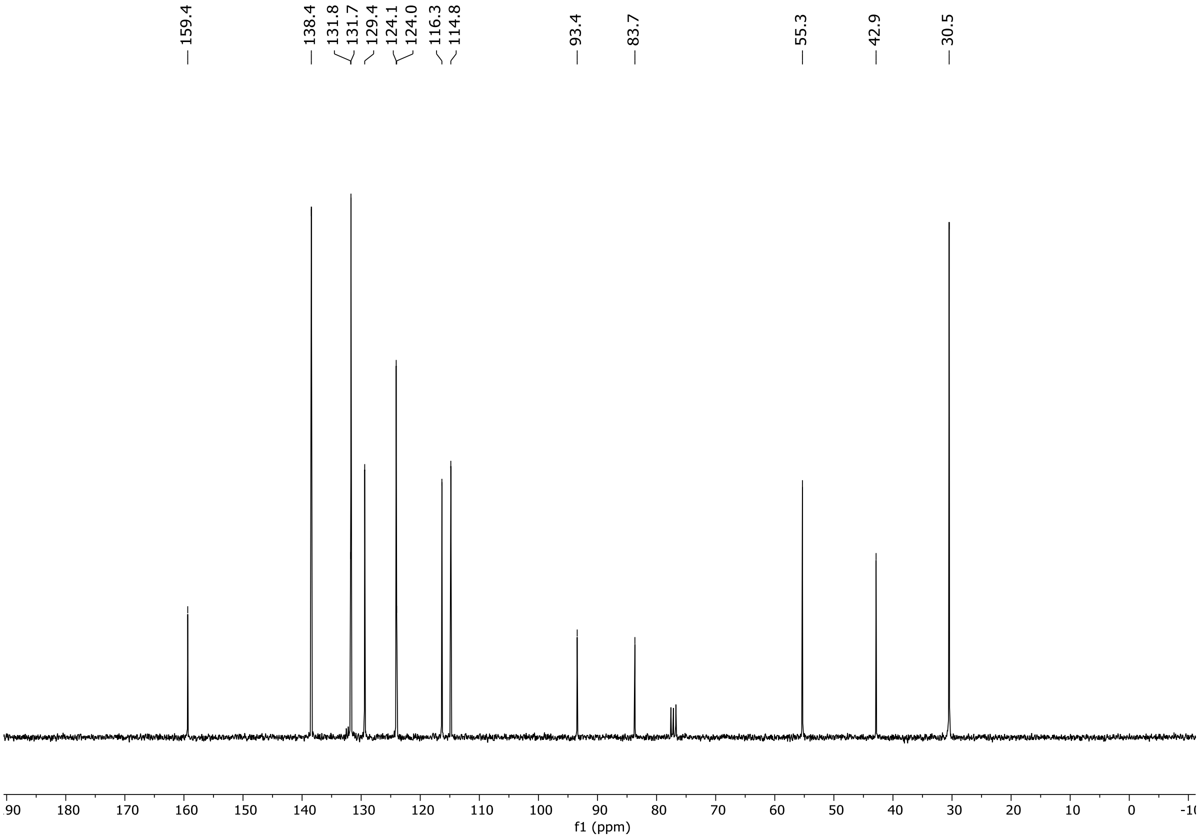

4kl: ^{1}H-NMR (300 MHz, $CDCl_3$)

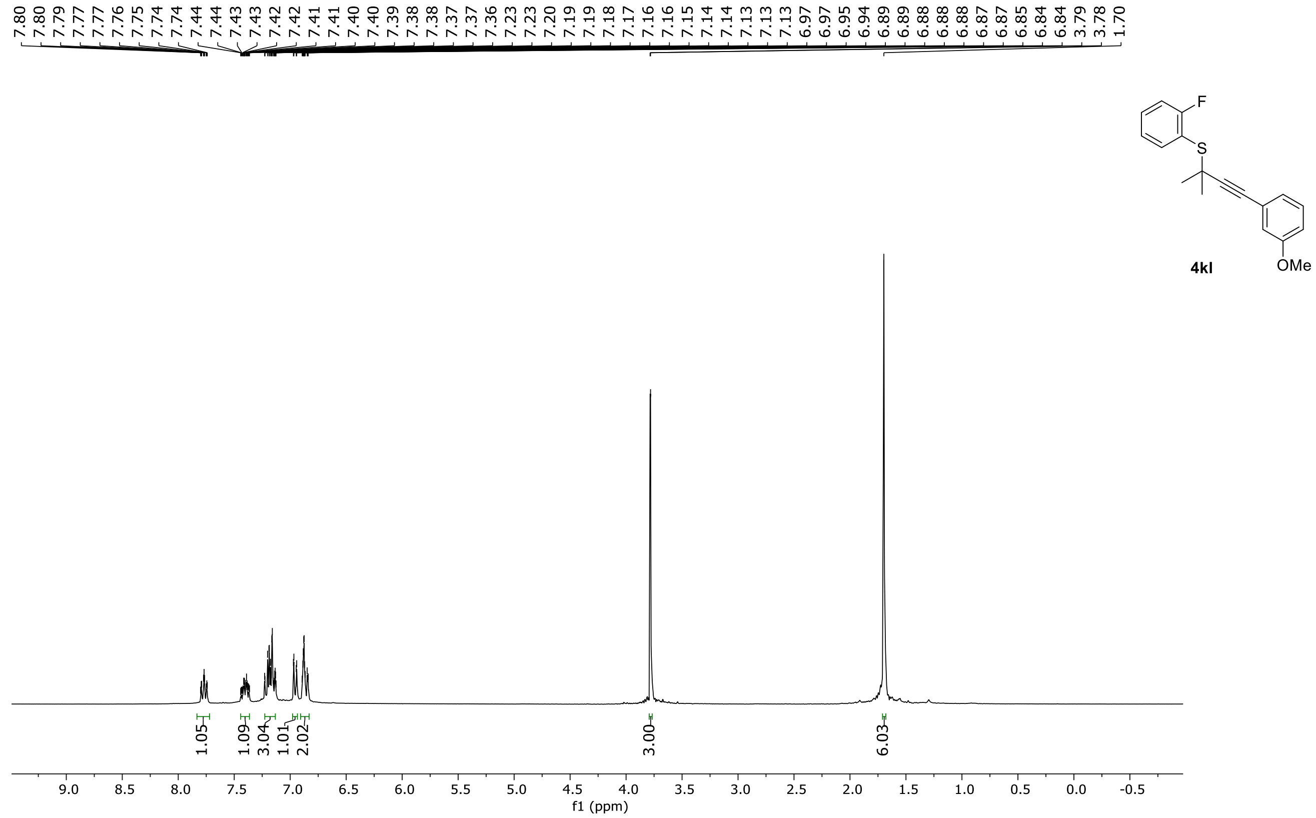

^{13}C{^{1}H} NMR (75.4 MHz, $CDCl_3$)

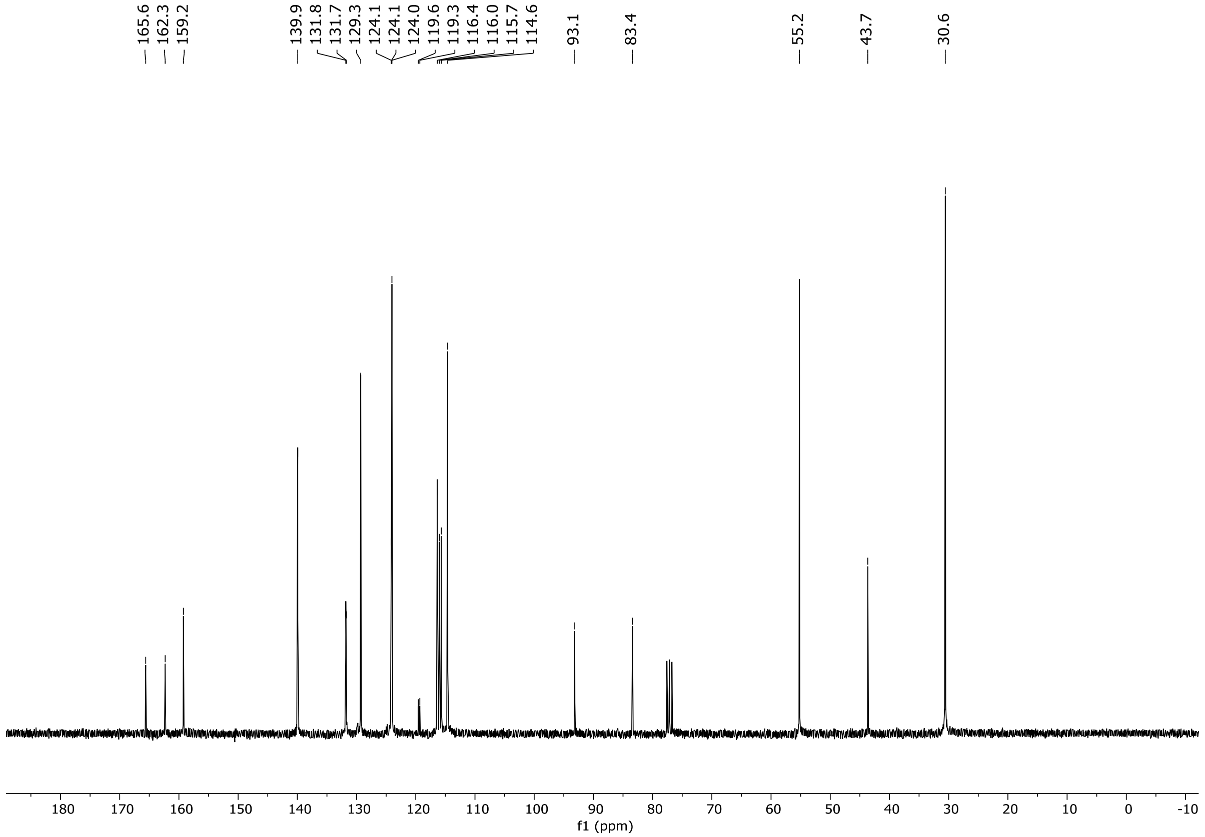

4la: ^{1}H-NMR (300 MHz, $CDCl_3$)

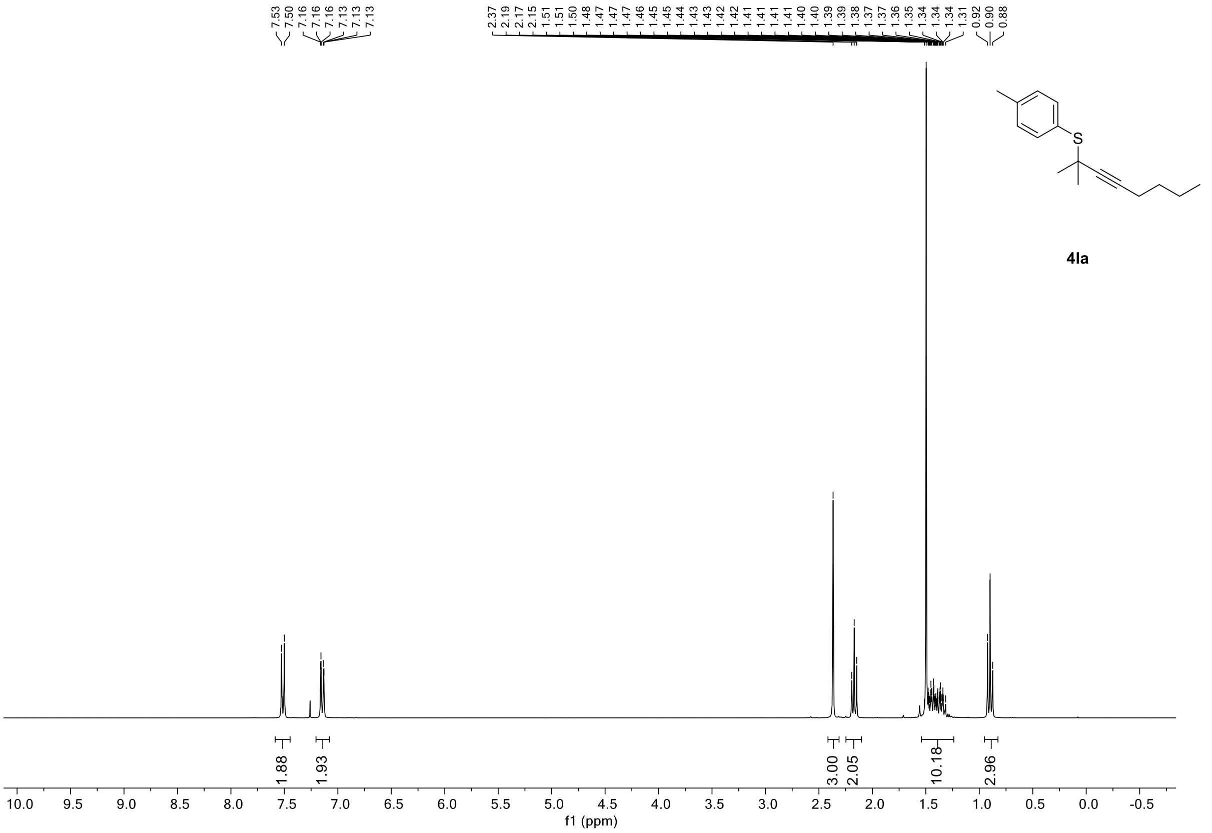

$^{13}C\{^{1}H\}$ NMR (75.4 MHz, $CDCl_3$)

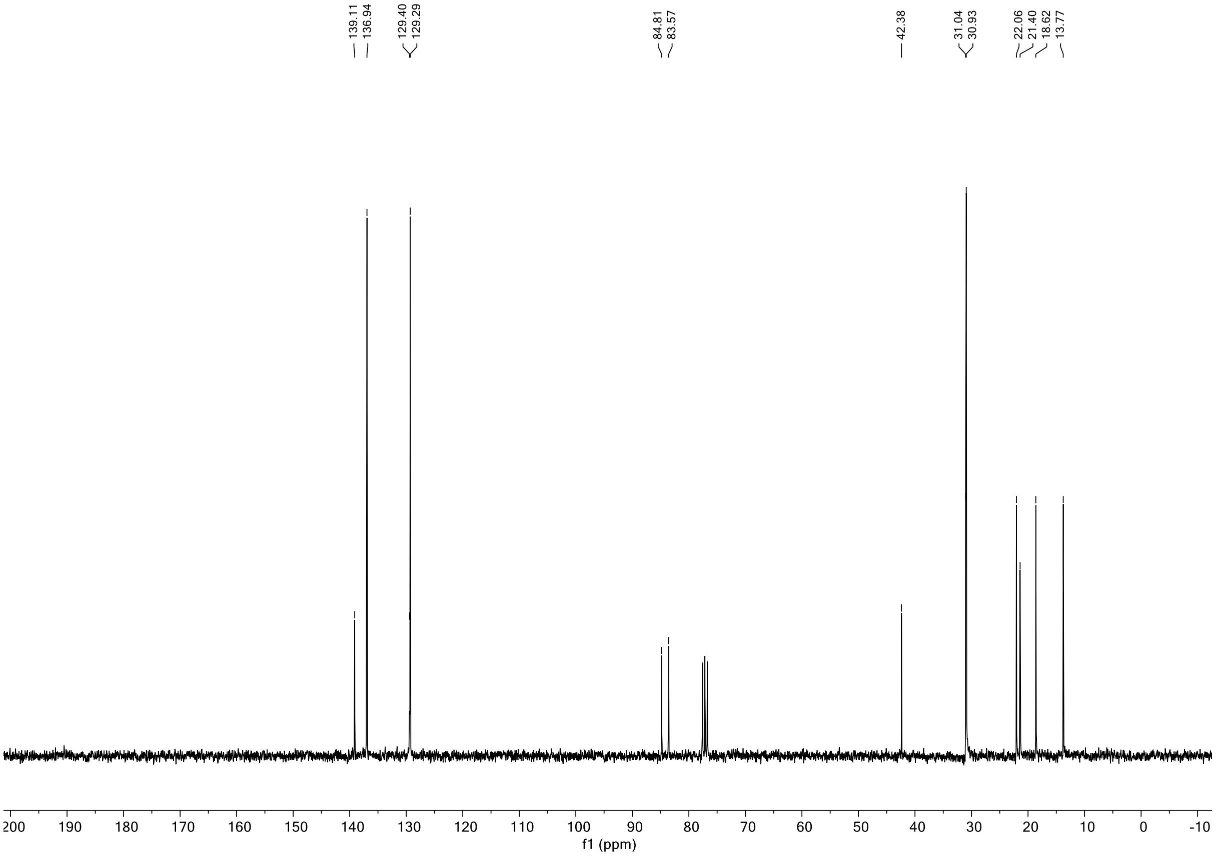

4bm: ^{1}H-NMR (300 MHz, $CDCl_3$)

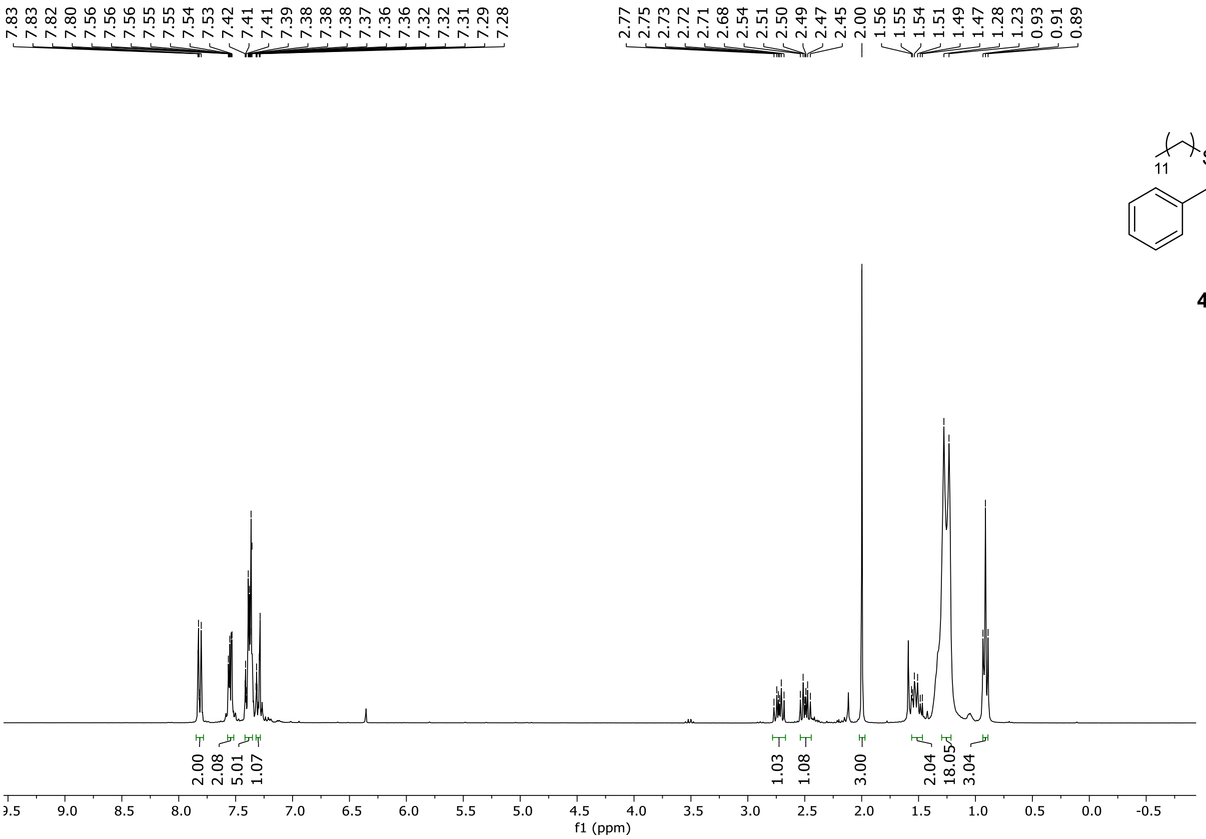

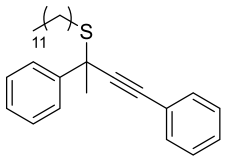

4bm

$^{13}C\{^{1}H\}$ NMR (75.4 MHz, $CDCl_3$)

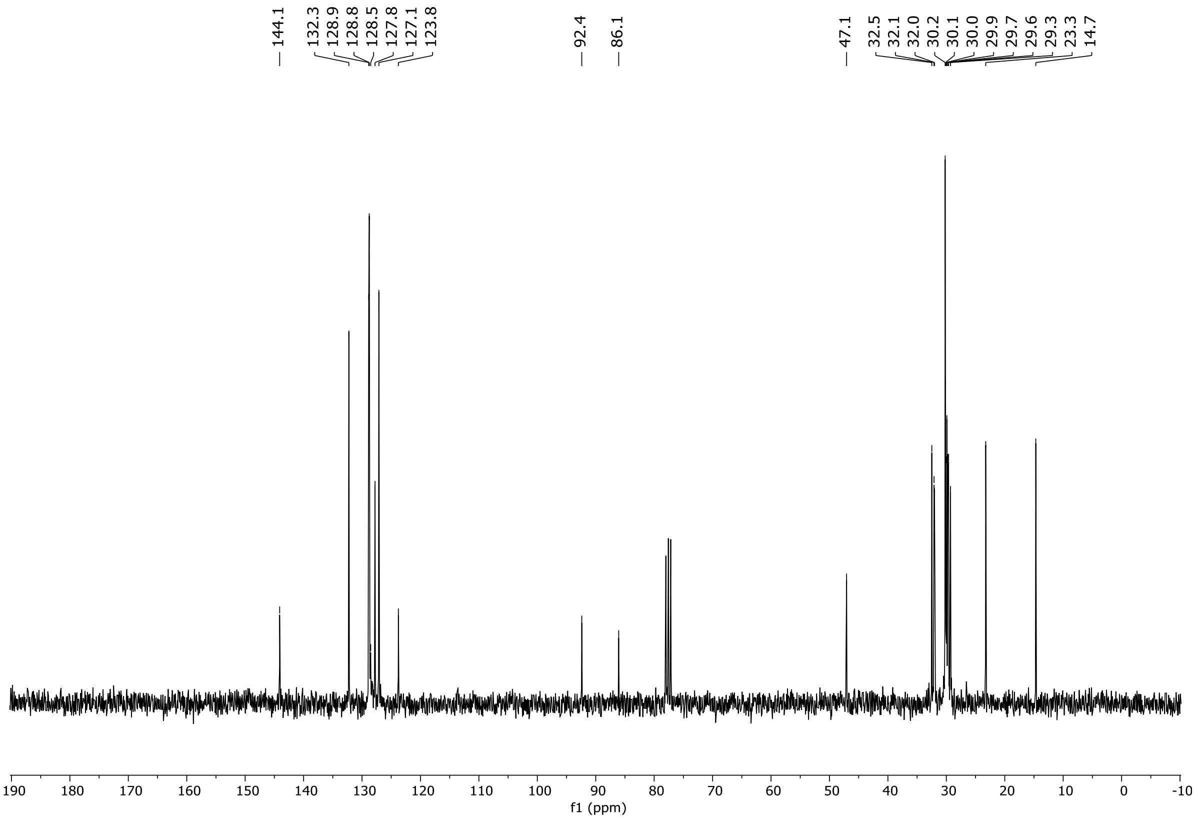

4fn: ^{1}H-NMR (300 MHz, $CDCl_3$)

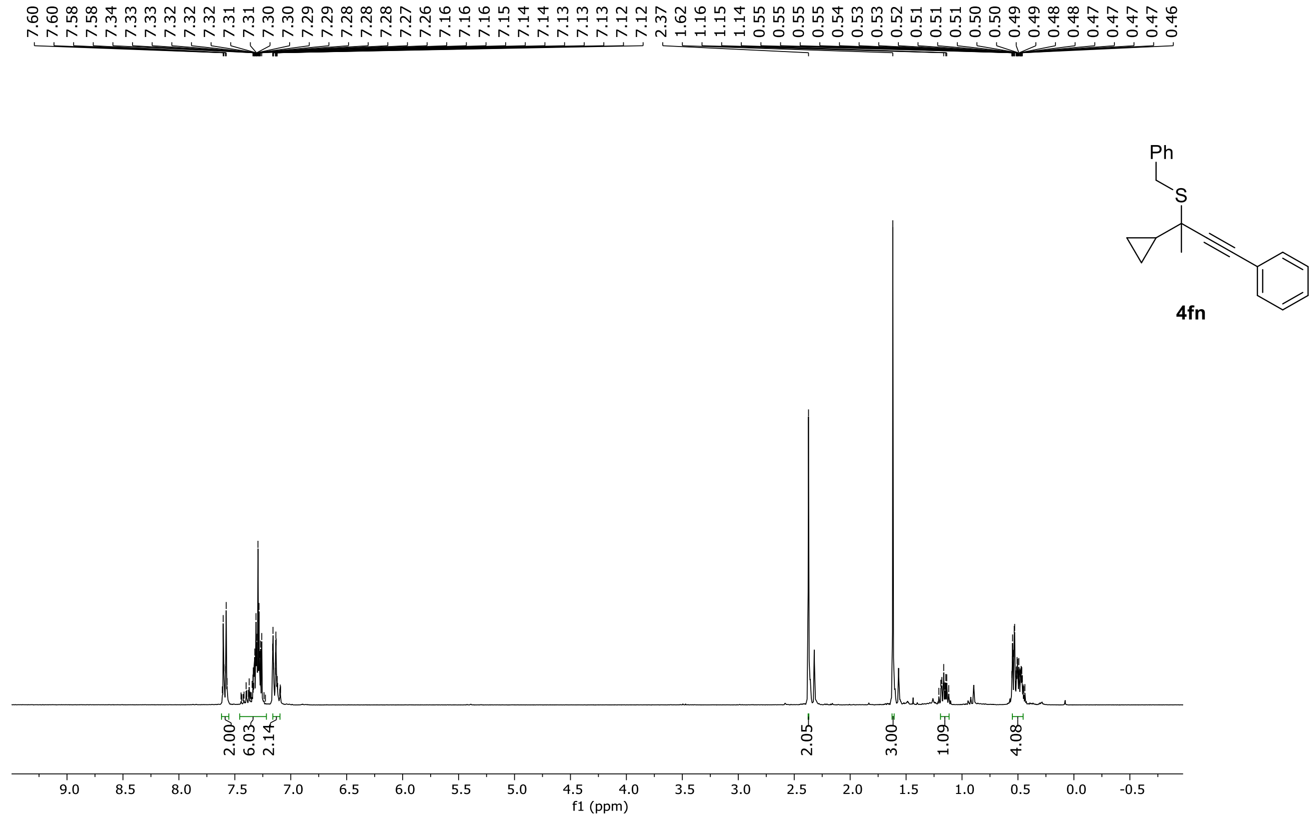

$^{13}C\{^1H\}$ NMR (75.4 MHz, $CDCl_3$)

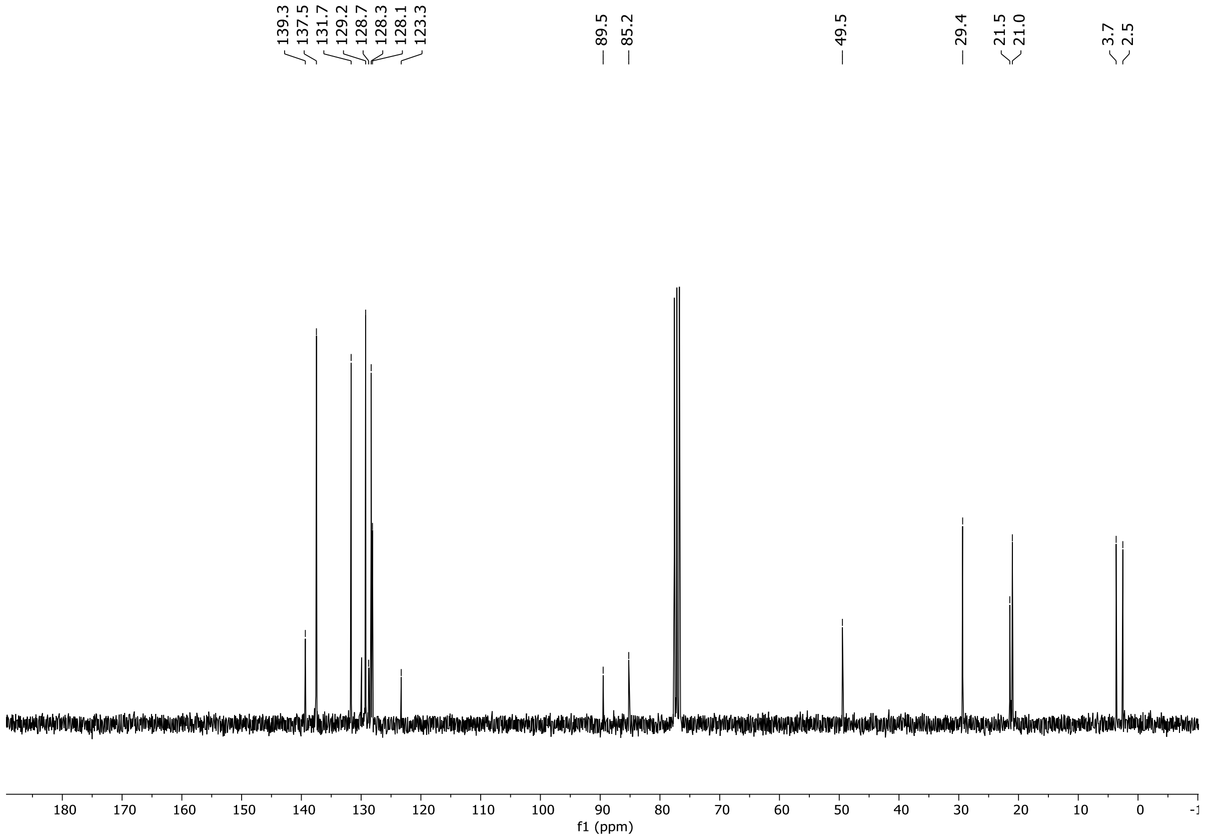

6: ^{1}H-NMR (300 MHz, $CDCl_3$)

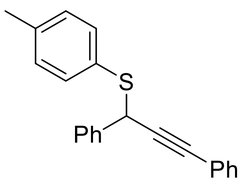

6

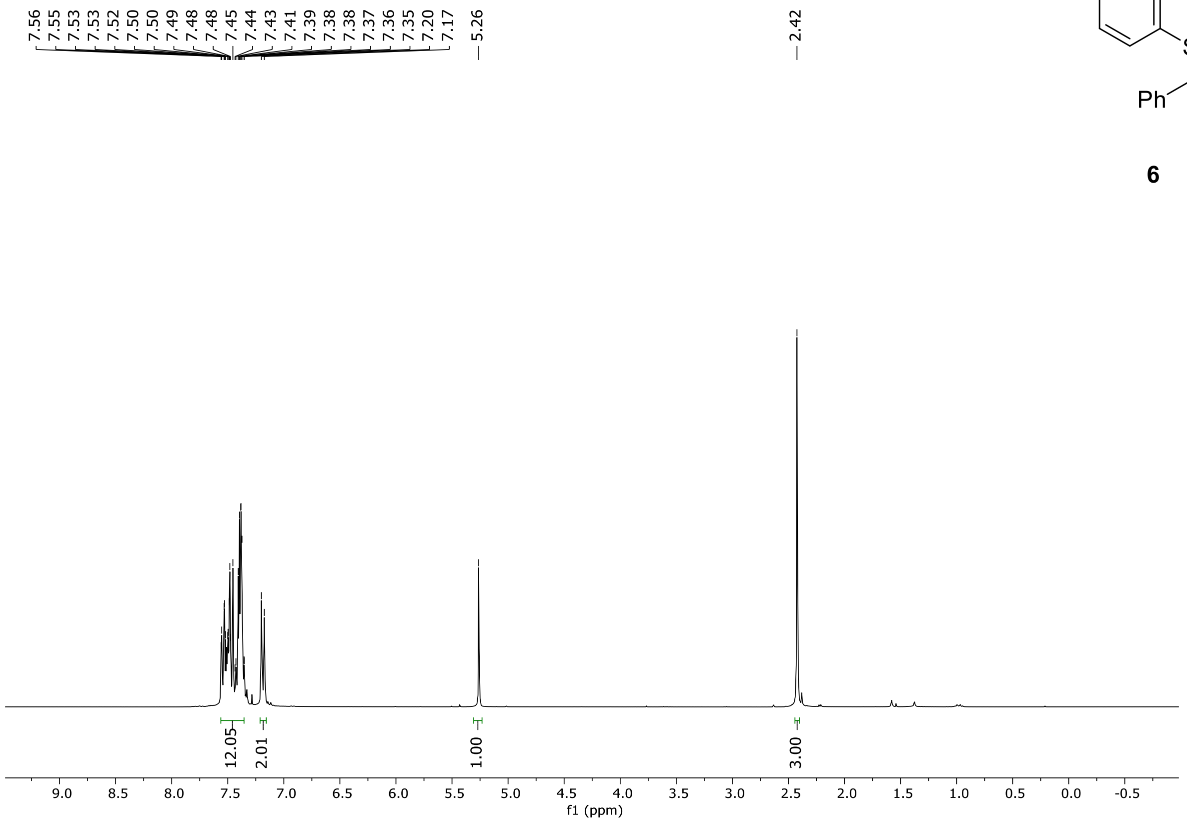

^{13}C{^{1}H} NMR (75.4 MHz, $CDCl_3$)

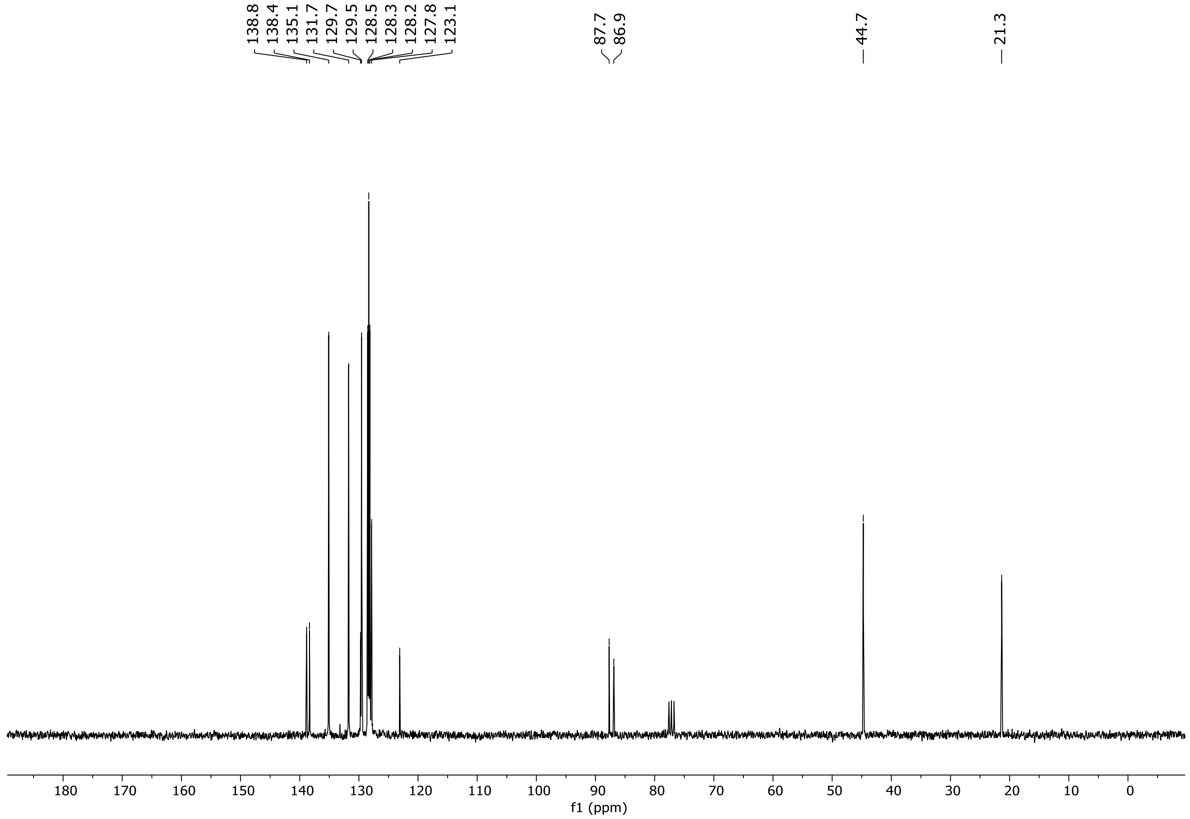

7aa: ^{1}H-NMR (300 MHz, $CDCl_3$)

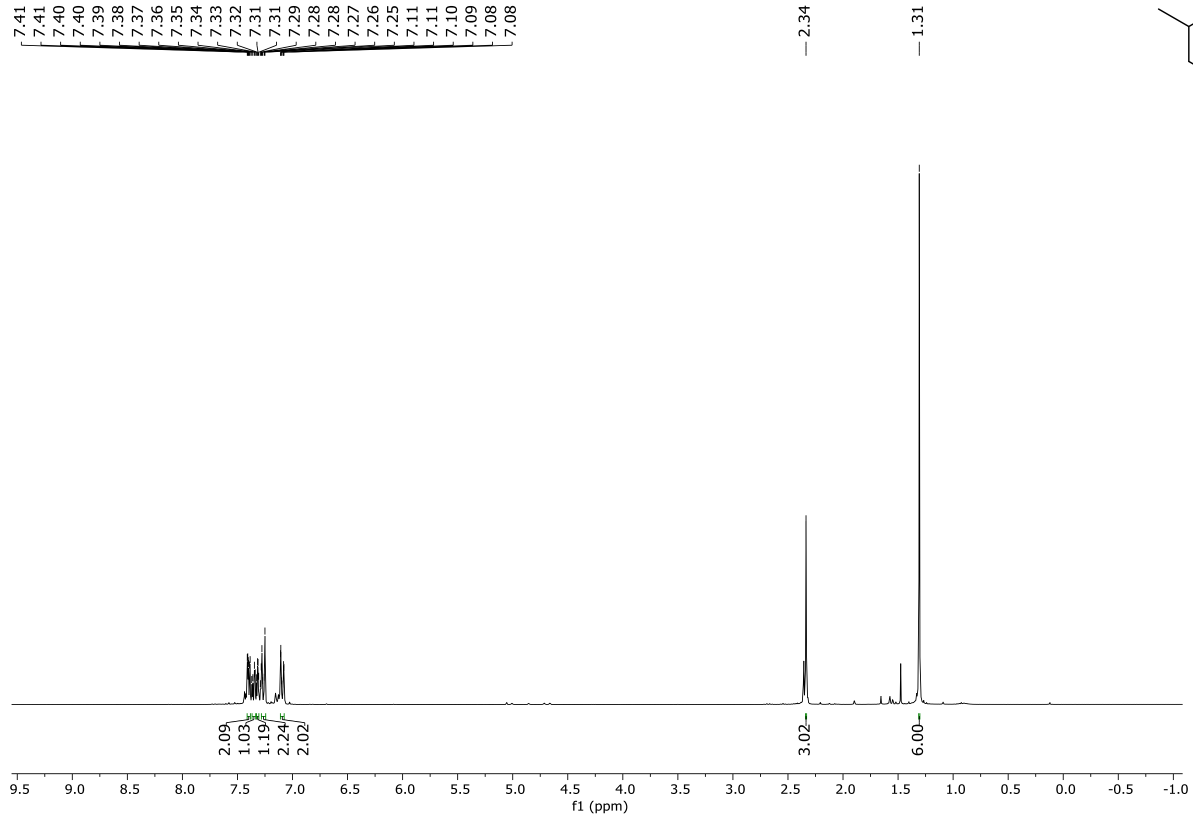

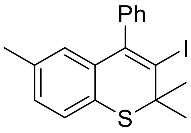

7aa

^{13}C{^{1}H} NMR (75.4 MHz, $CDCl_3$)

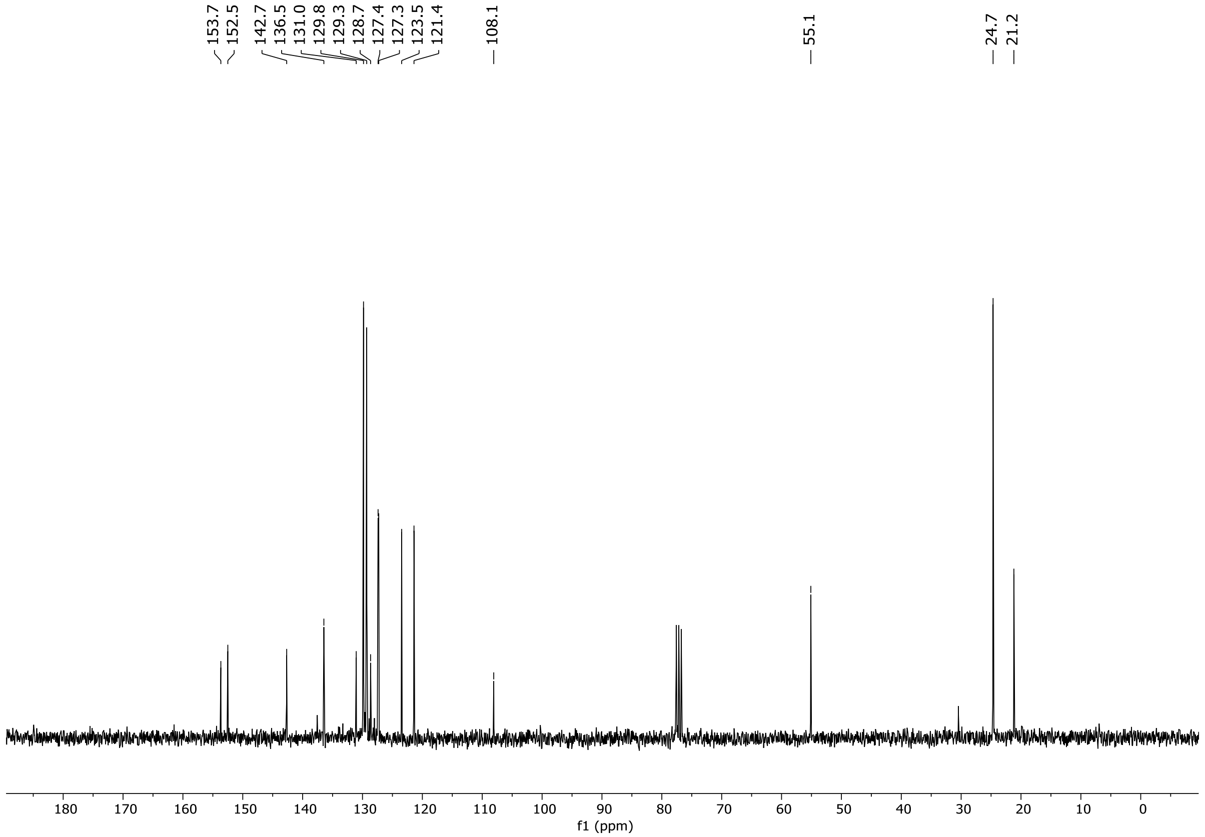

7ab: ^{1}H-NMR (300 MHz, $CDCl_3$)

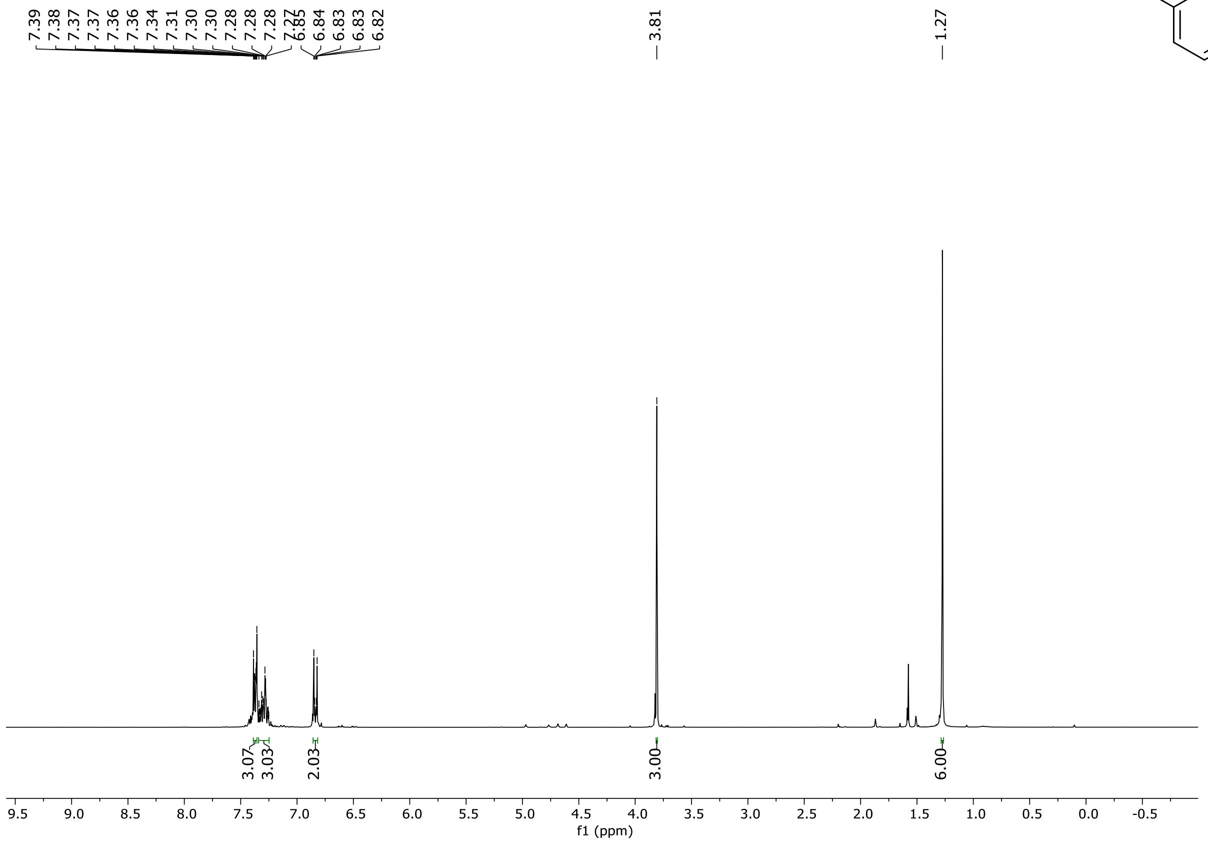

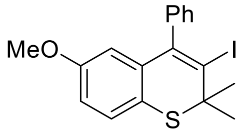

7ab

^{13}C{^{1}H} NMR (75.4 MHz, $CDCl_3$)

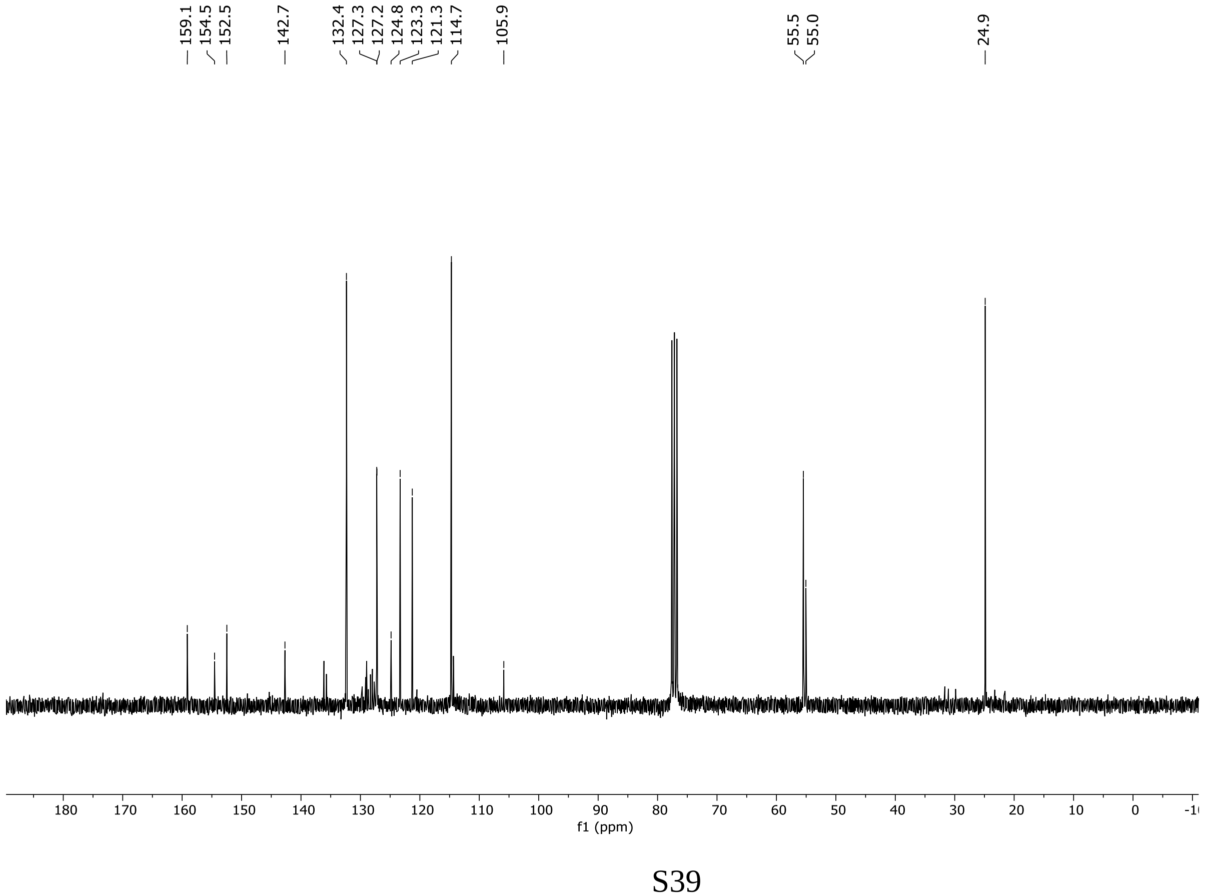

7ac: ^{1}H-NMR (300 MHz, CDCl$_3$)

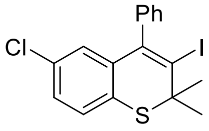

7ac

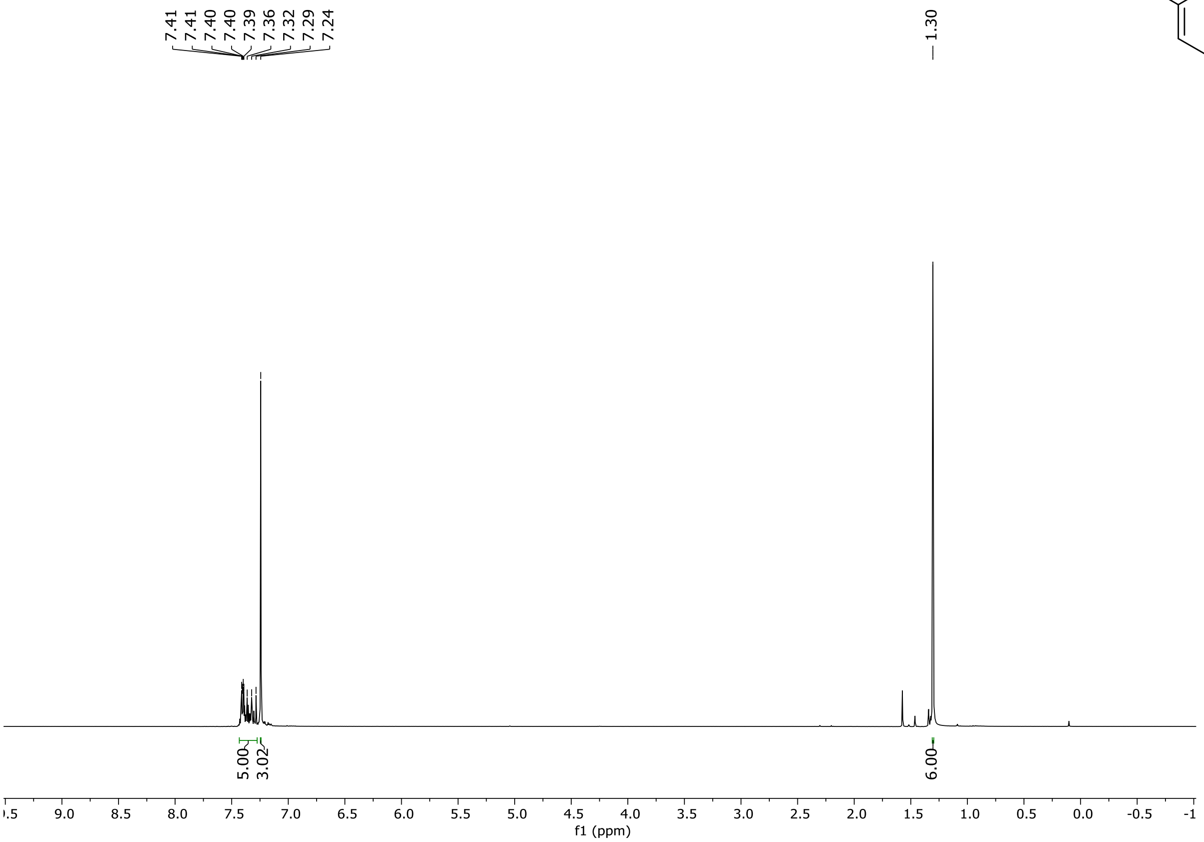

^{13}C{^{1}H} NMR (75.4 MHz, CDCl$_3$)

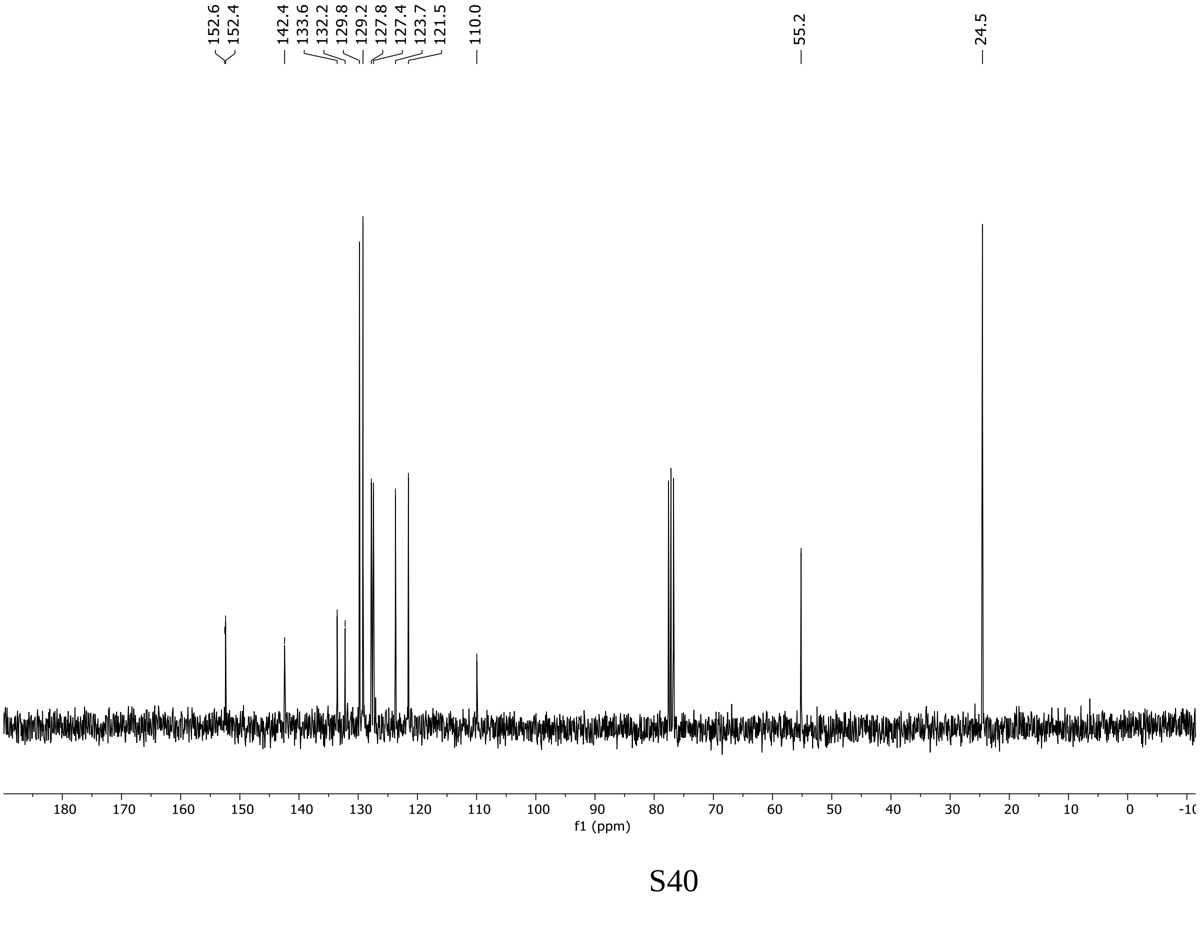

7ad: ^{1}H-NMR (300 MHz, $CDCl_3$)

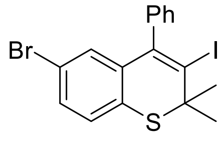

7ad

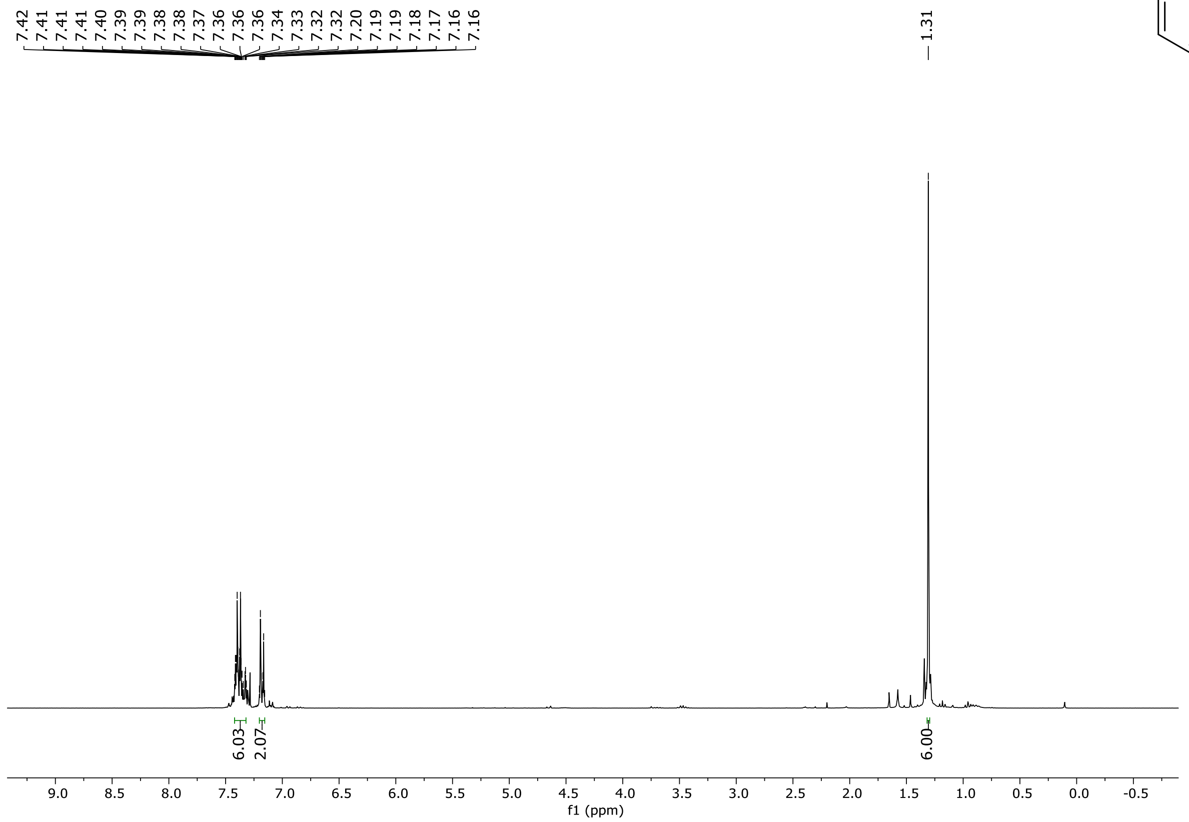

^{13}C{^{1}H} NMR (75.4 MHz, $CDCl_3$)

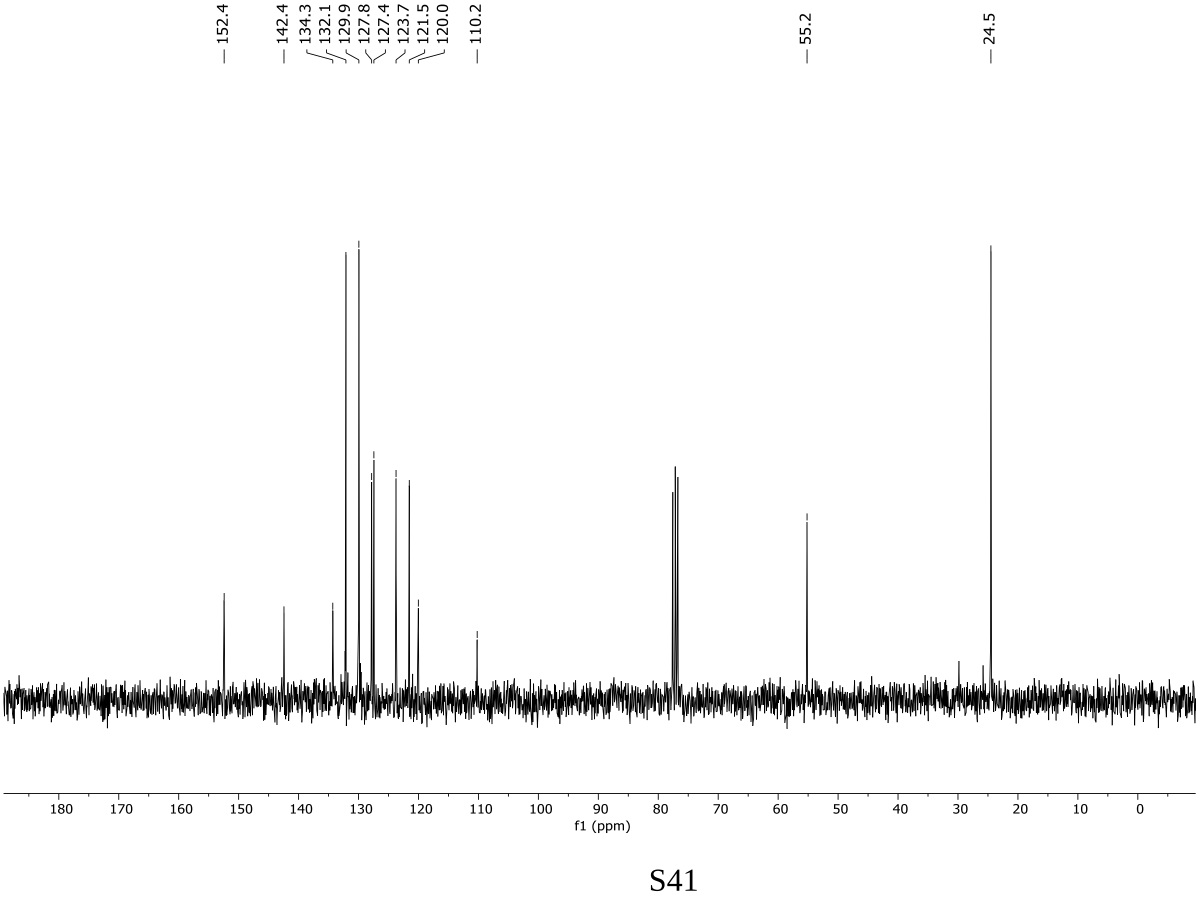

7ae: ^{1}H-NMR (300 MHz, $CDCl_3$)

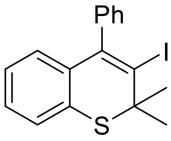

7ae

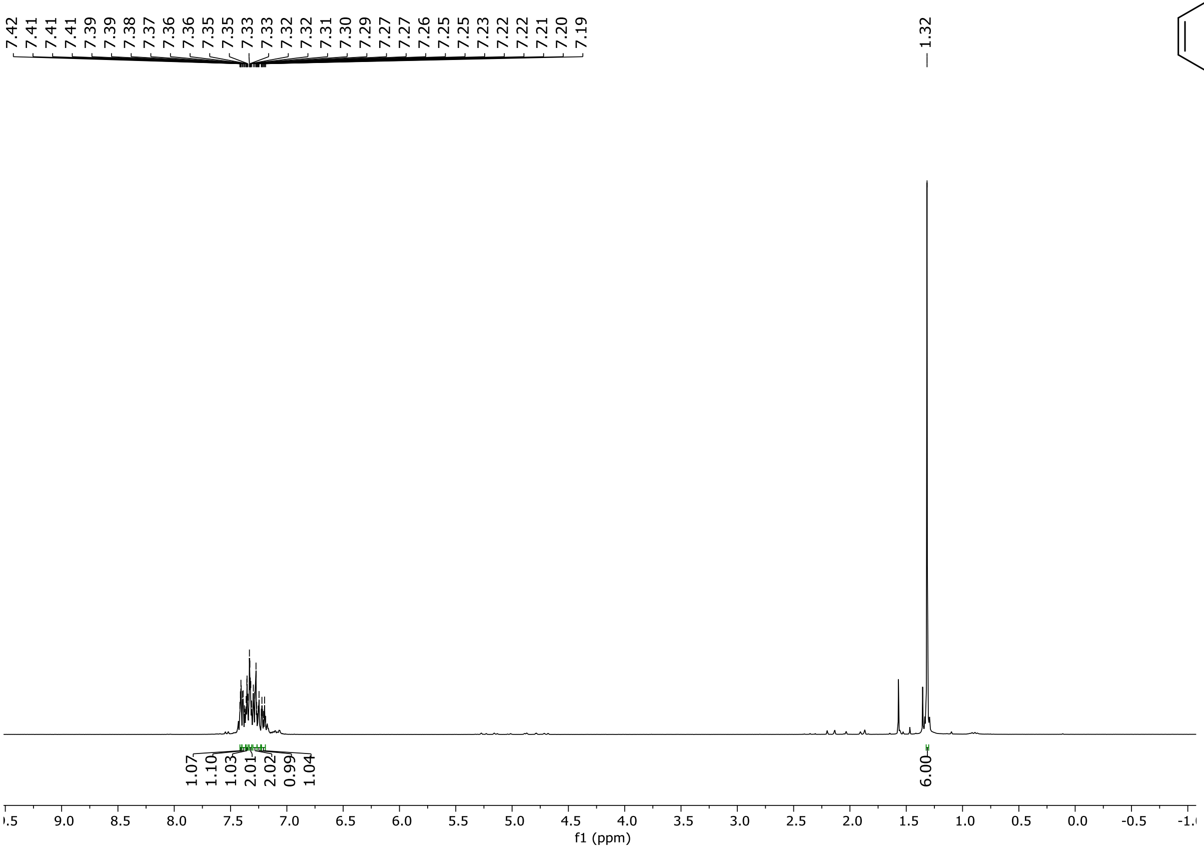

$^{13}C\{^1H\}$ NMR (75.4 MHz, $CDCl_3$)

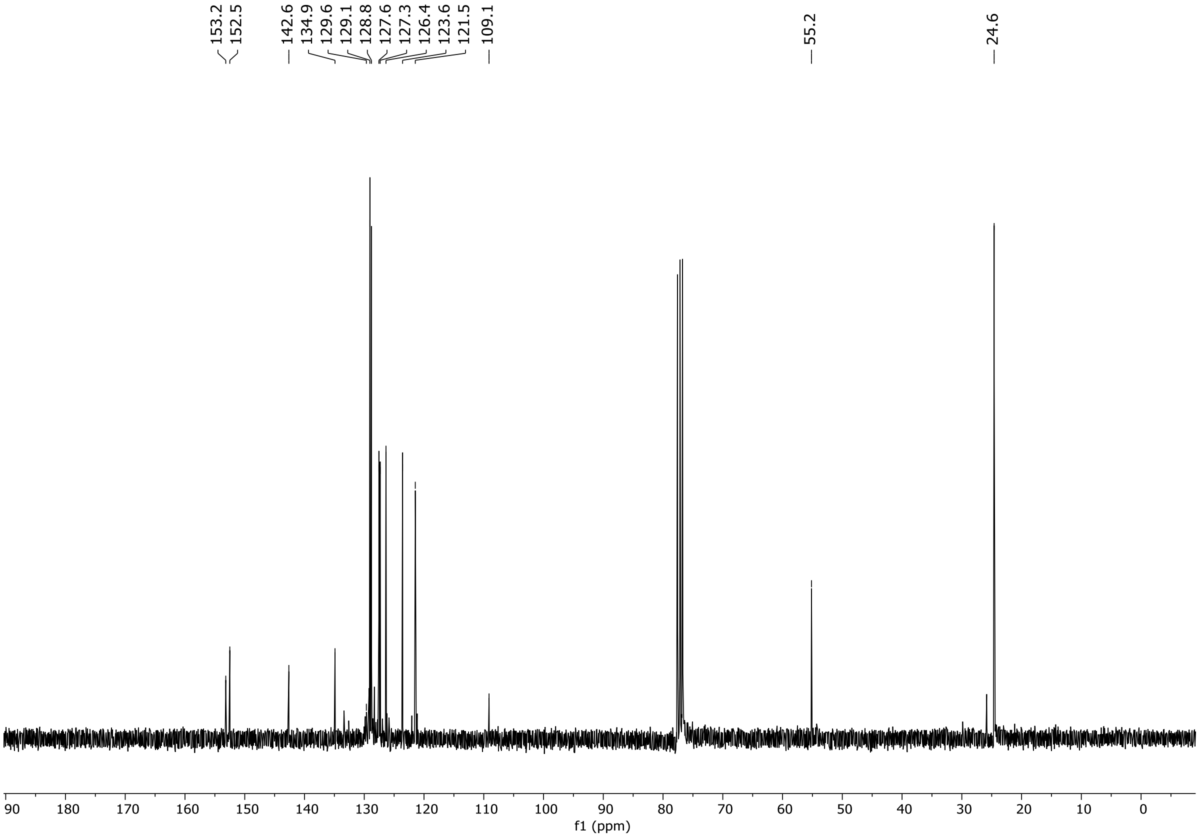

7ai: ^{1}H-NMR (300 MHz, $CDCl_3$)

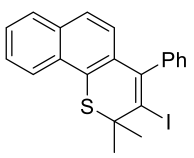

7ai

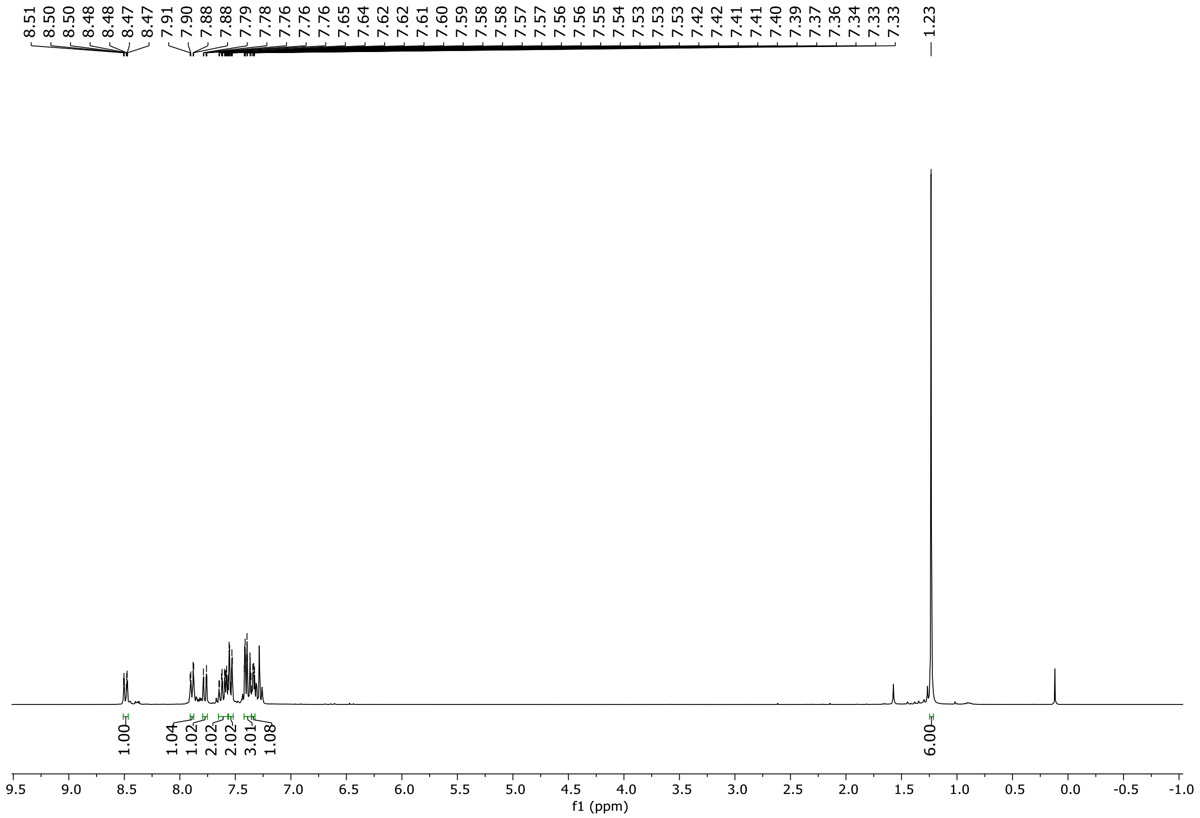

^{13}C{^{1}H} NMR (75.4 MHz, $CDCl_3$)

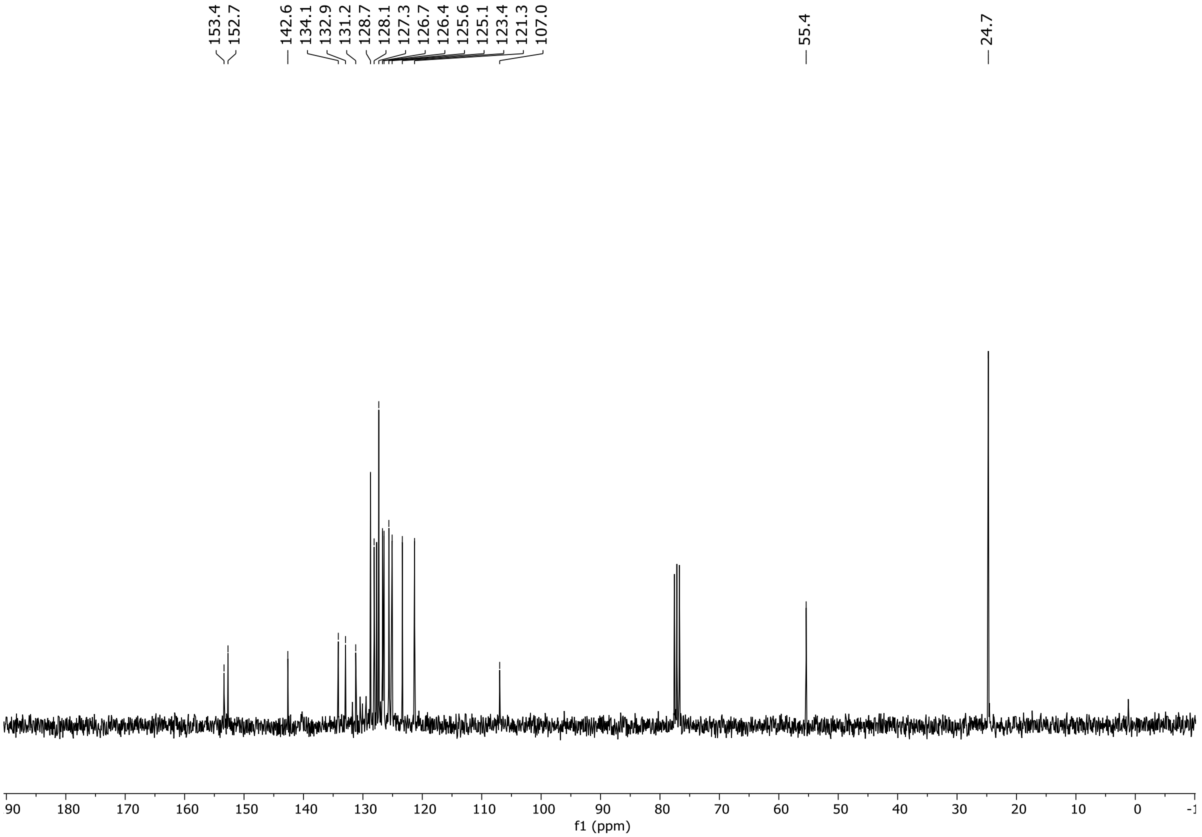

7ai: ^{1}H-NMR (300 MHz, $CDCl_3$)

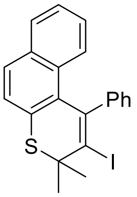

7aj

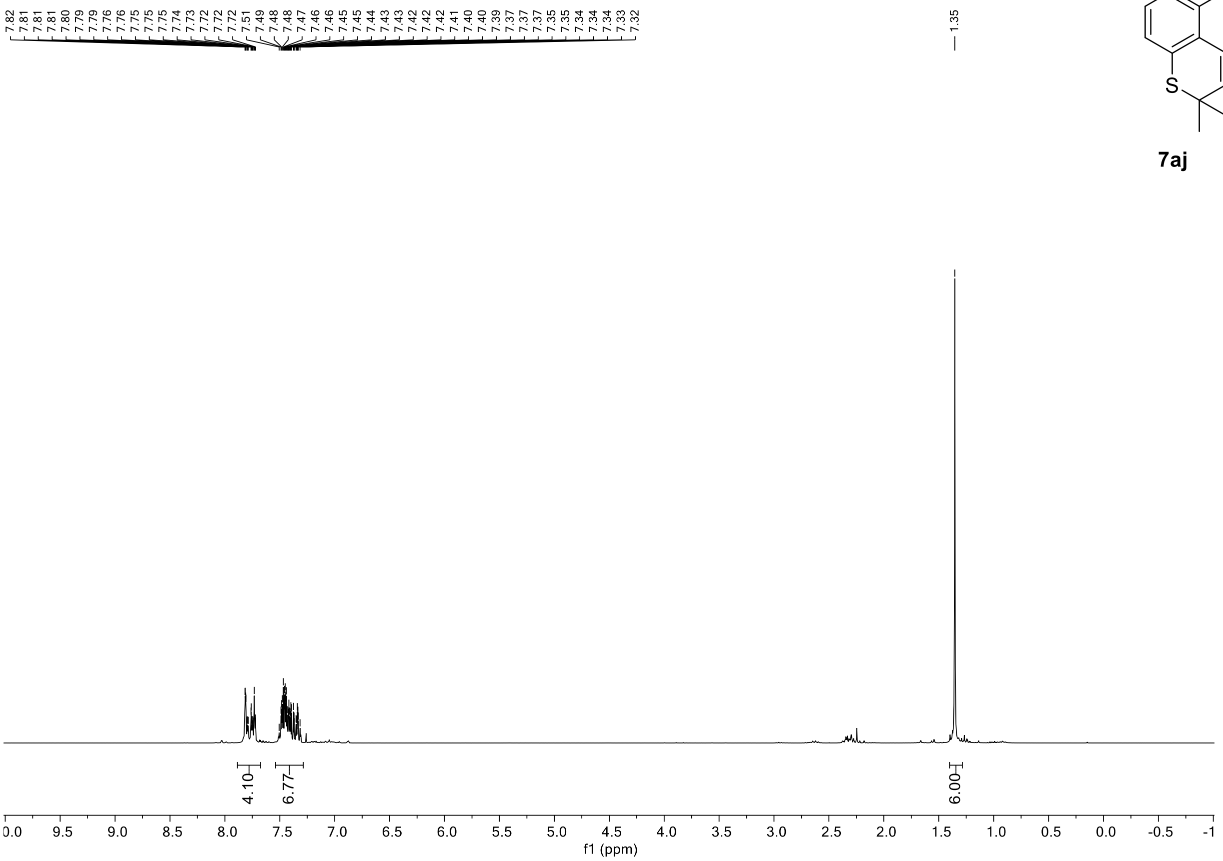

^{13}C{^{1}H} NMR (75.4 MHz, $CDCl_3$)

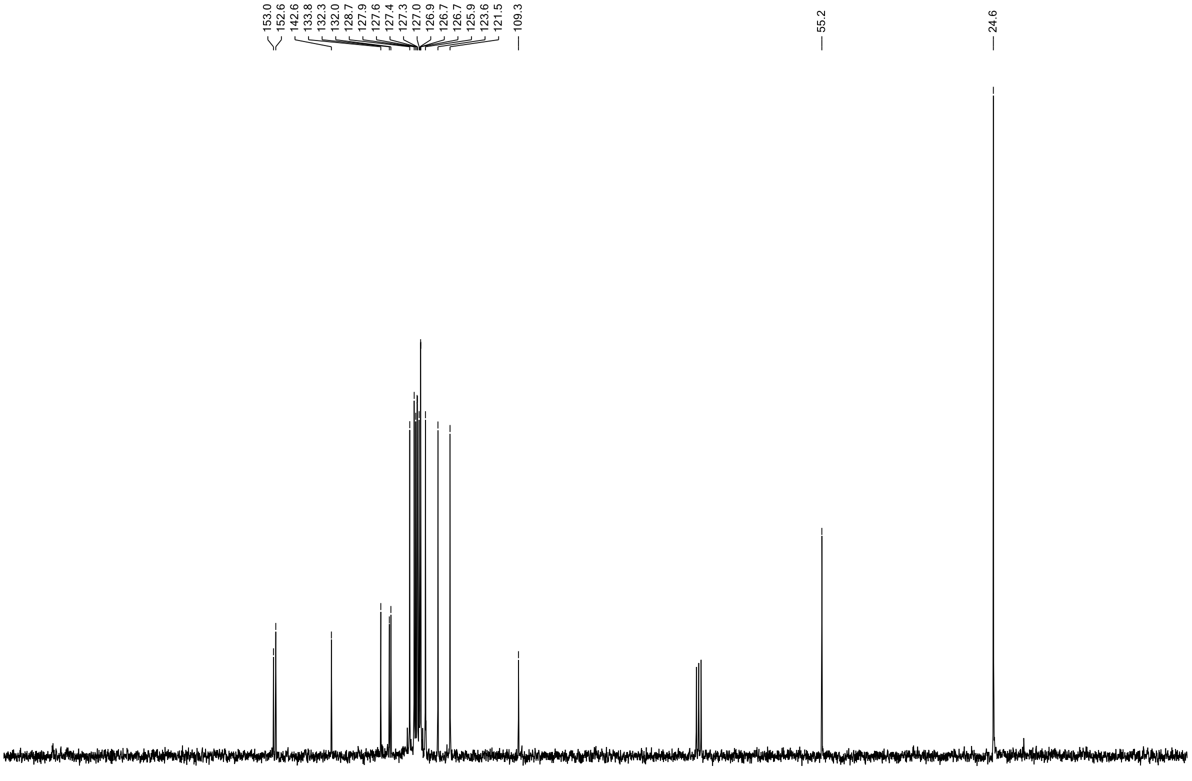

7ia: ¹H-NMR (300 MHz, $CDCl_3$)

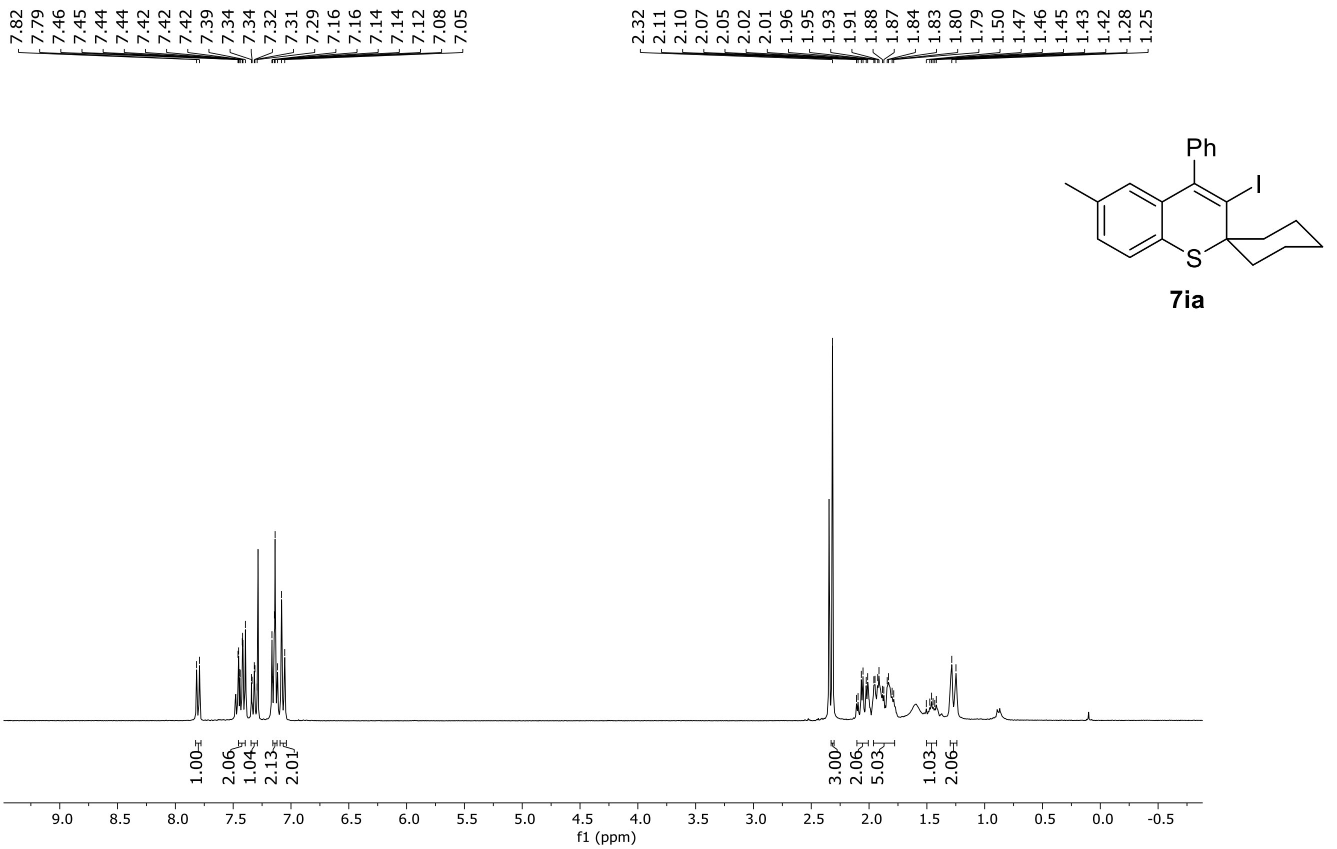

$^{13}C\{^1H\}$ NMR (75.4 MHz, $CDCl_3$)

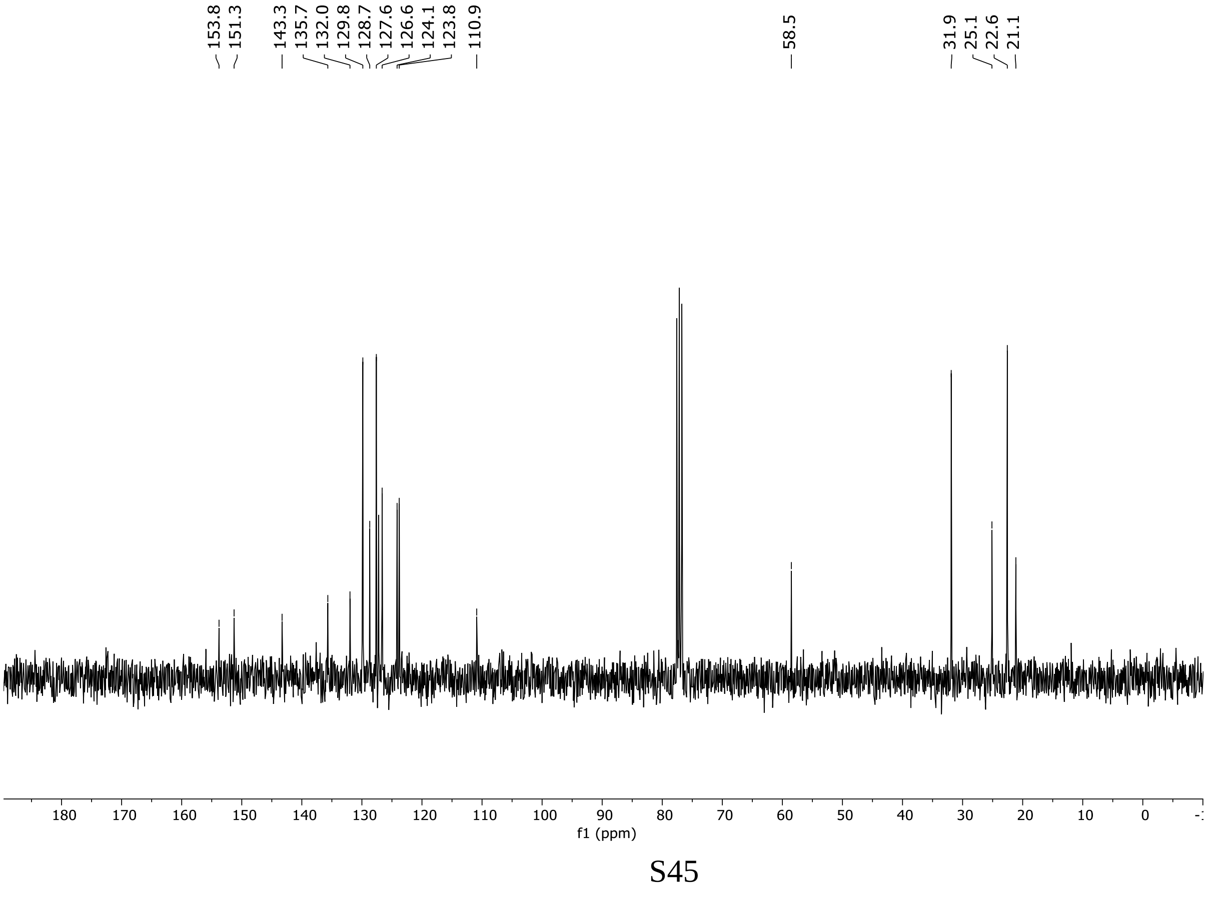

7ka: ^{1}H-NMR (300 MHz, $CDCl_3$)

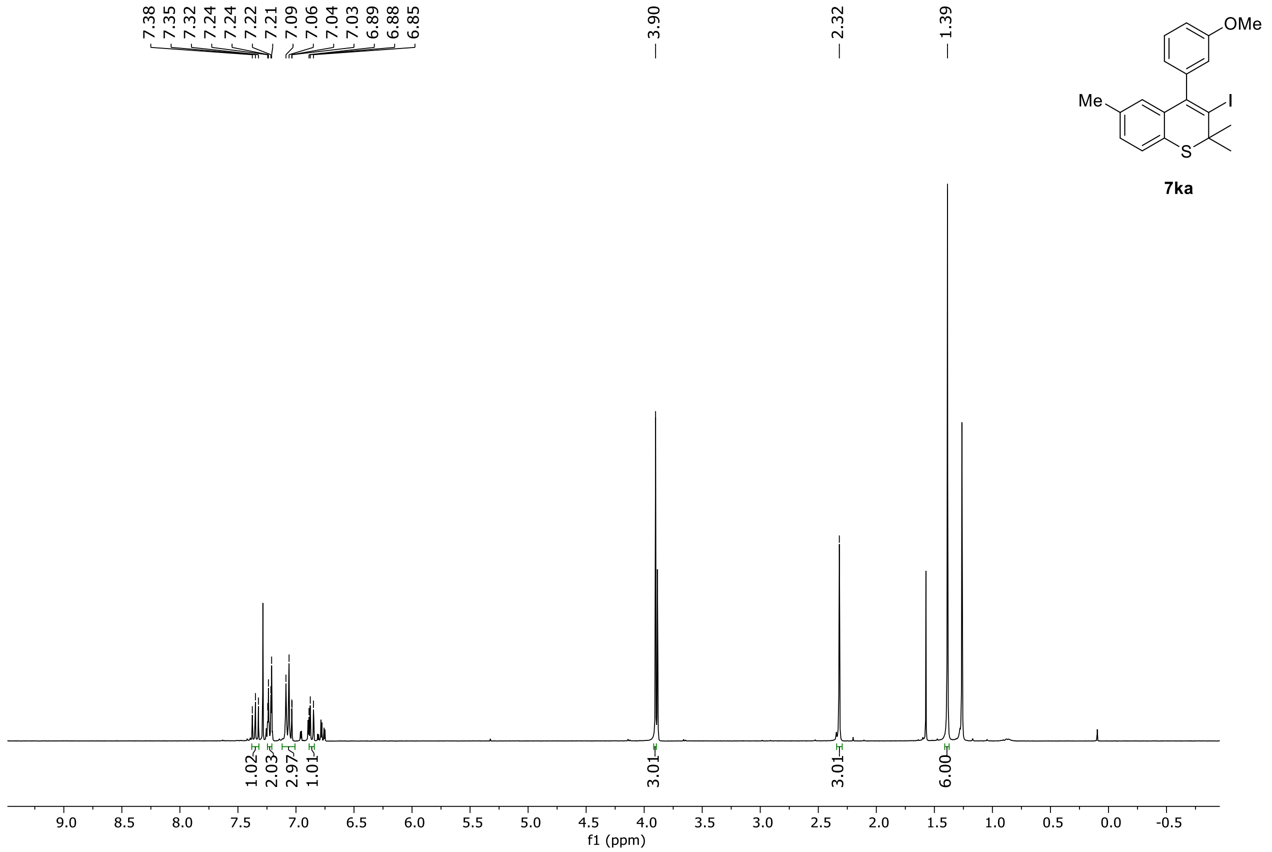

^{13}C{^{1}H} NMR (75.4 MHz, $CDCl_3$)

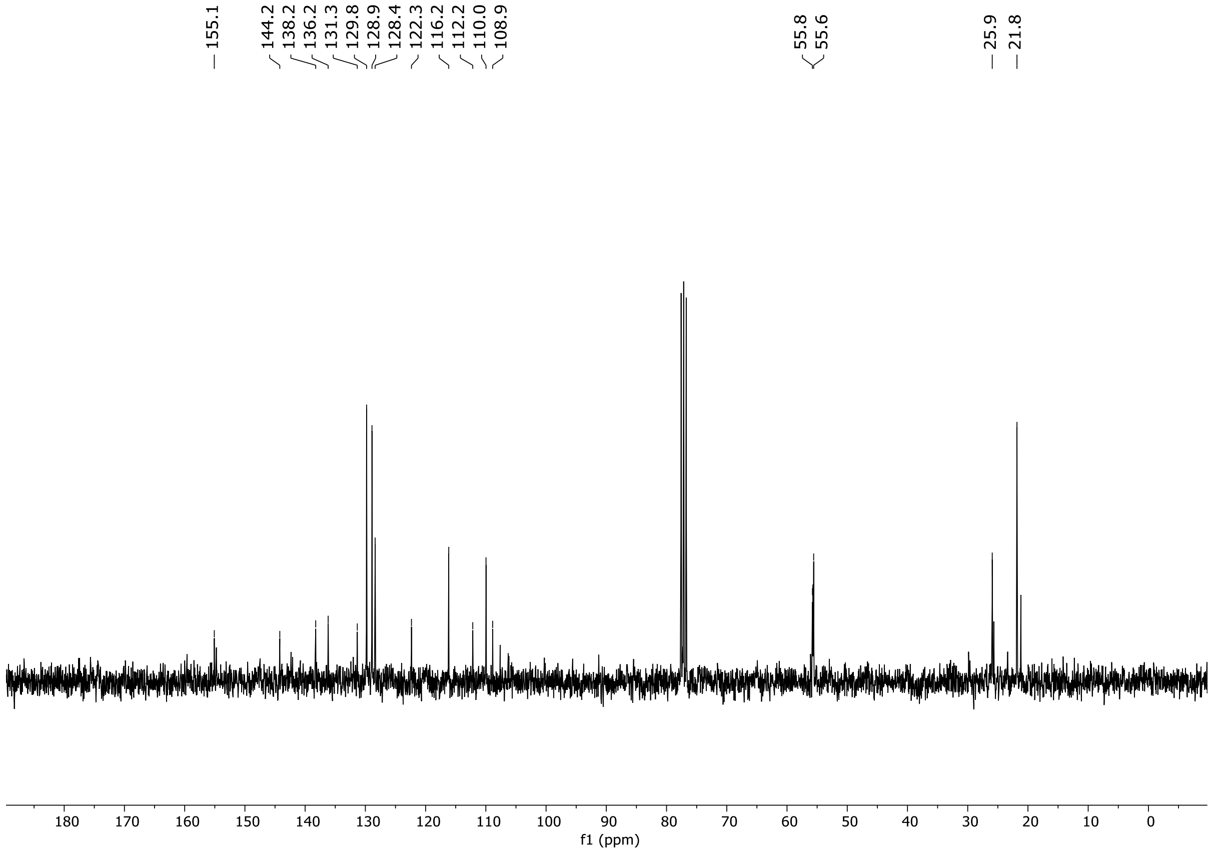

7kd: ^{1}H-NMR (300 MHz, $CDCl_3$)

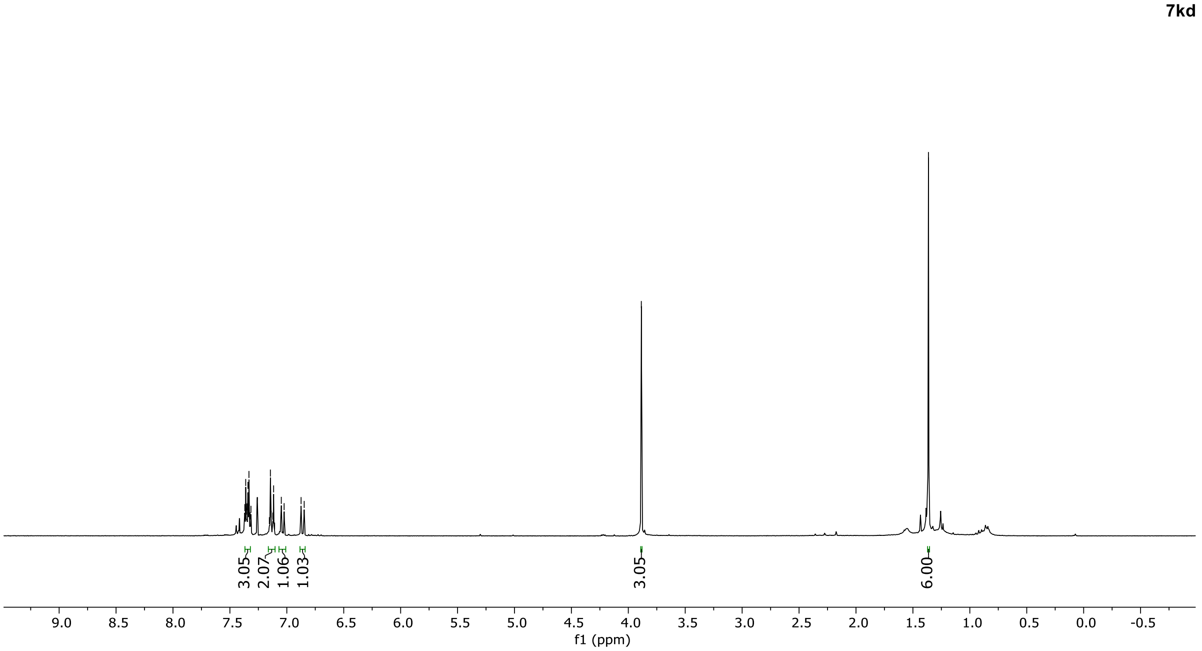

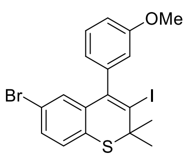

7kd

^{13}C{^{1}H} NMR (75.4 MHz, $CDCl_3$)

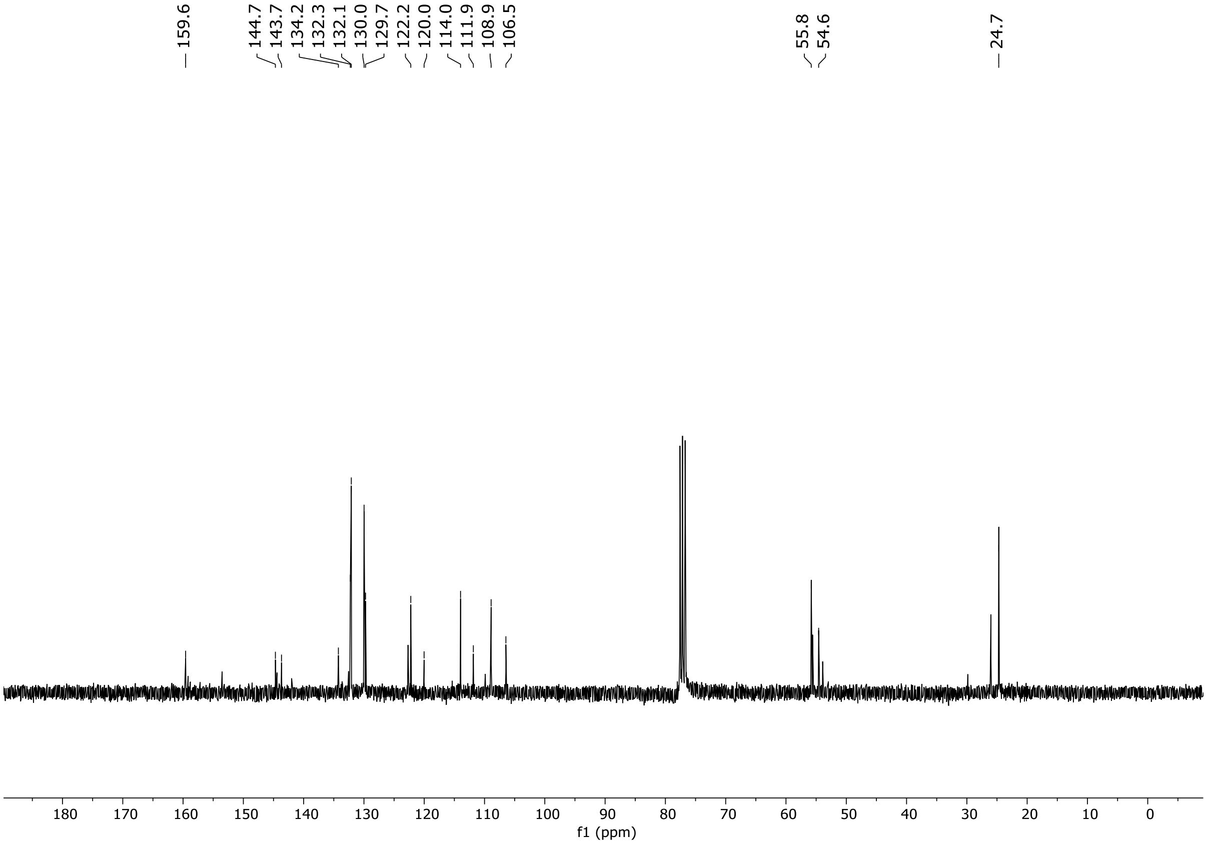

7kl: ^{1}H-NMR (300 MHz, $CDCl_3$)

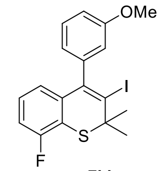

7kl

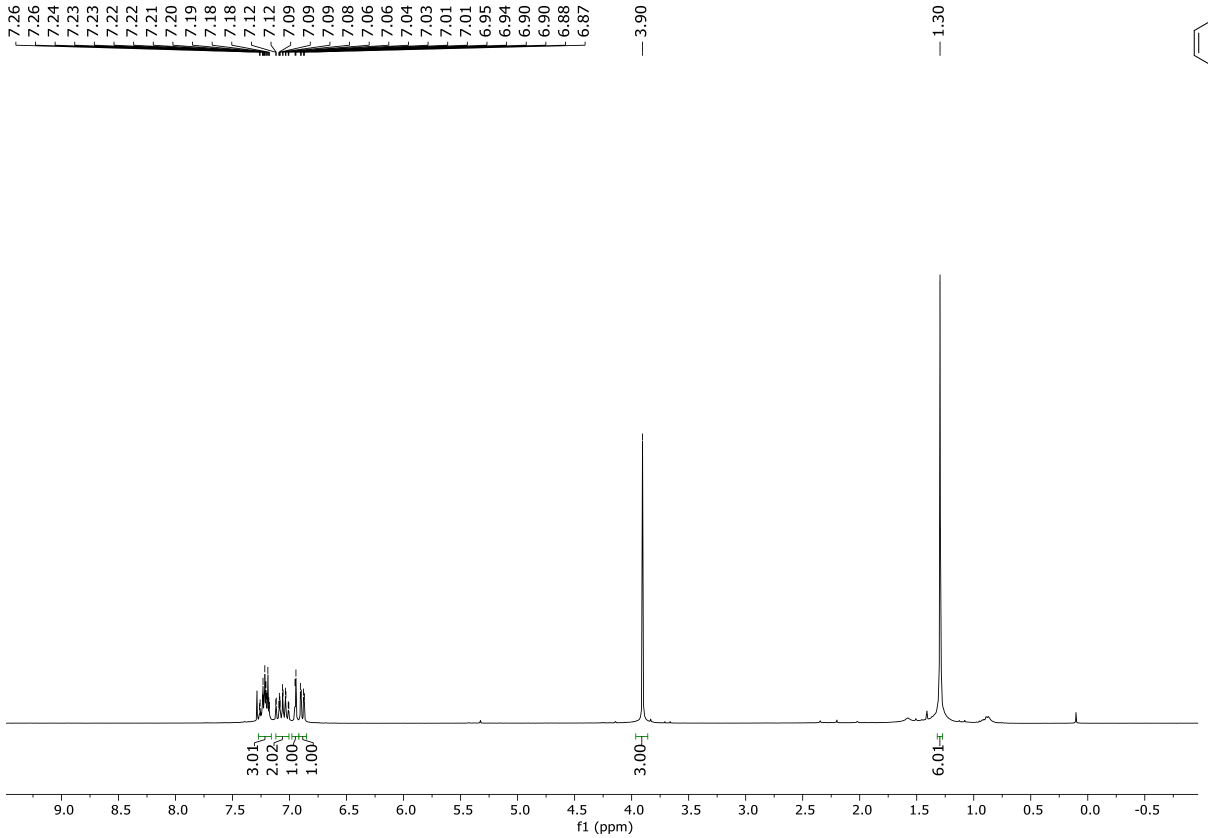

$^{13}C\{^{1}H\}$ NMR (75.4 MHz, $CDCl_3$)

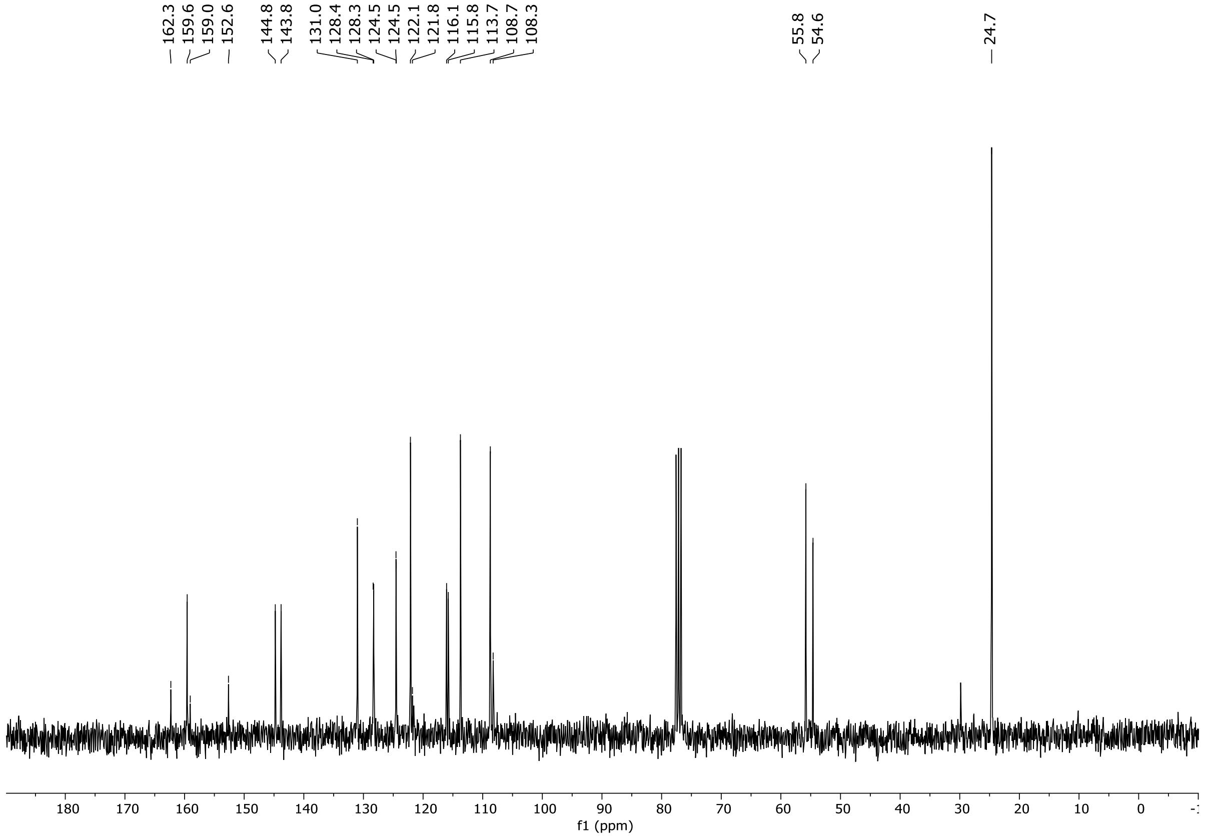

8aa: ^{1}H-NMR (300 MHz, $CDCl_3$)

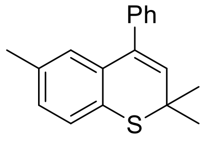

8aa

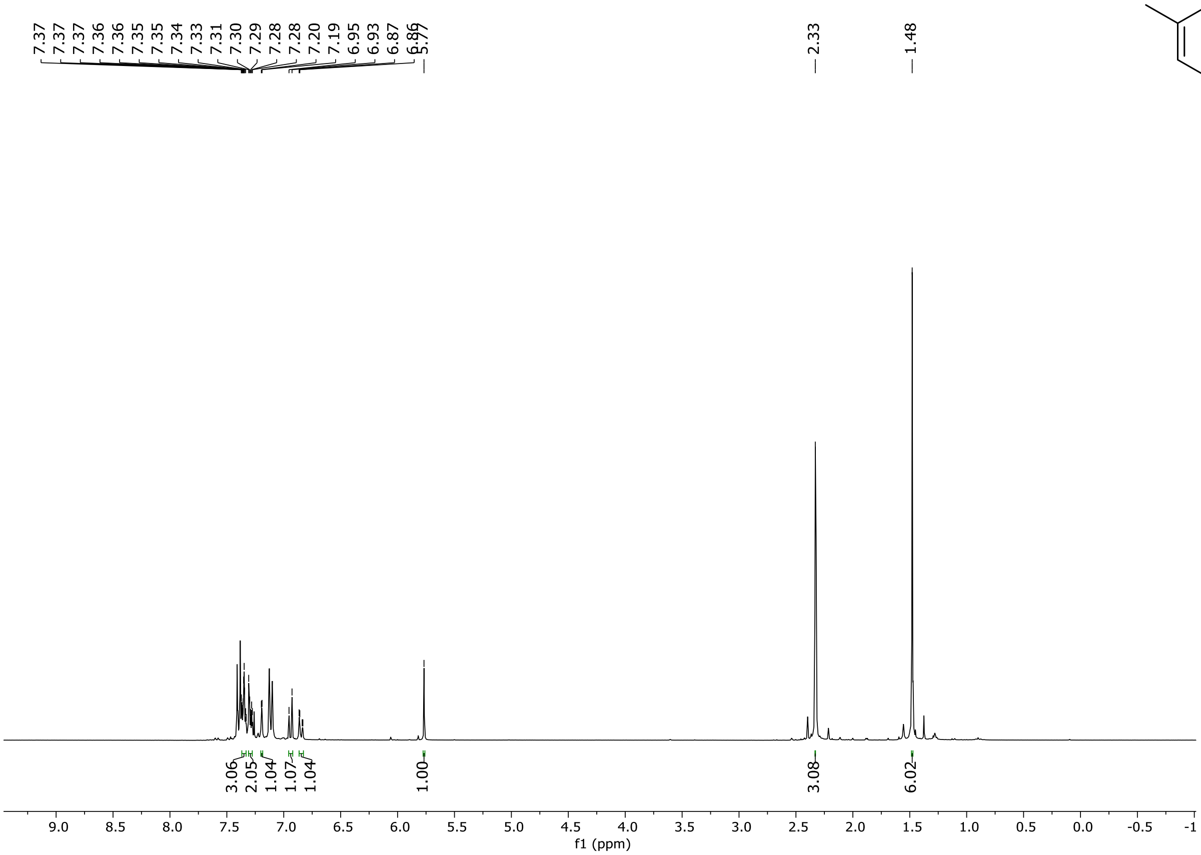

$^{13}C\{^{1}H\}$ NMR (75.4 MHz, $CDCl_3$)

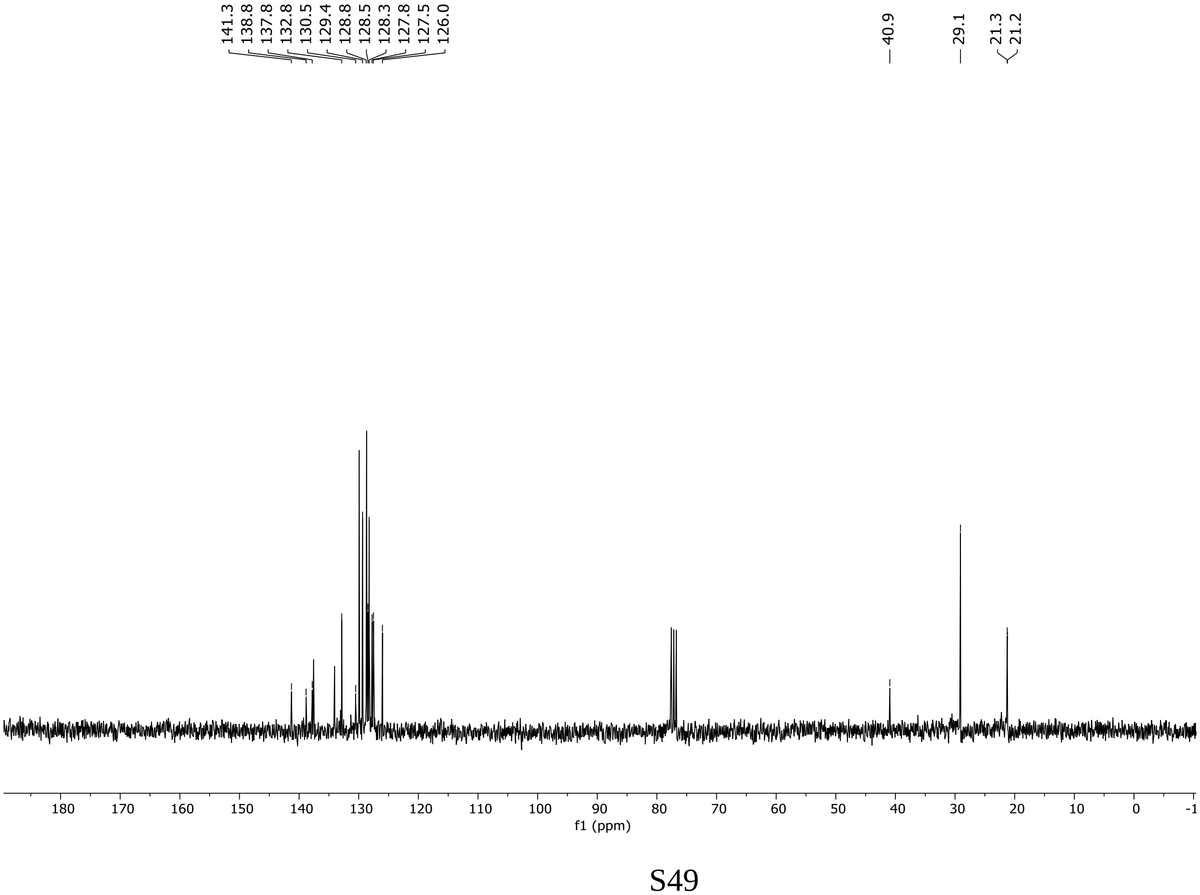

8ab: ^{1}H-NMR (300 MHz, $CDCl_3$)

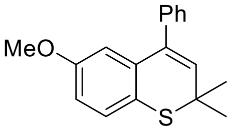

8ab

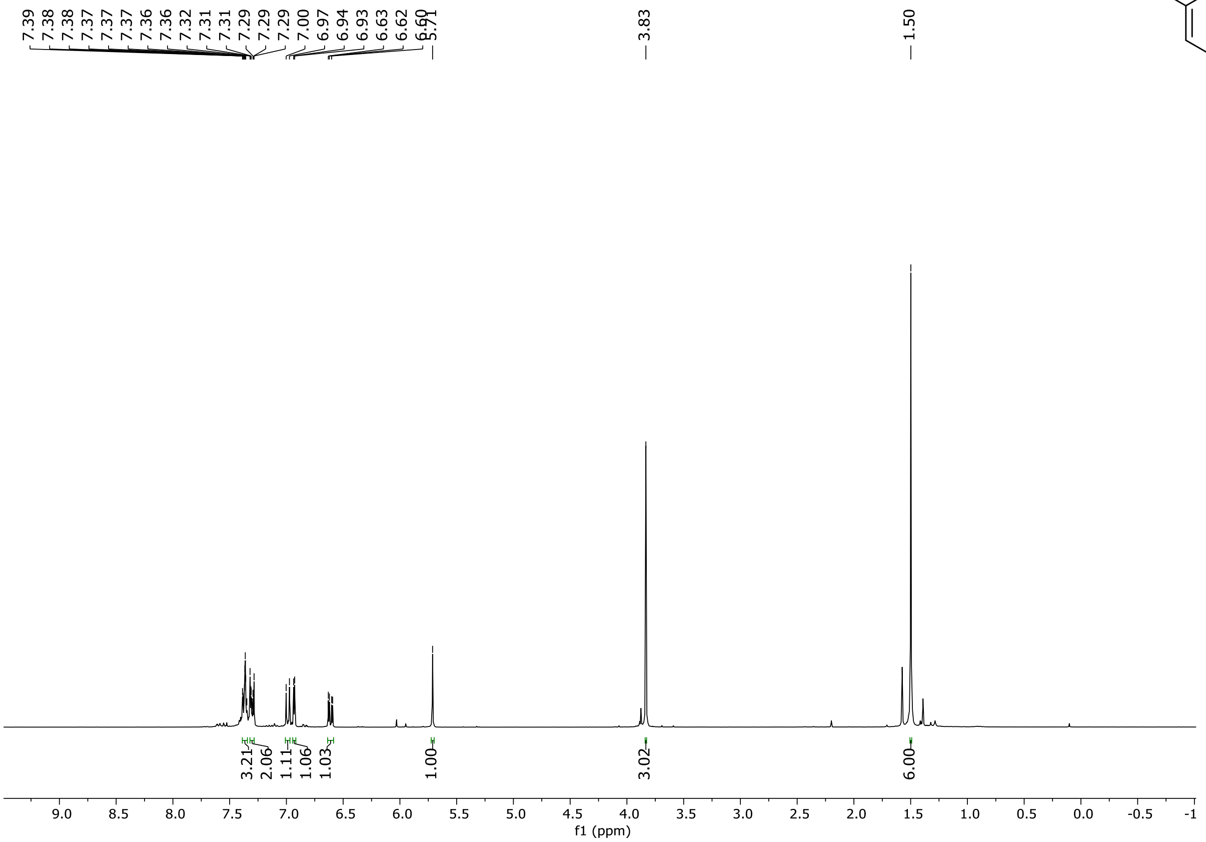

$^{13}C\{^{1}H\}$ NMR (75.4 MHz, $CDCl_3$)

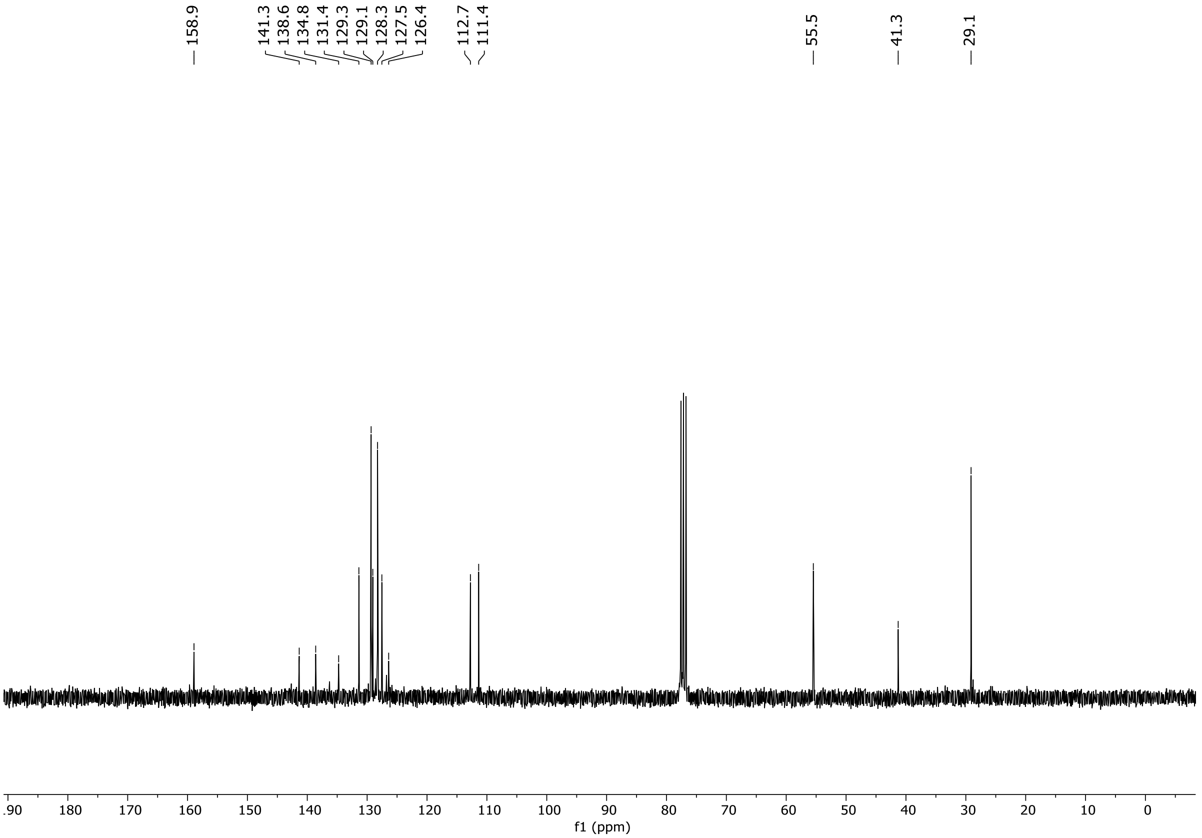

8ah: ^{1}H-NMR (300 MHz, $CDCl_3$)

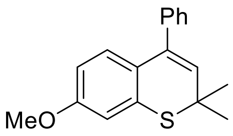

mayor 8ah + minor 8ah´

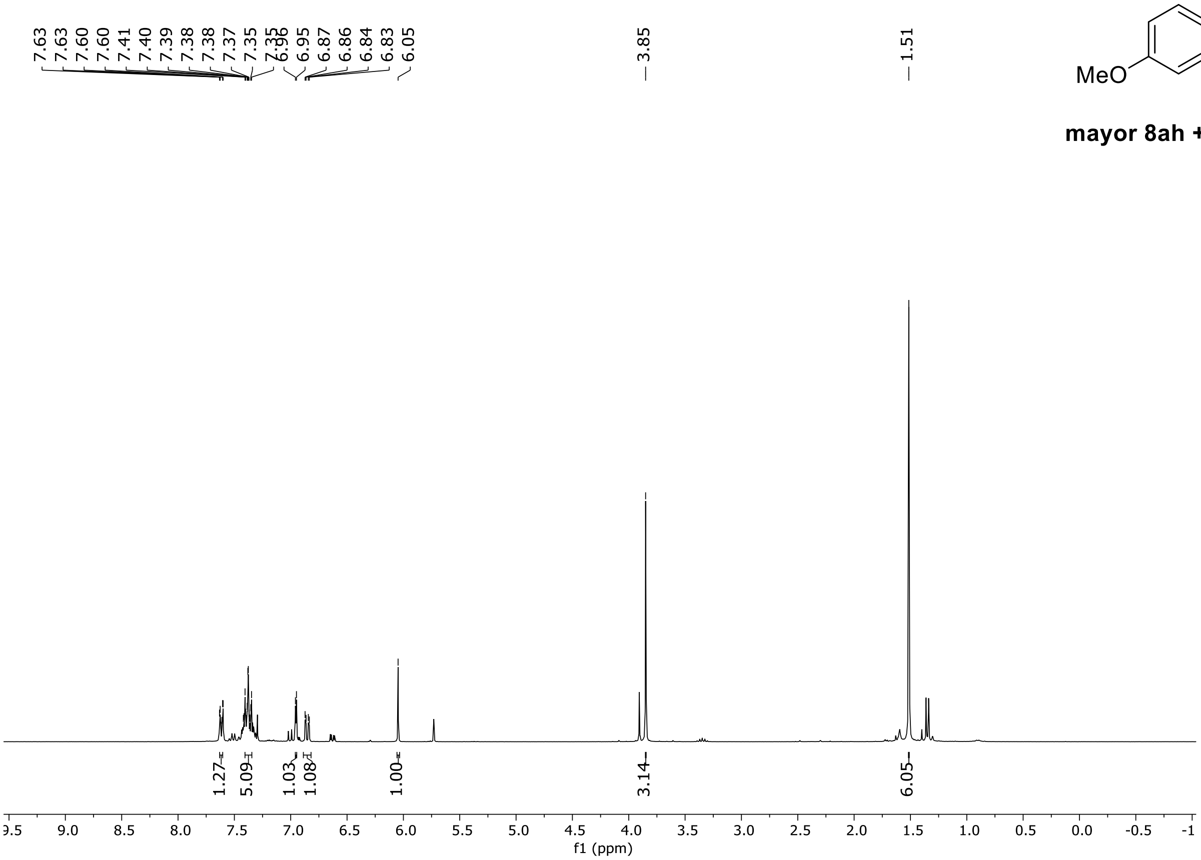

^{13}C{^{1}H} NMR (75.4 MHz, $CDCl_3$)

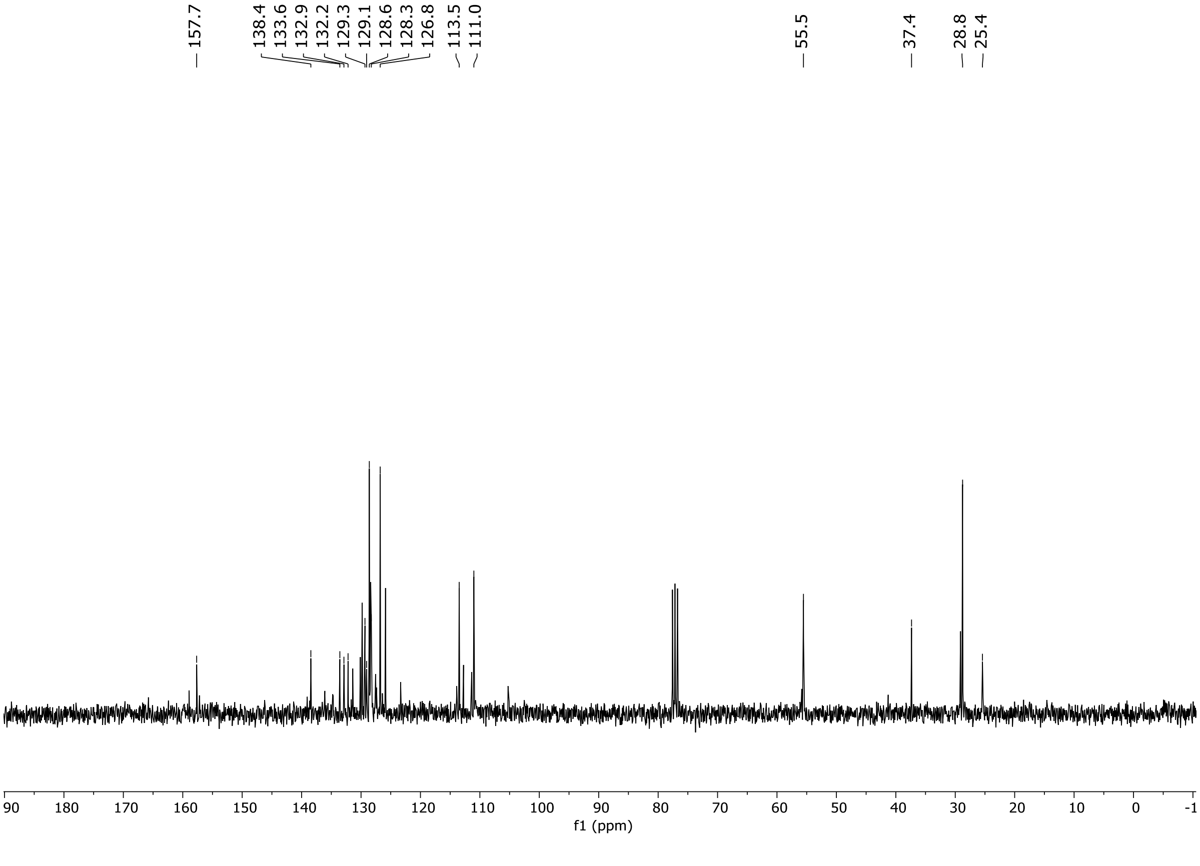

8ah´: ^{1}H-NMR (300 MHz, $CDCl_3$)

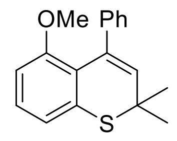

mayor 8ah´ + minor 8ah

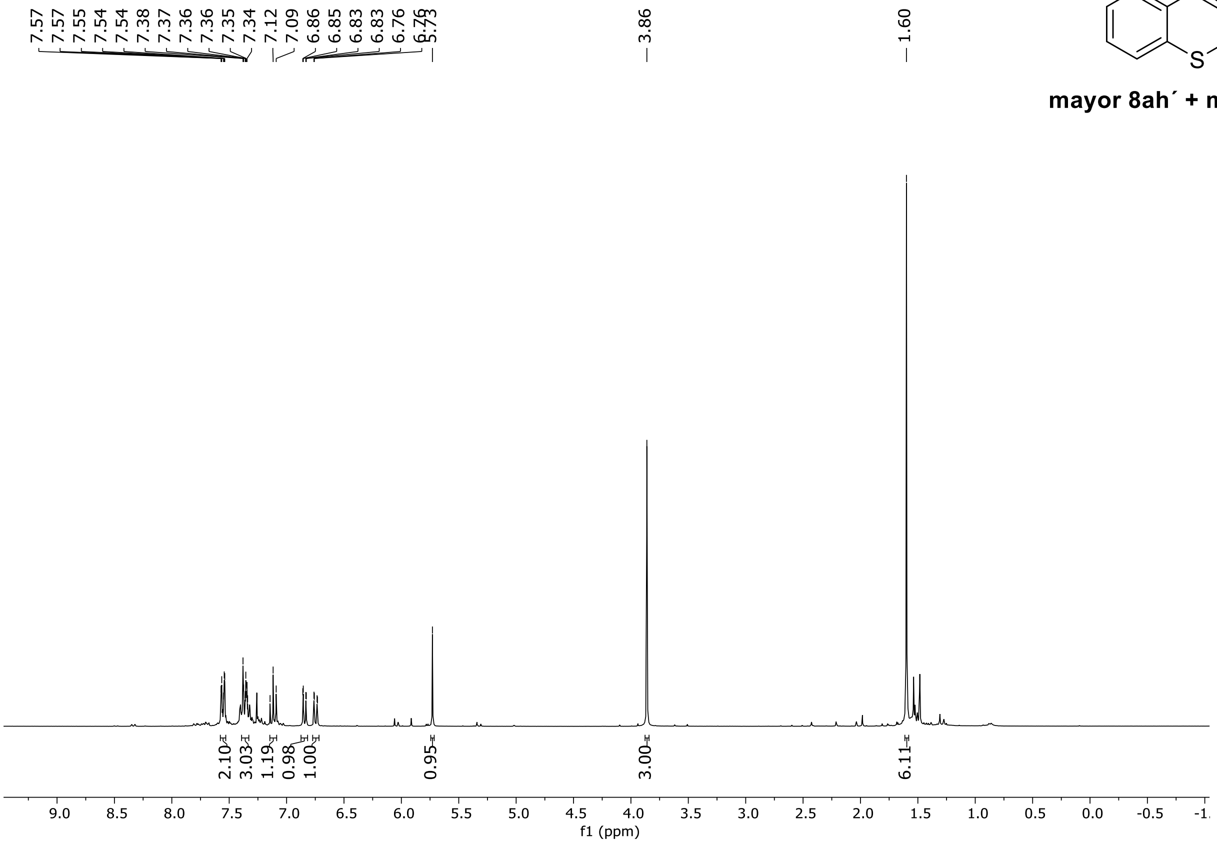

^{13}C{^{1}H} NMR (75.4 MHz, $CDCl_3$)

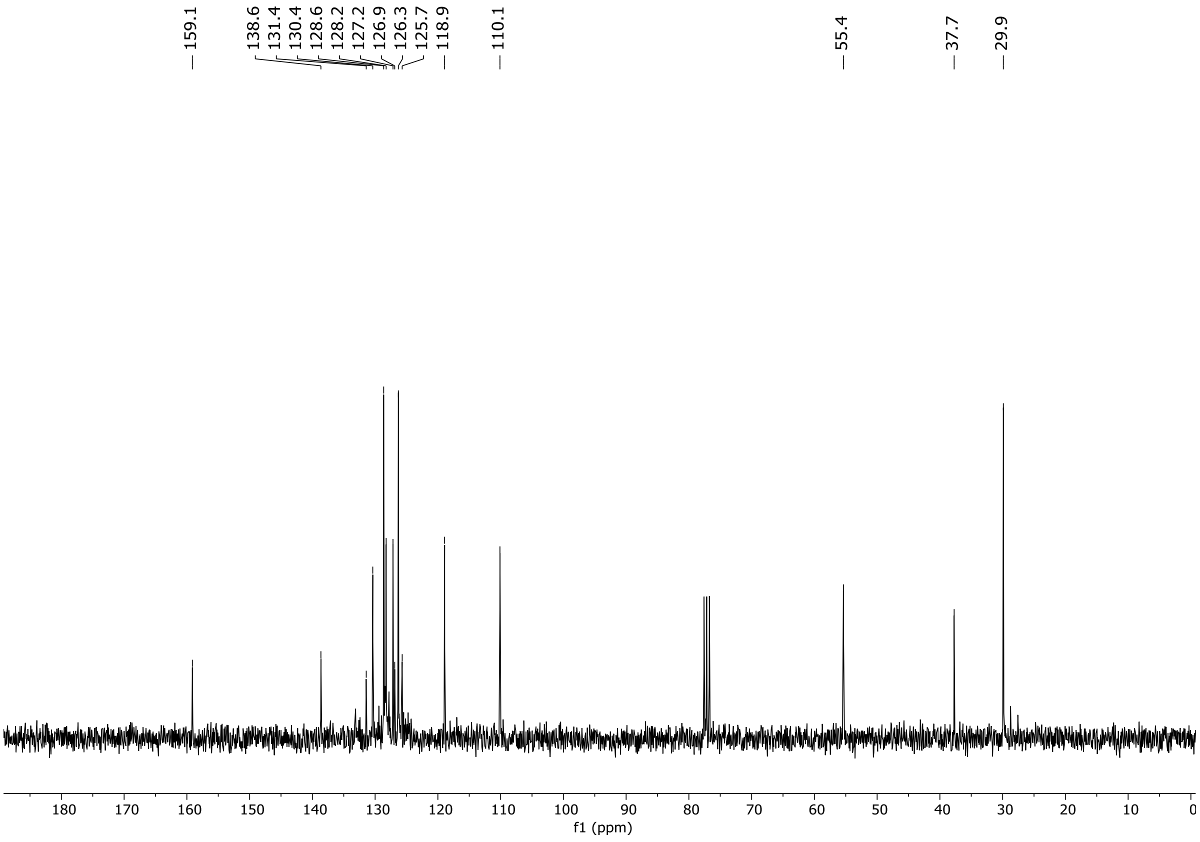

8ai: ^{1}H-NMR (300 MHz, $CDCl_3$)

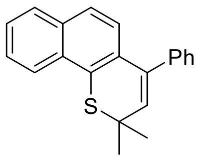

8ai

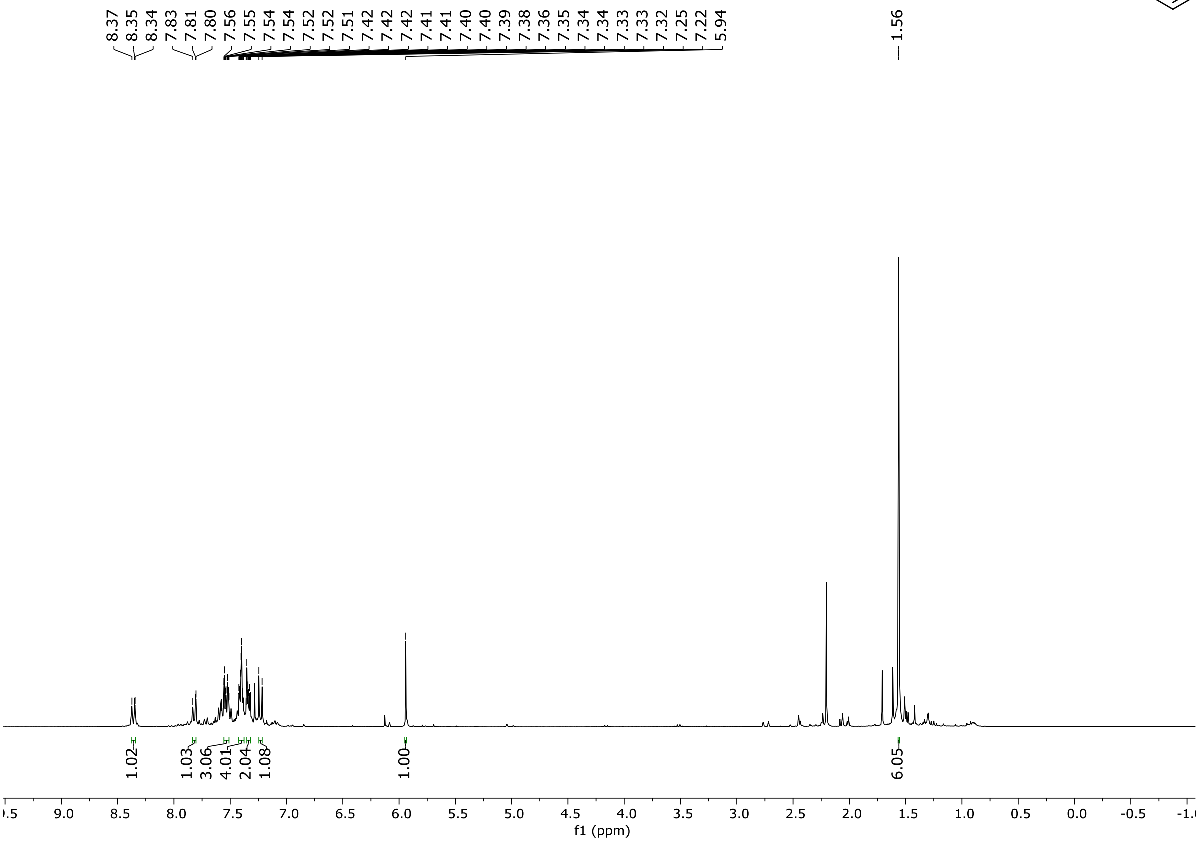

^{13}C{^{1}H} NMR (75.4 MHz, $CDCl_3$)

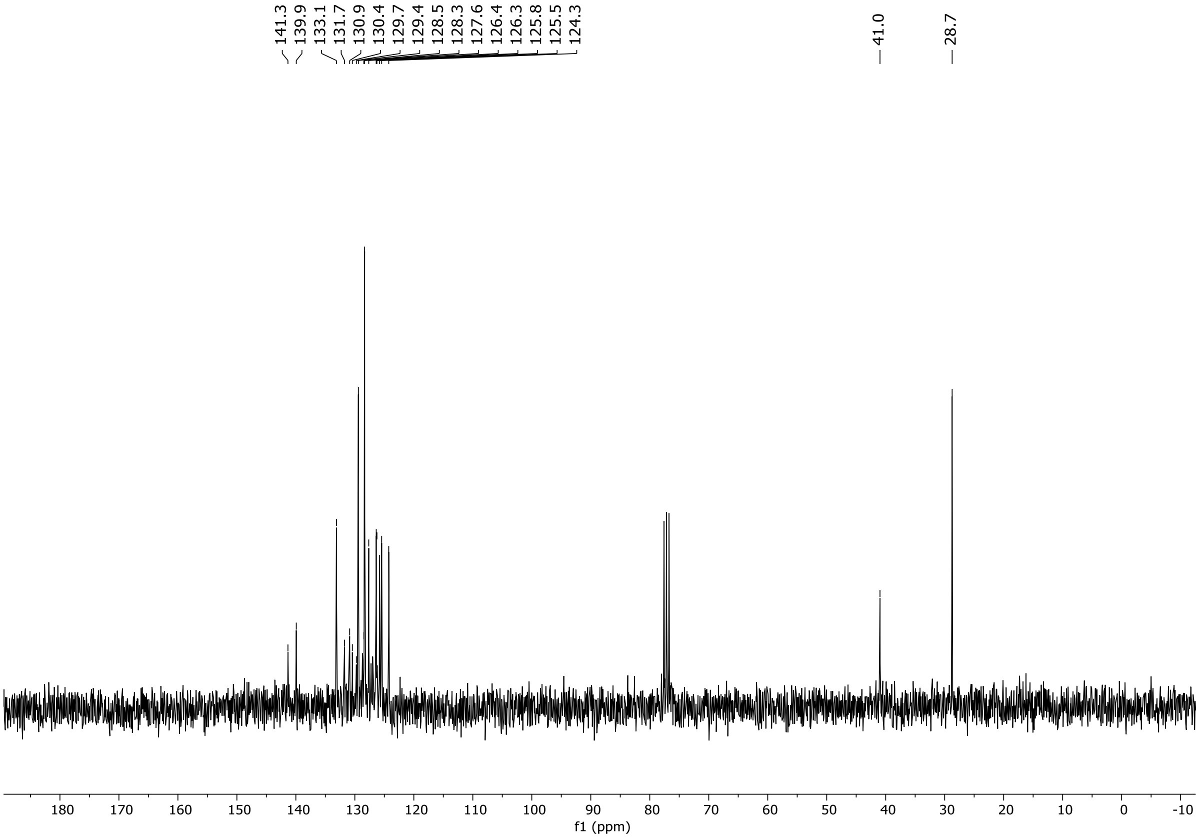

8aj: ^{1}H-NMR (300 MHz, $CDCl_3$)

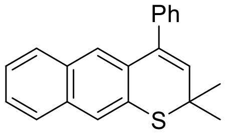

mayor 8aj
+
minor 8aj´

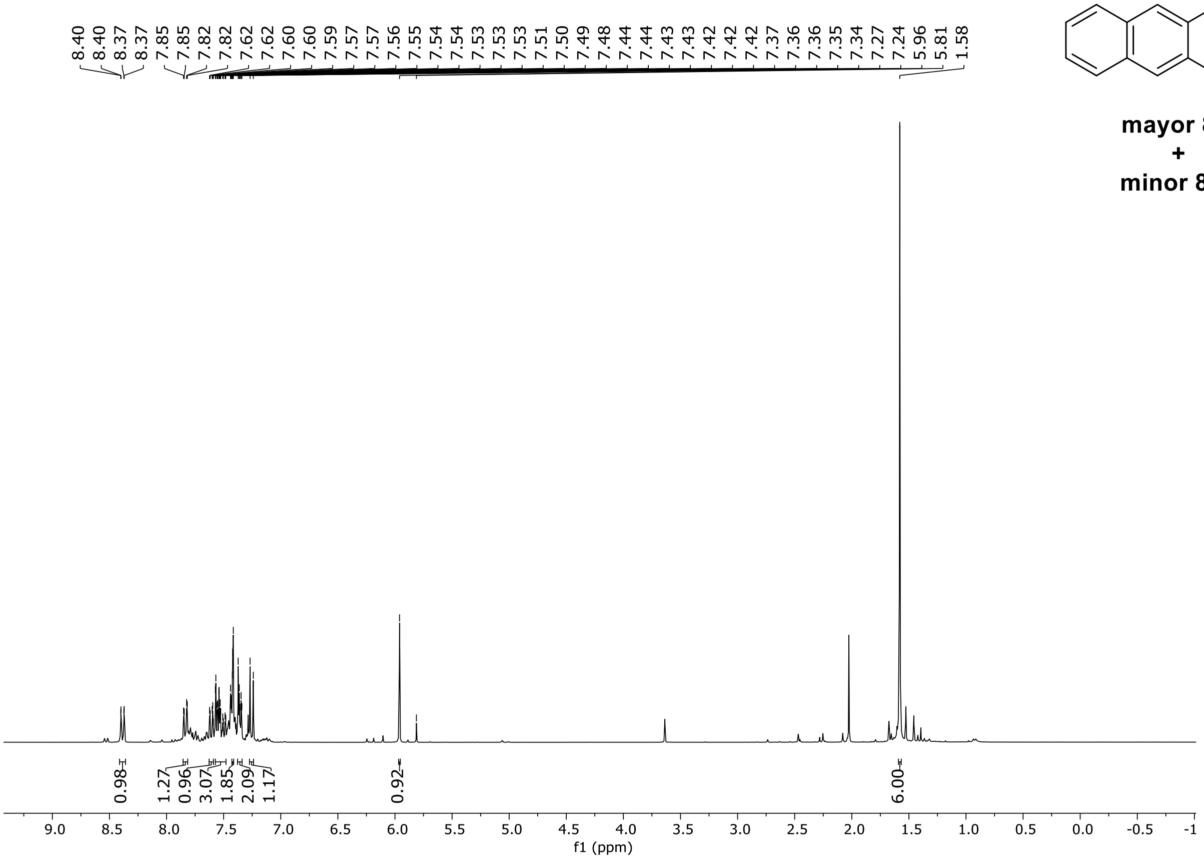

^{13}C{^{1}H} NMR (75.4 MHz, $CDCl_3$)

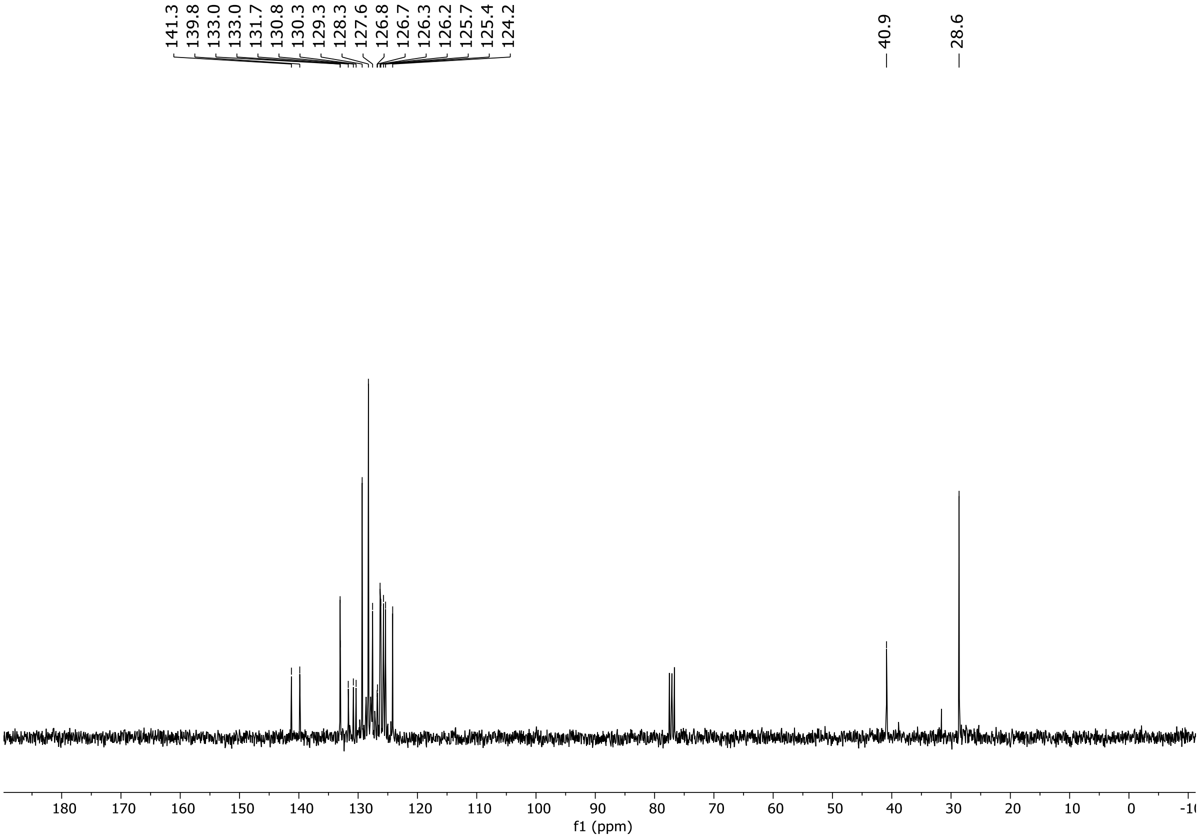

8aj´: ^{1}H-NMR (300 MHz, $CDCl_3$)

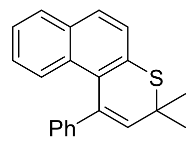

mayor 8aj´
+
minor 8aj

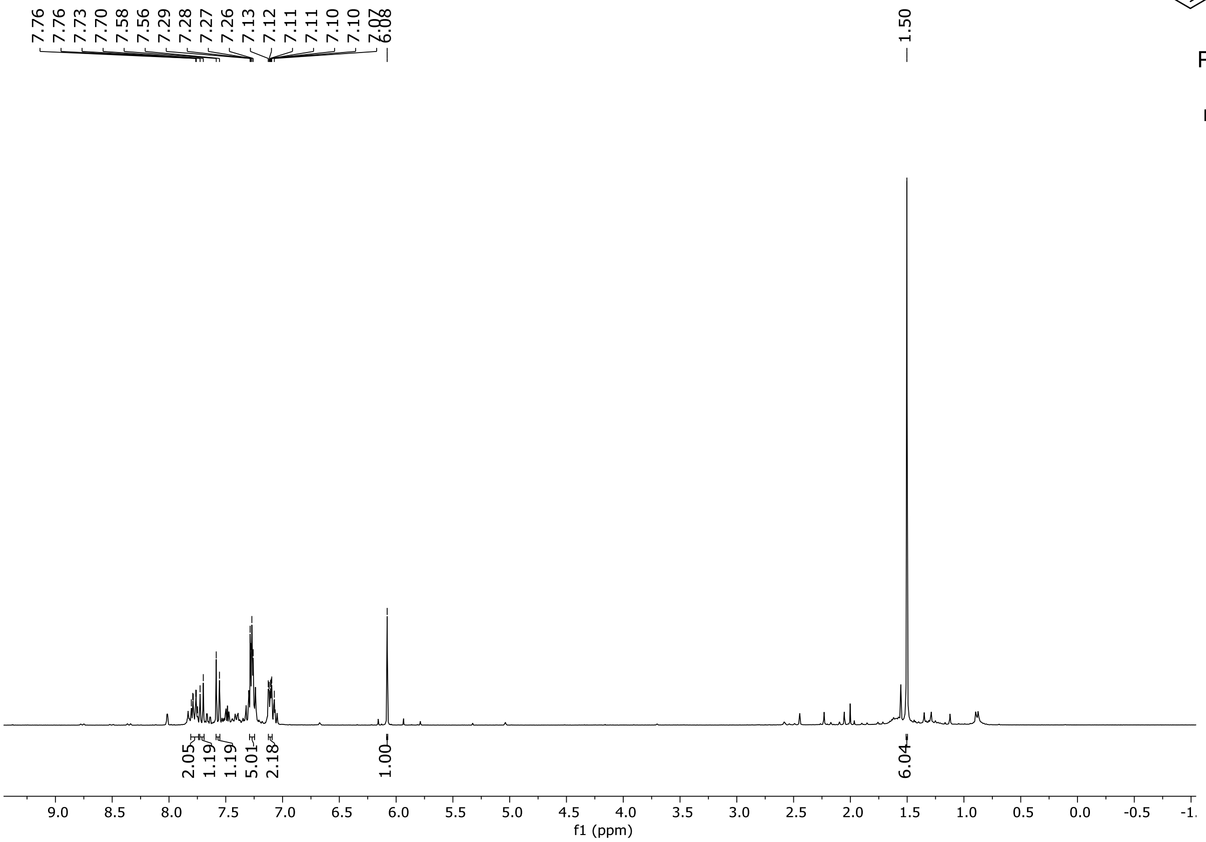

$^{13}C\{^1H\}$ NMR (75.4 MHz, $CDCl_3$)

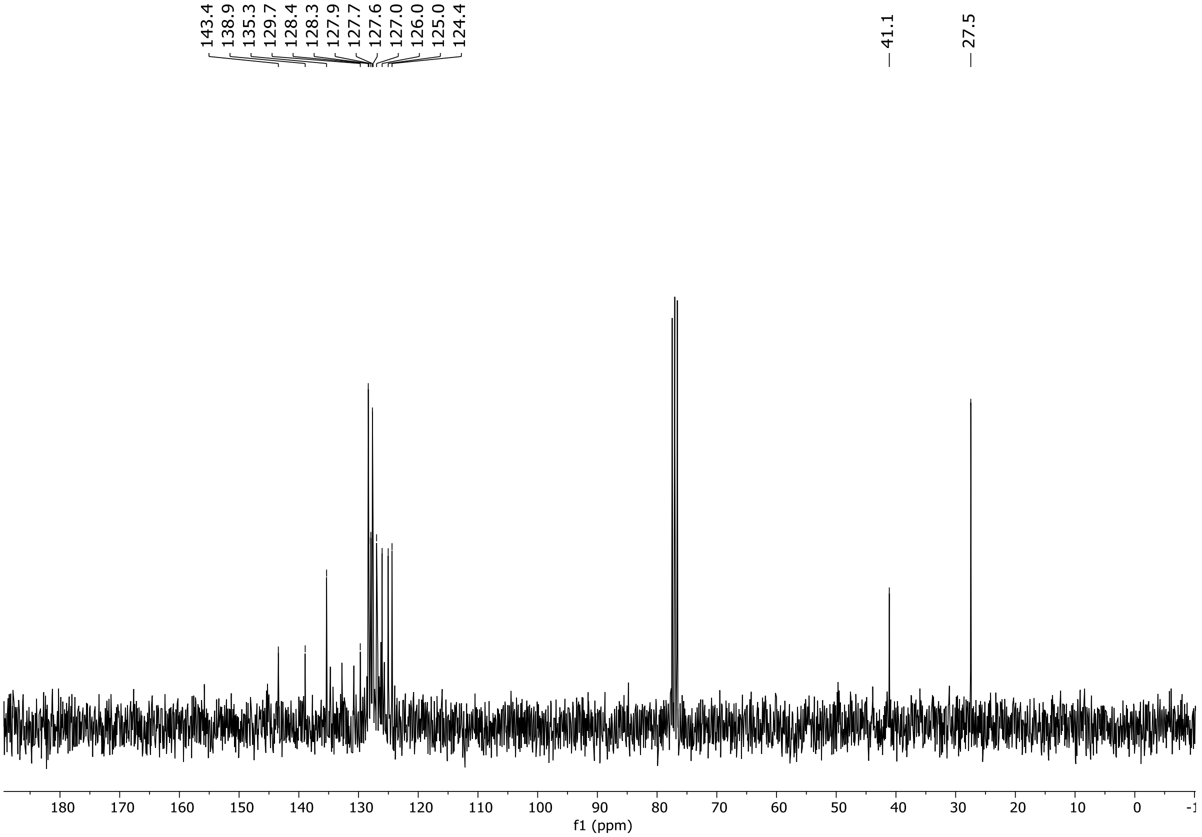

8ha: ^{1}H-NMR (300 MHz, $CDCl_3$)

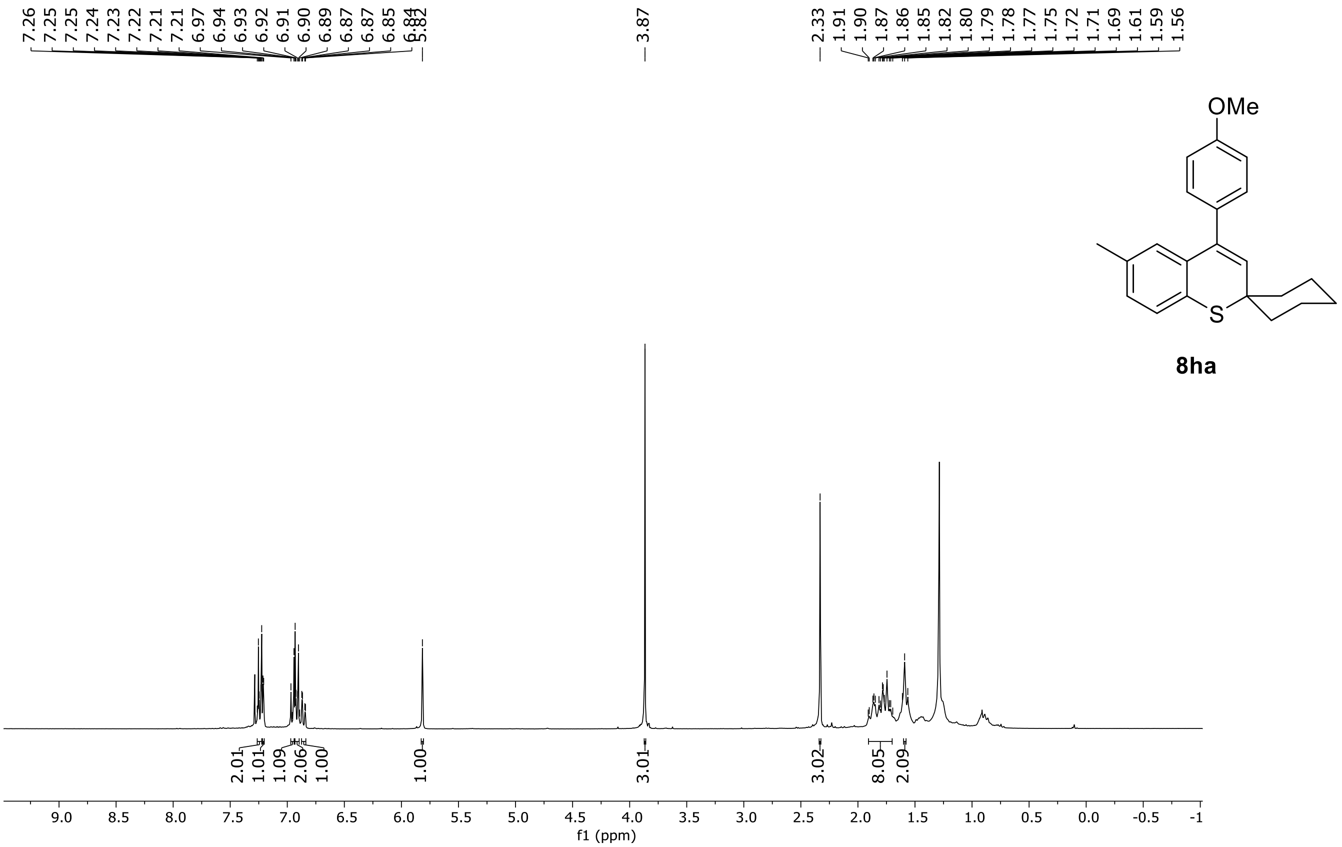

^{13}C{^{1}H} NMR (75.4 MHz, $CDCl_3$)

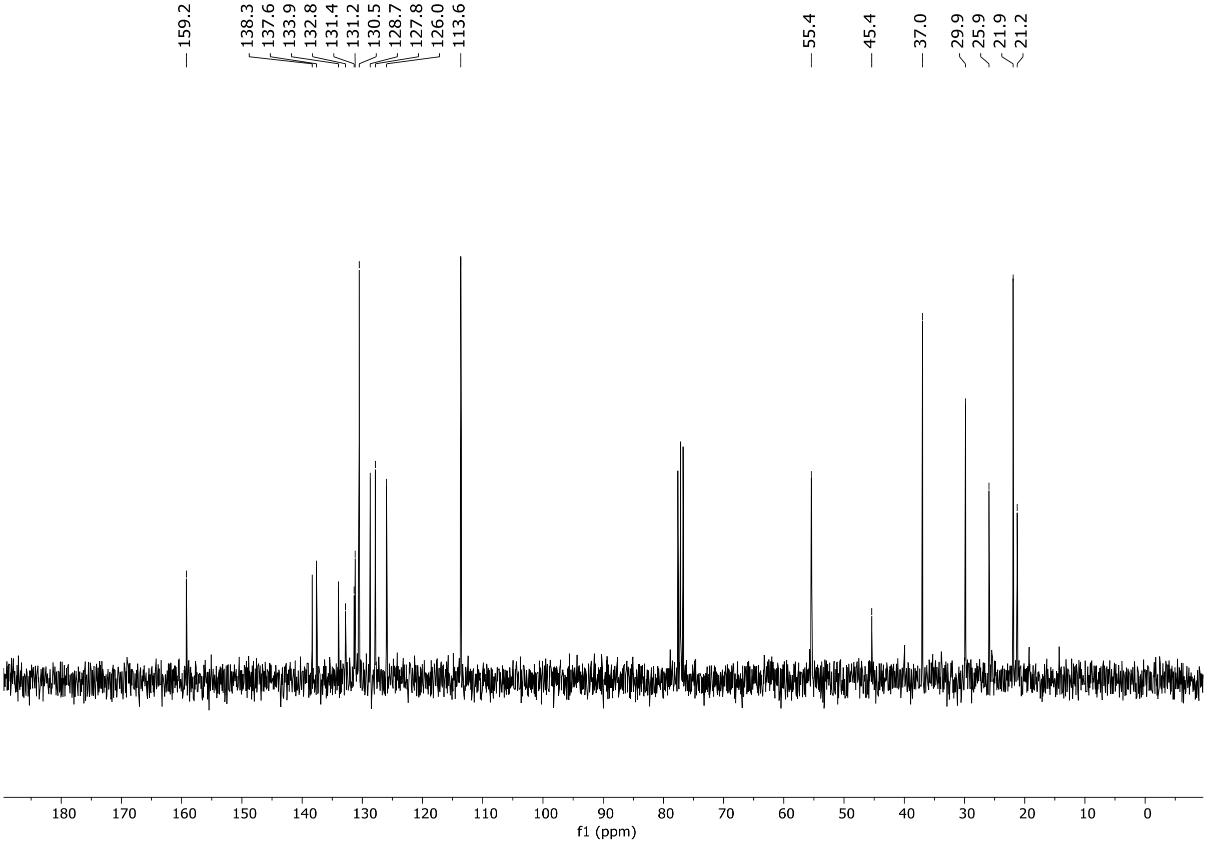

8ja: ^{1}H-NMR (300 MHz, $CDCl_3$)

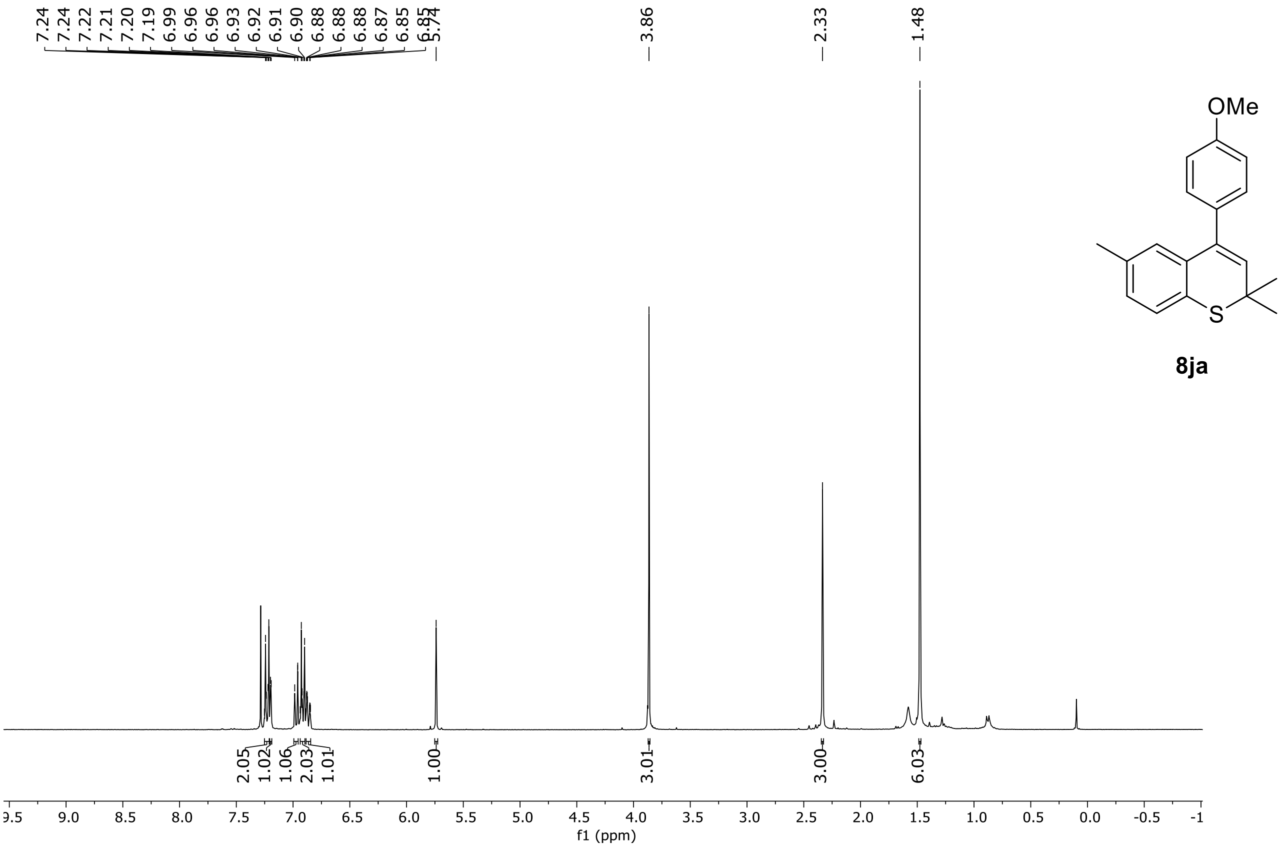

8ja

^{13}C{^{1}H} NMR (75.4 MHz, $CDCl_3$)

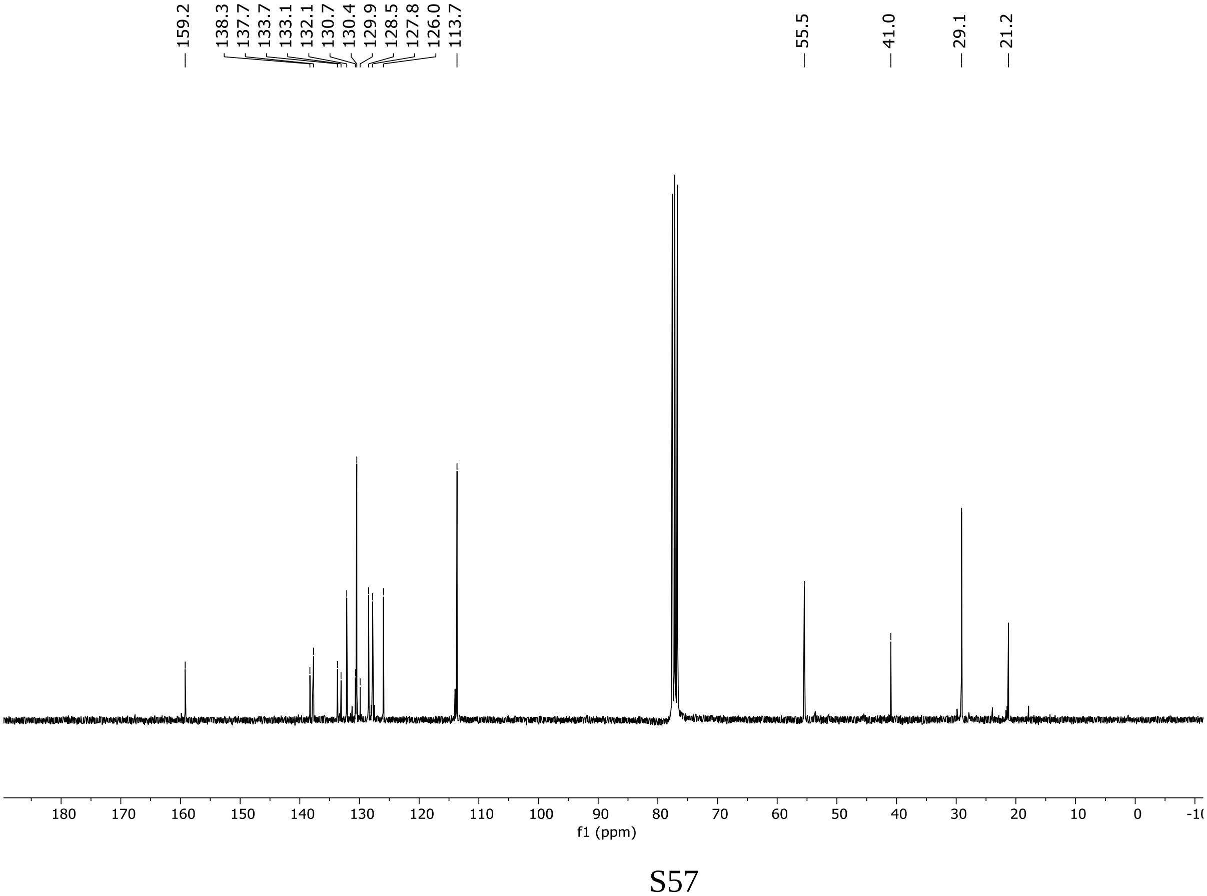

8jk: ^{1}H-NMR (300 MHz, $CDCl_3$)

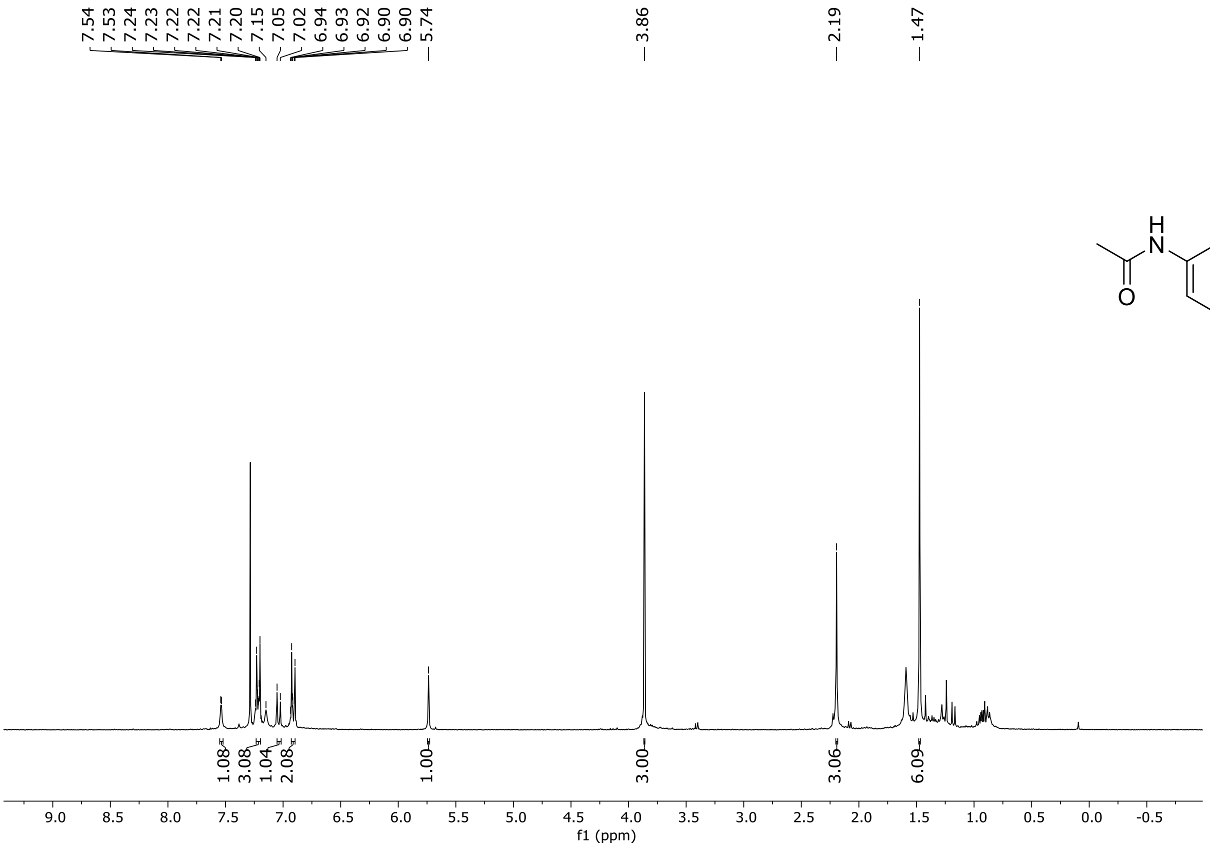

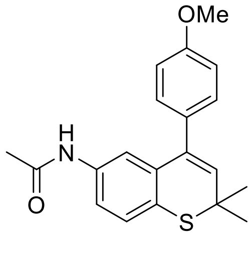

8jk

^{13}C{^{1}H} NMR (75.4 MHz, $CDCl_3$)

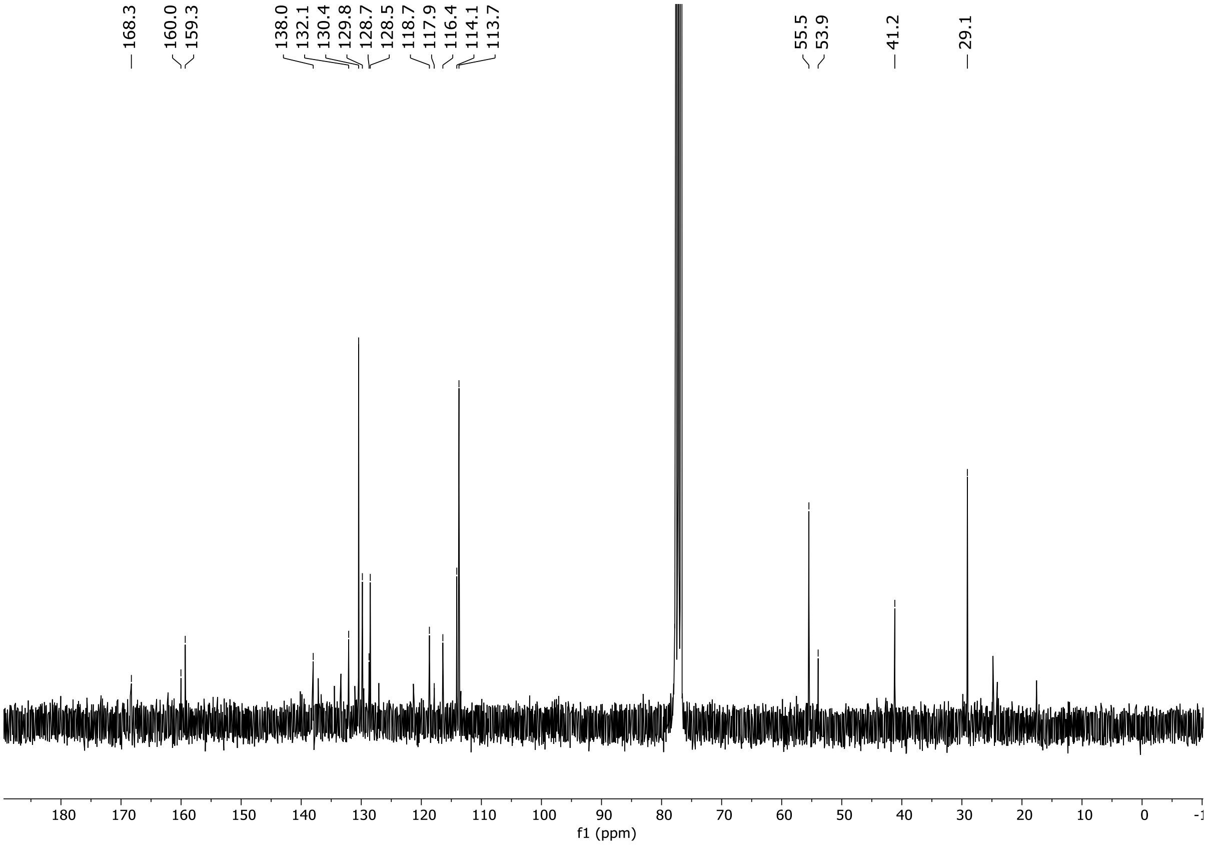

11: ^{1}H-NMR (300 MHz, $CDCl_3$)

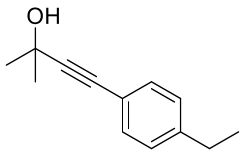

11

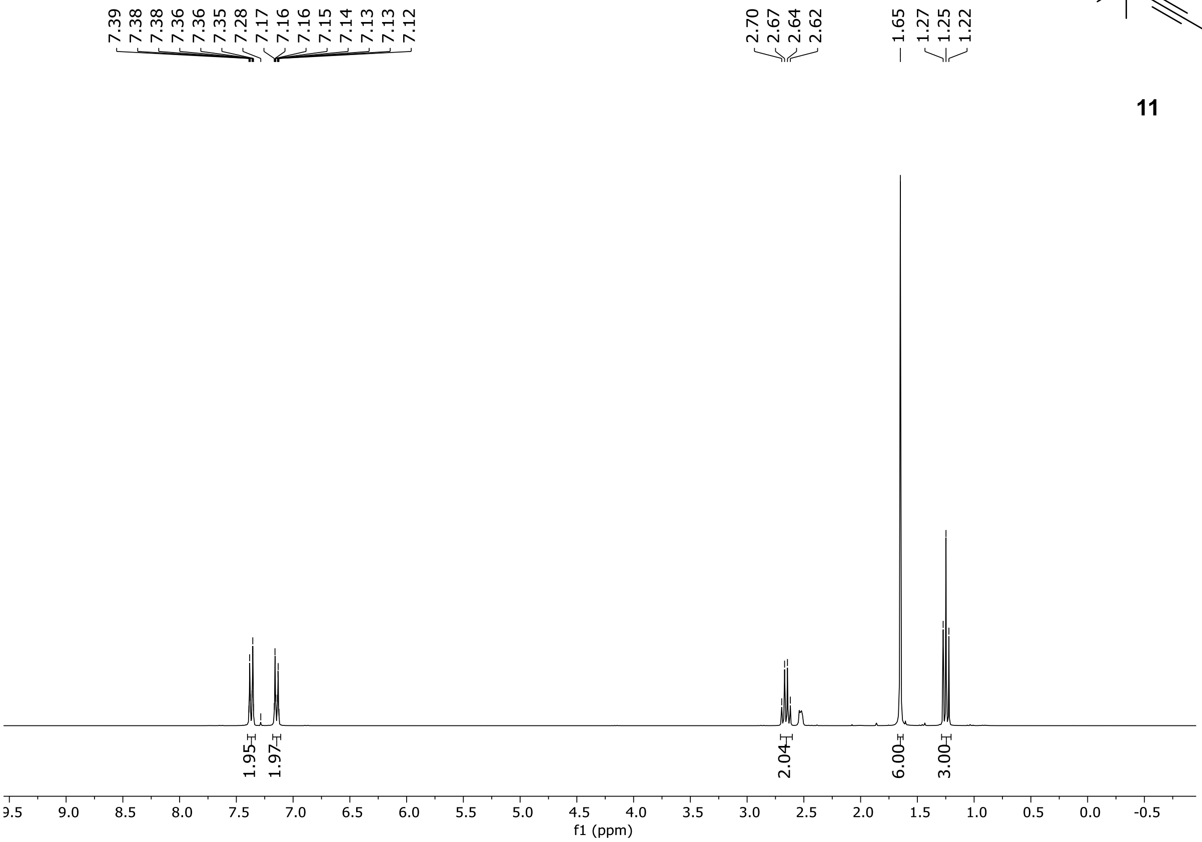

^{13}C{^{1}H} NMR (75.4 MHz, $CDCl_3$)

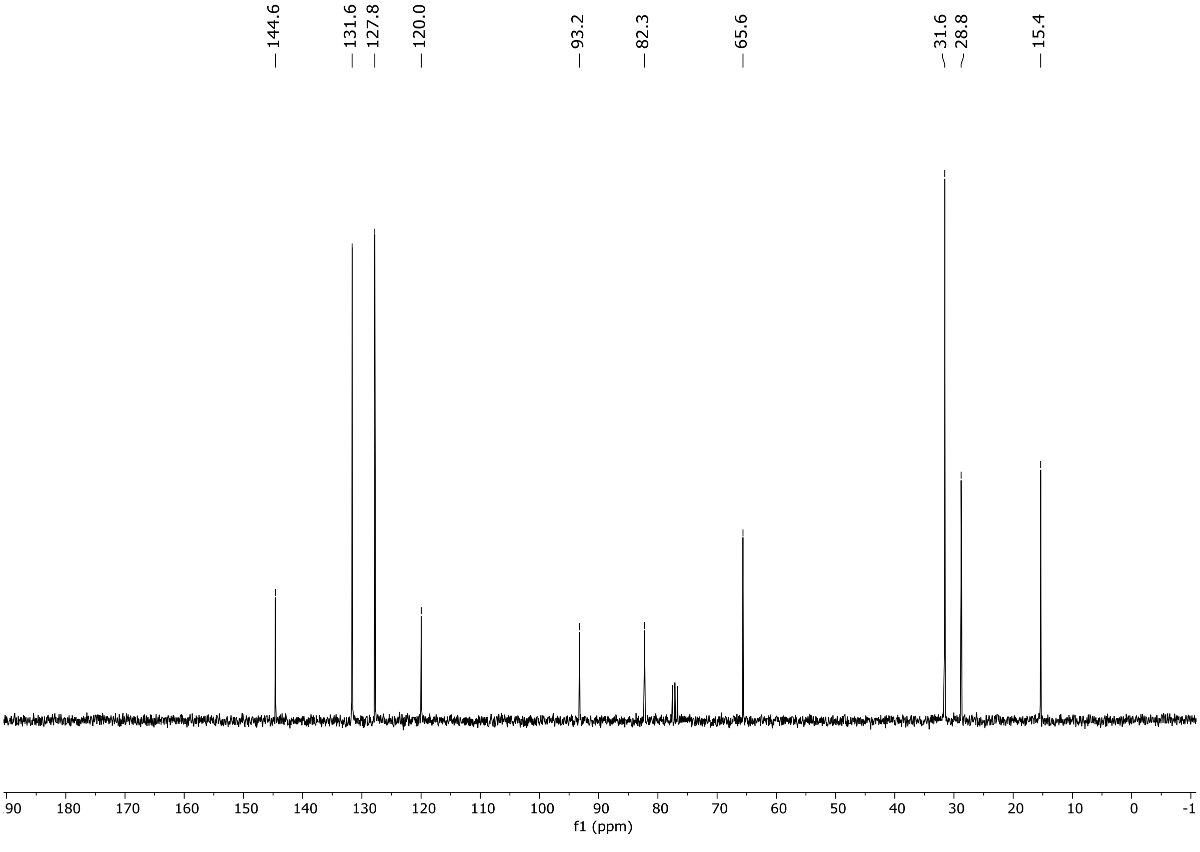

12: ^{1}H-NMR (300 MHz, $CDCl_3$)

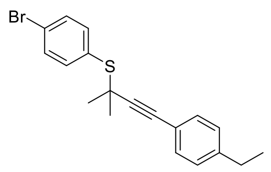

12

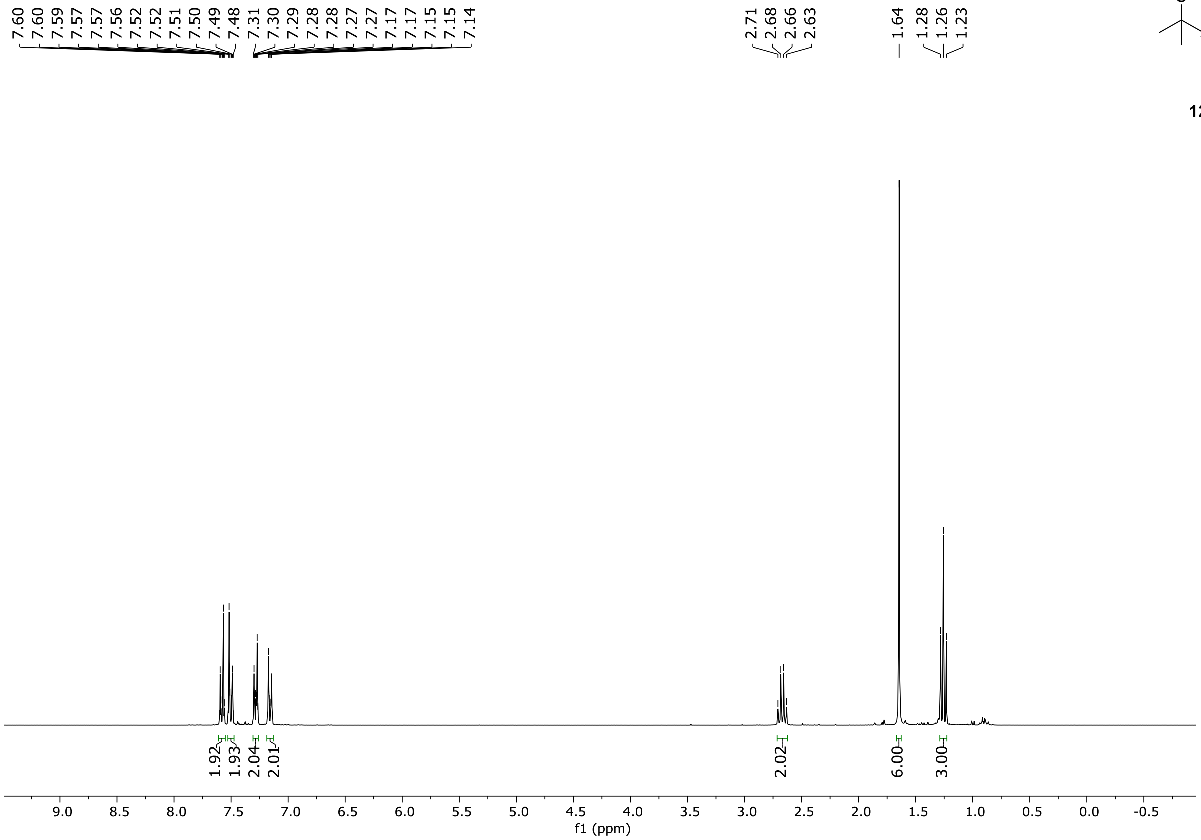

$^{13}C\{^{1}H\}$ NMR (75.4 MHz, $CDCl_3$)

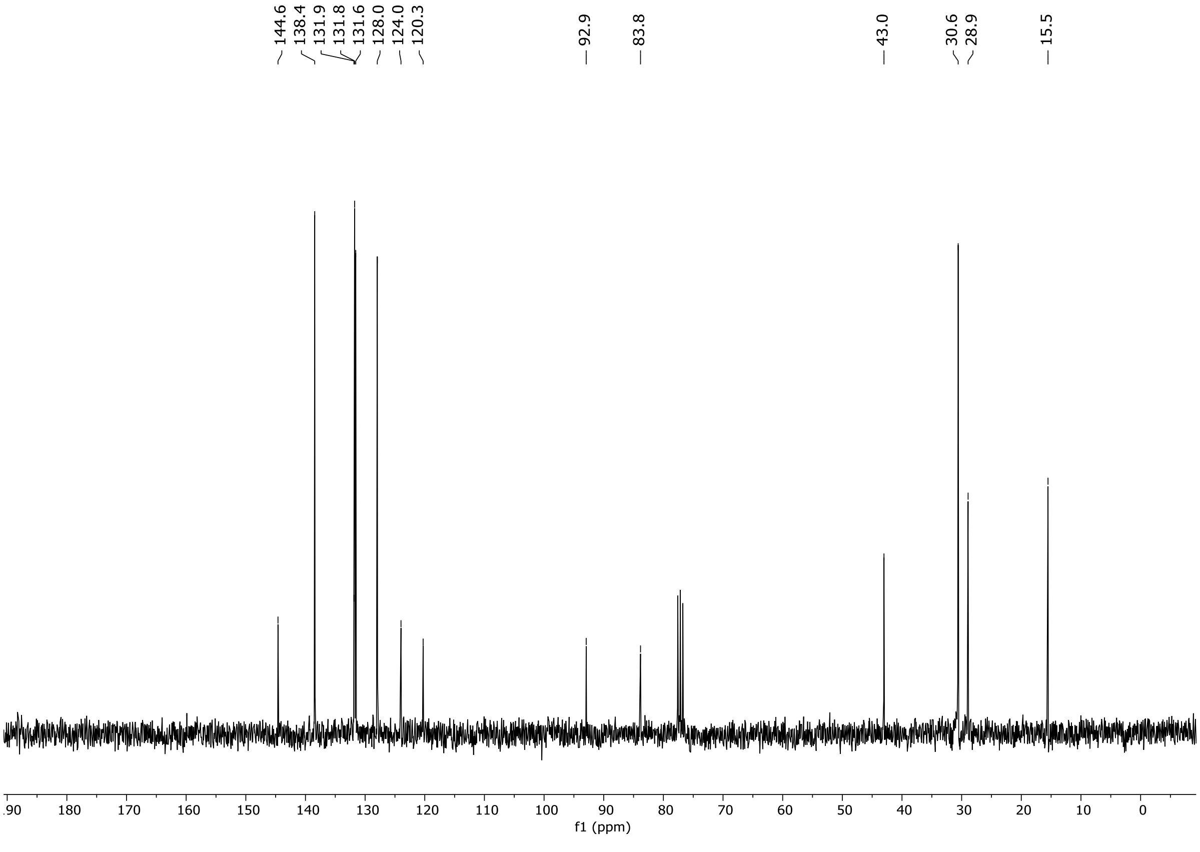

S13: ^{1}H-NMR (300 MHz, $CDCl_3$)

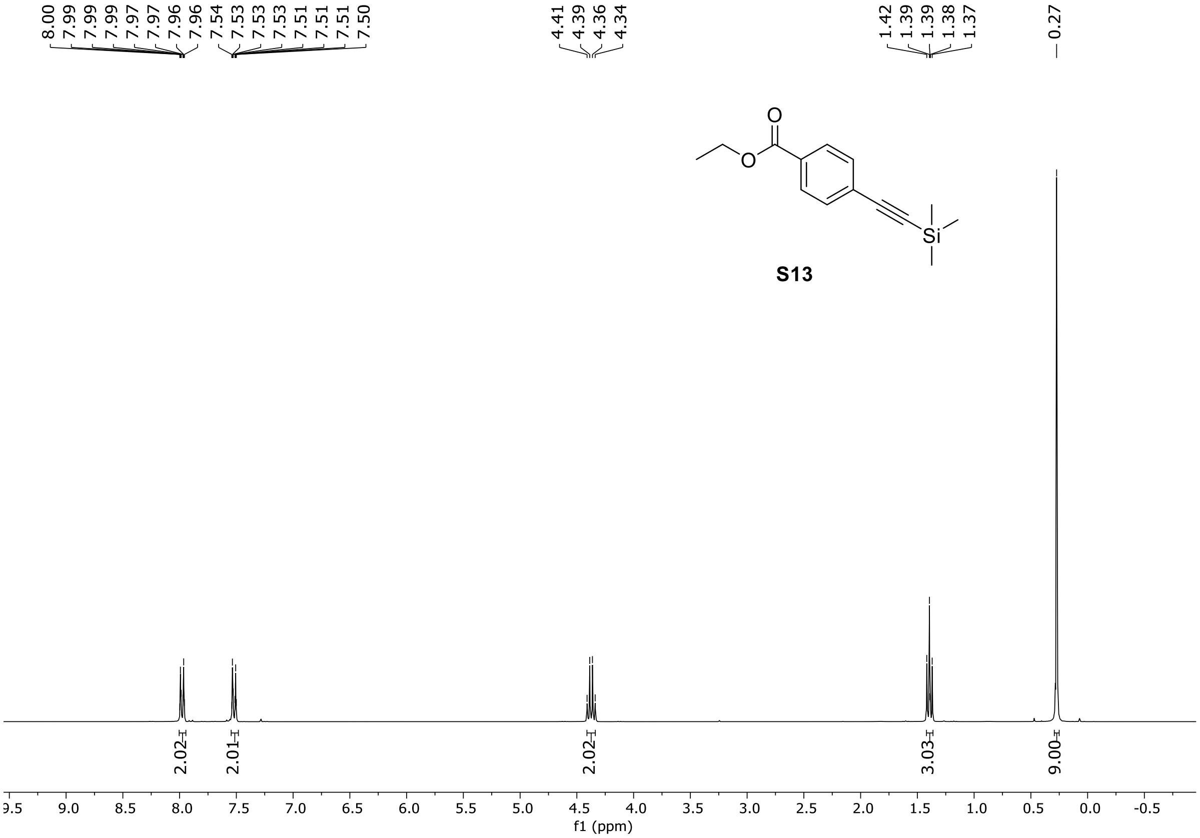

^{13}C{^{1}H} NMR (75.4 MHz, $CDCl_3$)

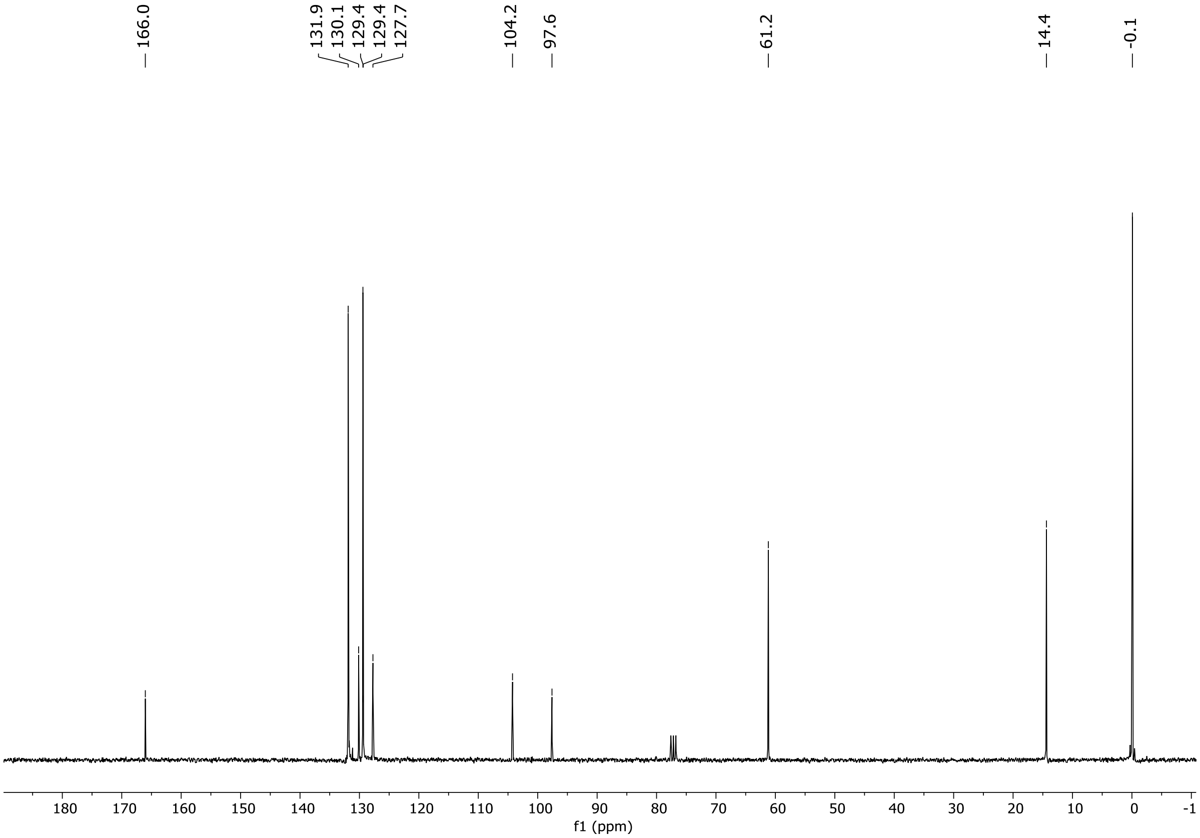

13: ^{1}H-NMR (300 MHz, $CDCl_3$)

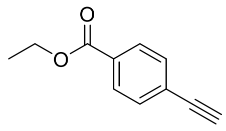

13

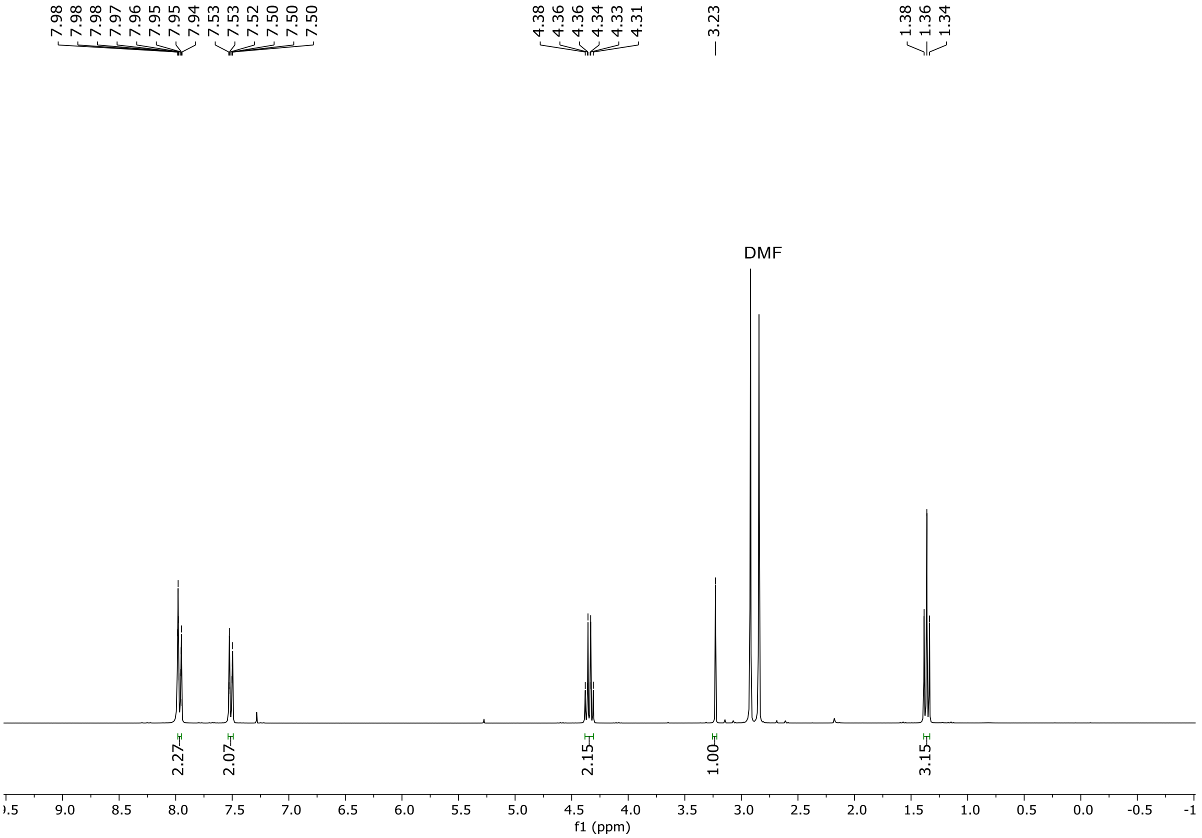

$^{13}C\{^{1}H\}$ NMR (75.4 MHz, $CDCl_3$)

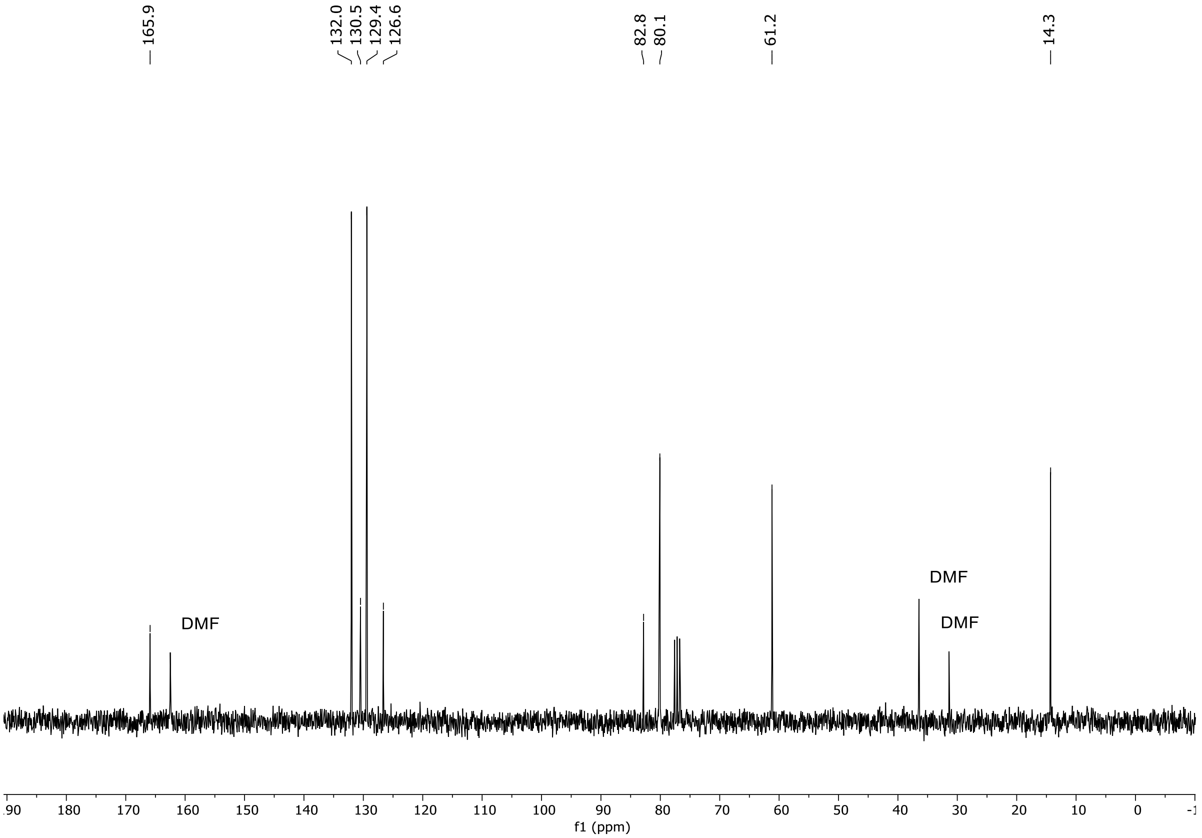

14: ^{1}H-NMR (300 MHz, $CDCl_3$)

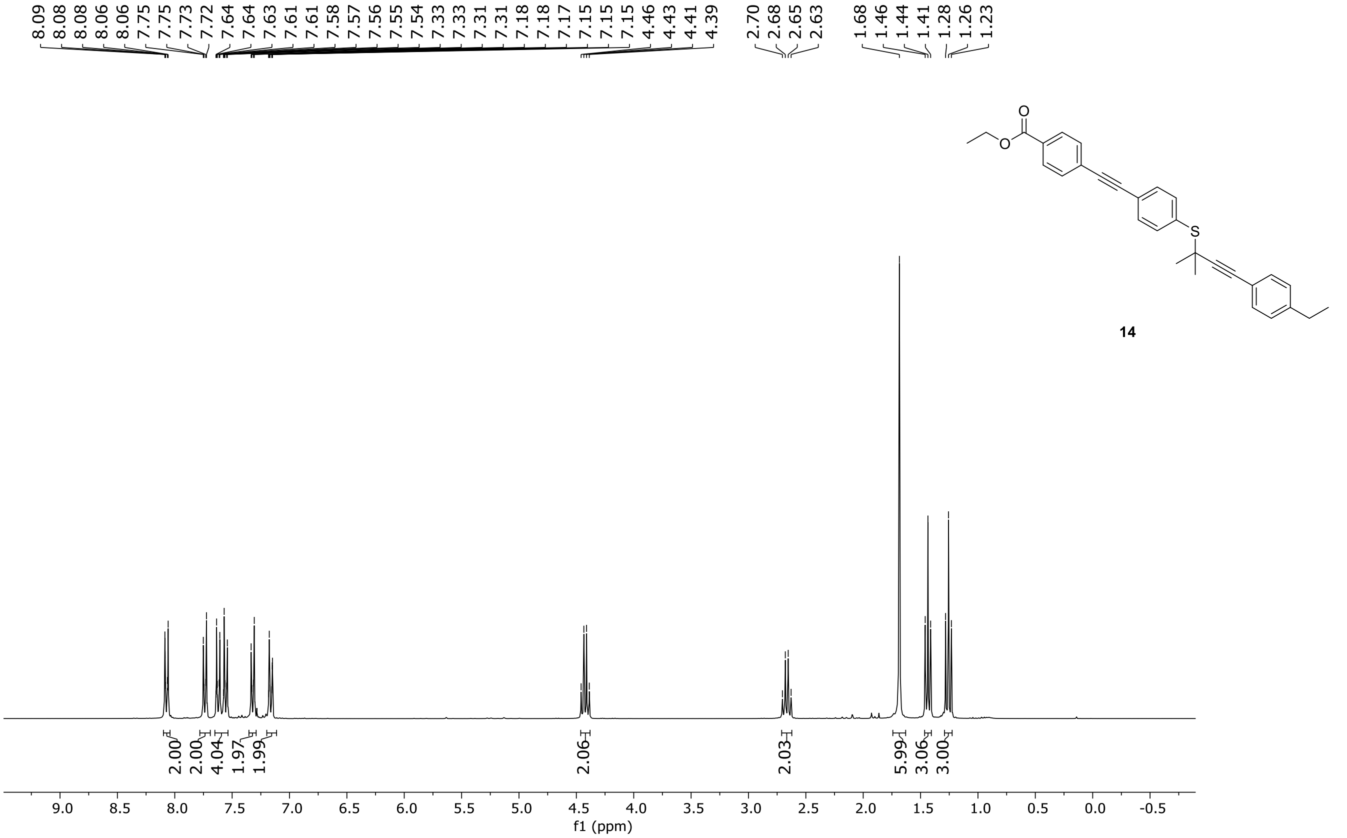

14

$^{13}C\{^1H\}$ NMR (75.4 MHz, $CDCl_3$)

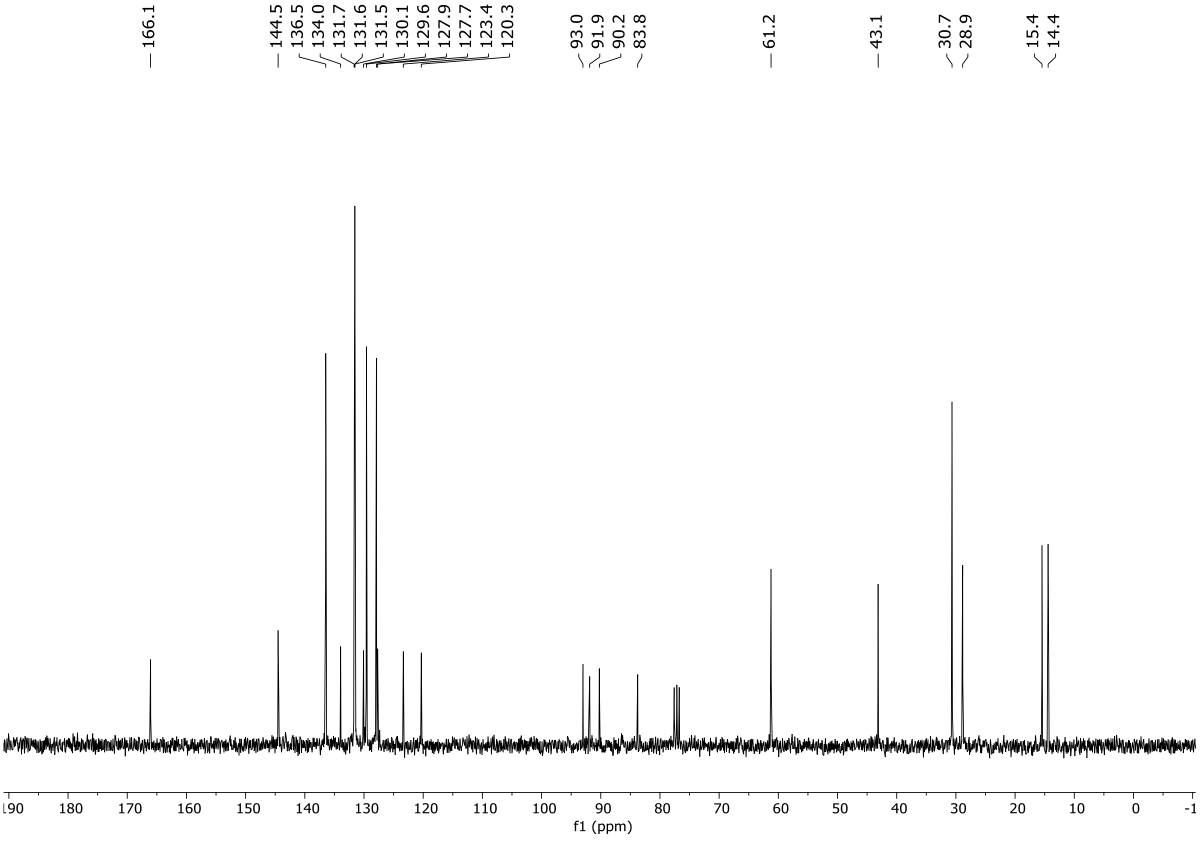

15: ^{1}H-NMR (300 MHz, $CDCl_3$)

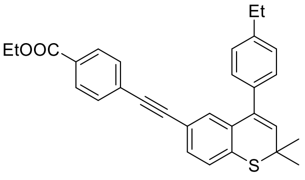

15

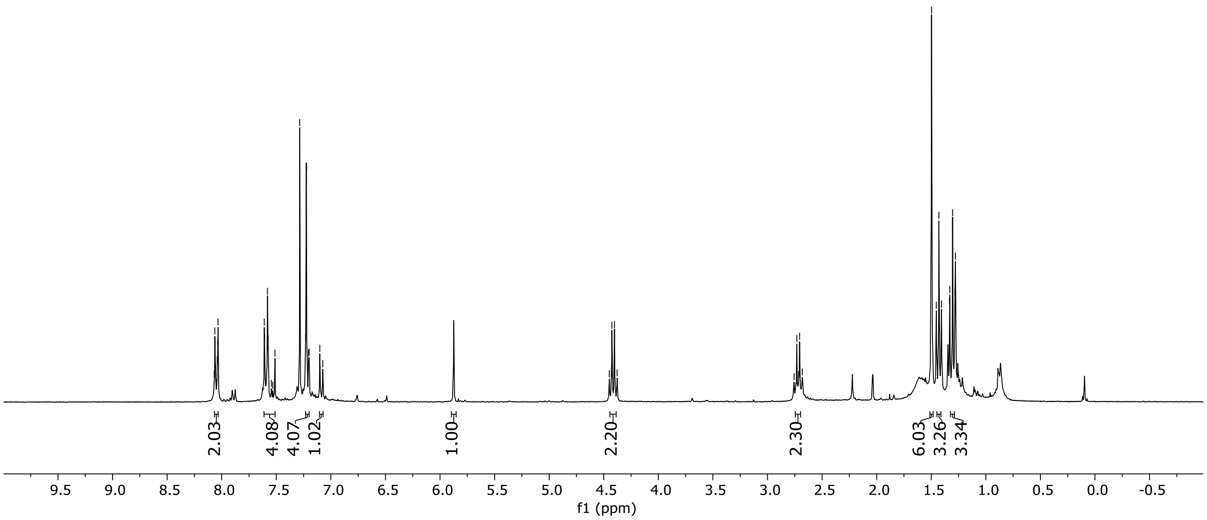

^{13}C{^{1}H} NMR (75.4 MHz, $CDCl_3$)

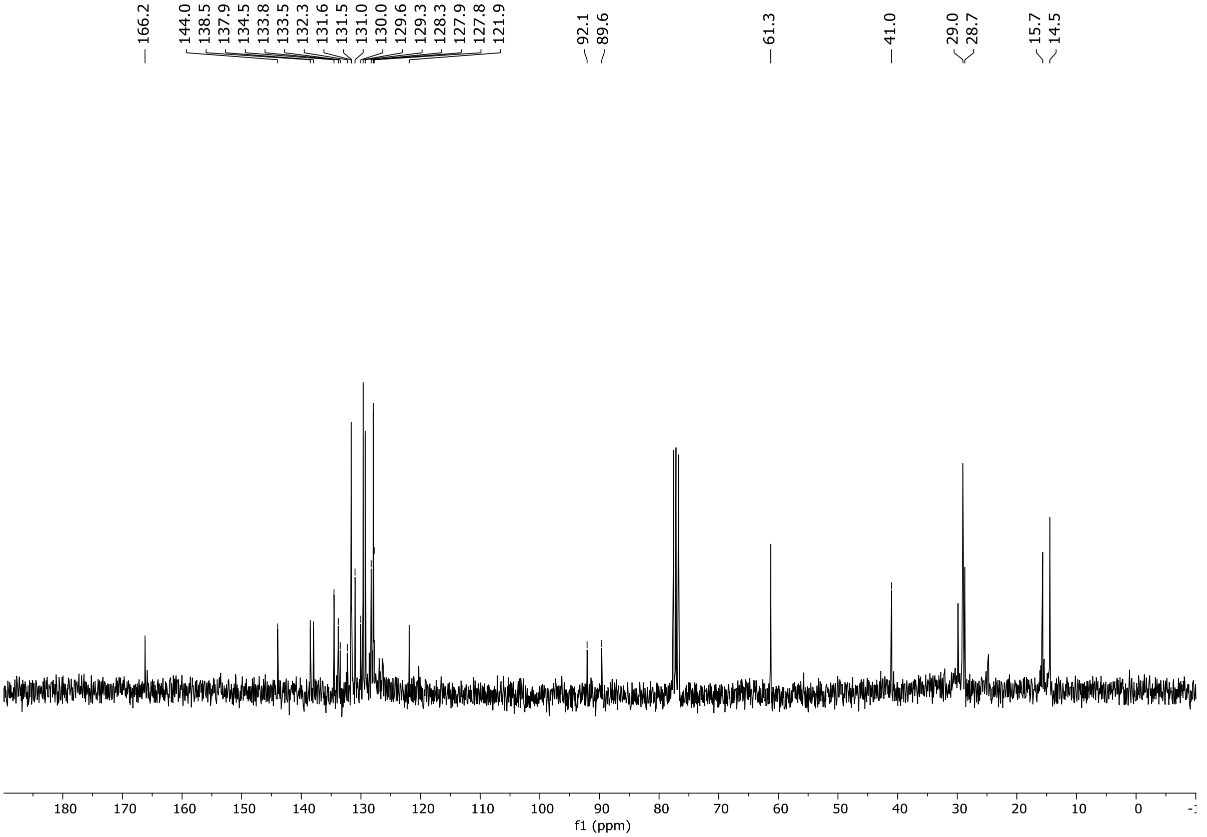

16: ^{1}H-NMR (300 MHz, $(CD_3)_2CO$)

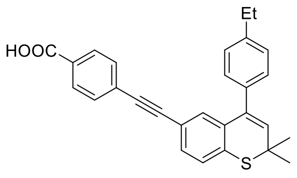

16

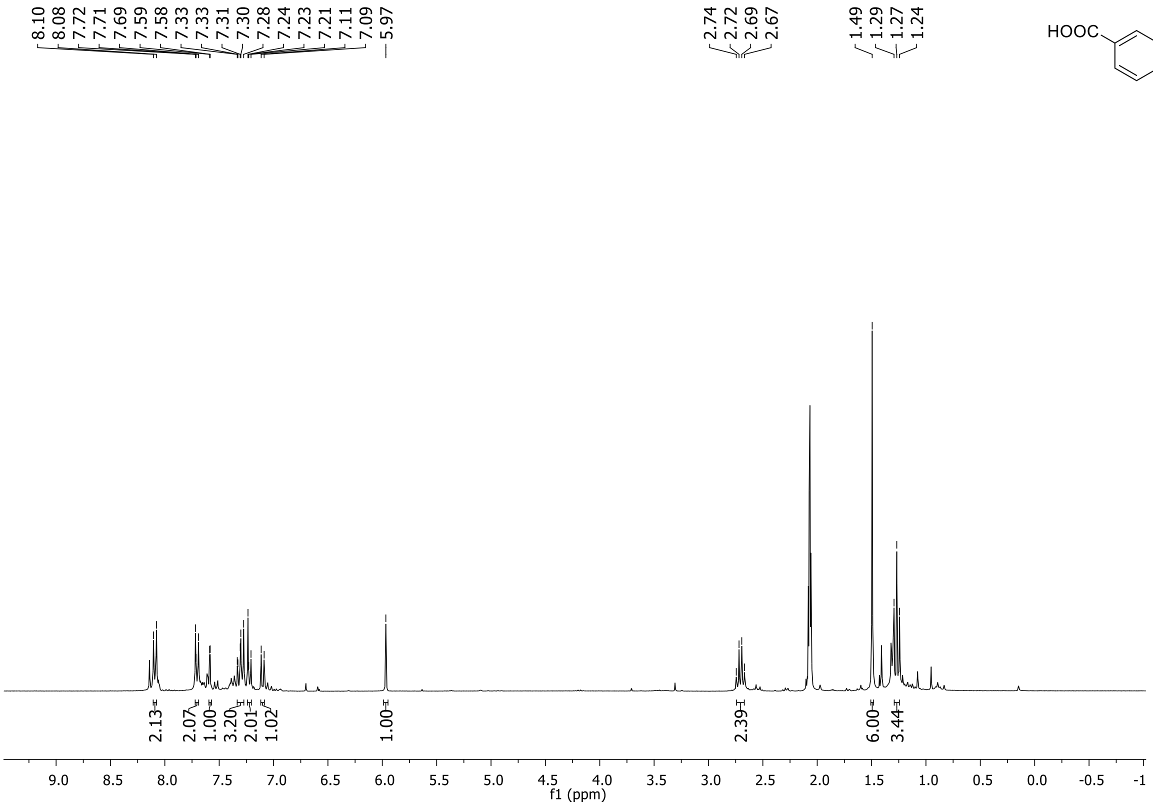

^{13}C{^{1}H} NMR (75.4 MHz, $CDCl_3$)

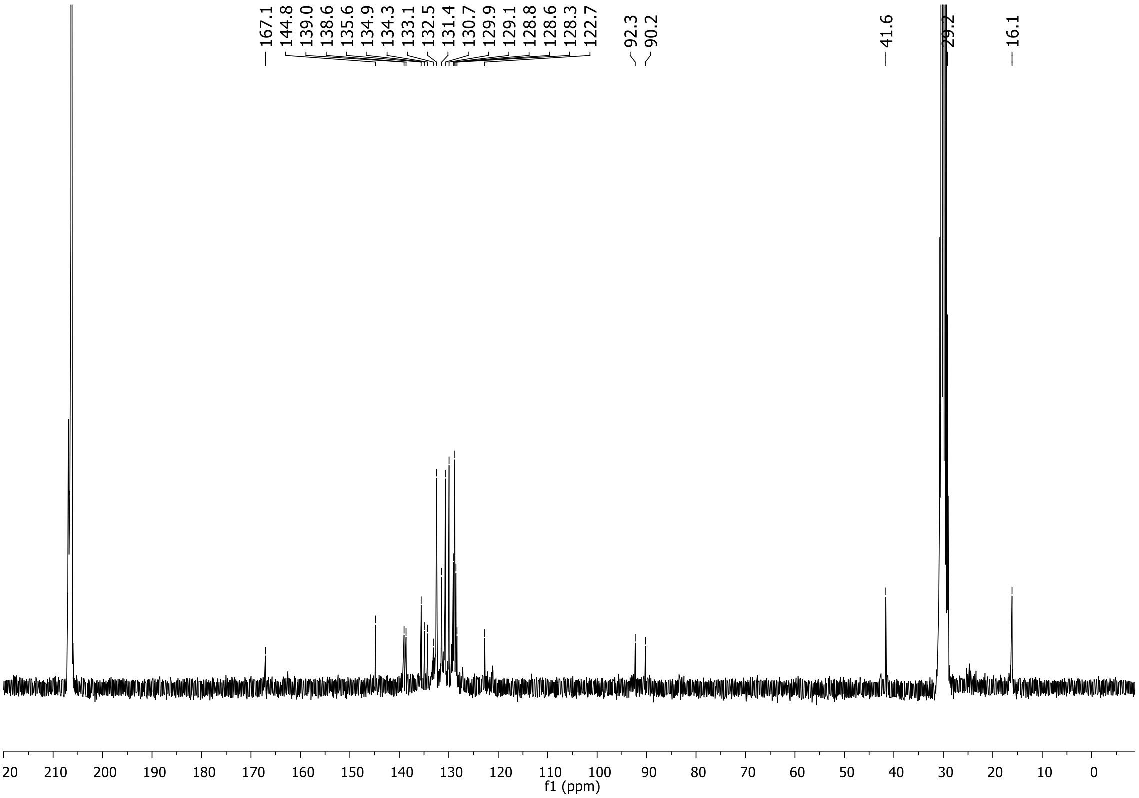

Supplement: Supplementary file 1 — jo1c00333_si_001.pdf [file jo1c00333_si_001.pdf]
