# Supplementary figures and images for: From Propargylic Alcohols to Substituted Thiochromenes: gem-Disubstituent Effect in Intramolecular Alkyne Iodo/hydroarylation
Source: J Org Chem. 2021 Apr 30;86(10):7078–91. doi: 10.1021/acs.joc.1c00333 (PMC8474117; doi:10.1021/acs.joc.1c00333)

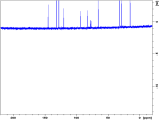

Supplement: Supplementary file 2 — jo1c00333_si_002.zip [file jo1c00333_si_002.zip › FIDS support/11/11 - C/pdata/1/thumb.png]

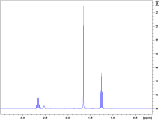

Supplement: Supplementary file 2 — jo1c00333_si_002.zip [file jo1c00333_si_002.zip › FIDS support/11/11 - H/pdata/1/thumb.png]

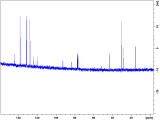

Supplement: Supplementary file 2 — jo1c00333_si_002.zip [file jo1c00333_si_002.zip › FIDS support/12/12 - C/pdata/1/thumb.png]

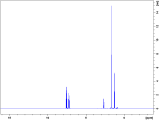

Supplement: Supplementary file 2 — jo1c00333_si_002.zip [file jo1c00333_si_002.zip › FIDS support/12/12 - H/pdata/1/thumb.png]

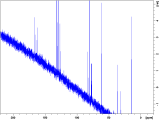

Supplement: Supplementary file 2 — jo1c00333_si_002.zip [file jo1c00333_si_002.zip › FIDS support/13/13 - C/pdata/1/thumb.png]

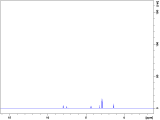

Supplement: Supplementary file 2 — jo1c00333_si_002.zip [file jo1c00333_si_002.zip › FIDS support/13/13 - H/pdata/1/thumb.png]

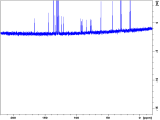

Supplement: Supplementary file 2 — jo1c00333_si_002.zip [file jo1c00333_si_002.zip › FIDS support/14/14 - C/pdata/1/thumb.png]

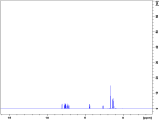

Supplement: Supplementary file 2 — jo1c00333_si_002.zip [file jo1c00333_si_002.zip › FIDS support/14/14 - H/pdata/1/thumb.png]

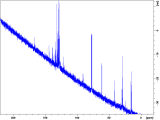

Supplement: Supplementary file 2 — jo1c00333_si_002.zip [file jo1c00333_si_002.zip › FIDS support/15/15 - C/pdata/1/thumb.png]

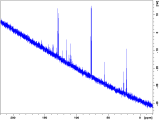

Supplement: Supplementary file 3 — jo1c00333_si_003.zip [file jo1c00333_si_003.zip › NMR_2/7ka/3/pdata/1/thumb.png]

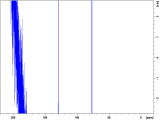

Supplement: Supplementary file 3 — jo1c00333_si_003.zip [file jo1c00333_si_003.zip › NMR_2/7ka/2/pdata/1/thumb.png]

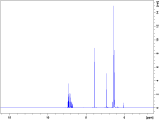

Supplement: Supplementary file 3 — jo1c00333_si_003.zip [file jo1c00333_si_003.zip › NMR_2/7ka/1/pdata/1/thumb.png]

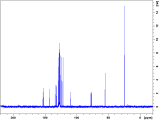

Supplement: Supplementary file 3 — jo1c00333_si_003.zip [file jo1c00333_si_003.zip › NMR_2/7aj/3/pdata/1/thumb.png]

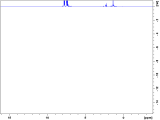

Supplement: Supplementary file 3 — jo1c00333_si_003.zip [file jo1c00333_si_003.zip › NMR_2/7aj/2/pdata/1/thumb.png]

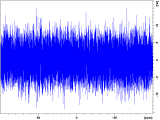

Supplement: Supplementary file 3 — jo1c00333_si_003.zip [file jo1c00333_si_003.zip › NMR_2/7aj/1/pdata/1/thumb.png]

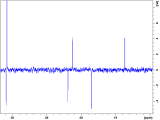

Supplement: Supplementary file 3 — jo1c00333_si_003.zip [file jo1c00333_si_003.zip › NMR_2/4al/9/pdata/1/thumb.png]

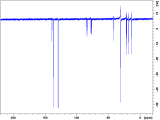

Supplement: Supplementary file 3 — jo1c00333_si_003.zip [file jo1c00333_si_003.zip › NMR_2/4al/8/pdata/1/thumb.png]

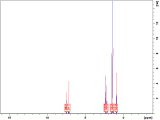

Supplement: Supplementary file 3 — jo1c00333_si_003.zip [file jo1c00333_si_003.zip › NMR_2/4al/7/pdata/1/thumb.png]
